# Genome of the house fly, *Musca domestica* L., a global vector of diseases with adaptations to a septic environment

**DOI:** 10.1186/s13059-014-0466-3

**Published:** 2014-10-14

**Authors:** Jeffrey G Scott, Wesley C Warren, Leo W Beukeboom, Daniel Bopp, Andrew G Clark, Sarah D Giers, Monika Hediger, Andrew K Jones, Shinji Kasai, Cheryl A Leichter, Ming Li, Richard P Meisel, Patrick Minx, Terence D Murphy, David R Nelson, William R Reid, Frank D Rinkevich, Hugh M Robertson, Timothy B Sackton, David B Sattelle, Francoise Thibaud-Nissen, Chad Tomlinson, Louis van de Zande, Kimberly KO Walden, Richard K Wilson, Nannan Liu

**Affiliations:** Department of Entomology, Comstock Hall, Cornell University, Ithaca, NY 14853 USA; The Genome Institute, Washington University School of Medicine, St Louis, MO 63108 USA; Evolutionary Genetics, Center for Ecological and Evolutionary Studies, University of Groningen, Groningen, 9747 The Netherlands; Institute of Molecular Life Sciences, University of Zurich, Zurich, 8057 Switzerland; Department of Molecular Biology and Genetics, Cornell University, Ithaca, NY 14853 USA; Department of Entomology, University of Illinois at Urbana-Champaign, 505 S. Goodwin Ave, Urbana, IL 61801 USA; Department of Biological and Medical Sciences, Faculty of Health and Life Sciences, Oxford Brookes University, Oxford, OX3 0BP UK; Department of Entomology and Plant Pathology, Auburn University, Auburn, AL 36849 USA; Department of Biology and Biochemistry, University of Houston, Houston, TX 77204 USA; NCBI/NLM/NIH/DHHS, 45 Center Drive, Room 5AS.43D-82, Bethesda, MD 20892 USA; Department of Microbiology, Immunology and Biochemistry, University of Tennessee Health Science Center, Memphis, TN 38163 USA; Department of Organismic and Evolutionary Biology, Harvard University, Cambridge, MA 02139 USA; Department of Medicine, Wolfson Institute for Biomedical Research, University College London, Gower Street, London, WC1E 6BT UK; NCBI/NLM/NIH/DHHS, 45 Center Drive, Room 4AS.37D-82, Bethesda, MD 20892 USA

## Abstract

**Background:**

Adult house flies, *Musca domestica* L., are mechanical vectors of more than 100 devastating diseases that have severe consequences for human and animal health. House fly larvae play a vital role as decomposers of animal wastes, and thus live in intimate association with many animal pathogens.

**Results:**

We have sequenced and analyzed the genome of the house fly using DNA from female flies. The sequenced genome is 691 Mb. Compared with *Drosophila melanogaster*, the genome contains a rich resource of shared and novel protein coding genes, a significantly higher amount of repetitive elements, and substantial increases in copy number and diversity of both the recognition and effector components of the immune system, consistent with life in a pathogen-rich environment. There are 146 P450 genes, plus 11 pseudogenes, in *M. domestica*, representing a significant increase relative to *D. melanogaster* and suggesting the presence of enhanced detoxification in house flies. Relative to *D. melanogaster*, *M. domestica* has also evolved an expanded repertoire of chemoreceptors and odorant binding proteins, many associated with gustation.

**Conclusions:**

This represents the first genome sequence of an insect that lives in intimate association with abundant animal pathogens. The house fly genome provides a rich resource for enabling work on innovative methods of insect control, for understanding the mechanisms of insecticide resistance, genetic adaptation to high pathogen loads, and for exploring the basic biology of this important pest. The genome of this species will also serve as a close out-group to *Drosophila* in comparative genomic studies.

**Electronic supplementary material:**

The online version of this article (doi:10.1186/s13059-014-0466-3) contains supplementary material, which is available to authorized users.

## Background

House flies, *Musca domestica* L. (Diptera: Muscidae), are ubiquitous and transmit more than 100 human and animal diseases [1-3], including bacterial infections such as salmonellosis, anthrax, ophthalmia, shigellosis, typhoid fever, tuberculosis, cholera and infantile diarrhea; protozoan infections such as amebic dysentery; helminthic infections such as pinworms, roundworms, hookworms and tapeworms; as well as viral and rickettsial infections. House flies can spread a deadly strain of *Escherichia coli* [4] and transmit life threatening antibiotic-resistant bacteria [5,6], which constitute an ever increasing threat in hospitals and other healthcare facilities [7-10]. Flies also transmit pathogens responsible for eye diseases such as trachoma and epidemic conjunctivitis, and infect wounds or skin with diseases such as cutaneous diphtheria, mycoses, yaws and leprosy [2]. Fly-transmitted trachoma alone causes 6 million cases of childhood blindness each year [11]. The mobility of house flies, their regular contact with excreta, carcasses, garbage and other septic matter, and intimate association with animal pathogens and humans all contribute to their roles in transmission of these diseases [1,2]. House fly larvae play a vital role in ecosystems as decomposers of animal wastes. This represents a unique niche, relative to other insects that have had their genomes sequenced.

House flies are always found in association with humans and human activities, following the spread of *Homo sapiens* across the planet [12]. They are also one of the most serious pests at animal production facilities worldwide. Insecticides have been used extensively for controlling house flies for a century and this pest has shown a remarkable ability to rapidly evolve resistance. This led to house flies being one of the primary insects used for studies on insecticide resistance and toxicology.

The house fly has been a model system for studies of insect olfaction [13,14] and (Z)-9-tricosene plays an important role in inter-sex communication and mate selection in house flies. New attractants would be valuable for baits in management systems [15,16] and could lead to a reduction in insecticide use for house fly control.

*Nasonia vitripennis* is a parasitoid of the house fly (*Nasonia* is sold commercially for fly control) and the *Nasonia* genome has been sequenced [17]. Having the genome of both the parasitoid (*Nasonia*) and the host (*M. domestica*) will allow unprecedented insights into the molecular mechanisms of host-parasitoid interaction.

The Diptera clade has radiated into over 120,000 known species since its origin in the late Jurassic. *M. domestica* is well placed within the Diptera to maximize the utility of sequence data for comparison between existing dipteran genomes [18]. Multiple, deeply divergent comparisons within the order allow identification of lineage effects on rates and patterns of genomic diversity. These comparisons become more powerful in elucidating genome evolution as the phylogenetic context is broadened. Given the well centered position between *Drosophila* and mosquitoes, the *Musca* genome is nearly ideal for leveraging analysis and annotation of the mosquito genomes [18].

*M. domestica* has a well described linkage map for the five autosomes (I to V) and two sex chromosomes (X and Y) [19-23]. In the house fly, male sex is determined by a dominant factor, M, which is located on the Y chromosome in 'standard' populations. Thus, males are XY^M^ and females are XX [24,25]. This is believed to be the ancestral state of sex determination in house flies [26,27]. However, M can be located on one or more of the five autosomes [28-34] or even rarely on X [26,35]. The former M factors are referred to as 'autosomal M' and both males and females in carrier populations typically have the XX genotype [24,26,29,36]. Autosomal M factors act similarly to Y chromosome M factors in determining maleness, by prohibiting the female specific splicing of the *transformer* (*Md-tra*, previously F) primary transcript [28,35]. In autosomal M populations from different continents, especially those having males with multiple M factors, a *Md-tra* variant was found, *Md-tra*^*D*^ (previously *F*^*D*^), which acts as a dominant female determiner. It allows females to be produced even in the presence of multiple copies of M (or a homozygous M) and effectively makes females the heterogametic sex (M/M; *Md-tra*^*D*^/*Md-tra*^*+*^), and males the homogametic sex (M/M; *Md-tra*^*+*^/ *Md-tra*^*+*^) [28,34]. The genome sequence will accelerate progress in understanding many of these and other aspects of house fly biology.

## Results and discussion

### Sequencing and assembly

Genomic DNA of a pool of six adult female flies was sequenced and assembled to a size of 0.691 Gb, comprising 20,487 scaffolds (N50 contig, 12 kb; N50 scaffold, 226 kb). Scaffolds ranged in length up to a maximum of 2.29 Mb (Table 1). This genome size of 0.691 Gb is 81% of the size estimated using kmer frequency plus depth of coverage calculations, 75% of the size determined spectrophotometrically [37] and 200% of the size estimated using quantitative PCR [38], respectively. More than half (52%) of the *M. domestica* genome is composed of interspersed repeats, suggesting a novel genome evolution trajectory compared with *Drosophila melanogaster* (Additional file 1). A majority of these repeat elements (representing 25% of the genome) are those that transpose by DNA excision and repair, class II or DNA transposons. Using the NCBI annotation pipeline and RNA-seq transcript evidence, we predicted a total of 14,180 protein-coding genes and 1,165 non-coding genes (Table 2). Alignment of 550 *M. domestica* transcripts (GenBank) to the assembly showed that 95% align over at least 90% of their length, and of 248 aligned universal single copy orthologs (CEGMA), 98% were complete, suggesting that the assembly has captured most of the protein-coding genes in the genome. A measure of aggregate transcript coverage by alignment of whole body and larva RNA-seq data to our *M. domestica* reference was 66%. This measure of transcript coverage varies (66 to 94%) among insect genomes as a result of assembly contiguity, dictated by repeat composition (Additional file 2). The average protein identity in comparison to *D. melanogaster* (RefSeq) was 64%. In addition to the RNA-seq data generated in our study, we also compared the *M. domestica* genome with a previously published transcriptome of *M. domestica* [39]*.* Of the 6,159 transcripts previously reported, 6,053 had matches in the *ab initio* predicted genes in *M. domestica* when compared using BLASTn [40]. The average percent identity was 98.7 ± 0.02% and ranged from 75.2 to 100%, identical to the *ab initio* predicted gene set (Additional file 3). The remaining 106 transcripts previously reported [39] were further compared with the genome using Exonerate [41] and had, on average, 97.6 ± 0.9% identity, ranging from 38.6 to 100% identical (Additional file 3).

**Table 1:** **House fly genome assembly scaffold length distribution**

**Scaffold length**	**Scaffold count**
>1 Mb	35
250 kb to 1 Mb	604
100 to 250 kb	1,082
10 to 100 kb	4,640
5 to 10 kb	2,584
2 to 5 kb	6,000
<2 kb	5,542

**Table 2:** **Genic characteristics of the house fly genome**

**Feature**	**Count**	**Mean length**
Genes	15,345	13,553
Transcripts	18,779	2,097
mRNA	17,508	2,221
tRNA	947	74
Single exon transcripts	2,566	797
Exons	67,886	431
Introns	52,875	3,889

We grouped the 14,180 predicted protein-coding genes in *M. domestica* into 10,427 orthologous groups based on homology to *D. melanogaster*. Each orthologous group contains at least one *M. domestica* protein, and can be assigned to one of three categories: single copy ortholog, for groups that contain a single *M. domestica* protein and a single *D. melanogaster* protein, conserved paralog, for groups that contain both *M. domestica* and *D. melanogaster* proteins, but are not single copy in both species, and lineage-restricted, for groups that contain only *M. domestica* proteins. Of the 14,180 predicted protein-coding genes, 7,006 (49%) are single copy orthologs of *D. melanogaster* proteins, 5,240 (37%) are in conserved paralogous groups (mean size in *M. domestica*: 2.88 proteins), and the remaining 1,934 (14%) are lineage-restricted and lack homologs in *D. melanogaster*.

### Gene ontology

The most abundant Gene Ontology (GO) biological processes represented by house fly genes were single-organism process (12.1%), cellular process (12.0%), metabolic process (11.1%) and biological regulation (10.8%; Figure S1A in Additional file 4). The most abundant cellular components were cell (32.6%) and organelle (29.2%; Figure S1B in Additional file 4). The most abundant molecular processes were binding (48.1%) and catalytic activity (28.9%; Figure S1C in Additional file 4). Overall, the distribution of genes within GO classifications was very similar between *M. domestica* and *D. melanogaster* (Additional file 5). Within the biological processes classification, the most notable difference was the more than two-fold greater percentage of genes in *M. domestica* associated with 'growth'. Within the cellular components classification *M. domestica* had an approximately two-fold greater percentage of genes in the 'membrane', and 'membrane-enclosed lumen' categories (relative to *D. melanogaster*). *M. domestica* also had a lower percentage of genes in the 'cell' category, relative to *D. melanogaster*. There were no remarkable differences between *M. domestica* and *D. melanogaster* in the percentage of genes in the molecular functions categories (Additional file 5).

### Immune-related genes

We identified 771 putative immune-related genes in the house fly, of which 416 have direct homology (see Materials and methods) to known or putative *D. melanogaster* immune-related proteins, and the remaining 355 are identified based on hidden Markov model (HMM) queries. As in other insects, these genes encode proteins with several functions: recognition proteins that identify pathogen-associated molecular patterns, proteins that belong to signaling pathways that activate the transcriptional response to infection, and effector molecules that kill pathogens.

Previous work, primarily in *Drosophila*, has identified four primary signaling pathways involved in the systemic transcriptional response to bacterial and fungal infection: the Toll, imd, JAK/STAT, and JNK pathways [42,43]. The *M. domestica* genome possess the full complement of signal transduction or signal modulation components of these pathways, and the vast majority are conserved as single copy orthologs between *M. domestica* and *D. melanogaster*. It appears likely, therefore, that immune signaling in *M. domestica* is substantially similar to immune signaling in *D. melanogaster.*

**Figure 1:**
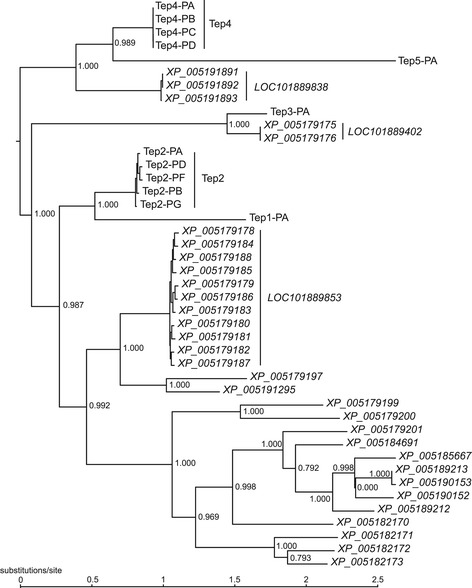
**Tep phylogeny.** Maximum likelihood amino acid phylogeny of *D. melanogaster* and *M. domestica* Teps. *D. melanogaster* proteins are labeled Tep1 to Tep5. *M. domestica* Teps are labeled XP_NNNNNNNNN and are shown in italics. For both species, multiple isoforms of the same gene are indicated with a bar. Branch support is the SH-like statistic estimated in phyml. The tree is rooted so as to minimize the number of duplications.

In contrast to the signaling pathways, both recognition and effector components of the immune system show substantial increases in copy number and genic diversity in *M. domestica* compared with *D. melanogaster*, suggesting the possibility that *M. domestica* possesses a more robust immune response to diverse pathogens encountered in the pathogen-rich environment in which it lives. In other insects, a variety of cell-surface and secreted proteins involved in recognition of pathogens have been identified, including peptidoglycan recognition proteins and beta-glucan binding proteins (also known as GNBPs in *Drosophila*), which are upstream of the main signaling pathways [44], and a variety of receptors likely involved in phagocytosis, including Nimrods, thioester-containing proteins (Teps) and scavenger receptors [45,46]. Of these, there are striking expansions in copy number of Nimrods and Teps in particular. The Nimrod gene family is one of the more variable in copy number among the sequenced *Drosophila* species [47], a trend that is continued in the more divergent comparison to *M. domestica* (17 Nim-containing proteins in *M. domestica* and 11 in *D. melanogaster*; only 8.7% of gene families have a greater degree of expansion in *M. domestica*). Even more striking is the expansion of the Tep family (Figure 1), which has 19 members in *M. domestica* and only 6 in *D. melanogaster*. The Tep1/2 gene family specifically has expanded dramatically in *M. domestica*: this family has 2 members in *D. melanogaster* but 16 members in *M. domestica*, which is the 15th largest species-specific expansion in the *M. domestica* genome (Additional file 1). In addition, the *M. domestica* genome contains three lineage-specific genes that encode proteins with Tep-like domains (identified by HMM), but which are not clearly homologous to any characterized *D. melanogaster* Teps.

In *M. domestica*, similar to recognition proteins, the effector molecule repertoire is also significantly expanded*. M. domestica* shares four antimicrobial families with *D. melanogaster*, the attacins, diptericins, cecropins, and defensins (*D. melanogaster* also possesses one drosocin, one metchnikowin, and seven drosomycins that are not identifiable in the *M. domestica* genome), three of which have expanded relative to *D. melanogaster* (10 attacins in *M. domestica*, 4 in *D. melanogaster*; 12 cecropins in *M. domestica*, 5 in *D. melanogaster* (including andropin); 5 defensins in *M. domestica*, 1 in *D. melanogaster*; 2 diptericins in *M. domestica*, 2 in *D. melanogaster*). Even including the 9 antimicrobial peptides in *Drosophila*-specific families, *M. domestica* has a significantly enlarged antimicrobial peptide (AMP) repertoire (29 versus 19). AMP gene families are known to evolve very rapidly [48], and it is likely that novel effectors remain to be discovered in *M. domestica* (as in mosquitos [49], bees [50], and wasps [51,52]), further increasing the potential diversity of the house fly AMP response.

### Metabolism/detoxification genes

**Figure 2:**
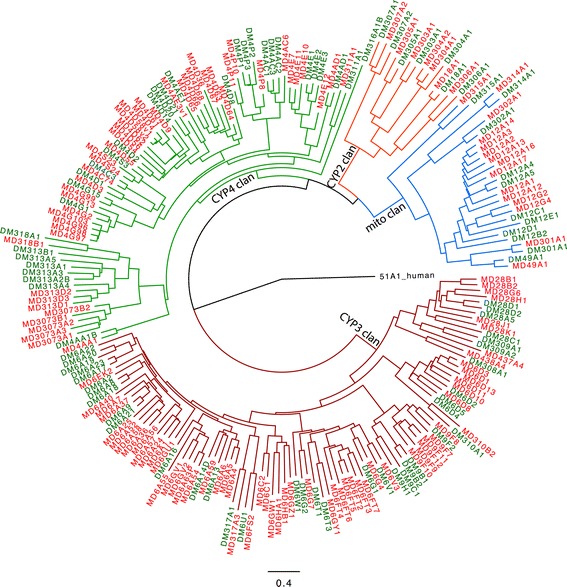
**Neighbor-joining tree showing the phylogenetic analysis of cytochrome P450 (CYP) genes of** ***M. domestica*** **(MD, red) in relation to CYP genes from** ***D. melanogaster*** **(DM, green).** Clustal W was used to perform multiple sequence alignment and Phylip was used to compute the tree. The tree was rooted with human CYP51 as an out-group. The four insect CYP clades are shown in different colors. Sequences for the cytochrome P450 genes and the gene names were taken from the DR Nelson P450 homepage [59].

Three groups of enzymes are commonly associated with detoxification of xenobiotics (although they have other functions as well [53]): cytochrome P450s, esterases/hydrolases, and conjugation enzymes. The largest group in *M. domestica* is the cytochrome P450s, for which a total of 146 genes plus 11 pseudogenes were identified (Additional file 6). This represents a significant expansion of P450s relative to *D. melanogaster*, which has 86 [54], and relative to *Glossina morsitans*, which has 72 (Additional file 7). Most of the predicted cytochrome P450 genes (135 genes) were full length while 11 genes were incomplete, or were contained within multiple predicted transcripts, six of which, *CYP6A56*, *CYP6GV1*, *CYP6A36*, *CYP304A2*, *CYP310B2*, and *CYP313D1*, had partial sequences either due to low sequence coverage or because the predicted gene spanned the edge of a supercontig (Additional file 8). One cytochrome P450 gene, *CYP4D68*, was predicted to have an alternative amino terminus (XM_005190900) upstream of the main cytochrome P450 predicted gene locus (XM_005190901), and four other P450s genes (*CYP4D3*, *CYP4D4*, *CYP4D58*, *CYP4AC6*) may have alternative splicing isoforms as well (Additional file 8). The remaining four cytochrome P450 genes, *CYP6A6*, *CYP6A58*, *CYP6D1*, and *CYP4D64*, were represented by more than one predicted transcript that either spanned the edges of different supercontigs or were positioned proximal to each other within the genome (Additional file 8). The expansion of cytochrome P450 genes in *M. domestica* was predominantly present within clans 3 and 4, which had 65 and 55 genes, respectively (Figure 2). The most predominant P450 families in *M. domestica* were CYP6 and CYP4, which contained 46 and 43 genes, respectively, and represented >60% of all cytochrome P450s in the *M. domestica* genome. This is a similar percentage to what is present in the *D. melanogaster* genome, in which the genes in the CYP6 and CYP4 families account for 50% of the total cytochrome P450 genes in the genome [55]. The Halloween genes, *CYP306A1* (*phantom*), *CYP302A1* (*disembodied*), *CYP307A2* (*spookier*), *CYP315A1* (*shadow*), and *CYP314A1* (*shade*), were identified [56], along with *CYP18A1*, which is involved in the inactivation of 20-hydroxyecdysone [57], and *CYP301A1*, which has recently been shown to be important for cuticle formation [58]. Taken together, the complement of cytochrome P450 genes identified in the *M. domestica* genome consist of those anticipated to be present, along with a predominance of cytochrome P450 genes from the CYP6 and CYP4 families as seen in *D. melanogaster*.

A total of 33 glutathione *S*-transferase (GST) genes and 3 splice variants were predicted from the *M. domestica* genome (Additional file 9). This is similar to the number present in *D. melanogaster*, which has 36 GST genes and 11 splice variants [60]. An unrooted phylogenetic analysis of the GSTs for *M. domestica* and *D. melanogaster* showed that the *M. domestica* GST genes were distributed among the different classes of cytosolic GSTs present in the *D. melanogaster* genome: epsilon, omega, theta, sigma, and zeta (Additional file 10). An additional four microsomal GST genes were predicted from the *M. domestica* genome, which is similar to the number of microsomal GST genes present in *D. melanogaster* (three genes with a total of four isoforms).

In the *M. domestica* genome, a total of 92 genes were predicted to have esterase activities, including phosphodiesterase, acetylcholinesterase, thioesterase, carboxylesterase, metallophosphoesterase, neuropathy target esterase and palmitoyl-protein thioesterase (Additional file 5). Based on the chemical reactions they catalyze, these enzymes were divided into four categories, containing a total of 39 carboxylic-ester hydrolases (EC 3.1.1), 10 thioester hydrolases (EC 3.1.2), three phosphoric-monoester hydrolases (EC 3.1.3) and 40 phosphoric-diester hydrolases (EC 3.1.4) [61].

### Cys-loop ligand-gated ion channels

**Figure 3:**
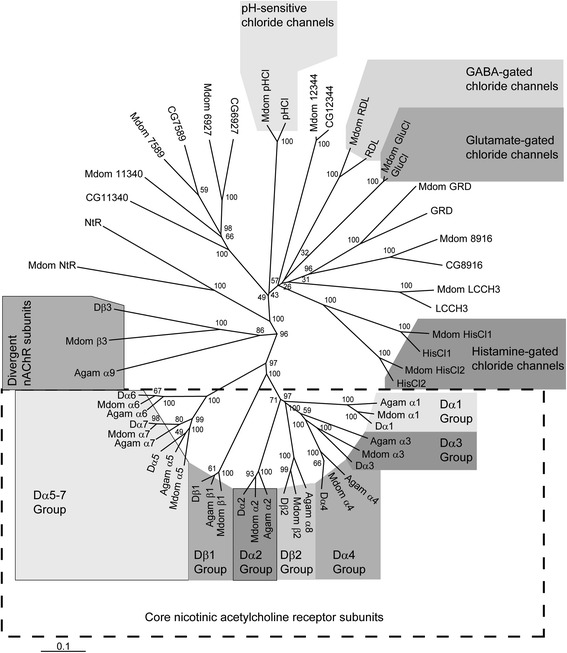
**Phylogeny showing relationships of** ***M. domestica*** **and** ***D. melanogaster*** **cysLGIC protein sequences.** *Anopheles gambiae* sequences were also included when comparing nAChR sequences. Numbers at each node signify bootstrap values with 100 replicates and the scale bar represents substitutions per site. Genbank ccession numbers for sequences shown in the tree are: *A. gambiae* Agamα1 (AY705394), Agamα2 (AY705395), Agamα3 (AY705396), Agam α4 (AY705397), Agamα5 (AY705399), Agamα6 (AY705400), Agamα7 (AY705402), Agamα8 (AY705403), Agamαβ9^α^ (AY705404) and Agamβ1 (AY705405); *D. melanogaster* Dα1 (CAA30172), Dα2 (CAA36517), Dα3 (CAA75688), Dα4 (CAB77445), Dα5 (AAM13390), Dα6 (AAM13392), Dα7 (AAK67257), Dβ1 (CAA27641), Dβ2 (CAA39211), Dβ3 (CAC48166), GluCl (AAG40735), GRD (Q24352), HisCl1 (AAL74413), HisCl2 (AAL74414), LCCH3 (AAB27090), the putative cysLGIC subunit Ntr (AF045471), pHCl (NP_001034025), RDL (AAA28556), CG6927 (AAF45992), CG7589 (AAF49337), CG8916 (BT022901), CG11340 (AAF57144), CG12344 (AAF58743); *M. domestica* Mdomα2 (DQ372062), Mdomα5 (EF203213), Mdomα6 (DQ498130), Mdomβ3 (EF203220), MdomRDL (Q75NA5), MdomGluCl (BAD16657). GABA, γ-aminobutyric acid.

Members of the cys-loop ligand-gated ion channel (cysLGIC) superfamily mediate fast synaptic transmission in insects. They play key roles in behavior, such as escape responses [62], olfactory learning and memory [63], as well as regulating sleep [64]. CysLGICs consist of five homologous subunits arranged around a central ion channel [65]. Analysis of the *M. domestica* genome has revealed 23 subunit-encoding genes, which is the same complement of genes found in *D. melanogaster* [66] (Figure 3). Ten of these genes encode putative nicotinic acetylcholine receptor (nAChR) subunits, which consist of a core group of subunit-encoding genes (α1 to α7 and β1 to β2) [67] that are highly conserved between insect species, four of which (α2, α5, α6 and β3) have been characterized from *M. domestica* [68-70]. The *M. domestica* genome also contains a single divergent subunit (β3) that is less well conserved [70]. For nAChRs, α subunits are traditionally defined by the presence of two vicinal cysteine residues important for interactions with acetylcholine, while β subunits lack this motif [71]. The putative *M. domestica* ortholog of Dβ2 is a non-α subunit (Mdomβ2; Figure 3), which is unusual considering that the orthologs of Dβ2 in other insect species possess the cysteine doublet and thus are α subunits, including Agamα8 from another member of the Diptera, the mosquito *Anopheles gambiae* [72]. The amino acid sequences and accession numbers for the cysLGICs are provided in Additional file 11.

The house fly cysLGIC superfamily also includes Rdl [73], GRD and LCCH3, which form ion channels gated by γ-aminobutyric acid (GABA) [74], a glutamate-gated chloride channel (GluCl) [73], two histamine-gated chloride channels (HisCl1 and HisCl2) [75], and a pH-sensitive chloride channel (Figure 3) [76]. The remaining cysLGICs have yet to be functionally characterized. *M. domestica* is the only other insect reported to possess a putative ortholog of *D. melanogaster* cysLGIC, NtR. Insect cysLGICs are of importance as they are targets of widely used insecticides [77], such as phenylpyrazoles (which act on GABA receptors and GluCls), spinosyns and neonicotinoids (which act on nAChRs). The cysLGIC sequence information from diverse species, including agricultural pests, disease vectors and pollinating insects [78-80], provides a valuable starting point for understanding the interactions of insecticides with their targets at the molecular level, as well as enhancing our understanding of mechanisms causing insecticide resistance, and may prove instructive in the future design and development of improved insecticides with enhanced specificity for pest species.

### Chemoreceptors

The olfactory and gustatory abilities of insects depend on many chemoreceptors and associated proteins encoded by at least four major gene families [81]. The odorant binding proteins (OBPs) are small, globular, secreted proteins that transport hydrophobic odorants to the receptors in sensory neuron membranes within sensory sensilla, primarily on the antennae, but also on the maxillary palps and other chemosensory organs [82]. The odorant receptors (ORs) are a relatively recently evolved family within the insect chemosensory superfamily of ligand-gated ion channels that mediate much of olfaction in insects [83]. The gustatory receptors (GRs) mediate much of gustation, especially perception of sugars and bitter tasting compounds, but as the basal family of highly divergent receptors within the superfamily, also mediate some aspects of olfaction, such as perception of carbon dioxide [84]. The ionotropic receptors (IRs) are a greatly expanded and divergent family of chemoreceptors that evolved from the ionotropic glutamate receptor superfamily in basal animals, and while some function in olfaction, many are involved in gustation [85].

As the obvious comparison for the *M. domestica* repertoire, *D. melanogaster* has 52 genes encoding OBPs [86], 60 genes encoding 62 ORs and 60 genes encoding 68 GRs via alternative splicing of some loci [87], and 65 genes encoding IRs [85]. We find that the *M. domestica* genome encodes at least 87 OBPs, has 85 genes encoding 86 ORs, 79 genes encoding 103 GRs, and 110 IRs (Additional files 12, 13, 14, 15, and 16). Detailed examination of the relationships of these gene families in these two flies (Additional files 17, 18, 19, and 20) reveals the expected patterns of birth-and-death gene family evolution typical of environmentally relevant genes. As expected, *M. domestica* shares the highly conserved members of these families, such as the OrCo protein that functions with each specific OR to make a functional olfactory receptor, and the apparently equivalent IR8a/25a proteins, along with the conserved suites of sugar and carbon dioxide receptors, and some highly conserved bitter taste receptors. In general, however, while there are roughly equal numbers of gene losses and pseudogenes in each species, *M. domestica* has duplicated and retained more genes in each family. These gene subfamily expansions are particularly prominent in the candidate gustatory receptors, especially those implicated in perception of bitter tasting compounds. For example, *M. domestica* has a large, mostly tandem array of 26 genes, one of which is alternatively spliced to encode 11 receptors, that are related to 8 *D. melanogaster* GRs implicated in bitter taste that are now spread around that genome. The expansions in the IR family are also primarily in receptors implicated in gustation. *M. domestica* also has large expansions of ORs related to Or45a and Or67d in *D. melanogaster*, receptors involved in repulsion from aversive chemicals in larvae and in perception of a male-produced pheromone, respectively.

In summary, *M. domestica* has evolved an expanded repertoire of chemoreceptors and associated proteins compared with *D. melanogaster*. This expansion is mostly associated with gustation, specifically perception of bitter tasting compounds. It may be that the more diverse and potentially toxic food sources and larval habitats of *M. domestica* have led to retention and specialization of gene duplicates in these receptor gene subfamilies. Unfortunately, the ligand specificity of most candidate bitter taste receptors in *Drosophila* have proven difficult to resolve, so it is not yet possible to infer likely ligands for these novel receptors in *M. domestica*.

### Sex determination, sex-biased gene expression and the evolution of sex-biased genes

**Figure 4:**
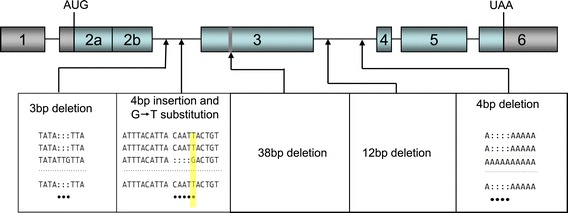
**Molecular lesions in** ***Md-tra*** ^***D***^ **alleles of different populations.** Schematic organization of exons in *Md-tra*. Exon 2b and/or exon 3 are included in male *Md-tra* transcripts and cause premature termination of translation due to the presence of in-frame translational termination signals. In females these exon sequences are skipped, giving rise to transcripts with an intact open reading frame. The positions of the reported *Md-tra* ^*D*^ sequence variations are indicated by arrows and further details are described in the boxes below [88].

The dominant female determining *Md-tra*^*D*^ (Genbank accession GU070694) allele sampled from a Japanese population contains four small deletions and a small insertion in the alternatively spliced sequences and one non-synonymous substitution in the coding region [88]. We sequenced *Md-tra*^*D*^ alleles of 22 to 24 individuals from 7 populations sampled across Europe, North America, Asia, Africa and Australia and found *Md-tra*^*D*^ alleles on all continents. Surprisingly, we detected exactly the same molecular signatures in *Md-tra*^*D*^ alleles of all populations tested (Figure 4), but different alleles for the non-dominant form, containing insertions or deletions in exon 3. Further studies will be necessary to elucidate the cause for its rapid global dispersion and fixation in autosomal populations. Availability of the genome sequence will facilitate such studies; particularly scans of the *Md-tra* locus can be conducted to look for more variants.

Genes with sexually dimorphic (sex-biased) expression, much like other sexually dimorphic traits, tend to evolve faster than genes with equal (unbiased) expression in males and females [89,90]. Among genes with sex-biased expression, genes expressed in reproductive tissues evolve fastest, particularly those expressed primarily in male-limited organs [91]. The faster evolution of genes with sex-biased expression is likely driven by a combination of positive Darwinian selection and relaxed purifying selection [89,92]. Genes with higher expression in males (male-biased) are also more likely than unbiased genes to not have identifiable homologs in comparisons between *Drosophila* species [89,93], suggesting that genes with male-biased functions are more dispensable or that their protein coding sequences evolve too fast for homolog identification [92].

We used RNA-seq to measure gene expression levels in whole male and female adult flies, and we identified genes with male- or female-biased expression (Additional file 21). Out of 10,096 genes with sufficiently high expression to allow a test contrasting the sexes, 113 have male-biased expression and 81 have female-biased expression (Table 3). There are no significant differences in GO categories between male-biased, female-biased, and unbiased genes, although this analysis is likely limited by small sample sizes. In *Drosophila* whole bodies, approximately 10 to 20% of genes have sex-biased expression [93,94], which is an order of magnitude greater than what we observe in house fly. *Drosophila* gonads and other reproductive organs make up a substantial portion of adult body mass [95,96], whereas house fly reproductive organs (especially in males) are relatively smaller [97]. Because sex-biased expression in whole bodies is driven primarily by genes that are differentially expressed between male and female reproductive tissues [98], the lower frequency of genes with sex-biased expression in house fly whole bodies could be the result of smaller reproductive organs and/or less severe sexual conflict. Alternatively, we may have less power to detect sex-biased expression in house fly due to low replication or noisy data.

**Table 3:** **Sex-biased expression of house fly genes and homology with** ***D. melanogaster*** **genes**

**Sex-bias**	**Conserved**	**Lineage-specific**	**Frequency conserved**
Male-biased	88	25	0.779
Female-biased	71	10	0.877
Unbiased	8,478	1,104	0.884

To test the hypothesis that genes with sex-biased expression experience more evolutionary turnover than unbiased genes, we used the ortholog calls described above to divide house fly genes into those with at least one *D. melanogaster* homolog (conserved) and those that are lineage-specific. Not all *M. domestica* genes tested for sex-biased expression were included in the test of homologous genes in *D. melanogaster* (and vice versa), because of how the data were handled. House fly genes with sex-biased expression are less likely to be conserved than genes with unbiased expression (*P* < 0.01, Fisher’s exact test (FET)) (Table 3). Genes with male-biased expression are less likely to be conserved than unbiased genes (*P* < 0.005, FET), whereas female-biased genes are as conserved as unbiased genes (*P* = 0.729, FET) (Table 3). While male-biased house fly genes have a lower frequency of homology matches to *D. melanogaster* than female-biased genes, this difference is not significant (*P* = 0.091, FET). These results support the hypothesis that genes with male-biased expression are gained/lost from the genome at a faster rate than other genes, and/or that genes with male-biased expression have faster evolving protein-coding sequences that evade homology detection.

**Figure 5:**
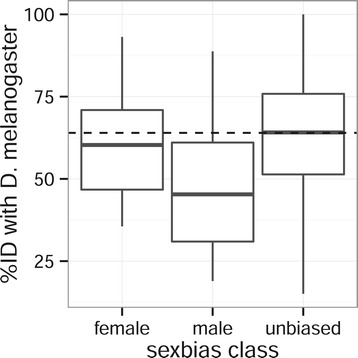
**Evolutionary divergence of sex-biased genes.** Boxplots show the amino acid sequence identity between *M. domestica* and *D. melanogaster* single copy orthologs for genes with female-biased, male-biased, and unbiased expression in house fly.

We next calculated amino acid sequence identity between house fly and *D. melanogaster* for genes that are single-copy orthologs to determine whether genes with sex-biased expression experience elevated rates of evolution (Figure 5). Genes with male-biased expression are more divergent than both female-biased (*P* < 0.005, Mann–Whitney (MW) test) and unbiased (*P* < 10^−6^, MW test) genes. There is not a significant difference in evolutionary divergence between female-biased and unbiased genes (*P* = 0.280, MW test). These results suggest that at least some of the lineage-specific house fly male-biased genes are the result of genes with male-biased expression evading homology detection because of their faster evolving protein-coding sequences.

We further divided house fly genes with *D. melanogaster* homologs into those that were duplicated specifically in the house fly lineage (remaining single-copy in *D. melanogaster*) and those that are one-to-one homologs with *D. melanogaster* genes (Table 4). Duplicated genes are more likely to have male-biased expression than single-copy genes (*P* < 0.005, FET), consistent with what has been observed in *D. melanogaster* [99]. Unlike the pattern in *D. melanogaster*, house fly duplicated genes are also more likely to have female-biased expression than single-copy genes (*P* < 0.05, FET). In general, house fly duplicated genes are more likely to have sex-biased expression than single-copy genes (*P* < 0.0005, FET), and there is no difference in duplication frequency between male- and female-biased genes (*P* = 0.665, FET). These results suggest that gene duplication is a mechanism for generating both male- and female-biased expression in house fly, which differs from the observation in *Drosophila* [99].

**Table 4:** **Sex-biased expression of house fly genes and duplication status**

**Sex-bias**	**Duplicated**	**Single-copy**	**Frequency duplicated**
Male-biased	17	42	0.288
Female-biased	12	39	0.235
Unbiased	832	5,782	0.126

### Actin

Five actin genes were found in house fly (LOC101901018, LOC101887443, LOC101890414, LOC101895248, LOC101888968), one less than found in *D. melanogaster*. The sequences were highly similar at the amino acid level (95.5 to 99.5%; Martinez-NW pairwise alignments) and the nucleotide level (84.4 to 96.8%). Like *D. melanogaster* each sequence was 1,131 nucleotides in length. The high degree of similarity between these genes suggests that the use of one of these genes as an internal standard for quantitative PCR should be carefully scrutinized to avoid detection of multiple actin genes. The deduced amino acid sequences of LOC101888968 and LOC101895248 were 100% identical to *D. melanogaster* actin 5c and 88 F, respectively. Other orthologs could not be unambiguously identified.

### MicroRNA and microRNA machinery

After mapping all of the miRbase sequences against the *M. domestica* genome, a total of 69 microRNAs (miRNAs) along with their hairpin precursors were predicted to be present in the *M. domestica* genome (Additional file 22). Seven miRNAs had two copies and two had three copies, making a total of at least 81 miRNA loci in the *M. domestica* genome. Within the multiple copy miRNAs, two miRNAs, miR-1-5p and miR-996-5p, had the same hairpin precursors, but were located in two distinct locations of the genome, suggesting a duplication event. In addition to the predicted miRNA, 25 isoforms of 11 genes were predicted to be involved in the transporting, processing and function of miRNAs, so-called miRNA machinery [100] (Additional file 23). These genes include *Drosha*, *DGCR* (partner of Drosha), *ran-like GTPase*, *exportin-5*, *Dicer*, multiple *argonaute* proteins, including one *ago-1-like* gene with four isoforms, two *ago-2-like* genes, one with two isoforms and another *ago-2-like* gene with five isoforms, and one *ago-3-like* gene, and two *RISC-loading* genes. Similar to other dipteran species, no *sid-1* homolog, which is involved in systemic miRNA, was identified in the *M. domestica* genome [101]. However, multiple genes putatively involved in the uptake of exogenous double-stranded RNA were identified; including two *eater* genes, and one *nibbler* gene (Additional file 23). Although the biological function of these miRNAs are currently unknown, the availability of bioinformatics information will provide a valuable tool for future studies targeting on the gene regulation in physiological processes [102] of house flies.

## Conclusions

We have sequenced and analyzed the genome of the *M. domestica* using DNA from female flies. This represents the first genome sequence of an insect that lives in intimate association with abundant animal pathogens. The sequenced genome size is 691 Mb and contains 15,345 genes. Compared with *D. melanogaster*, the genome contains a rich resource of shared and novel protein coding genes and a significantly higher amount of repetitive elements.

In comparison to *D. melanogaster*, the house fly genome has a larger number of genes associated with immune response, detoxification and chemosensation. Relative to *D. melanogaster* there are substantial increases in copy number and diversity of both the recognition and effector components of the immune system in the house fly genome, consistent with life in a pathogen-rich environment. For detoxification genes, there are 146 P450 genes (plus 11 pseudogenes) in *M. domestica*, representing a significant increase relative to *D. melanogaster* (or *G. morsitans*) and suggesting the presence of enhanced detoxification capacity in house flies. Relative to *D. melanogaster*, *M. domestica* has also evolved an expanded repertoire of chemoreceptors and odorant binding proteins, many associated with gustation.

The availability of the house fly genome should accelerate the pace of research on this important vector of human and animal diseases. The house fly genome provides a rich resource for enabling work on innovative methods of insect control, for understanding the mechanisms of insecticide resistance, genetic adaptation to high pathogen loads, host parasitoid interactions, and for exploring the basic biology of this important pest. The genome of this species will also serve as a close out-group to *G. morsitans* in comparative genomic studies.

## Materials and methods

### Genome and transcriptomes

DNA was extracted [103] from individual unmated adult females of the *M. domestica* aabys strain [104] and used for genome sequencing. The aabys strain was selected because it was inbred (to reduce polymorphisms and thus facilitate the genome assembly), is an XY strain, and because it is a widely disseminated and commonly used strain, particularly for linkage analyses. A total sequence coverage of approximately 90× was generated from fragment and jumping libraries then assembled using the ALLPATHS-LG assembler [105]. Contaminating contigs, adaptors, ambiguous bases as N's in the sequence and all contigs 200 bp and smaller were removed. The final *M. domestica* 2.0.2 genome sequence is available under the GenBank accession number AQPM00000000.1 and NCBI assembly accession GCA_000371365.1. All genome sequences utilized in *de novo* assembly of *M. domestica* were submitted to the NCBI short read archive (SRA) under accession numbers SRX217932-217940.

RNA was isolated from individual last instar larvae (n = 1; accession SRX208995), individual 1-day-old adult males (n = 2; accessions SRX208993 and SRX208994) and individual unmated 1-day-old adult females (n = 2; accessions SRX208996 and SRX208997) as described previously [106]. Poly(A) + RNA was isolated as a starting input for the Ovation® RNASeq System V2 (NuGEN, San Carlos, CA USA). A check of quality was measured with the Agilent Bioanalyzer. From samples that passed our quality control (minimum RNA integrity number (RIN) score of 7), a non-normalized cDNA library was constructed using a modified version of the Ovation® RNASeq System V2 [107] that generates strand specificity, an important factor in alignment biases seen with non-directional RNA-seq data. We sequenced each cDNA library (0.125 lane) on an Illumina HiSeq 2000 instrument (approximately 36 Gb per lane) at 100 bp length. These data provided sufficient sequence coverage of the estimated exon content (approximately 29 Mb) of a 691 Mb assembled house fly genome.

### Gene annotation

The pipeline used for the annotation of house fly is fully documented in the NCBI handbook [108] and is briefly described here. Prior to gene annotation 52% of the assembly was masked with WindowMasker [109], a word-based algorithm that identifies repeats *de novo*. By comparison, masking with the RepeatMasker library would have resulted in only 2.15% of the genome being masked. The annotation process was initiated by the alignment to the masked genome of publicly available house fly transcripts and RNA-seq from project SRP015949 with Splign [110], and Diptera proteins by ProSplign. Overlapping alignments with compatible frames were assembled into chains and extended or filled-in as needed by the *ab initio* prediction component of Gnomon to form complete models [137]. The resulting models were then evaluated and retained or discarded based on multiple criteria, including evidence support and homology to existing proteins. Following manual checks we predicted a total house fly gene count of 15,349, consisting of 14,180 protein-coding genes (with 17,508 transcripts), 1,165 non-coding genes and 4 pseudogenes. The number of genes is comparable to *D. melanogaster* (15,771) [111]. A total of 3,985 transcripts were filled-in or extended by *ab initio* prediction for 5% of their length or more, and 1,375 models were marked partial. The mean number of exons per transcript were estimated to be 4.9.

### Gene ontology

In total, 17,508 protein sequences were searched against Swiss-Prot with the BLASTp algorithm [40]. The E-value cutoff was set at 10^−5^ and taking the best 20 hits for annotation. Blast2GO [112,113] was used to predict the functions of the sequences and assign GO terms. Simplification of the annotation into functional categories was also done by Blast2GO using GO slim. Proteins were summarized at level 2 into three main GO categories (biological process, cellular component, and molecular function) and 33 subcategories.

### Defining homology to *D. melanogaster*

To define homology between *D. melanogaster* and *M. domestica* proteins, we started with an all-against-all BLASTp, using standard parameters and an E-value cutoff of 1e-5. We then filtered hits to remove all hits with similarity below 30% and at least 70% alignment coverage (dropping to 40% if the aligned region is at least 100 amino acids long). After filtering, we converted E-values to scores by taking the negative log10 (capped at 200), and then for each query computed a minimum score to keep by subtracting 10 from the minimum of the maximum score to the other species or the average of the top 5 hits. After removing hits below the minimum score for each query, we clustered proteins into groups using MCL [114] with the following parameters: −we 2 --force-connected = y -scheme 7. These are considered homologous groups. This procedure is tuned to be conservative about missing true orthologs at the cost of inflating group size by linking sets of reciprocal best hits into a single group.

To resolve relationships among groups that contain more than one *D. melanogaster* or *M. domestica* member, we aligned members of each cluster using mafft (with the --auto option) [115], trimmed the resulting alignments with trimal (default options) [116], and then computed trees with phyml [117] using default options. After computing trees for each paralogous group, we used the SDI algorithm implemented in TreeBest [138] and RIO [118] to resolve speciation and duplication events. We split groups that could be completely parsed into smaller orthologous groups, but retained as large families cases with complicated histories, which implied lineage-specific losses at the root of the tree. In many cases these likely resulted from low-confidence basal nodes. For all single copy orthologous gene pairs between *M. domestica* and *D. melanogaster*, we produced protein alignments using mafft [115] with the --auto flag and otherwise default parameters. Rates of protein divergence were calculated for each alignment with PAML version 4.4d [119].

### Immune-related genes

Two complementary computational approaches were used to define the repertoire of immune-related genes in *M. domestica*. The first relied on the well-annotated *D. melanogaster* genome. We curated a list of immune-related *D. melanogaster* proteins from the literature (updated from [47]), and assumed an immune function for proteins in *M. domestica* that are homologous (as defined by the method described above) to proteins with immune function in *D. melanogaster*. To supplement the homology-based annotations, our second approach applied a HMM originally developed to characterize the mosquito immune system [120]. Using curated alignments of putative immune-related proteins and domains from *D. melanogaster* and two mosquitoes available at ImmunoDB, plus a NIM domain alignment [46], we built HMMs using HMMER [139], and then computed the likelihood of containing each domain for each *M. domestica* predicted protein. After correcting for the number of domains tested, we retained all calls with an E-value <0.01, assigning genes to the class with the lowest E-value in cases where a single protein hit multiple domains. The classes included are: several antimicrobial peptides (attacins, cecropins, defensins, diptericins), CLIP-domain serine proteases (−A, −B, −C-, −D, and -E), serpins, C-type lectins (CTLs) and galectins, beta-glucan binding proteins, peptidoglycan recognition proteins, fibrinogen-related proteins (FREPs), peroxidases, lysozymes, MD2-like receptors, Nimrods, prophenoloxidases, scavenger receptors (A, B, and C), thioester-containing proteins, Tolls, spaetzle-like proteins, and Rel-domain proteins. In some cases (for example, SrcA, galectin, FREPs, CLIP-A, CTLs, peroxidases) there is little evidence for an immune role in *D. melanogaster*, but we included them in our analysis given the evidence for an immune role in other metazoans. However, it is important to note that similarity to an HMM cannot guarantee an immune function, as many immune-related proteins in insects share domains with non-immune functions (that is, serine proteases).

### Metabolism/detoxification genes

Primary metabolism of xenobiotics is most commonly carried out by cytochrome P450s, esterases/hydrolases and/or GSTs. To identify these genes two approaches were taken. TBLASTn searches [121] were carried out using all the known sequences of insect P450s, GSTs and esterases. We also searched the annotated genome for appropriately named sequences.

### Cys-loop ligand-gated ion channels and actin

Putative *M. domestica* cys-loop ligand-gated ion channel subunits were identified by searching the annotated genome with TBLASTn [121] using protein sequences of every member of the *D. melanogaster* cys-loop ligand-gated ion channel superfamily. The neighbor-joining method [122], available with the Clustal X program [123], was used to construct a phylogenetic tree, which was then viewed using TreeView [140]. Actin sequences were identified using the same approach.

### Chemoreceptors

The GR family was manually annotated using methods employed for other insect genomes [87]. Briefly, TBLASTn searches were performed using all *D. melanogaster* GRs as queries, and gene models were manually assembled in TextWrangler [141]. Additional details are provided in Additional file 16.

### Sex determination, sex-biased gene expression and the evolution of sex-biased genes

*Md-tra*^*D*^ females were collected from seven populations in different countries and different continents: Trabzon (Turkey), Faverges (France), Santa Fé (Spain), Tansania, North Carolina (USA), Osaka (Japan) and Ipswich (Australia).

RNA-seq reads of two biological replicates each of adult males and females were aligned to the reference genome using TopHat2 (v2.0.8b) [124] with the default parameters. We tested for differential expression between the male and female samples using Cuffdiff version 2.1.1 [125] with the default parameters and a false discovery rate of 0.05.

### microRNA and microRNA machinery

The mature miRNAs from the miRbase database (release 20) [126] were tested against the *M. domestica* supercontigs using miRdeep2, version 2.0.0.5 [127,128]. The known *D. melanogaster* miRNAs were used as the reference mature miRNA sets [129-131]. The miRNA machinery was primarily predicted from the Gnomon annotated *M. domestica* genome. Additional gene prediction for genes putatively involved in double-stranded RNA uptake was predicted by BLASTp comparison [121] to known genes in the *D. melanogaster genome* (v. dmel_r5.9_FB2008_06 [60]).

## Additional files

Additional file 1: Table S1. Gene families with the largest increases in copy number in the *M. domestica* genome, relative to *D. melanogaster*. Additional file 2: Table S2. Transcript coverage varies among insect genomes as a result of assembly contiguity, dictated by repeat composition. Additional file 3: Table S3. Comparison of the *M. domestica* genome to a previously published transcriptome of *M. domestica.*
Additional file 4: Figure S1. Gene Ontology analysis of the *M. domestica* genome. Additional file 5: Table S4. Comparative gene ontology between *M. domestica* (*Md*) and *D. melanogaster* (*Dm*). Additional file 6: Table S5. Cytochrome P450 genes in the *M. domestica* genome with reference to the cytochrome P450 genes present in the *D. melanogaster* genome. Additional file 7: Table S6. Predicted cytochrome P450 genes in the *Glossina* genome. Additional file 8: Table S7. Predicted cytochrome P450 genes in the *M. domestica* genome. Additional file 9: Table S8. Predicted GSTs and esterases in the *M. domestica* genome. Additional file 10: Figure S2. Unrooted neighbor-joining tree showing the phylogenetic analysis of GSTs of *M. domestica* (MD, red) in relation to GSTs from *D. melanogaster* (DM, green). MUSCLE software was used to perform multiple sequence alignment [132]. The neighbor-joining method was applied to the multiple sequence alignment using MEGA 5.0 [133]. Distance bootstrap values of >70% (1,000 replicates) are indicated at the corresponding nodes. The GST classes are colored distinctively: microsomal, turquoise; sigma, dark blue; omega, orange; zeta, dark red; theta, pink; delta, light blue and epsilon, light green. Sequences and the names for the *D. melanogaster* GST genes were taken from FlyBase [60]. Additional file 11: Table S9. CysLGICs in the house fly, *M. domestica*. Additional file 12: Table S10. Details of MdOBP family genes and proteins. Additional file 13: Table S11. Details of MdOR family genes and proteins. Additional file 14: Table S12." Details of MdGR family genes and proteins. Additional file 15: Table S13. Details of MdIR family genes and proteins. Additional file 16:
**Chemoreceptors, including protein sequences.**
Additional file 17: Figure S3. Phylogenetic tree of the *M. domestica* and *D. melanogaster* ORs. This is a corrected distance tree with the OrCo orthologs as the out-group to root the tree. The OrCo orthologs were declared as the out-group to root the tree, based on the basal position of this gene in the OR family in analysis of the entire chemoreceptor superfamily in *D. melanogaster* [87]. Comments on major gene lineages are on the right. Suffixes after the gene/protein names include: FIX, sequence fixed with raw reads; INT, internal sequence missing; JOI, gene model joined across scaffolds; multiple suffixes are abbreviated to single letters. The *M. domestica* and *D. melanogaster* gene/protein names are highlighted in blue and red, respectively, as are the branches leading to them to emphasize gene lineages. Bootstrap support level in percentage of 10,000 replications of uncorrected distance analysis is shown above major branches. Inferred ancestral and orthologous lineages are highlighted in double thickness. Suffixes after the gene/protein names are: NTE, amino terminus missing; CTE, carboxyl terminus missing; PSE, pseudogene. Additional file 18: Figure S4. Phylogenetic tree of the *M. domestica* and *D. melanogaster* OBPs. This is a corrected distance tree and was rooted at the midpoint in the absence of a simple obvious out-group. See Additional file 17 legend for other details. Additional file 19: Figure S5. Phylogenetic tree of the *M. domestica* and *D. melanogaster* IRs. This is a corrected distance tree rooted with IR8a/25a as the out-group, based on their highly conserved sequences and ancestral position in the family [134-136]. See Additional file 17 legend for other details. Additional file 20: Figure S6. Phylogenetic tree of the *M. domestica* and *D. melanogaster* GRs. This is a corrected distance tree rooted by declaring the distantly related and divergent carbon dioxide and sugar receptor subfamilies as the out-groups. The relationships within the sugar receptor subfamily are not accurate in this tree because many of these genes in *M. domestica* are only partially assembled. See Additional file 17 legend for other details. Additional file 21: Table S14. Test of sex-biased expression in *M. domestica* using cuffdiff [122]. Additional file 22: Table S15. Locations of putative microRNA and their predicted precursor hairpins from the *M. domestica* genome. Additional file 23: Table S16. MicroRNA machinery and genes predicted to be involved in exogenous double-stranded RNA uptake in *M. domestica*.

## Abbreviations

AMP: antimicrobial peptide
bp: base pair
FET: Fisher’s exact test
FREP: fibrinogen-related protein
GABA: γ-aminobutyric acid
GO: Gene Ontology
GR: gustatory receptor
GST: glutathione *S*-transferase
HMM: hidden Markov model
IR: ionotropic receptor
miRNA: microRNA
MW: Mann–Whitney
nAChR: nicotinic acetylcholine receptor
OBP: odorant binding protein
OR: odorant receptor
Tep: thioester-containing protein
