# Supplementary material for: Genome of the house fly, Musca domestica L., a global vector of diseases with adaptations to a septic environment
Source: Genome Biol. 2014 Oct 14;15:466. doi: 10.1186/s13059-014-0466-3 (PMC4195910; doi:10.1186/s13059-014-0466-3)
Supplement: Additional file 4: Figure S1. — Gene Ontology analysis of the M. domestica genome. [file 13059_2014_466_MOESM4_ESM.pdf]

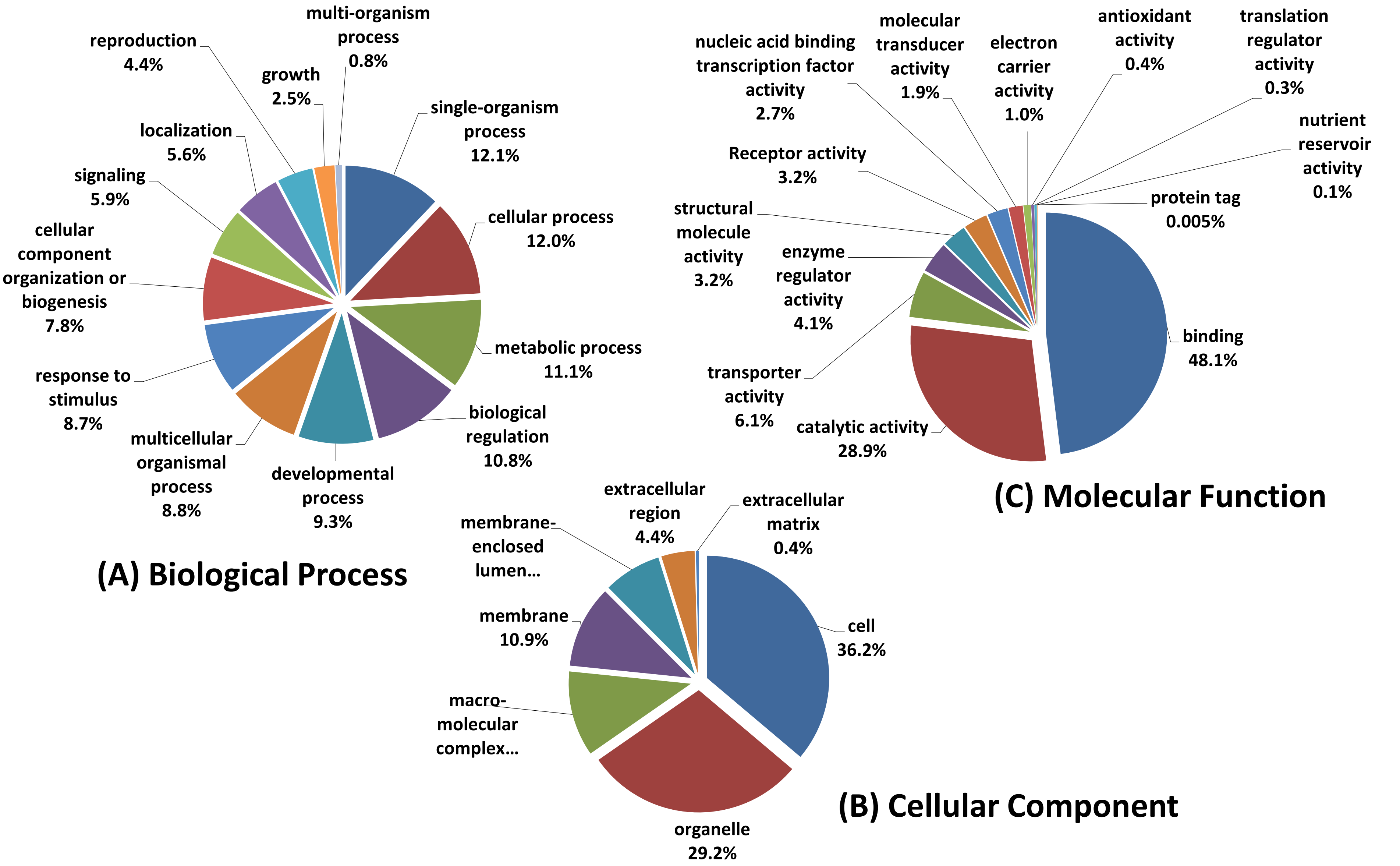

Supplementa Figure 1. Classification of *Musca domestica* genes based on Predicted Gene Ontology (GO) terms. (A) Biological Process, (B) Cellular Components, and (C) Molecular Function. GO terms were determined using Blast2GO with an e-value cutoff of $1e^{-5}$ and sorted based on level 2 classifications.
