# Supplementary material for: Genome of the house fly, Musca domestica L., a global vector of diseases with adaptations to a septic environment
Source: Genome Biol. 2014 Oct 14;15:466. doi: 10.1186/s13059-014-0466-3 (PMC4195910; doi:10.1186/s13059-014-0466-3)
Supplement: Additional file 5: Table S4. — Comparative gene ontology between M. domestica (Md) and D. melanogaster (Dm). [file 13059_2014_466_MOESM5_ESM.docx]

**Table S4 Comparative gene ontology between *M. domestica* (*Md*) and *D. melanogaster* (*Dm*)**

| **Biological processes** | | | | | **Cellular components** | | | | | **Molecular function** | | | | |
| --- | --- | --- | --- | --- | --- | --- | --- | --- | --- | --- | --- | --- | --- | --- |
| process | *Md* | | *Dm* | | Component | *Md* | | *Dm* | | Activity | *Md* | | *Dm* | |
|  | % | Rank | % | Rank |  | % | Rank | % | Rank |  | % | Rank | % | Rank |
| single-organism | 12.1 | 1 | 15.3 | 2 | cell | 36.2 | 1 | 43.6 | 1 | binding | 48.1 | 1 | 42.4 | 1 |
| cellular | 12.0 | 2 | 15.6 | 1 | organelle | 29.2 | 2 | 26.6 | 2 | catalytic | 28.9 | 2 | 32.9 | 2 |
| metabolic | 11.1 | 3 | 13.5 | 3 | macromolecular complex | 11.3 | 3 | 13.9 | 3 | transporter | 6.1 | 3 | 7.0 | 3 |
| biological regulation | 10.8 | 4 | 8.2 | 6 | membrane | 10.9 | 4 | 5.0 | 5 | enzyme regulator | 4.1 | 4 | 3.3 | 6 |
| developmental | 9.3 | 5 | 10.5 | 4 | membrane-enclosed lumen | 7.7 | 5 | 4.0 | 6 | structural molecule | 3.2 | 5 | 4.9 | 4 |
| multicellular organismal | 8.8 | 6 | 9.2 | 5 | extracellular region | 4.4 | 6 | 6.5 | 4 | receptor | 3.2 | 6 | 3.8 | 5 |
| response to stimulus | 8.7 | 7 | 7.0 | 8 | extracellular matrix | 0.4 | 7 | 0.5 | 7 | nucleic acid binding transcription factor | 2.7 | 7 | 3.3 | 7 |
| cellular component organization or biogenesis | 7.8 | 8 | 7.0 | 7 |  |  |  |  |  | molecular transducer | 1.9 | 8 | 1.0 | 9 |
| signaling | 5.9 | 9 | 4.6 | 9 |  |  |  |  |  | electron carrier | 1.0 | 9 | 1.0 | 8 |
| localization | 5.6 | 10 | 4.2 | 10 |  |  |  |  |  | antioxidant | 0.4 | 10 | 0.3 | 10 |
| reproduction | 4.4 | 11 | 3.9 | 11 |  |  |  |  |  | translation regulator | 0.3 | 11 | 0.1 | 11 |
| growth | 2.5 | 12 | 0.9 | 12 |  |  |  |  |  | nutrient reservoir | 0.1 | 12 |  |  |
| multi-organism | 0.8 | 13 | 0.0 | 13 |  |  |  |  |  | protein tag | 0.0 | 13 |  |  |
