# Supplementary material for: Genome of the house fly, Musca domestica L., a global vector of diseases with adaptations to a septic environment
Source: Genome Biol. 2014 Oct 14;15:466. doi: 10.1186/s13059-014-0466-3 (PMC4195910; doi:10.1186/s13059-014-0466-3)
Supplement: Additional file 6: Table S5. — Cytochrome P450 genes in the M. domestica genome with reference to the cytochrome P450 genes present in the D. melanogaster genome. [file 13059_2014_466_MOESM6_ESM.docx]

**Table S5 Cytochrome P450 genes in the *Musca domestica* genome with reference to the cytochrome P450 genes present in the *Drosophila melanogaster* genome**

| **Clan** | **Family^†^** | **Number of transcripts^*^** | | **Number of** **pseudogenes** | |
| --- | --- | --- | --- | --- | --- |
|  |  | *M. domestica* | *D. melanogaster* | *M. domestica* | *D. melanogaster* |
| 2 | CYP18 | 1 | 1 | - | - |
|  | CYP303 | 1 | 1 | - | - |
|  | CYP304 | 3 | 1 | - | - |
|  | CYP305 | 1 | 1 | - | - |
|  | CYP306 | 1 | 1 | - | - |
|  | CYP307 | 1 | 2 | - | - |
| 3 | CYP6 | 46 | 22 | 7 | 3 |
|  | CYP9 | 7 | 5 | - | 1 |
|  | CYP28 | 6 | 4 | 2 | - |
|  | CYP308 | - | 1 | - | - |
|  | CYP309 | - | 2 | - | - |
|  | CYP310 | 2 | 1 | - | - |
|  | CYP437 | 3 | - | - | - |
|  | CYP438 | 1 | - | - | - |
| 4 | CYP4 | 43 | 22 | 1 | - |
|  | CYP311 | 1 | 1 | - | - |
|  | CYP312 | - | 1 | - | - |
|  | CYP313 | 4 | 6 | 1 | - |
|  | CYP316 | - | 1 | - | - |
|  | CYP317 | 1 | 1 | - | - |
|  | CYP318 | 1 | 1 | - | - |
|  | CYP3073 | 5 | - | - | - |
| mito | CYP12 | 12 | 6 | - | - |
|  | CYP49 | 1 | 1 | - | - |
|  | CYP301 | 1 | 1 | - | - |
|  | CYP302 | 2 | 1 | - | - |
|  | CYP314 | 1 | 1 | - | - |
|  | CYP315 | 1 | 1 | - | - |
|  | TOTAL | 146 | 86 | 11 | 4 |

^†^Names provided by the cytochrome P450 nomenclature committee.

^*^Includes predicted splice variants within gene.
