# Supplementary material for: Genome of the house fly, Musca domestica L., a global vector of diseases with adaptations to a septic environment
Source: Genome Biol. 2014 Oct 14;15:466. doi: 10.1186/s13059-014-0466-3 (PMC4195910; doi:10.1186/s13059-014-0466-3)
Supplement: Additional file 7: Table S6. — Predicted cytochrome P450 genes in the Glossina genome. [file 13059_2014_466_MOESM7_ESM.docx]

**Table S7 Predicted cytochrome P450 genes in the *Musca domestica* genome**

| **Clan** | **CYP name^†^** | **XM_number^‡^** | **XP_number^‡^** | **Genome locus** | **Comments** |
| --- | --- | --- | --- | --- | --- |
| 2 | CYP18A1 | XM_005183375 | XP_005183432 | LOC101900728 |  |
|  | CYP303A1 | XM_005184116 | XP_005184173 | LOC101898085 |  |
|  | CYP304A1 | XM_005183058 | XP_005183115 | LOC101890089 |  |
|  | CYP304A2^*^ | XM_005183057 | XP_005183114 | LOC101889924 |  |
|  | CYP304A2^*^ | XM_005191871 | XP_005191928 | LOC101895933 | C-terminus end starting near the end of supercontig gi\|519130807\|ref\|NW_004773940\| at position 10281 of 10300 (- strand orientation) |
|  | CYP305A1 | XM_005180589 | XP_005180646 | LOC101898997 |  |
|  | CYP306A1 | XM_005183378 | XP_005183435 | LOC101901255 |  |
|  | CYP307A2 | XM_005175638 | XP_005175695 | LOC101900639 |  |
|  |  |  |  |  |  |
| 3 | CYP28 | XM_005191969 | XP_005192026 | LOC101900938 | fragment^§^ |
|  | CYP28 | XM_005176371 | XP_005176428 | LOC101900413 | possible fragment^§^ |
|  | CYP28B1 | XM_005180394 | XP_005180451 | LOC101897279 |  |
|  | CYP28B2 | XM_005180398 | XP_005180455 | LOC101897848 |  |
|  | CYP28G6 | XM_005184255 | XP_005184312 | LOC101890714 |  |
|  | CYP28H1 | XM_005184258 | XP_005184315 | LOC101891407 |  |
|  | CYP28J1 | XM_005184259 | XP_005184316 | LOC101891587 |  |
|  | CYP28K1 | XM_005180399 | XP_005180456 | LOC101898011 |  |
|  | CYP310B2^*^ | XM_005184125 | XP_005184182 | LOC101899919 |  |
|  | CYP310B2^*^ | XM_005191537 | XP_005191594 | LOC101890973 | C-terminus end starting near the end of supercontig gi\|519132605\|ref\|NW_004772142\| at position 1881 of 13069 (+ strand orientation) |
|  | CYP437A4v1 | XM_005187739 | XP_005187796 | LOC101890335 | variant for LOC101890335 |
|  | CYP437A4v2 | XM_005187740 | XP_005187797 | LOC101890335 | variant for LOC101890335 |
|  | CYP437A4v3 | XM_005187741 | XP_005187798 | LOC101890335 | variant for LOC101890335 |
|  | CYP438A4 | XM_005183376 | XP_005183433 | LOC101900906 |  |
|  | CYP6 | XM_005186700 | XP_005186757 | LOC101901429 | pseudogene^§^ |
|  | CYP6 | XM_005186701 | XP_005186758 | LOC101901592 | pseudogene^§^ |
|  | CYP6A | XM_005190470 | XP_005190527 | LOC101892586 | fragment 1^§^ |
|  | CYP6A | XM_005184342 | XP_005184399 | LOC101891588 | fragment 2^§^ |
|  | CYP6A1 | XM_005184331 | XP_005184388 | LOC101889365 |  |
|  | CYP6A24 | XM_005190469 | XP_005190526 | LOC101892246 |  |
|  | CYP6A25 | XM_005190472 | XP_005190529 | LOC101892931 |  |
|  | CYP6A36^*^ | XM_005184332 | XP_005184389 | LOC101889539 |  |
|  | CYP6A36^*^ | XM_005191950 | XP_005192007 | LOC101897022 | missing C-terminus, sequence runs to the end of supercontig gi\|519130513\|ref\|NW_004774234\| (- strand orientation) |
|  | CYP6A37 | XM_005184336 | XP_005184393 | LOC101890373 |  |
|  | CYP6A37 | XM_005184335 | XP_005184392 | LOC101890199 | partial^§^ |
|  | CYP6A4 | XM_005184338 | XP_005184395 | LOC101890889 |  |
|  | CYP6A40^*^ | XM_005191930 | XP_005191987 | LOC101893114 |  |
|  | CYP6A40^*^ | XM_005184343 | XP_005184400 | LOC101891761 |  |
|  | CYP6A5 | XM_005184348 | XP_005184405 | LOC101892622 |  |
|  | CYP6A52 | XM_005184334 | XP_005184391 | LOC101890041 |  |
|  | CYP6A54 | XM_005175565 | XP_005175622 | LOC101899899 |  |
|  | CYP6A56 | XM_005190826 | XP_005190883 | LOC101895803 | C-terminus end missing due to low read coverage for supercontig gi\|519136837\|ref\|NW_004769377\| at position 5673 |
|  | CYP6A57 | XM_005190468 | XP_005190525 | LOC101892072 |  |
|  | CYP6A58^††^ | XM_005184340 | XP_005184397 | LOC101891234 | missing EXXR containing exon, possible partial matching with XM_005184341 |
|  | CYP6A58^††^ | XM_005184341 | XP_005184398 | LOC101891408 | possible partial matching with XM_005184340 |
|  | CYP6A59 | XM_005184344 | XP_005184401 | LOC101891933 |  |
|  | CYP6A6^††^ | XM_005184347 | XP_005184404 | LOC101892447 | partial C-term EXXR, possible matching with XM_005184345 and XM_005184346 |
|  | CYP6A6^††^ | XM_005184345 | XP_005184402 | LOC101892108 | N-terminus partial, possible matching with XM_005184346 and XM_005184347 |
|  | CYP6A6^††^ | XM_005184346 | XP_005184403 | LOC101892278 | internal fragment, possible partial matching with XM_005184345 and XM_005184347 |
|  | CYP6A63P | XM_005175566 | XP_005175623 | LOC101900065 | pseudogene^§^ |
|  | CYP6A7 | XM_005184333 | XP_005184390 | LOC101889704 |  |
|  | CYP6C1 | XM_005184349 | XP_005184406 | LOC101892797 |  |
|  | CYP6C2 | XM_005184350 | XP_005184407 | LOC101892970 |  |
|  | CYP6D | XM_005180560 | XP_005180617 | LOC101894260 | fragment 1^§^ |
|  | CYP6D10 | XM_005184128 | XP_005184185 | LOC101900431 |  |
|  | CYP6D11 | XM_005183145 | XP_005183202 | LOC101889532 |  |
|  | CYP6D12 | XM_005185632 | XP_005185689 | LOC101899135 |  |
|  | CYP6D1^††^ | XM_005184124 | XP_005184181 | LOC101899746 | C-terminus running from 235004 – 235583 on supercontig gi\|5191410541\|ref\|NW_004765160\| (+ strand orientation), possible partial matching with XM_005184130 |
|  | CYP6D1^††^ | XM_005184130 | XP_005184187 | LOC101900791 | N-terminus running from 239705-23367 on supercontig gi\|519141054\|ref\|NW_004765160\| (+ strand orientation), possible partial matching with XM_005184124 |
|  | CYP6D3 | XM_005184123 | XP_005184180 | LOC101899585 |  |
|  | CYP6D8 | XM_005185673 | XP_005185730 | LOC101891297 |  |
|  | CYP6EK2 | XM_005185208 | XP_005185265 | LOC101898668 |  |
|  | CYP6FS2v1 | XM_005182161 | XP_005182218 | LOC101887226 | variant for LOC101887226 |
|  | CYP6FS2v2 | XM_005182160 | XP_005182217 | LOC101887226 | variant for LOC101887226 |
|  | CYP6FT2 | XM_005190444 | XP_005190501 | LOC101887865 |  |
|  | CYP6FT3 | XM_005190452 | XP_005190509 | LOC101889269 |  |
|  | CYP6FT4 | XM_005190451 | XP_005190508 | LOC101889092 |  |
|  | CYP6FT5 | XM_005190450 | XP_005190507 | LOC101888912 |  |
|  | CYP6FT6 | XM_005190449 | XP_005190506 | LOC101888732 |  |
|  | CYP6FT7 | XM_005190425 | XP_005190482 | LOC101899768 |  |
|  | CYP6G4 | XM_005188667 | XP_005188724 | LOC101898562 |  |
|  | CYP6G7 | XM_005187884 | XP_005187941 | LOC101900444 |  |
|  | CYP6GU1 | XM_005184337 | XP_005184394 | LOC101890543 |  |
|  | CYP6GV1 | XM_005189530 | XP_005189587 | LOC101894510 | C-terminus missing due to low read coverage for supercontig gi\|519139462\|ref\|NW_004766752\| at position 19089 |
|  | CYP6GV2 | XM_005189529 | XP_005189586 | LOC101894349 |  |
|  | CYP6GW1 | XM_005190471 | XP_005190528 | LOC101892757 |  |
|  | CYP6GY1 | XM_005188672 | XP_005188729 | LOC101899434 |  |
|  | CYP6GZ1 | XM_005175852 | XP_005175909 | LOC101887482 |  |
|  | CYP6HA1 | XM_005177719 | XP_005177776 | LOC101887383 |  |
|  | CYP6HB1 | XM_005186997 | XP_005187054 | LOC101893767 |  |
|  | CYP6V3 | XM_005176346 | XP_005176403 | LOC101896204 |  |
|  | CYP9F10 | XM_005180062 | XP_005180119 | LOC101900482 |  |
|  | CYP9F11 | XM_005180054 | XP_005180111 | LOC101899118 |  |
|  | CYP9F12 | XM_005180063 | XP_005180120 | LOC101900658 |  |
|  | CYP9F7 | XM_005180052 | XP_005180109 | LOC101898775 |  |
|  | CYP9F8v1 | XM_005180050 | XP_005180107 | LOC101898478 | variant for LOC101898478 |
|  | CYP9F8v2 | XM_005180051 | XP_005180108 | LOC101898478 | variant for LOC101898478 |
|  | CYP9F9 | XM_005180053 | XP_005180110 | LOC101898942 |  |
|  |  |  |  |  |  |
| 4 | CYP3073A1 | XM_005187010 | XP_005187067 | LOC101896297 |  |
|  | CYP3073A2 | XM_005187012 | XP_005187069 | LOC101896650 |  |
|  | CYP3073A3 | XM_005187013 | XP_005187070 | LOC101896830 |  |
|  | CYP3073B1 | XM_005187011 | XP_005187068 | LOC101896469 |  |
|  | CYP3073B2 | XM_005187014 | XP_005187071 | LOC101897002 |  |
|  | CYP311A1 | XM_005180423 | XP_005180480 | LOC101887394 |  |
|  | CYP313D1^*^ | XM_005189825 | XP_005189882 | LOC101890728 |  |
|  | CYP313D1^*^ | XM_005175041 | XP_005175098 | LOC101887770 | N-terminus end starting near the end of supercontig gi\|519150122\|ref\|NW_004756092\| at position 2491 of 3149 (+ strand orientation) |
|  | CYP313D2 | XM_005188279 | XP_005188336 | LOC101893162 |  |
|  | CYP313D3 | XM_005189830 | XP_005189887 | LOC101891548 |  |
|  | CYP313D4 | XM_005182545 | XP_005182602 | LOC101894207 | N-terminus no stop codon^§^ |
|  | CYP317A3 | XM_005184339 | XP_005184396 | LOC101891061 |  |
|  | CYP318B1 | XM_005189277 | XP_005189334 | LOC101892636 |  |
|  | CYP4AA1 | XM_005175977 | XP_005176034 | LOC101895233 |  |
|  | CYP4AC6 | XM_005186465 | XP_005186522 | LOC101901643 |  |
|  | CYP4AD1 | XM_005186278 | XP_005186335 | LOC101897760 |  |
|  | CYP4AE3v1 | XM_005177255 | XP_005177312 | LOC101898177 | variant for LOC101898177 |
|  | CYP4AE3v2 | XM_005177253 | XP_005177310 | LOC101898177 | variant for LOC101898177 |
|  | CYP4AE3v3 | XM_005177254 | XP_005177311 | LOC101898177 | variant for LOC101898177 |
|  | CYP4C74 | XM_005185973 | XP_005186030 | LOC101889014 |  |
|  | CYP4D3v1 | XM_005177259 | XP_005177316 | LOC101898930 | variant for LOC101898930 |
|  | CYP4D3v2 | XM_005177258 | XP_005177315 | LOC101898930 | variant for LOC101898930 |
|  | CYP4D36 | XM_005180553 | XP_005180610 | LOC101892899 |  |
|  | CYP4D4 | XM_005183986 | XP_005184043 | LOC101891759 |  |
|  | CYP4D54 | XM_005177250 | XP_005177307 | LOC101897669 |  |
|  | CYP4D55 | XM_005177252 | XP_005177309 | LOC101898004 |  |
|  | CYP4D56 | XM_005177251 | XP_005177308 | LOC101897841 |  |
|  | CYP4D58v1 | XM_005183988 | XP_005184045 | LOC101891931 | variant for LOC101891931 |
|  | CYP4D58v2 | XM_005183987 | XP_005184044 | LOC101891931 | variant for LOC101891931 |
|  | CYP4D58v3 | XM_005183989 | XP_005184046 | LOC101891931 | variant for LOC101891931 |
|  | CYP4D61 | XM_005183993 | XP_005184050 | LOC101892853 |  |
|  | CYP4D62 | XM_005177344 | XP_005177401 | LOC101897033 |  |
|  | CYP4D63 | XM_005180561 | XP_005180618 | LOC101894425 |  |
|  | CYP4D64^††^ | XM_005190767 | XP_005190824 | LOC101899206 | N-terminus ending at position 835232 of 837024 of supercontig gi\|519136945\|ref\|NW_0047692691\| (+ strand orientation), possible partial matching with XM_005191906 or XM_005189730 |
|  | CYP4D64^††^ | XM_005189730 | XP_005189787 | LOC101888549 | identical to XM_005191906, C-terminus sequence starting near the edge of supercontig gi\|519139065\|ref\|NW_004767149\| at position 299 of 23537 (+ strand orientation), possible partial matching with XM_005190767 |
|  | CYP4D64^††^ | XM_005191906 | XP_005191963 | LOC101888620 | identical to XM_005189730, C-terminus sequence ending near the edge of supercontig gi\|519139065\|ref\|NW_004767149\| at position 7715 of 8551 (- strand orientation), possible partial matching with XM_005190767 |
|  | CYP4D65 | XM_005189731 | XP_005189788 | LOC101888728 |  |
|  | CYP4D66 | XM_005189732 | XP_005189789 | LOC101888909 |  |
|  | CYP4D67 | XM_005180562 | XP_005180619 | LOC101894586 |  |
|  | CYP4D68^*^ | XM_005190901 | XP_005190958 | LOC101896035 |  |
|  | CYP4D68^*^ | XM_005190900 | XP_005190957 | LOC101895863 | N-terminus partial, possible alternative transcription start site for XM_005190901 |
|  | CYP4D9 | XM_005177345 | XP_005177402 | LOC101897209 |  |
|  | CYP4E10v1 | XM_005186272 | XP_005186329 | LOC101896588 | variant for LOC101896588 |
|  | CYP4E10v2 | XM_005186271 | XP_005186328 | LOC101896588 | variant for LOC101896588 |
|  | CYP4E11 | XM_005186268 | XP_005186325 | LOC101896081 |  |
|  | CYP4E12 | XM_005186277 | XP_005186334 | LOC101897587 | missing a fragment^§^ |
|  | CYP4E7 | XM_005186267 | XP_005186324 | LOC101895915 |  |
|  | CYP4G103 | XM_005176293 | XP_005176350 | LOC101887719 |  |
|  | CYP4G13 | XM_005176292 | XP_005176349 | LOC101887550 |  |
|  | CYP4G2 | XM_005176294 | XP_005176351 | LOC101887882 |  |
|  | CYP4G96 | XM_005176299 | XP_005176356 | LOC101888747 |  |
|  | CYP4G97 | XM_005176300 | XP_005176357 | LOC101888923 |  |
|  | CYP4G98 | XM_005176301 | XP_005176358 | LOC101889105 |  |
|  | CYP4G99 | XM_005177736 | XP_005177793 | LOC101890640 |  |
|  | CYP4P10 | XM_005180896 | XP_005180953 | LOC101891224 |  |
|  | CYP4P11 | XM_005180895 | XP_005180952 | LOC101891052 |  |
|  | CYP4P8 | XM_005180909 | XP_005180966 | LOC101893472 |  |
|  | CYP4S23 | XM_005177495 | XP_005177552 | LOC101892717 |  |
|  | CYP4S24 | XM_005177488 | XP_005177545 | LOC101891157 |  |
|  |  |  |  |  |  |
| mito | CYP12A1^*^ | XM_005180004 | XP_005180061 | LOC101890758 |  |
|  | CYP12A1^*^ | XM_005190677 | XP_005190734 | LOC101898453 |  |
|  | CYP12A12 | XM_005180006 | XP_005180063 | LOC101891103 |  |
|  | CYP12A13 | XM_005180007 | XP_005180064 | LOC101891274 |  |
|  | CYP12A14 | XM_005179996 | XP_005180053 | LOC101889524 |  |
|  | CYP12A16^*^ | XM_005180005 | XP_005180062 | LOC101890931 |  |
|  | CYP12A16^*^ | XM_005190663 | XP_005190720 | LOC101896195 |  |
|  | CYP12A17 | XM_005177016 | XP_005177073 | LOC101889672 |  |
|  | CYP12A2 | XM_005179998 | XP_005180055 | LOC101889857 |  |
|  | CYP12A3 | XM_005179997 | XP_005180054 | LOC101889684 |  |
|  | CYP12G2 | XM_005180644 | XP_005180701 | LOC101893522 |  |
|  | CYP12G4 | XM_005183241 | XP_005183298 | LOC101892495 |  |
|  | CYP301A1 | XM_005177409 | XP_005177466 | LOC101893000 |  |
|  | CYP302A1v1 | XM_005179206 | XP_005179263 | LOC101888518 | variant for LOC101888518 |
|  | CYP302A1v2 | XM_005179205 | XP_005179262 | LOC101888518 | variant for LOC101888518 |
|  | CYP314A1 | XM_005178726 | XP_005178783 | LOC101896979 |  |
|  | CYP315A1 | XM_005184970 | XP_005185027 | LOC101901025 |  |
|  | CYP49A1 | XM_005174794 | XP_005174851 | LOC101887655 |  |

^†^Nomenclature provided by the cytochrome P450 nomenclature committee, David R. Nelson

^‡^RefSeq accession number, National Center for Biotechnology Information, Bethesda, MD

^*^Denotes occurrences of cytochrome P450 genes that sharing the same name but different genomic locations

^§^Probable pseudogene

^††^Denotes cytochrome P450 genes that are predicted from among more than one transcript, but are likely to belong to the same transcript.

The predicted transcripts for *CYP6A6*, *CYP6A58*, *CYP6D1*, and *CYP4D64* contained separate parts of the cytochrome P450 genes. For example, genes XM_005184124 and XM_005184130 contained the C-terminus and the N-terminus of *CYP6D1*, respectively, and were predicted to be transcribed in the same orientation (+ strand) on the same supercontig, suggesting that while the *ab initio* gene prediction predicted two partial cytochrome P450 genes, they may, in fact, represent a single complete gene.

One cytochrome P450 gene, *CYP4D68*, was predicted to have an alternative N-terminus (XM_005190900) upstream of the main cytochrome P450 predicted gene locus (XM_005190901), and four other P450s (CYP4D3, CYP4D4, CYP4D58, CYP4AC6 may have alternative splicing isoforms as well. The remaining four cytochrome P450 genes, *CYP6A6*, *CYP6A58*, *CYP6D1*, and *CYP4D64*, were represented by more than one predicted transcript that either spanned the edges of different supercontigs or were positioned proximal to each other within the genome.
