# Supplementary material for: Genome of the house fly, Musca domestica L., a global vector of diseases with adaptations to a septic environment
Source: Genome Biol. 2014 Oct 14;15:466. doi: 10.1186/s13059-014-0466-3 (PMC4195910; doi:10.1186/s13059-014-0466-3)
Supplement: Additional file 9: Table S8. — Predicted GSTs and esterases in the M. domestica genome. [file 13059_2014_466_MOESM9_ESM.doc]

**Table S8 Predicted glutathione *S*-transferases and esterases in the *Musca domestica*** genome

| **Predicted function** | **Genome locus** | **XM_number** | **XP_number** |
| --- | --- | --- | --- |
| Glutathione S-transferase | LOC101890402 | XM_005175528 | XP_005175585 |
|  | LOC101900949 | XM_005177543 | XP_005177600 |
|  | LOC101888110 | XM_005177555 | XP_005177612 |
|  | LOC101897212 | XM_005177693 | XP_005177750 |
|  | LOC101891696 | XM_005179225 | XP_005179282 |
|  | LOC101899848 | XM_005179451 | XP_005179508 |
|  | LOC101900016 | XM_005179452 | XP_005179509 |
|  | LOC101897094 | XM_005180042 | XP_005180099 |
|  | LOC101897277 | XM_005180043 | XP_005180100 |
|  | LOC101897452 | XM_005180044 | XP_005180101 |
|  | LOC101897621 | XM_005180045 | XP_005180102 |
|  | LOC101897797 | XM_005180046 | XP_005180103 |
|  | LOC101899791 | XM_005180058 | XP_005180115 |
|  | LOC101888181 | XM_005180696 | XP_005180753 |
|  | LOC101888349 | XM_005180697 | XP_005180754 |
|  | LOC101896120 | XM_005182646 | XP_005182703 |
|  | LOC101900726 | XM_005182675 | XP_005182732 |
|  | LOC101895956 | XM_005183587 | XP_005183644 |
|  | LOC101895956 | XM_005183588 | XP_005183645 |
|  | LOC101895956 | XM_005183589 | XP_005183646 |
|  | LOC101900672 | XM_005183706 | XP_005183763 |
|  | LOC101895316 | XM_005184605 | XP_005184662 |
|  | LOC101895316 | XM_005184606 | XP_005184663 |
|  | LOC101895607 | XM_005184607 | XP_005184664 |
|  | LOC101894873 | XM_005185108 | XP_005185165 |
|  | LOC101895036 | XM_005185109 | XP_005185166 |
|  | LOC101895555 | XM_005185112 | XP_005185169 |
|  | LOC101897519 | XM_005185937 | XP_005185994 |
|  | LOC101897863 | XM_005185939 | XP_005185996 |
|  | LOC101895262 | XM_005186599 | XP_005186656 |
|  | LOC101901044 | XM_005188597 | XP_005188654 |
|  | LOC101887250 | XM_005189970 | XP_005190027 |
|  | LOC101887423 | XM_005189971 | XP_005190028 |
|  | LOC101898455 | XM_005190996 | XP_005191053 |
|  | LOC101890455 | XM_005191032 | XP_005191089 |
|  | LOC101897781 | XM_005191498 | XP_005191555 |
| Acetylcholinesterase | LOC101890429 | XM_005183228 | XP_005183285 |
|  | LOC101895319 | XM_005185021 | XP_005185078 |
| Acyl-protein thioesterase | LOC101890399 | XM_005192151 | XP_005192208 |
|  | LOC101895446 | XM_005189687 | XP_005189744 |
|  | LOC101895446 | XM_005189688 | XP_005189745 |
| Phosphodiesterase | LOC101888887 | XM_005183542 | XP_005183599 |
| Carboxylesterase 1E | LOC101900211 | XM_005185388 | XP_005185445 |
| Esterase B1 | LOC101897154 | XM_005178638 | XP_005178695 |
|  | LOC101897501 | XM_005178640 | XP_005178697 |
|  | LOC101898347 | XM_005175103 | XP_005175160 |
|  | LOC101898526 | XM_005175104 | XP_005175161 |
|  | LOC101898698 | XM_005175105 | XP_005175162 |
|  | LOC101895121 | XM_005176152 | XP_005176209 |
|  | LOC101895815 | XM_005176156 | XP_005176213 |
|  | LOC101898009 | XM_005178642 | XP_005178699 |
|  | LOC101889275 | XM_005174719 | XP_005174776 |
|  | LOC101896445 | XM_005178634 | XP_005178691 |
|  | LOC101896625 | XM_005178635 | XP_005178692 |
|  | LOC101896807 | XM_005178636 | XP_005178693 |
|  | LOC101896978 | XM_005178637 | XP_005178694 |
|  | LOC101897334 | XM_005178639 | XP_005178696 |
|  | LOC101898181 | XM_005178643 | XP_005178700 |
|  | LOC101898354 | XM_005178644 | XP_005178701 |
|  | LOC101900490 | XM_005182230 | XP_005182287 |
| Esterase FE4 | LOC101890018 | XM_005177391 | XP_005177448 |
| Esterase P | LOC101889188 | XM_005183882 | XP_005183939 |
| Juvenile hormone esterase | LOC101887391 | XM_005179982 | XP_005180039 |
| Neuropathy target esterase | LOC101897743 | XM_005181277 | XP_005181334 |
|  | LOC101897743 | XM_005181278 | XP_005181335 |
|  | LOC101897743 | XM_005181279 | XP_005181336 |
| Methylesterase | LOC101894178 | XM_005189138 | XP_005189195 |
| Venom carboxylesterase | LOC101898361 | XM_005181454 | XP_005181511 |
|  | LOC101893731 | XM_005175393 | XP_005175450 |
|  | LOC101890179 | XM_005177392 | XP_005177449 |
|  | LOC101887332 | XM_005180691 | XP_005180748 |
|  | LOC101887499 | XM_005180692 | XP_005180749 |
|  | LOC101887671 | XM_005180693 | XP_005180750 |
|  | LOC101888003 | XM_005180695 | XP_005180752 |
|  | LOC101887955 | XM_005180961 | XP_005181018 |
| Acyl-coenzyme A thioesterase | LOC101897518 | XM_005185709 | XP_005185766 |
|  | LOC101888219 | XM_005190788 | XP_005190845 |
|  | LOC101888386 | XM_005190789 | XP_005190846 |
|  | LOC101892966 | XM_005183480 | XP_005183537 |
| Lysosomal thioesterase | LOC101892750 | XM_005189448 | XP_005189505 |
| Palmitoyl-protein thioesterase | LOC101899138 | XM_005186941 | XP_005186998 |
|  | LOC101900161 | XM_005186947 | XP_005187004 |
| Phosphodiesterase | LOC101892347 | XM_005187247 | XP_005187304 |
| Ubiquitin thioesterase | LOC101888928 | XM_005177980 | XP_005178037 |
|  | LOC101901059 | XM_005175571 | XP_005175628 |
| Metallophosphoesterase | LOC101901123 | XM_005175846 | XP_005175903 |
|  | LOC101892935 | XM_005191693 | XP_005191750 |
|  | LOC101893988 | XM_005183831 | XP_005183888 |
| Phosphodiesterase | LOC101900853 | XM_005184969 | XP_005185026 |
|  | LOC101897683 | XM_005183772 | XP_005183829 |
|  | LOC101900732 | XM_005183790 | XP_005183847 |
|  | LOC101900910 | XM_005183791 | XP_005183848 |
|  | LOC101896046 | XM_005177514 | XP_005177571 |
|  | LOC101896046 | XM_005177515 | XP_005177572 |
|  | LOC101900328 | XM_005184476 | XP_005184533 |
|  | LOC101890926 | XM_005177302 | XP_005177359 |
|  | LOC101890926 | XM_005177303 | XP_005177360 |
|  | LOC101890926 | XM_005177304 | XP_005177361 |
|  | LOC101890926 | XM_005177305 | XP_005177362 |
|  | LOC101890926 | XM_005177306 | XP_005177363 |
|  | LOC101888377 | XM_005187899 | XP_005187956 |
|  | LOC101888377 | XM_005187900 | XP_005187957 |
|  | LOC101898725 | XM_005185716 | XP_005185773 |
|  | LOC101901297 | XM_005178312 | XP_005178369 |
|  | LOC101897380 | XM_005192186 | XP_005192243 |
|  | LOC101888529 | XM_005183540 | XP_005183597 |
|  | LOC101894508 | XM_005188471 | XP_005188528 |
|  | LOC101894508 | XM_005188472 | XP_005188529 |
|  | LOC101895958 | XM_005185191 | XP_005185248 |
|  | LOC101895958 | XM_005185192 | XP_005185249 |
|  | LOC101895958 | XM_005185193 | XP_005185250 |
|  | LOC101897609 | XM_005177167 | XP_005177224 |
|  | LOC101891622 | XM_005177219 | XP_005177276 |
|  | LOC101888991 | XM_005179475 | XP_005179532 |
|  | LOC101897749 | XM_005183852 | XP_005183909 |
|  | LOC101888955 | XM_005186805 | XP_005186862 |
|  | LOC101897601 | XM_005191152 | XP_005191209 |
|  | LOC101894458 | XM_005190481 | XP_005190538 |
|  | LOC101889102 | XM_005175706 | XP_005175763 |
|  | LOC101889102 | XM_005175707 | XP_005175764 |
|  | LOC101890186 | XM_005180087 | XP_005180144 |
|  | LOC101898885 | XM_005184873 | XP_005184930 |
|  | LOC101901477 | XM_005185226 | XP_005185283 |
|  | LOC101901477 | XM_005185227 | XP_005185284 |
|  | LOC101901477 | XM_005185228 | XP_005185285 |
|  | LOC101887384 | XM_005178055 | XP_005178112 |
| Esterase 5A | LOC101889364 | XM_005183883 | XP_005183940 |
| Esterase 5B | LOC101889009 | XM_005183881 | XP_005183938 |
