# Supplementary material for: Genome of the house fly, Musca domestica L., a global vector of diseases with adaptations to a septic environment
Source: Genome Biol. 2014 Oct 14;15:466. doi: 10.1186/s13059-014-0466-3 (PMC4195910; doi:10.1186/s13059-014-0466-3)
Supplement: Additional file 10: Figure S2. — Unrooted neighbor-joining tree showing the phylogenetic analysis of GSTs of M. domestica (MD, red) in relation to GSTs from D. melanogaster (DM, green). MUSCLE software was used to perform multiple sequence alignment [132]. The neighbor-joining method was applied to the multiple sequence alignment using MEGA 5.0 [133]. Distance bootstrap values of >70% (1,000 replicates) are indicated at the corresponding nodes. The GST classes are colored distinctively: microsomal, turquoise; sigma, dark blue; omega, orange; zeta, dark red; theta, pink; delta, light blue and epsilon, light green. Sequences and the names for the D. melanogaster GST genes were taken from FlyBase [60]. [file 13059_2014_466_MOESM10_ESM.pptx]

## Slide 1
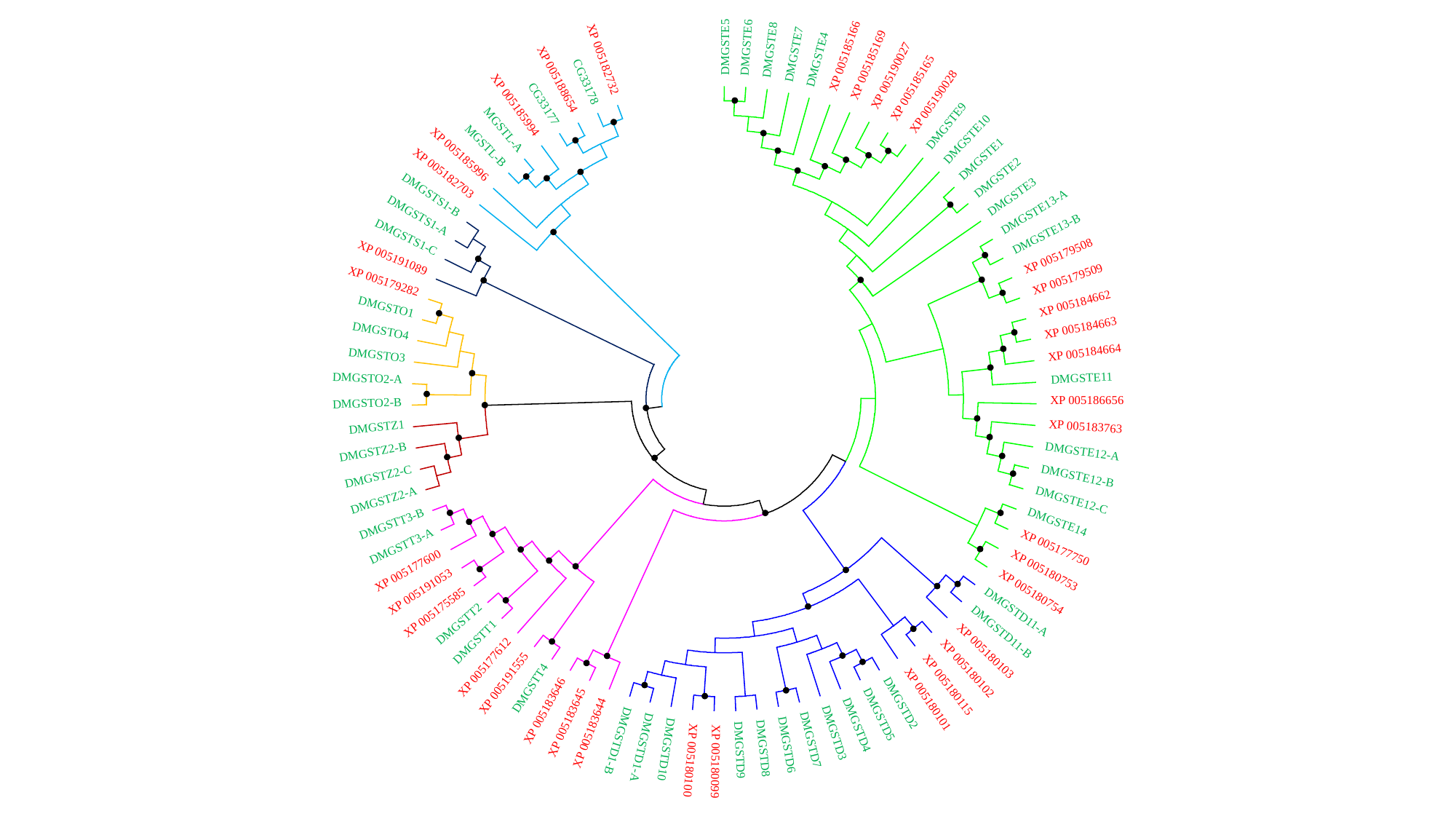

DMGSTE5
DMGSTE6
DMGSTE8
DMGSTE7
XP 005185166
DMGSTE4
XP 005185169
XP 005190027
XP 005185165
XP 005190028
DMGSTE9
DMGSTE10
DMGSTE1
DMGSTE2
DMGSTE3
DMGSTE13-A
DMGSTE13-B
XP 005179508
XP 005179509
XP 005184662
XP 005184663
XP 005184664
DMGSTE11
XP 005186656
XP 005183763
DMGSTE12-A
DMGSTE12-B
DMGSTE12-C
DMGSTE14
XP 005177750
XP 005180753
XP 005180754
DMGSTD11-A
DMGSTD11-B
XP 005180103
XP 005180102
XP 005180115
XP 005182732
XP 005188654
CG33178
CG33177
XP 005185994
MGSTL-A
MGSTL-B
XP 005185996
XP 005182703
DMGSTS1-B
DMGSTS1-A
DMGSTS1-C
XP 005191089
XP 005179282
DMGSTO1
DMGSTO4
DMGSTO3
DMGSTO2-A
DMGSTO2-B
DMGSTZ1
DMGSTZ2-B
DMGSTZ2-C
DMGSTZ2-A
DMGSTT3-B
DMGSTT3-A
XP 005177600
XP 005191053
XP 005175585
DMGSTT2
DMGSTT1
XP 005177612
XP 005191555
DMGSTT4
XP 005180101
DMGSTD2
XP 005183646
DMGSTD5
XP 005183645
DMGSTD4
XP 005183644
DMGSTD3
DMGSTD7
DMGSTD1-B
DMGSTD6
DMGSTD1-A
DMGSTD8
DMGSTD10
DMGSTD9
XP 005180100
XP 005180099
