# Supplementary material for: Genome of the house fly, Musca domestica L., a global vector of diseases with adaptations to a septic environment
Source: Genome Biol. 2014 Oct 14;15:466. doi: 10.1186/s13059-014-0466-3 (PMC4195910; doi:10.1186/s13059-014-0466-3)
Supplement: Additional file 12: Table S10. — Details of MdOBP family genes and proteins. [file 13059_2014_466_MOESM12_ESM.doc]

**Table S10 Details of MdOBP family genes and proteins.** Columns are: Gene – the gene and protein name we are assigning (suffixes are PSE – pseudogene; NTE – N-terminus unidentified; CTE C-terminus unidentified); Ortholog – the *Drosophila melanogaster* ortholog, if relevant (? indicates uncertainty); OGS – the official gene number in the 17508 REFSEQ proteins (prefix is XP_00); Scaffold – the genome assembly scaffold ID (amongst 20,487 scaffolds in assembly v2.0.2); Coordinates – the nucleotide range from the first position of the start codon to the last position of the stop codon in the scaffold; Strand – + is forward and - is reverse; Introns – number of introns in the coding region; AAs – number of encoded amino acids in the protein; Comments – comments on the OGS gene model, repairs to the genome assembly, and pseudogene status (numbers in parentheses are the number of obvious pseudogenizing mutations).

**Gene Ortholog OGS Scaffold Coordinates Strand Introns AAs Comments**

Obp1 Obp19a 5189784 2547 810-6955 - 2 147 Fine as is

Obp2 Obp19b 5183335 19084 295651-301726 + 4 152 Fine as is

Obp3 Obp19c 5183344 19084 289024-289726 - 2 151 Shortened second exon

Obp4NTE Obp19d 5190597 4178 3542-6935 + 4 114 N terminus missing

Obp5 Obp28a 5188786 20141 82482-83120 + 2 149 Fine as is

Obp6 Obp28a 5188784 20141 87383-89634 + 2 152 Fine as is

Obp7 Obp28a 5188787 20141 95501-96139 - 2 149 Fine as is

Obp8 Obp28a 5188785 20141 104002-104812 + 2 149 Fine as is

Obp9 Obp28a 5188788 20141 107604-108244 - 2 149 Fine as is

Obp10 Obp28a 5190164 3075 5396-5982 - 2 149 Fine as is

Obp11 Obp28a 5190165 3075 9287-9870 + 2 149 Fine as is

Obp12 Obp28a 5190166 3075 14734-17377 + 2 149 Fine as is

Obp13 Obp28a 5184499 19226 1862-4340 + 2 145 Fine as is

Obp14 Obp28a 5184493 19226 6819-8868 + 2 148 Fine as is

Obp15 Obp44a 5179081 18710 135921-136416 - 1 141 Fine as is

Obp16 Obp56b/c? - 20139 33485-33998 - 1 147 New gene model

Obp17 Obp56b/c? 5188766 20139 40633-41137 - 1 143 Fine as is

Obp18 Obp56b/c? 5188767 20139 45336-45832 - 1 143 Fine as is

Obp19 Obp56b/c? - 20139 55880-56374 + 1 143 New gene model

Obp20 Obp56b/c? - 20139 62956-63446 - 1 143 New gene model

Obp21 Obp56f/i? - 20139 63805-64337 + 1 155 New gene model

Obp22 Obp56a 5188768 20139 74042-74654 - 1 137 Fine as is

Obp23 Obp56a 5188762 20139 82770-83247 - 1 134 Fine as is

Obp24 Obp56a 5188763 20139 86453-86927 - 1 133 Fine as is

Obp25PSE Obp56a 5188769 20139 95579-96049 - 2 134 Pseudogene (1)

Obp26 Obp56a 5188764 20139 99512-99990 + 1 134 Fine as is

Obp27 Obp56d/e 5188765 20139 110820-111290 + 1 136 Fine as is

Obp28 Obp56d/e 5188770 20139 115940-116413 + 1 137 Fine as is

Obp29 Obp56f/i? - 19365 2448-2933 - 1 141 New gene model

Obp30 Obp56h? 5185234 19365 895063-907878 + 3 234 Remove internal exon

Obp31 Obp56h? 5185232 19365 894462-894943 + 1 140 Fine as is

Obp32 Obp56h? - 19365 910660-911121 + 1 134 New gene model

Obp33 Obp56h? - 19365 914295-914759 + 1 135 New gene model

Obp34 Obp56h? 5185235 19365 918231-921213 + 2 228 Extend second exon

Obp35 Obp56h? 5185237 19365 931776-932242 - 1 134 Fine as is

Obp36 Obp56h? 5185236 19365 923141-923921 + 1 141 Fine as is

Obp37 Obp56h? - 19365 928535-928789 + 1 149 New gene model

Obp38 Obp56h? 5185238 19365 937121-937575 + 1 129 Fine as is

Obp39 Obp57a-i 5187178 19740 70360-72579 + 1 153 Fine as is

Obp40NTE Obp57a-i - 19740 77512-77838 + 1 108 New gene model

Obp41 Obp57a-i 5187179 19740 78152-78651 + 1 126 Fine as is

Obp42 Obp57a-i 5187180 19740 83288-83706 - 1 119 Fine as is

Obp43 Obp57a-i 5187181 19740 86858-87382 - 1 137 Fine as is

Obp44 Obp57a-i 5187182 19740 90537-90985 - 1 130 Fine as is

Obp45 Obp57a-i 5187177 19740 96535-97006 - 1 134 Extended N terminus

Obp46 Obp57a-i 5189169 20313 96568-97147 - 1 129 Fine as is

Obp47 Obp69a 5177698 18633 225459-228939 - 4 145 Fine as is

Obp48 Obp76aLUSH 5187266 19762 36325-38142 - 4 149 Fine as is

Obp49 - 5176972 18609 608861-616276 + 3 147 Fine as is

Obp50 - 5176973 18609 620710-628583 - 3 148 Fine as is

Obp51 Obp83a/b? 5176974 18609 657153-658056 + 3 155 Fine as is

Obp52 Obp83g 5176964 18609 20923-21446 + 1 149 Fine as is

Obp53 Obp83c/d 5176975 18609 28974-29887 + 2 251 Double-OBP

Obp54 Obp83e/f 5176966 18609 34514-35502 - 2 259 Double-OBP

Obp55 Obp84a 5185930 19488 47515-56921 + 4 150 Multiple changes

Obp56 Obp99a 5189390 20435 71286-71783 + 1 144 Fine as is

Obp57 Obp99a 5189394 20435 79288-79805 + 1 144 Fine as is

Obp58 Obp99c/8a 5185305 19368 395067-395583 + 1 152 Fine as is

Obp59 Obp99d 5186856 19667 141104-141667 - 1 163 Fine as is

Obp60 Obp99b 5192089 908 3426-7799 - 1 150 Fine as is

Obp61CTE - 5192092 908 18348-18798 - 1 124 C terminus missing

Obp62 - 5192093 908 20292-20791 + 1 146 Multiple changes

Obp63 - 5192090 908 31491-32030 + 1 154 Fine as is

Obp64 - - 908 33540-34078 - 1 150 New gene model

Obp65 - 5192091 908 44224-44699 + 1 138 Fine as is

Obp66 - 5192094 908 52357-52842 - 1 138 Fine as is

Obp67 - 5187748 19868 142551-143037 - 1 141 Fine as is

Obp68 - 5187749 19868 147184-147661 - 1 132 Fine as is

Obp69 - - 560 2388-2952 + 1 166 New gene model

Obp70 - 5191173 560 7233-7718 - 1 138 Fine as is

Obp71 - - 560 12997-13468 + 1 136 New gene model

Obp72 - - 560 13848-14324 + 1 138 New gene model

Obp73PSE - - 560 18399-18869 - 1 136 Pseudogene (1)

Obp74 - 5191174 560 20067-20522 + 0 151 Fine as is

Obp75 - 5180291 18801 338026-338499 - 1 138 Fine as is

Obp76 - 5189513 2087 12469-16283 + 3 150 Fine as is

Obp77 - - 1705 16371-16981 + 3 142 New gene model

Obp78 - - 1705 21179-21862 + 4 143 New gene model

Obp79NTE - - 1705 26963-27748 + 2 104 New gene model

Obp80 - 5175762 1705 28271-31053 + 4 145 Fine as is

Obp81 - - 1794 8296-9481 - 4 139 New gene model

Obp82 - 5175828 1794 24152-24982 - 4 158 Fine as is

Obp83INT - - 1794 27789-28585 - 3 135 New gene model

Obp84 - 5190378 3684 230-1988 - 3 147 Multiple changes

Obp85 - - 3684 2585-11548 + 4 150 New gene model

Obp86 - - 13431 869-1958 + 4 152 New gene model

Obp87NTE - - 18554 454-967 - 3 107 New gene model
