# Supplementary material for: Genome of the house fly, Musca domestica L., a global vector of diseases with adaptations to a septic environment
Source: Genome Biol. 2014 Oct 14;15:466. doi: 10.1186/s13059-014-0466-3 (PMC4195910; doi:10.1186/s13059-014-0466-3)
Supplement: Additional file 13: Table S11. — Details of MdOR family genes and proteins. [file 13059_2014_466_MOESM13_ESM.doc]

**Table S11 Details of MdOR family genes and proteins.** Columns are: Gene – the gene and protein name we are assigning (suffixes are PSE – pseudogene; FIX – assembly was repaired; JOI – gene model spans scaffolds; NTE – N-terminus unidentified; CTE C-terminus unidentified; INT- internal regions unidentified; multiple suffixes are abbreviated to single letters); Ortholog – the *Drosophila melanogaster* ortholog, if relevant; OGS – the official gene number in the 17508 REFSEQ proteins (prefix is XP_00); Scaffold – the genome assembly scaffold ID (amongst 20,487 scaffolds in assembly v2.0.2); Coordinates – the nucleotide range from the first position of the start codon to the last position of the stop codon in the scaffold; Strand – + is forward and - is reverse; Introns – number of introns in the coding region; AAs – number of encoded amino acids in the protein; Comments – comments on the OGS gene model, repairs to the genome assembly, and pseudogene status (numbers in parentheses are the number of obvious pseudogenizing mutations).

**Gene Ortholog OGS Scaffold Coordinates Strand Introns AAs Comments**

OrCo OrCo/83b 5175278 1259 14618-39294 + 6 478 Fine as is

Or1JOI DpOrN - 1870 <1-704 - 5 410 Join across scaffolds

5190411 3803 1138->12456 -

Or2 Or1a 5191432 6947 296-4448 - 4 397 Adjust intron

Or3FIX Or1a 5192136 926 7639->8205 + 4 394 Fix assembly

5192135 926 <1-1303 +

Or4 Or2a 5188267 2 1376434-1380567 - 2 403 Fine as is

Or5 - 5187291 19768 143389-145795 - 2 409 Fine as is

Or6 - 5187290 19768 138355-141817 - 2 392 Fine as is

Or7PSE Or7a 5190908 469 31864-35096 - 2 301 Pseudogene (2)

Or8 Or7a 5190907 469 18390-27574 - 3 395 Fine as is

Or9 Or7a 5190906 469 1710-3196 - 3 435 Fine as is

Or10 Or7a 5189494 2052 6088-7581 + 3 437 Fine as is

Or11 Or10a 5178484 18678 341323-347596 + 4 404 Fine as is

Or12 Or13a 5191323 622 48571-53025 - 4 429 Fine as is

Or13 - 5189591 2206 16714-25745 - 5 458 Fine as is

Or14 - 5189590 2206 850-7860 - 5 461 Multiple changes

Or15JOI Or22c 5187358 19788 164641->167811 + 5 398 Join across scaffolds

5191818 840 <1-15841 +

Or16 Or24a 5181476 18895 47341-53063 - 5 399 Fine as is

Or17 Or30a 5181900 18939 276808-280725 - 5 373 Fine as is

Or18 Or30a 5187208 19749 35029-43469 + 5 373 Fine as is

Or19 - 5190189 3153 9025-11972 + 4 390 Fine as is

Or20PSE - 20052 61513-67014 - 4 378 Pseudogene (1)

Or21 Or33a-c 5185739 19448 85849-87055 + 1 372 Fine as is

Or22 multiple 5187966 19928 114212-116248 + 2 433 Fine as is

Or23 Or43a 5175768 172 5489-13041 - 6 375 Remove final exon

Or24 Or45a 5184443 19219 250742-255417 + 4 409 Fine as is

Or25 Or45a 5191600 707 52120-53601 - 4 409 Fine as is

Or26 Or45a 5191599 707 45043-49510 - 4 410 Extend N-terminus

Or27 Or45a 5191598 707 28376-37151 - 4 410 Remove double exon

Or28 Or45a 5191597 707 21161-23884 - 4 401 Fine as is

Or29IP Or45a - 707 17156-18697 - 4 379 Pseudogene (1)

Or30JI Or45a - 6047 <1-630 - 4 392 Join across scaffolds

- 11325 2175->2947 -

Or31 Or45a 5176399 18582 264366-270628 + 4 412 Fine as is

Or32PSE Or45a 5176400 18582 279858-283416 + 4 401 Pseudogene (1)

Or33 Or45a - 18582 286433-293007 + 4 401 New gene model

Or34 Or46aA/B 5190201 3215 667-4827 + 2 390 Fine as is

Or35 Or46aA/B 5180017 18775 6136-10301 + 2 387 Change intron

Or36 Or46aA/B 5190949 4876 3163-7162 - 2 388 Extend N-terminus

Or37 Or49a/85f 5180070 18780 379848-399553 - 3 405 Remove an intron

Or38 Or49a/85f 5180069 18780 367541-370610 - 3 416 First half of model

Or39 Or49a/85f 5180069 18780 362526-365181 - 4 417 Second half of model

Or40 Or49a/85f 5180068 18780 355026-357699 - 3 405 Fine as is

Or41 Or49a/85f 5180067 18780 350440-347195 + 3 415 Extend N-terminus

Or42 Or49b 5179498 18733 464-7606 + 5 371 Fine as is

Or43 Or59a 5184787 19276 22286-28146 + 1 398 Fine as is

Or44 - 5182355 18985 218891-220146 + 1 381 Multiple changes

Or45 - 5182356/7 18985 224017-225238 + 1 385 Join two models

Or46 - 5182358 18985 228629-231719 + 1 381 Extend N-terminus

Or47 - 5182359 18985 235409-238825 + 1 379 Fine as is

Or48 - 5186318 19571 23826-25032 + 1 381 Fine as is

Or49 Or63a 5178182 18661 230762-246942 - 9 415 Fine as is

Or50 - 5182424 1899 14153-21208 - 8 418 Fine as is

Or51JP - - 1899 24301->37688 + 9 374 Join across scaffolds

5189964 282 <1-5485 + Pseudogene (2)

Or52 - 5189964 282 9213-18804 + 9 415 Second part of model

Or53 Or67d 5191896 86 43081-47468 + 4 392 Fine as is

Or54 Or67d 5191897 86 59515-63895 + 4 391 Fine as is

Or55CTE Or67d 5190382 371 <35334-39602 - 2 283 C-terminus missing

Or56 Or67d 5190383 371 45516-50769 + 3 389 Remove an exon

Or57 Or67d 5190384 371 55524-57553 + 3 401 Fine as is

Or58 Or67d 5190385 371 60142-64096 + 3 392 Change N-terminus

Or59 Or67d 5184154 19194 200473-205423 + 3 401 Fine as is

Or60 Or67d 5187726 1986 5170-6520 - 3 393 Fine as is

Or61PSE Or67d 5187725 1986 551-1914 - 3 393 Pseudogene (1)

Or62NTE Or67d 5187292 19769 <155517-156592 + 3 315 N-terminus missing

Or63INT Or67d - 19329 84501-94471 + 3 340 Internal exon missing

Or64CTE Or67d 5185047 19329 100221->101311 + 3 320 C-terminus missing

Or65 Or67d 5176290 18578 784217-787484 + 3 392 Add N-terminal exon

Or66 Or69aA/B 5180133 18785 70492-77903 + 4 414 Fine as is

Or67 Or35a/74a 5188535 20038 58824-61223 - 4 405 Fine as is

Or68CTE Or35a/74a 5188534 20038 <52090-53182 - 3 325 C-terminus missing

Or69 Or35a/74a 5185292 19367 744621-746455 + 4 404 Fine as is

Or70 Or82a 5189776 254 14537-21410 - 5 382 Extend N-terminus

Or71CTE Or85b-d 5186501 19612 <131090-132093 - 1 314 First part of model

Or72PSE Or85b-d 5186501 19612 117647-123777 - 3 407 Pseudogene (1)

Or73 Or85b-d - 19612 109012-113608 - 3 430 New gene model

Or74CTE Or85b-d 5186500 19612 27601-33931 + 3 431 Last exon missing

Or75 Or85b-d - 19158 246021-256220 - 3 422 New gene model

Or76 Or83a 5189143 20298 44324-57416 - 5 453 Fine as is

Or77 Or83a 5189142 20298 30730-38016 - 5 480 Fine as is

Or78 Or85e 5182622 19003 148795-151719 + 4 462 Fine as is

Or79 Or88a 5179127 18710 611653-616793 + 3 409 Fine as is

Or80 Or94a/b 5185000 19322 151735-155579 + 3 394 Fine as is

Or81 - 5180861 18837 332652-347139 - 2 393 Fine as is

Or82 - 5181716 18920 328080-337530 - 4 399 Fine as is

Or83 - 5186825 19662 78318-83939 + 6 381 Fine as is

Or84A - - 18719 42819-57451 + 3 354 Alternatively spliced?

Or84B - 5179301 18719 54021-57451 + 3 354 Fine as is
