# Supplementary material for: Genome of the house fly, Musca domestica L., a global vector of diseases with adaptations to a septic environment
Source: Genome Biol. 2014 Oct 14;15:466. doi: 10.1186/s13059-014-0466-3 (PMC4195910; doi:10.1186/s13059-014-0466-3)
Supplement: Additional file 14: Table S12." — Details of MdGR family genes and proteins. [file 13059_2014_466_MOESM14_ESM.doc]

**Table S12 Details of MdGR family genes and proteins.** Columns are: Gene – the gene and protein name we are assigning (suffixes are PSE – pseudogene; FIX – assembly is repaired; NTE – N-terminus unidentified; CTE C-terminus unidentified; INT – internal regions unidentified; multiple suffices are abbreviated to single letters); Ortholog – the *Drosophila melanogaster* ortholog, if relevant; OGS – the official gene number in the 17508 REFSEQ proteins (prefix is XP_00); Scaffold – the genome assembly scaffold ID (amongst 20,487 scaffolds in assembly v2.0.2); Coordinates – the nucleotide range from the first position of the start codon to the last position of the stop codon in the scaffold; Strand – + is forward and - is reverse; Introns – number of introns in the coding region; AAs – number of encoded amino acids in the protein; Comments – comments on the OGS gene model, repairs to the genome assembly, and pseudogene status (numbers in parentheses are the number of obvious pseudogenizing mutations).

**Gene Ortholog OGS Scaffold Coordinates Strand Introns AAs Comments**

Gr1.1 Gr21a 5189990 285 69789-75453 + 3 453 Fine as is

Gr1.2 Gr21a 5189991 285 76436-78642 - 3 455 Fine as is

Gr3 Gr63a 5181396 18880 372106-387682 - 6 489 Fine as is

Gr4JFI Gr61a - 15573 763->1242 + ? 319 Join across scaffolds,

5175036 1064 <1-800 + partially fixed

Gr5NI Gr5a 5186933 1969 31211->34076 - ? 333 Exons missing

Gr6FI Gr64a - 6065 3997->4806 - ? 282 Exons missing

Gr7CTE Gr64b 5191267 6065 <1-3189 - ? 237 C-terminus missing

Gr8CTE Gr64c 5190447 3945 <1-2360 - ? 238 C-terminus missing

Gr9CTE Gr64d 5189491 2050 23499->36814 + ? 360 C-terminus missing

Gr10JIN Gr64e - 2050 <1->948 - ? 364 Join across scaffolds,

5175753 1689 22764->40124 - exons still missing

Gr11 Gr63f 5175752 1689 336-9104 - 7 463 Fine as is

Gr12 Gr43a 5178823 18699 439229-442148 - 9 425 Multiple changes

Gr13 Gr43a 5178821 18699 422167-428969 - 9 419 Fine as is

Gr14 Gr32a 5186853 19666 70762-88011 - 3 464 Fine as is

Gr15a Gr39a - 18712 48035-87057 + 3 373 Alternatively spliced

Gr15b Gr39a - 18712 53136-87057 + 3 381 Alternatively spliced

Gr15c Gr39a - 18712 59745-87057 + 3 383 Alternatively spliced

Gr15d Gr39a 5179166 18712 72042-87057 + 3 383 Alternatively spliced

Gr16 Gr2a 5191421 684 32313-35339 - 5 392 Fine as is

Gr17 - 5191420 684 29446-31275 + 2 397 Fine as is

Gr18 Gr23aA 5191419 684 24546-28659 - 2 413 Fine as is

Gr19FIX Gr39b? 5181901 18939 159488-154106 + 2 463 Rearrange assembly

Gr20 Gr98a? 5189065 20264 34772-36140 + 3 393 First part of model

Gr21 Gr98a? 5189065 20264 39085-41408 + 3 388 Second part of model

Gr22PC Gr8a - 19673 141730-142857 - 3 336 Pseudogene (1)

Gr23CTE Gr8a - 19673 136453-137585 - 3 335 C-terminus missing

Gr24CTE Gr8a - 19673 131097-132229 - 3 335 C-terminus missing

Gr25CTE Gr8a - 19673 127180-128309 - 3 335 C-terminus missing

Gr26PC Gr8a - 19673 123080-124206 - 3 329 Pseudogene (1)

Gr27 Gr8a 5186881 19673 117884-120861 - 4 382 Fine as is

Gr28 Gr8a 5186880 19673 110609-113446 - 4 384 Fine as is

Gr29a - - 18827 79063-92267 + 4 385 Alternatively spliced

Gr29bPSE - - 18827 84046-92267 + 4 383 Pseudogene (1)

Gr29c - - 18827 88989-92267 + 4 378 Alternatively spliced

Gr30JOI Gr98b-d 5175352 1306 <1-17200 - 3 413 Join across scaffolds

- 7039 84->3257

Gr31 Gr98b-d 5175353 1306 17331-22890 + 3 433 Fine as is

Gr32 Gr98b-d 5175354 1306 23596-25042 + 3 420 Fine as is

Gr33 Gr98b-d 5192087 906 24142-29830 - 3 422 N-terminal extension

Gr34 Gr98b-d 5185576 19414 172280-178049 - 3 412 Multiple changes

Gr35 Gr98b-d 5185575 19414 160527-169024 - 3 417 Multiple changes

Gr36 Gr66a 5187565 19833 60993-70533 + 4 541 Fine as is

Gr37 Gr66a-like 5188739 20125 57443-60506 - 4 523 Fine as is

Gr38NTE Gr33a 5182527 18998 <174966-180732 + 4 436 N-terminus unidentified

Gr39aJOI Gr28b 5192001 891 21439->56382 + 3 452 Alternatively spliced

28 55446->87922 - across two scaffolds

Gr39bJOI Gr28b 5192002 891 46833->56382 + 3 445 Alternatively spliced

28 55446->87922 - across two scaffolds

Gr39c Gr28b 5189945 28 55466-83211 - 3 456 Alternatively spliced

Gr39d Gr28b 5189944 28 55466-73445 - 3 455 Alternatively spliced

Gr39e Gr28b 5189943 28 55446-70806 - 3 469 Alternatively spliced

Gr39fPSE Gr28b - 28 55446-66092 - 3 448 Pseudogene (1)

Gr39g Gr28b 5189942 28 55466-60663 - 3 446 Alternatively spliced

Gr40 Gr28a 5189942 28 40543-47188 - 3 447 Fine as is

Gr41 - - 836 18429-42968 + 2 383 New gene model

Gr42 Gr10a 5178485 18678 379564-380858 - 1 409 Extend C-terminus

Gr43 - - 18678 357626-358800 + 1 360 New gene model

Gr44a Gr59a/b - 18678 349230-354576 - 1 395 Alternatively spliced

Gr44b Gr59a/b - 18678 349230-350663 - 1 392 Alternatively spliced

Gr45c Gr59a/b - 18678 336053-337528 + 1 394 Alternatively spliced

Gr45b Gr59a/b - 18678 333406-337528 + 1 395 Alternatively spliced

Gr45a Gr59a/b - 18678 329440-337528 + 1 390 Alternatively spliced

Gr46 - - 18678 326288-327472 + 1 373 New gene model

Gr47 - - 18678 324073-325249 + 1 371 New gene model

Gr48PSE 36a-c/59c/d 5178483 18678 319571-322456 - 1 404 Pseudogene (1)

Gr49 - - 18678 317354-318546 + 1 373 New gene model

Gr50 - - 18678 314575-315829 - 1 396 New gene model

Gr51PSE 36a-c/59c/d 5178482 18678 311956-313233 + 1 405 Pseduogene (1)

Gr52a 36a-c/59c/d 5178481 18678 279584-309355 - 1 403 Alternatively spliced

Gr52b 36a-c/59c/d 5178481 18678 279584-307588 - 1 397 Alternatively spliced

Gr52c 36a-c/59c/d 5178480 18678 279584-305495 - 1 397 Alternatively spliced

Gr52d 36a-c/59c/d 5178480 18678 279584-303691 - 1 397 Alternatively spliced

Gr52ePSE 36a-c/59c/d - 18678 279584-301453 - 1 392 Pseudogene (1)

Gr52fPSE 36a-c/59c/d - 18678 279584-299911 - 1 412 Pseudogene (1)

Gr52g 36a-c/59c/d - 18678 279584-298106 - 1 397 Alternatively spliced

Gr52h 36a-c/59c/d 5178479 18678 279584-296339 - 1 404 Alternatively spliced

Gr52i 36a-c/59c/d - 18678 279584-290858 - 1 400 Alternatively spliced

Gr52j 36a-c/59c/d 5178478 18678 279584-285373 - 1 402 Alternatively spliced

Gr52k 36a-c/59c/d 5178477 18678 279584-282208 - 1 419 Fine as is

Gr53 - - 18678 122384-123557 + 1 367 New gene model

Gr54 - - 18678 133141-134324 + 1 369 New gene model

Gr55 - - 18678 140029-141190 + 1 367 New gene model

Gr56 - - 18678 144294-145425 + 1 359 New gene model

Gr57PSE - - 18678 147329-148506 + 1 370 Pseudogene (1)

Gr58 - - 18678 152167-153358 + 1 378 New gene model

Gr59PSE - - 18678 156155-157298 + 1 362 Pseudogene (1)

Gr60 - - 18678 159634-160821 + 1 374 New gene model

Gr61PSE - - 18678 161837-162921 - 1 338 Pseudogene (1)

Gr62PSE - - 18678 169147-170081 - 1 283 Pseudogene (3)

Gr63PSE - - 18678 170725-171857 - 1 358 Pseudogene (1)

Gr64 - - 18678 174442-175635 - 1 374 New gene model

Gr65 Gr47b 5174911 1 1987208-1989215 - 2 415 Multiple changes

Gr66 Gr57a 5174809 0 1431749-1433247 + 2 404 Fine as is

Gr67 Gr58c - 19367 732706-733941 + 1 389 New gene model

Gr68 Gr58b 5185293 19367 762569-763834 - 1 400 Multiple changes

Gr69 - - 596 55401-56661 + 1 395 New gene model

Gr70 Gr59e 5179131 18710 1279014-1290743 - 2 441 Fine as is

Gr71 Gr59f 5179132 18710 1298830-1300330 + 3 429 Fine as is

Gr72 Gr77a 5190780 4456 337245-338685 - 1 431 Multiple changes

Gr73 Gr89a 5180112 18783 35388-36724 - 1 398 Extend ends

Gr74 Gr93a 5176123 18572 224330-225661 - 1 419 Remove N-terminus

Gr75a Gr94a/97a 5183039 19047 122249-131605 + 2 397 Alternatively spliced

Gr75b Gr94a/97a 5183040 19047 126129-131605 + 2 398 Alternatively spliced

Gr76 - - 19009 36100-37925 + 3 414 New gene model
