# Supplementary material for: Genome of the house fly, Musca domestica L., a global vector of diseases with adaptations to a septic environment
Source: Genome Biol. 2014 Oct 14;15:466. doi: 10.1186/s13059-014-0466-3 (PMC4195910; doi:10.1186/s13059-014-0466-3)
Supplement: Additional file 15: Table S13. — Details of MdIR family genes and proteins. [file 13059_2014_466_MOESM15_ESM.doc]

**Table S13 Details of MdIR family genes and proteins.** Columns are: Gene – the gene and protein name we are assigning (suffixes are NTE – N-terminus missing; CTE – C-terminus missing; INT – internal sequence missing; JOI – joined across scaffolds; PSE – pseudogene; multiple suffixes are abbreviated to single letters; Ortholog – the *Drosophila melanogaster* ortholog, if relevant; OGS – the official gene number in the 17508 REFSEQ proteins (prefix is XP_00); Scaffold – the genome assembly scaffold ID (amongst 20,487 scaffolds in assembly v2.0.2); Coordinates – the nucleotide range from the first position of the start codon to the last position of the stop codon in the scaffold; Strand – + is forward and - is reverse; Introns – number of introns in the coding region; AAs – number of encoded amino acids in the protein; Comments – comments on the OGS gene model, repairs to the genome assembly, and pseudogene status (numbers in parentheses are the number of obvious pseudogenizing mutations).

**Gene Ortholog OGS Scaffold Coordinates Strand Introns AAs Comments**

IR8a IR8a 5183869 19149 247856-254643 + 8 928 Fine as is

IR10a1 - 5179336 18721 91332-96653 + 1 682 First part of model

IR10a2 - 5179336 18721 100541-104085 + 1 617 Second part of model

IR10a3IP - - 18721 108645-111644 + 1 527 Pseudogene (1)

IR10a4IP - - 18721 116749-119696 + 1 527 Pseudogene (1)

IR10a5 IR10a 5179337 18721 125114-129146 + 1 638 Fine as is

IR21a IR21a 5178446 18677 277973-287771 + 5 892 Fine as is

IR25a IR25a 5175883 1821 250287-261352 + 8 941 Fine as is

IR31aNJ IR31a - 2422 <19162->24046 + 5 588 Join across scaffolds

5191726 56704 <1->56704 + First exon unidentified

- 1364 <1->42209 +

- 2155 10768->27093 -

IR40aNJ IR40a 5191713 7881 <1->1721 - 7 700 Join across scaffolds

5190185 3136 10415->16682 - N-terminus missing

IR41a IR41a 5180963 18847 98806-102335 - 9 655 Fine as is

IR60a IR60a 5185287 19367 1063921-1066719 + 3 700 Fine as is

IR64a IR64a 5185688 19434 82641-105823 - 13 885 Change both ends

IR68a IR68a 5180224 18796 385231-388031 - 4 662 Fine as is

IR68b IR68b 5179068 18709 596437-598476 + 0 679 Fine as is

IR75a IR75a 5176740 18600 227387-234184 + 6 634 First half of model

IR75b IR75a-c 5176740 18600 238614-242009 + 6 637 Second half of model

IR75d IR75d 5190942 480 31799-36599 - 4 665 Fine as is

IR76a1 IR76a 5177868 18644 227877-235272 + 7 645 First part of model

IR76a2 IR76a 5177868 18644 245243-252803 + 7 656 Second part of model

IR76a3 IR76a 5177869 18644 257647-265081 + 7 625 Add an exon

IR76b IR76b 5179851 18760 100662-105925 - 4 652 Fine as is

IR84a1 IR84a 5178703 18689 421735-427758 - 5 591 Fine as is

IR84a2 IR84a 5178702 18689 414500-418659 - 5 545 Fine as is

IR85a IR85a - 18858 18478-20389 + 1 616 New gene model

IR87a IR87a 5180602 18821 809480-814182 - 3 785 Fine as is

IR92aJIN IR92a 5190306 4471 <1->10885 - 8 405 Join across scaffolds

3540 15491->16948 - Two exons missing

IR93a IR93a 5184900 19301 191038-212973 - 12 859 Fine as is

IR94e IR94e 5179663 18746 461914-468825 - 1 672 Fine as is

IR100a IR100a - 18721 131115-132933 + 1 595 New gene model

IR101 IR7a/11a 5178410 18674 335241-341690 - 2 634 First part of model

IR102 IR7a/11a 5178410 18674 326041-332385 - 2 639 Second part of model

IR103 IR7a/11a 5178410 18674 314180-317581 - 2 643 Third part of model

IR104 IR7c 5178138 18656 272797-279060 - 2 638 First part of model

IR105 IR7c 5178138 18656 264318-268661 - 2 636 Second part of model

IR106 IR7c 5178138 18656 245506-248870 - 2 641 Third part of model

IR107NTE IR7b 5178138 18656 208202->217103 - 2 517 N-terminus missing

IR108 - 5178137 18656 190774-196551 + 2 613 First part of model

IR109NTE - 5178137 18656 <197481-198497 + 1 338 N-terminus missing

IR110PSE IR7d - 18656 101119-105637 - 2 613 Pseudogene (2)

IR111 IR7d 5178136 18656 89125-96453 - 2 604 Remove final exon

IR112 IR7c 5178135 18656 82236-85630 + 2 640 Fine as is

IR113 - 5178134 18656 73719-77909 + 2 660 Fine as is

IR114 - 5178133 18656 66717-72070 + 2 642 Fine as is

IR115 - 5178132 18656 56987-61102 + 2 648 Fine as is

IR116 - 5178131 18656 45076-49354 - 2 629 Fine as is

IR117 IR7g 5178130 18656 38651-43220 - 2 603 Fine as is

IR118 IR7e 5178129 18656 31449-36660 + 2 593 Fine as is

IR119 IR7f 5178128 18656 25277-27193 - 2 597 Remove final 2 exons

IR120 IR7f 5178127 18656 10414-20217 - 2 597 Remove internal exon

IR121 IR7f 5178126 18656 2546-6053 - 2 599 Extend final exon

IR122NTE IR7f 5191646 7398 <1299-2291 + 1 330 First two exons missing

IR123PSE - 5184768 19274 233854-239060 - 1 634 Pseudogene (1)

IR124 - 5184767 19274 217743-227601 - 1 619 First part of model

IR125PSE - 5184767 19274 207266-211734 - 1 609 Pseudogene (2)

IR126 - 5184766 19274 198587-204478 - 1 612 Multiple changes

IR127 IR56d 5181637 18909 81037-82923 + 0 628 Remove second exon

IR128 IR56d 5181638 18909 86403-88256 + 0 617 Fine as is

IR129 IR56d 5181639 18909 93957-95831 + 0 624 Fine as is

IR130 IR56c 5181640 18909 98771-100588 - 0 605 Fine as is

IR131 IR56c 5181641 18909 104138-105940 - 0 600 Multiple changes

IR132 IR56c 5181642 18909 108418-110322 - 0 638 Multiple changes

IR133 IR56c 5181643 18909 110503-112353 + 0 616 Fine as is

IR134 IR56c 5181644 18909 116105-117937 + 0 610 Fine as is

IR135 IR56a 5181645 18909 121531-123494 + 1 636 Fine as is

IR136 IR56a 5184909 19303 117813-123919 + 1 614 Fine as is

IR137 IR56b 5179573 18738 2700-3872 - 0 390 Remove second exon

IR138 IR62a 5184782 19275 143400-145319 + 0 639 Multiple changes

IR139 IR62a 5184783 19275 148695-150563 - 0 622 Fine as is

IR140 IR62a 5184784 19275 155161-157080 + 0 639 First half of model

IR141 IR62a 5184784 19275 159783-161699 + 0 638 Second half of model

IR142 IR62a 5184785 19275 164105-166003 - 0 632 Extend N-terminus

IR143 IR62a 5184786 19275 177534-179447 + 0 637 Extend N-terminus

IR144 - 5179832 18758 379590-381293 + 0 567 Fine as is

IR145 - 5179833 18758 384810-386513 + 0 568 Fine as is

IR146 - - 18813 36473-388224 - 0 583 New gene model

IR147CTE - - 18813 <26031-27671 - 0 564 C-terminus truncated

IR148PSE - 5190034 2932 16374-18155 + 0 593 Pseudogene (2)

IR149 - 5178424 18675 12348-14147 + 0 599 Fine as is

IR150 - 5190703 4453 282526-284319 + 0 597 Multiple changes

IR151 - 5175490 1445 11596-13451 + 0 631 Fine as is

IR152 - 5175491/2 1445 17496-19466 + 0 656 Fuse models

IR153 - 5175493 1445 25133-27028 + 0 631 Fine as is

IR154PSE - - 63 16421-18333 + 0 636 Pseudogene (2)

IR155 - 5191336 63 21911-23707 - 0 598 Remove intron

IR156 - - 63 28048-29841 + 0 597 New gene model

IR157 - - 63 37221-39035 + 0 604 New gene model

IR158 - 5191337 63 42735-44575 - 0 616 Extend N-terminus

IR159 - 5186065 19524 149485-151299 - 0 604 Multiple changes

IR160 - 5186064 19524 142325-144148 - 0 607 First part of model

IR161 - 5186064 19524 138727-140547 - 0 606 Second part of model

IR162 IR54a? 5186063 19524 133758-135581 - 0 607 Remove second exon

IR163 - 5189910 2752 6366-8186 - 0 606 Extend N-terminus

IR164 - 5189909 2752 1606-3444 - 0 612 Extend C-terminus

IR165 - 5189858 2640 11739-13577 + 0 612 Extend N-termiinus

IR166 - 5189859 2640 18561-20384 - 0 606 Extend N-terminus

IR167 - 5192155 938 19109-20920 + 0 603 Multiple changes

IR168 - 5190314 3550 10928-12739 + 0 603 Extend N-terminus

IR169CTE - 5175448 13904 <1-1799 - 0 599 C-terminus missing

IR170 - 5190132 3006 18716-20617 - 0 633 Extend N-terminus

IR171 - 5190131 3006 16383-18236 + 0 617 Extend N-terminus

IR172 - 5190130 3006 13701-15485 - 0 594 Fine as is

IR173 - 5187544 19832 73313-75316 + 0 667 Multiple changes

IR174 - 5189419 20454 79101-80870 + 0 589 Fine as is

IR175PSE - 5184920 20454 85240-86693 + 0 483 Pseudogene (2)

IR176 - 5175739 1670 9875-11539 - 0 554 Fine as is

IR177 - 5175740 1670 26983-28818 + 0 611 Multiple changes

IR178PSE - 5175741 1670 32654-34569 - 0 637 Pseudogene (2)

IR179 - 5185822 19471 75340-77133 - 0 597 Fine as is
