# Supplementary material for: Genome of the house fly, Musca domestica L., a global vector of diseases with adaptations to a septic environment
Source: Genome Biol. 2014 Oct 14;15:466. doi: 10.1186/s13059-014-0466-3 (PMC4195910; doi:10.1186/s13059-014-0466-3)
Supplement: Additional file 16: — Chemoreceptors, including protein sequences. [file 13059_2014_466_MOESM16_ESM.docx]

**Additional file for the *Musca domestica* odorant binding proteins and chemoreceptors**

**Methods**

These four gene families were manual annotated and analyzed with the aid of corrected distance phylogenetic trees. Although the methods for each of the four families were similar, the nature of the families required some differences, which are noted below. Briefly, BLASTP searches were performed on the available Official Gene Set of proteins in REFSEQ at NCBI. TBLASTN searches were also performed using all *Drosophila melanogaster* relatives, as well as all *Musca* *domestica* proteins, as queries. Gene models were manually assembled in TextWrangler. All of the Musca genes and encoded proteins are detailed in Supplementary Tables 5-8. All *M. domestica* proteins are provided below each family text in FASTA format.

Several difficulties with the genome assembly were encountered in these gene families. Common problems involved absence of exons in gaps between contigs within scaffolds or off ends of scaffolds (suffices NTE, CTE, and INT in the figures, tables, and proteins). Only a few of these gene models were corrected using raw reads (suffix FIX in the figures, tables, and proteins), because they commonly have large complicated introns and hence manual assembly repair is difficult. Several gene models were designed that span scaffolds, with no support other than the agreement of the available exons on both scaffolds, and their appropriate relatedness to similar genes (suffix JOI in the figures, tables, and proteins). These problems are noted in the Tables. Every family has multiple instances of genes on short scaffolds that are identical to ones in longer scaffolds and hence were ignored as likely resulting from separate assembly of another haplotype, as well as extremely short fragments of genes and some highly degraded pseudogenes. For the OBPs, there are two instances of identical genes that were nevertheless included in the gene set (MdObp5/7 and 61/68). These pairs of identical genes are in different locations within an array of genes in the same scaffold (5/7) or in arrays of genes on different large scaffolds (61/68), so they could be recent duplications within the genome, although they could also be the result of duplicate misassemblies. For the OR family, the highly conserved OrCo gene has the last two exons duplicated 4kb downstream, and the first four exons are duplicated at the 5’ end of another 231kb scaffold (and are modeled as XP_005184813). Both of these duplications were ignored on the grounds they are likely assembly artifacts due to polymorphisms, but even if real they are not worth including in analyses because they would be identical fragments. For the GR family, the major problem was the sugar receptor subfamily, due to fragmented assembly of this major gene array, where in some cases several exons are missing from otherwise conserved genes.

Pseudogenes were translated as best possible to provide an encoded protein that could be aligned with the intact proteins for phylogenetic analysis, and attention was paid to the number of pseudogenizing mutations in each pseudogene. The possible translations of pseudogenes had to be at least half the average length of the relevant proteins to be included in the analysis and there are several shorter fragments of genes that were not included (suffix PSE in the figures, tables, and proteins). Protein families were aligned in CLUSTALX v2.0 [1] using default settings with the relevant families of *D. melanogaster.* Problematic gene models and pseudogenes were refined in light of these alignments. Less obvious pseudogenes (for example with small in-frame deletions or insertions, crucial amino acids changes, or promoter defects) would not be recognized, so the provided gene totals might be high.

For phylogenetic analysis, the poorly aligned and variable length N-terminal and C-terminal regions were excluded from each family analysis, as well as an internal region of the ORs that does not align with the OrCo proteins, and several regions of major internal length differences in the IR family. Other regions of potentially uncertain alignment between these highly divergent proteins were retained, because while potentially misleading for relationships of the subfamilies (which are poorly supported anyway), they provide important information for relationships within subfamilies. Phylogenetic analysis involved a combination of model-based correction of distances between each pair of proteins, and distance-based phylogenetic tree building. Pairwise distances were corrected for multiple changes in the past using the BLOSUM62 amino acid exchange matrix in the maximum likelihood phylogenetic program TREEPUZZLE v5.2 [2]. These corrected distances were fed into PAUP*v4.0b10 [3] where a full heuristic distance search was conducted with tree-bisection-and-reconnection branch swapping to search for the shortest tree. Bootstrap analysis with 10,000 replications of neighbor-joining using uncorrected distances was performed to assess the confidence of branches, and are shown above major branches in the figures. Trees were manually colored and labels attached to lineages and subfamilies in Adobe Illustrator.

**The Odorant Binding Protein (OBP) family**

The OBPs are a family of small secreted globular proteins thought to function in binding and transporting hydrophobic compounds (e.g. [4]). Originally discovered as genes that are highly expressed in insect antennae, the gene family in some insects also contains members that are expressed elsewhere (e.g. [5]). Their binding of odorants is usually not highly specific, but they are thought to play an important role in olfaction by transporting hydrophobic ligands from the air through the sensillar lymph to the dendrites of olfactory sensory neurons, and some have been proposed to interact directly with olfactory receptors (but see [6]). They are expressed, often at high levels, in the support cells at the base of each sensillum, and secreted into the sensillar lymph. Most insects with complete genome sequences have been found to encode tens of these proteins. The family consists of several subtypes. The “classic” OBPs usually have six highly conserved cysteines, and three disulfide bonds between them maintain their tertiary structure in extracellular regions, however some have lost two of these cysteines and one disulfide bond. In addition, *Drosophila* flies have “double” OBPs where two “classic” OBP domains are fused into one protein. *M. domestica* has both of these kinds of OBP genes (see below for “Plus-C” OBPs) [7-10].

Eighty seven OBP genes were modeled (Supplementary Table 5). Four of these are double OBPs (MdObp30, 34, 53, and 54), so their OBP domains were separated for phylogenetic analysis and are indicated in the tree below with the suffixes a and b. 53 of these were already perfectly modeled, another 6 genes were partially modeled and only required minor fixes to the model, while six genes remain incomplete in the assembly. Two pseudogenes were included in the analyzed set. 22 new gene models are proposed. As is commonly the case in other insects, most of these genes are in arrays of multiple genes, albeit not always all in tandem, nor currently on the same scaffold. Their gene structures are fairly complicated, with 0-4 introns. The encoded proteins are generally of typical length for classic OBPs, except of course the four double OBPs.

The *M. domestica* OBPs were named roughly in order of the *Drosophila* gene numbering system, which is arbitrarily based on cytological position, except that DmObp8a and 18a do not have simple MdObp orthologs, so were skipped (Supplementary Table 5). There are three apparent housefly OBPs reported from antennal cDNAs in GenBank [11], however their sequences are enigmatic. They do not have good matches in this housefly genome assembly, and when included in the phylogenetic analysis (not shown in Supplementary Figure 4), they cluster very close to DmObp83a (OBP1/3) and Obp83b (OBP2). Because it is hard to understand the origin of these three OBPs, and because this genome assembly will serve as the reference genome sequence for housefly going forward, the OBP naming system here ignores these three genes/proteins and starts with a different MdObp1 gene/protein (the ortholog of DmObp19a).

Only the mature OBP peptides of about 120 amino acids can be confidently aligned, and then only the four regions surrounding the conserved cysteines can be utilized for phylogenetic analysis and even then are not very reliable (e.g. [12]). Nevertheless, given the relatively close relationship with *D. melanogaster*, to facilitate ortholog identification and analysis of gene family evolution a phylogenetic analysis was undertaken and the tree is in Figure Sw. Assignment of orthology following the tree is not always simple, given the relatively poor bootstrap support for many apparently clear relationships of these short proteins. While most simple apparent orthologous pairings are well supported, there are many complicated relationships. For example, in the middle of the tree the set of DmObp57a-e, which are in an interrupted and inverted array in the *Drosophila* genome, are apparently related to the set of MdObp39-45, which are in an array on 172 kb scaffold1974, and MdObp46, which is on its own in 127 kb scaffold20313. There is, however, no bootstrap support for this clustering, and unfortunately these are the only modeled genes in these two *M. domestica* scaffolds, so it is not possible to use microsynteny to further evaluate orthology (note that DmObp18a might be an escapee from the DmObp57a-e array, just as MdObp46 appears to be an escapee from the MdObp39-45 array). It is also therefore not possible to discern whether these two sets of genes duplicated independently in each fly lineage, or whether at least some gene duplications predate the fly lineage split.

Viewed broadly, there appear to be at least 30 orthologous or ancestral gene lineages in the OBP gene family in these two flies, implying that the common ancestor had at least that many OBP genes. Fifteen of these are simple 1:1 orthologous relationships, for example, MdObp48 is the ortholog of DmObp76a, which is also known as LUSH [13]. Another four have simple duplications in one or both species (e.g. MdObp56/57 are duplicates of DmOr99a). There are two instances of considerable gene lineage expansion in *M. domestica* compared with a single *Drosophila* gene (DmObp28a is expanded to MdObp5-14, which are on three separate scaffolds but compatible with being a single contiguous array in the genome, and DmObp56a is expanded to MdObp22-26). In addition to the complicated apparent relationship of DmObp57a-e and 18a with MdObp39-46 described above, there are several more apparent complicated relationships without bootstrap support in the tree. Thus while DmObp56a-i are again in a somewhat messy and interrupted array, their *M. domestica* relatives form two large arrays. MdObp16-28 constitute most of 119 kb scaffold20139 extending to the 3’ end of it, while their clear relative MdObp29 is at the 5’ end of 1,164 kb scaffold19365, suggesting that these scaffolds are adjacent in the genome. The remaining MdObp30-38 are ~900 kb further along in a second array in scaffold19365. The fact that these two sets of genes are in large arrays strongly supports their relationship, indicated on the right in Supplementary Figure 4, despite no bootstrap support (there is even an unrelated orthologous pair of DmObp84a/MdObp55 that clusters with them in the tree, along with DmObp51a, 22a, and 47a, although the latter is probably truly a transposed duplicate from DmObp56c), while DmObp56g does not even cluster with these. Furthermore, in this case it seems likely that some of these duplications occurred before these two fly lineages split, for example, there is bootstrap support for the clustering of DmObp56a with MdObp22-26 and for DmObp56d/e with MdObp27/28.

The double OBP MdObp53 and 54 genes are clear orthologs of the DmObp83c/d and e/f double-OBP genes, hence these genes are older than the split of the fly lineages. In contrast, the MdObp30 and 34 genes also encode double OBPs, which given their novel origin as duplications within the *M. domestica* lineage, indicates that such “double” OBPs can evolve easily by fusion of two duplicated “classic” genes.

The reason that *M. domestica* has 87 OBP genes versus the 37 classic OBPs in *Drosophila* (counting the two double OBPs, DmObp83c/d and 83e/f as single genes, in keeping with the *M. domestica* naming system), is the large and sometimes recent expansions of several *M. domestica* gene lineages, especially MdObp16-38, 39-46, 61-75 (which have no clear *Drosophila* ortholog, but are in an array with MdObp60 which is the ortholog of DmObp99b), and 77-87 (which have no *Drosophila* ortholog). In contrast, the few *Drosophila* expansions, like DmObp56a-i, Obp57a-e, and Obp83a-g are smaller and apparently older. In addition, *Drosophila* appears to have lost five lineages (double thickness blue lines in Supplementary Figure 4), while it is not clear that *M. domestica* has lost any, although the orthologs of the divergent and weakly clustering DmObp18a, 22a, and 51a might have been lost from *M. domestica*. Even discounting the two pseudogenes and two sets of identical genes, *M. domestica* has double the gene family size of *Drosophila*. This increase corresponds well with the increases in the numbers of Odorant, Gustatory, and Ionotropic Receptors described below, suggesting that the chemosensory repertoire of *M. domestica* is considerably larger than that of *Drosophila*.

Finally, Hekmat-Scafe et al. [8] described a highly divergent “subfamily” of OBPs in *D. melanogaster* called “Plus-C” OBPs that might contain the same conserved 6 cysteine motif, but also three conserved cysteines on either side of this central motif (Obp46a, 47b, 49a, 50a-e, 58b-d, 85a, and 93a). These proteins are so divergent they deserve their own family, and their involvement in chemosensation has not been established, although Jeong et al. [14] recently described a role for Obp49a in integration of sweet and bitter taste. *M. domestica domestica* has only six members of this “Plus-C” subfamily, compared with 12 in *D. melanogaster* (there are apparent orthologs for Obp47b, 49a, and 50e). The apparent contraction of this “subfamily” in *M. domestica* (or expansion in *Drosophila*) is in contrast to the expansion of the OBP, OR, GR, and IR families clearly involved in chemosensation, raising the question of whether they are indeed all involved in chemosensation (see [15]). Their protein sequences are included below.

**87 MdObps in FASTA format:**

>MdObp1

MISTMNILFAICAVVCIFRVQDVVGGATEEQMWAAGGLMRDVCLPKFPKVTKEIADGIRAGNLPNEKDAKCYVNCILEMMQTMKKGKFLYEASLKQVEILMPDHYKEEYRAGLAKCKDVAVGVKNNCEAAYTIFTCLRGEITKFVFP

>MdObp2

MHFCKHLFICLSLIAIAYADDDDDDIGMTSEELIDALEPFGENCDPKPDREHIRQLIKNDENPHQSSKCFRHCLMHEFELIAEGSTTLDEEKTVDMLSMMYTDGKDDLEEIVKICNIENEGIAEKCENAHSHGMCILRELRQRNYKIPQPGK

>MdObp3

MKFAATVIFFAFAYINLAHSKSRQIPQAIQDLQDLLTNTKKDCAKELGFGSSVNDKTLLYEENPTPQEKCLMACILRKVNLMDKNNRLSVDTIARIAGSVSQNNELVISVAVATANNCNNLISTNHPCEAAAQINKCIGGALKANKLKLFY

>MdObp4NTE

SEELTKENAIAVAAACKEEQGASDDDVEALKNHEAPSTHEGKCMAACIMEKFGVLADGKMVKEKAIEVGIALFGDDEAKATAIVEACESLEVDDDHCEAAVQYGACLKEHALAH

>MdObp5

MSKLLSVLFVMGIVAAVVVRGEFDRQAAHEKLKMKAGECKTEVGATDADIEELVGRKPASTMEGKCLRACLMKKFEVMDASGKFVTDVALKHAEKVTDGAADKMKVASEIINACAGIEVSSDHCQAAEDYGKCFKQQASAHGINENYQF

>MdObp6

MAKVFLIVALAVLSLLAATTVVKADLDRNQAMAVLKAKADECKKEVNAKDSDVEELATRNPASTKEGKCLRACLMKKFDVMDENGKFVADVAEKHAAKITNGSADAMKISREIIDACANIEVSSDHCEAAEAYGKCFKDQAAAHGINHDYEF

>MdObp7

MSKLLSVLFVMGIVAAVVVRGEFDRQAAHEKLKMKAGECKTEVGATDADIEELVGRKPASTMEGKCLRACLMKKFEVMDASGKFVTDVALKHAEKVTDGAADKMKVASEIINACAGIEVSSDHCQAAEDYGKCFKQQASAHGINENYQF

>MdObp8

MAKLLVVLAVMGIVAAAVVRGEFDKTAAREKLKTKAAECKTEVGATDADIEELVGKKPASTMEGKCLRACLMKKFEVMNDSGKFVSDVALKHAEKVTNGAADKMKVATEIINACAGIEVSSDHCQAAEDYGKCFKQQANAHGIDESYEY

>MdObp9

MAKRLLTLTVMCIVGAVIVRGEFDKNEAIAKFISKAEQCKTEVGATDADIGEMVGRKPASTMEGKCMRACLMKKFEVMDDSGKFVADVALKHAEKVTEGAADKMQVASEIINACAGIEVSSDHCQAAEDYGKCFKHEANAHGIDENYQF

>MdObp10

MAKFWMSLAVMCAIGAVVVQGGFDKKEAIAKFMTKASDCKTNVGAADVDMEELIERKPASTMEGKCLRACLMKKFEVMNDSGKFVADVALKHVEKVTDGAVDKMQVASEIINACADIEVSSDHCQAAEDYGKCFKQQANAHGINENYQF

>MdObp11

MTKLVATLAVVCIVGAVVVQGEFDKKEAIAKFMTKANECKTEVGATDADMEEMHQWKSSSTMEGKCLRACLMKKYQVMDDSGKFVADVAMKHAEKATDGAADKMKVAAEIVNACAGIEVSSELCQAAEDYDKCFIQQAKDHGIDENYLF

>MdObp12

MAKFLVVLAVVCIVGAVAVRGEFDKKEAQAKLKARAGECKTEVGATEADIKELMEMKPASTKEGKCLRACLMQKYEVMDASGKFVTSVALKHAEKATNGSADKMKLALEIINACASTQVSSDLCQAAEDYGKCFKQQATAHGIDDNYQF

>MdObp13

MAKYLFALSVLCIFGIAASLEKQETEDDLMSKMETCKTEAGATDADLKAIVAQNSSSTAEGKCLRSCLMKKYEMMTVNGTFVPDIALKYAERYADGDAEKLKKAKEIVKSCARIKVSPDHCQAAEQYSKCLMKKAADRGLTQFKL

>MdObp14

MAKYLFTLTVLCIFGAVIVRGAIDKSAVIADFMSKGEACKAEVGANDADLGEIIGKKPASTPEGKCLRACIMKKYEVIDANGKFAPAVALKHAQMYTEGAEDKMKIAQEIIDSCAKLSVSDDHCEAAEEYCKCLHEQAMAHGVEDMDI

>MdObp15

MKVTAVLLFALFAVATAEYKLRTQDDLMKARKECMEAKKVTPEMIEKYKKFEFPDDEITRCYIQCIFEKFELFDAKDGFKNDNLVAQLGQGKENKDEVKADVEKCADKNEQKSDSCSWAFRGFKCFITKNMPLVMDSLKKN

>MdObp16

MQIQFSKYCLSLILLSYLQLSQSTILEEAVIEYIQSLVQICGNESGLSEQDIHLIASDQVDDLYRAPISDNFKCFLHCFYLKLNLFDENGQPIVSEYFKEYIGDHFSVSEDKAAAAMEKCAAIRDENKCENVIKVELCIMDVVNYKY

>MdObp17

MKAVIGLFALLATLMALVELTQAMDKKELEEKVKKLGAECAKEVGISDDEMKLFIANQSKAIDERKFTDKMKCYMLCWYKKIGIFDADGKPKIAEIIKFFEERYHSKKDKVKPALNKCASIKEDNMCEHVFKFERCVAKAIEG

>MdObp18

MKVLIYQIGLLAIILATVELTQAMDSKELEEKAKKIGAECVKESGISGDESNLIMADDLEKIDEKKFTDKMKCYMLCFYKKLGIVNADGKPNVAPLIAFMEERYDHNKAKVKPAITKCGSIKDANQCEQVFKFERCIAQAIEG

>MdObp19

MKTYNFLSGLLLLGLYLGWQHTTEATVEPEIRAVVKFSVLTCAHDTNVPPQQAEHFMPEKSSLMENYTHDMKCFLLCFYRKMDLITYDDHPNHEAFASFMEKRFVSNKDRIKPALAKCLDIDDKDPCEEVYKFELCMLKNVQG

>MdObp20

MKSANFLTGILVMVVFVGHLHISEALTDEEAEFVIQHAIVQCANLTKVNLQEAVHFLPINTKLMDNFSHDMKCYLLCFYRKINLIDFKDHPKHDDFALFMESRFEENKAKVQPALKKCLAIQHKDPCEEIYEFELCMVKNVQG

>MdObp21

MSAKTTFSNTKLLLIIGMVVVIINCWKIKPVGIDAQDPEERVKAIRKKCIAANKLTDDQVKLIMDHDLFTPTTTAANTPKNLQCYCLCYLHEANIFQNNKPNEKFLREVLPVMINDKTKAEKILEKCKKLEGKDDCEIGFNYELCLIKESGLYMY

>MdObp22

MKTFITLAVVCLIASVLATPVELNEDQKAKAKVHFEECIKQENITEEEATKLRNKDFANPSHNLKCFGTCFFEKVGTLKDSVIQEDVVLKTLGSIIGEEKTKKALDKCRDIKGEDRCDTGFQLYQCFEAAKAEMVEA

>MdObp23

MKAFITLAVVCLVACALANPLELSEEQIVKARQHIEECAKQENVPEEDVVKFRNKDVENPSKAFKCLGTCFFERAGTLKNDELQDDVVIAKLGGLVGEEKAKEVLEKCKGIKAEDRCETGYKIFQCFHAAKAAY

>MdObp24

MKAFATLAVIVCLAALATSLELTDEQKAKAKVHIEECAKQENVPEEDVVKFRNKDIENPSKAFKCLGTCFFERAGTLKNDELQDDVVIAKLGSLIGEEKAKSILEKCKGIKAEDRCETGYKTFQCFHAANAAY

>MdObp25PSE

LKAFITLAVVCLVASALASPLELSEEQKVKAREHIEECAKQENVPEEDVLKFRNKDVENPSKAFKCLGTCFIERAGTLKNDELQDDVVIAKLGGLVGEEKAKAVLEKCKGIKAEDRCETGYKIFQCFHAANAAY

>MdObp26

MKAFITLAVVCLVASALANPLELSEEQKVKAREHIEECAKQENVPEEDVVKFRNKDVENPSKAFKCLGTCFFERAGTLKNDELQDDVVIAKLGGLVGEEKAKAVLEKCKGIKAEDRCETGYKIFQCFHAAKAAY

>MdObp27

MKFAVAFVALIVCGIAYGQQHLNLTEEQKLKALKYSAECLETEKSTTDAAKALIKGQFEGLDKNAKCFGNCFLEKAGFLVDGVVQPAVLSEKLGPNVGQDKLDVIMSKCNSLKGSDNCETAFVLYQCYYREHAAFF

>MdObp28

MKSILVTFLVIYSANLITGLVPLKIPDDQKARAAGIASDCIAQEKITTEQAVEFSKGEFSKANKNVKCYANCFLTKAGVLVDGVLQTSVVMEKMAPSVGEAKLKAIMEKCGKVKGSDQCETAFMMFECYHKEHADIA

>MdObp29

MKILAFAVFVIFLHSPFINGDKTFTIPADKRAALEAIIDQCREQVNLSPEMLNKIRHCKHGNVDVEENVKCFYECTLSKVGFFIDGVIQPTKIAKVLGPIIGMDKLNDIMAKCNNLTTGGSICDTVFNKYDCYCKNRVEVD

>MdObp30

MYNQSITIKTVISNFGILADMDDILASMRACHETHPTSVAEVEKFINDKNAEFGDVFKCHVKCVLEKENAFKNDKFDDQAFVKLSLEIPELKNRQADIQKAAEECKNEKGANECETAYKADLNSFKASVEHCLKEFPITEGEMRRFIEEKDVQFGETFKCHMKCVLEKEHIFQNGTLVVDGFIKHSLEMATLKGREDELQKIADECKVENGVNDCDTAFKLGKCLFAHHTIFVH

>MdObp31

MKIFYVCICFAFLAVTFVQANLNEELEKHAEICTEQSKVTPEELEKFFANGMQAQDATDPVKCHFKCIMEQNQFFADGNLESEAILKYLEAKESMKDHLDDVAAAIAACNNMKVEHDCDGAFKLIECFGYTDAGKMAFVA

>MdObp32

MKFLYSALVCLAFFADIIIADLETYAASVEACKKLFPVSEDEIKSFYENKTVQFSNDFKCHTKCILEKEHIFKNGKMDADSFLKHALQMPSLKNHQAEVLKTLAECKNIKGSNECDTAFKLGKCLFVDHTTFVH

>MdObp33

MMKFLYIGLVYMAFAIGTIRADKKSFIASIDACSKQYPVTEQEMKQFVEDKTMQFSESLKCYVKCVLEKEHILKNGMLDTEVFVKGALQIPSLKNHEDEIRKTAEKCKNVKGVNDCDTAFKLAKCGYAYHNLFVH

>MdObp34

MKFQYFSLFLYLVITIAVVKADKKSFTAAIEACSKDHPITEAEQRQFFEDKNAEFSDTFKCHMKCVLEKEHIFKNGTLDEEAFIKHSQENPALNTHENDIHKTIEECKTVKGANECDTIFKIADLDSLSLAMKDCLLKHSITQKEMDMFLENMYANVTENFKCSMKCVLEKEGIMKNGTFDDKTFEKKALSVSLLKGQEKQVIQAAEKCKNIKGSNDCDTAYKIVLCL

>MdObp35

MILYFSTLLIALILSWVNLSSADCIKETGLQERDVPKNFEALPNATETYKCFVKCLMEEAGILQNGEFRLDKAAEEWKQDSVYKTNLPKMLEIGNSCKLLKGENDCKQAFNINVCILQKAAEVFPVVKDEFNLE

>MdObp36

MKLLHFVLFYILYDITGAKANMDAILNAMKQCNDKFPVSQEEMEKLMHEVHDDVSDNFKCHIKCIMELEETFENGTFVDENFVKEVMEVPLLKDHQADIKTATDECKKQHGLNDCDTAYEIAMCIYGRLPEELATGLLSML

>MdObp37

MMLFYWTLCVLIFFSWVRLQIFLRHYIFSHPCHAMPNKCLKEFPFSMEDAPTTLEEYVNAKEDFQCYIKCTMEEINTFSNGEYRLENAKKRWENNPVTKNHIPQMEEIAKECAILKGTNECETAHLINICLLKNFIKAIPDLQRVYGIH

>MdObp38

MIVYGLVAAIEASGEEEFKNIKAACEQEHPLDSDEVIDFGEDPANNVNDHVKCFLECLFKKQNILKNGIVDVKALIKSLEIYPSFKSRNHQVLQAVDNCHTERGPNDCETAYKLMMCLKNHAADVYGNE

>MdObp39

MKVFNIVFAVVAVAALLIAECHSSKDPAKHATCLQENNLSEEEFYGILKEAKNGSNDIDSRMKCYTHCMLEASKHLDENGKLNLNSLQDEENVTEDDIKIAEECKKEFENVEEKCEYSYQVSICVAKAMAAKNAAVKALLEGESAHMNEEGEE

>MdObp40NTE

ISADEISQSAACLQENNLTKNELLEILDSIRAGAKEVDSRVKCHTHCLMKSFGHLDENGKFDPQSIGDGTDLSDIGMADLEKCYEEYQASDDKCEYAYCVITTMENVE

>MdObp41

MYRQCTVLVLALLIFVGKISTEDTSKHTACLEENQMSEDELYNILDEIKAGATEIDRRFKCYTFCMMQSWEHLDENGILDMSTLKHHSNMTESEVEPLEKCTEEYRGSDDKCEYGYCVIAALGNMD

>MdObp42

MNFFNIALCVALAVVFVVGKTSADHAACLDKNGLSQDEFDSIVKKLEDGAEDADTKFKCYTHCMMESDGLIDGSGKFDVSSLDDGEDKDEAEKCKKEYDGVSDKCEYAFKLSNCYFKHE

>MdObp43

MNFPQIVLGIAFIIVSVVEKISADDADDLRHAICLKESEIGEDEIDDLMDSLYDDATAVDERFKCYAHCMLERWGHFGEDGKLDVETFNDQNMTDQDMAAVEKCKSEKDNIEDKCEYAFEVTACFMEAFTSSLVEDE

>MdObp44

MHFLKIVFIIVTAAALTKAKTFAEVTHNKCRRMYGLSDNETTTMTNLLATIPNDIDVRYKCYMHCIMIGWGHLDEDGRFRIEWIKEDQHLSEDHLKVLENCIERHNGIDDQCEYVFTTTICAMEGYKDLE

>MdObp45

MQLLKGALFIAICAVLATGEPLPDRSMIHAECLEKHELTENEFQEMAEKMSLDIDNRFKCYMHCMMSGYGHLNESGKIVIEKIQEQQYLPERHVEIFTECGEQHEAVEDQCEYVFTLSTCVMAQIRKEAEERMG

>MdObp46

MKFYLCLSICAVVLMGGALAEYEEYKEMATKCMEQNNITEDEFEAIPKGEDFDPETLDERFKCFTHCMVEDMGYLDETGKLDLSKLEQDERVTQEHMDAAIKCKAENEFIDEPCEYSFKMMTCALDAMM

>MdObp47

MKAFTVALIALIMISYIIQNEGFEVPEHFKKHAKKLHKRCQNQTNTSDDVIRAGFSGTLPQDDNFACYIHCIFDMIGVIDEKNVMRLESLTQVLPEELHPMITTLVESCGTKDGDDKCKVAYNTLKCYVDVNPIMLSDKLHFILD

>MdObp48

MFIFFILIKLCLLHLTWIPSINSVTMEQFEQSLDMMRNGCAPKFKNSIETLDALRFGRFEQIDESSTDIKCYAKCIAQLAGTLTKKGDFSIPKATAQIPIILPKEIQDSARDALNSCKEVQKDYKDSCDKVFFTTKCVYNFKPEVYKFP

>MdObp49

MEKRFLIVLPVLILMPFLVSAQKPRRDENYPPPEFLKRFIIIHDVCVEKTGATEEAIKEFSDGEIHEDPALKCYMNCLFHEVNMVDDDGELHYEKLKRVLPDELTQFVQHIIDACESHVPQGSNQCERAWSWHVCFKQTDPVHYFLP

>MdObp50

MRAMAVLYGILLVAIIFMVGAQSQTVPRRDETYPPPELLAKLRPVHDTCVGKTGVTEEAIKKFSDEEIHEDELLKCYMYCVFDEMDVLHDDGEVHLEKVLDLMPDSMHDLAINMGKRCLYPKGDTTCDRAFWLHSCWKKADPVHYFLV

>MdObp51

MSFAGIWRSGRTQLLCTILIVVSLLSCGCQAQQPRRDAEYPPPAILKMAKPFHDTCVEKTGVTDAAIKEFSDGEIHEDEALKCYMNCLFHEFDVVDDNGDVHLEKLFAAIPGSLRELIVNASQNCVHPVGDTLCHKAWWFHQCWKKADPVHYFLV

>MdObp52

MKFQLVCLLVCGLALQAFAAAKFEPRTPEDALKAHEECREEYNVPDEIYEQYLQYNFPDHKRTKCYIKCWVEKMGIFTEKKGFDEKAIYKQYTRNNTQYLSSVQHGLEKCIDHNEWESDVCTFAHRVFSCWLPINRHVVRAVLGTQKDN

>MdObp53

MKTCQSVLSIALFILLCQHLVAADINKHEGYVLGKCLERYGGPSYENAERLKRFKDWSIDYEELPCFTNCYLANMYDFYNETDGFSEQKVIDKFGASVYEVCKPKFSEGKDKCETAYKGFHCLVNLENDPFVVIDGMDNIDMDAKLAMKDCLHRFDRSEWQLFGEYSRFPVKEPIPCYSRCFLDKLQLFNHRLHKWDIRGLNTKLNISVENANTSACEAMAVKRNRNICAWMYREFTCYAMASIAKEELKK

>MdObp54

MKYYSVLFTVATILIAQALCNLEHDMNSDILRQCLQDISHHNETVTERLLEKFNTYANWTKEEIPCFARCVAAEKGWFDIERHRWNKQKIVDDLGANMYNYCRYEFNRPFSNVCTYAFKGLKCLKQAELNVIVTYSHLVTCVKEKATSMSQLLEYYHFPAGERIPCLFNCFANKAQLYDDNYQWIVKNWLKAFGPIRDESANISICRISDEKRRTMNVCSWMYDEYNCWERLNYNTNGSVAYRRALRKISNSNSIDHNN

>MdObp55

MSSLNHSQFKRHTSMKYCICIISLESIVSSTPSHLDANIIDFDRVIATCNSSFSIPMDHYRTFNTTAELPDVVDKTGMCFLRCLYEKSGLLENWKLNTTKIRLNIWPATGDSIEVCEMEGANEKNPCVRAYDIAKCLTIRALVDARNQPL

>MdObp56

MKLFVVLCTLFVLNASAYVVKSRDDLLQFRNECVSELEIPENLVEQYKKWQYPNDSVTQCYLKCVFVKFGFFDTASGFNVENIHQQLVGSQGEANHDDAVHATIESCVDNNEQGSNACEWAYRGATCFIKNNLQLVQRSVAPSA

>MdObp57

MKIFVAVCFLFAVSTSAYVVKSRDEHLQFRNECIAELKVPTDLLNQYKQFQYPNDSTTQCYLKCIFVKFGFFDTTNGFSVENIHQQLVGAAAEANHGDDLHTKISSCIDKNEQGSNACEWVYRGATCLIKNNLPLVQRSVATQT

>MdObp58

MKSRTFVALLLCNILILVTGQNTISDNFYDKSEKCFDQLHVPQRYKATFQAFRYPDEEIVHKYVHCLAMKLEIWTNRSGFNIEKIYNQYRNRVNDEIMLPTISNCNRSAQNSNKELWCYRAFLCILNTDVGKWFKEDVQRSRQANNVPNGHH

>MdObp59

MKVFIAILCLTAAVTVSAHHEEGHTGHDHHIIHDGHDYTVKTKEDLARFRDECGKQLDVPADKMEKYKAWEYPNDEITRCYMKCVFEKFGFFDETHGFNPYLVHHQLAGGHEPVDHSDEIHQKIDLCADKNSQKSDACTWAYRGGMCFLANHLKLVQDSIHSH

>MdObp60

MKLFLALLAIVACVSADDWTPKTADEIKTIRAACLEEVPLTEEQMNHMKSFDFPNEEAVRKYLMCTSVKMDIFCTHQGWHPDRIAKQFKMDMEESDVKKLADDCVAKYPKADKENDVHVYEVHKCLMDSEVGQKVKTYIKKRQEQLSKQA

>MdObp61CTE

MNKIFGVIILEALADDPHDWYPKNPVAVHEKCREENPLTEESRNDLEKGIIHAHPDLIAFFLCTAKSMNFYTTQNGFDANRLIYALEKMDLLHNRNAVEECVKKNKDVSPEETKVFNVAKCIED

>MdObp62

MNKIICIFIALILTKALADDDHDWYPKDPAAAQHKCNDVLSAETKFNLMKGVIHNSPEVSGLFMCTAKALNIYTSENGFDTARLIYALEKMNRLHNRSAVEECVRRNLDVKPEGTKVFNVAKCVEDENVLVEKVKYGVERKIIEKF

>MdObp63

MFKLILLSFVCLHLMQVYAGQNDWYPTNAYSILQQCKEEHKLPEAVIDDIDHGRIEDSPTFRQLVLCASKGFNVYTSENGYNADRLAYALYRIGMNRTCRRQLVGQCVTKYKDIKPEDEMVFHIIKCILEKEVSPEVVEKDGPPSEWKGCDINA

>MdObp64

MIFPNQHRLQVQRLLEATMHKILIVLANRPDWYPENPTDIEKDCMQQYPISAEAKADIRNFKLTDAPNMKSLLLCVANGQNVYSPDEDLEPERMAYSLYRSLHLECELDLVRECLGNHKEHSVNGNHEDFMYLTLECIFEGAPGKCTNTE

>MdObp65

MNKLSIVLIISCFAVIFAERPDWYPKDELAVEAKCREENSISPELMTKIWSSRIEDTPQVRKYVMCLGHNKNFYNSEIGFKADRLLVIMKERANMDCKPGFVEGCAEEGKDIEPEDAMLFKIIKCVIVGGEENCKKAE

>MdObp66

MTKFCCVVLICCLAMVSAELPDWYPQDEPAIEAKCRDENSISSDTMTKIWSHQIDDTPEIRKFLLCLAENKNVFNSDMGFKADRLQIIMKERAKMDCKLEFIEECEMGAKDMKPDDAMIFNIMKCIVGGIKENCKKIE

>MdObp67

MNKSFFILIGIIFTQVLANEHDWYPKDPGAIQDQCAESNPLTDESKADLLLGLVHYHPDLIAYIICTAKGMNFYTTEKGFDTERLLYALDKMNRLHNRNMVVDCVNKYKEIKSEYEMVYHVAKCLKEGNNADGDVKNERPT

>MdObp68

MNKIFGVIILEALADDPHDWYPKNPVAVHEKCREENPLTEESRNDLEKGIIHAHPDLIAFFLCTAKSMNFYTTQNGFDANRLIYALEKMDLLHNRNAVEECVKKNKDVSPEETKVFNVAKCIEDENVSGEKH

>MdObp69

MEMQFKRGFSFLAIPVDLNSNSEKTIKMNTLSCVLILICCSAMIFAERPDWYPQDIPAIEIKCREENSIKTDIMAKEWSNQIEDTPKLRKLMLCLARKKNIFNSEMGFKADRFQIILKDRKKVDCKLEFMEECVNGAKDIKPDDVMIMNIMKCFVPGMEENCKKIE

>MdObp70

MNKFCFVVLICCLAMVSAELPDWYPQDEPAIEAKCRDENSITSDTMTKIWSHQIDDTPEIRKFLLCLAENKNVFNSDMGFKADRLQIIMKERAKMDCKLEFVEGCEMGAKDIKPDDAMIFNIMKCIVDGLKENCKKIE

>MdObp71

MFKIIITICLFSLVFAERPDWYPENPQEIEAECMKKYNVDAETIAKIRAFQLEDTPTVRSVLFCSAVGKNVYRPESGFDPERFAVGLKYGLNVDCNVDFIRNCANKYNNIESQEGKYFHFFKCVFDDIKGNCKKIE

>MdObp72

MFKILSIALLCVTAIFVQELPWNPANSNEIEAKCREQYPLADEMIANENGHLKVKHNPTFRSYLFCTAMGKNLYSPEVGFIAERLAYEIQNTYKYNCPLNLIQDCIDNSYEDSYSEDIIYFNIMKCILENAFEECERV

>MdObp73PSE

MFKIIIXIFFISLVFAKRPDWYPENPLEIEAECMKKYNVNAETIAKNRSFQLEDTPIVRSLVFCIAVGKNVYRPESGYDPERLALGLKYGLNIDCNVDFLSNCAHRYNDVESQEGKFFDFFKCVFGGIEGNCEKNQ

>MdObp74

MKNCAVLLVFCFGMIAICQVYAEILDLGKTPKWYPRDGPEIEAECMEDHSTSAATIAEIKKFEIKNTPEVRAYLLCFLTETNVYRPAKGPEIKRIAWSLKESFNLNKCDLDMIRDCVEEHQSDELKDYAYFKIIKCAYEKAPARCLQKIEK

>MdObp75

MNKLSFVFLICAIAMISADRPDWYPEDEAAVEAKCREENNVSAETVTKTWANEVEDTPELRKFLLCLSENKHLYHADTGFKADRLQYVLKEKSKLNCKDDFVEGCVNAAKDVKPDEALVFDVTKCVVAGAKEHCENVE

>MdObp76

MDKFIFIVIMVCIKETLQQADSSNALLEMVKMSVEDCYEDDEKTKKIEISDDGFQDIVKGSRDAVRNAKCIRYCIMKKHELFSDDNSLDETAVIPFFTYLFNNAIEIHHLKGIIASCNEAITGEADRCERSHKATMCILEKFNAAGLKNI

>MdObp77

MKVLIILVCGLAVSCGFNYNCLGEYFHTVYEECLFEHGGDTAFIANWQEFKPTDNENEKCFRSCTMRKCGVLNREGTINDDVSVGLAHILSGGDVDKVAAIHQAVQACRGLMNYEKNVCHNGENWSRCIIGHCKHCGLVLNV

>MdObp78

MRTIVAILAICSICCGFDVRCLDKYLDTAFEECTFEHGGNKAFTTNWSEFRKTIDPNEKCFRACVQRKCDFWDEEAKIKEDVPLGLAIMLSGGNRSKVPSIEKAARACRKLMEYGDNLCENGENWSRCVIEQCKRCGLVLKFE

>MdObp79NTE

ALITDWIAHKNAEDEKSKCFRTCAMKNCGWFDSNGKLKKEVPERSAYALYGGDASKIPQIMEAGKKCLDTIQYDEKNMCNSGENFSRCIMGNCKKCSLNLSAAL

>MdObp80

MKIIGILLLVIVGCYGLIDKCPGSDLKKVFGDCLKEYGGDKALLADWIAHKDAQDEKGKCFRTCTMRDCGWFDENGKLTEEVPLRAAYVLYGGDESKIPRILEAGKKCLNSIKYDEKDICNTGENWSRCILGTCKDCGLDLAASI

>MdObp81

MSLFSIMNNIVMAEWCRGDYFRKAAIRCAAAHGTSEVDFQDYLHFRPAKSEAAKCLKACIFDECKLFNADHTFSLDLPRRAAYVSSHGNWKVFKVMEQVGNYCVQHVRTGENTCESAEALLKCYAANLPFPVSLEGALQ

>MdObp82

MKTSIGLLLIYLICNVNSSGALQPNDWCSGEFLQNSLRRCGEVHGATLADLNDFRYLKPARNARMKCFRACAYIACKAYNVDGSFVANAAETTAFTFTRKNPHLWGPTLNAANFCLKTLPEITYQYAYRSYTVCDKTEDFIQCVRANLPHKSSYEGLF

>MdObp83INT

MKTIIGFFVIYFICNAVVTSGALKPADYYDLRYLKPARNYPIKCYRACAFIDCKAFNADGSFVANAGENLAFSMSRKNPHIWNQAFDVANFCIKTLPEITFEHAQKSYNVCDKTEDFLQCVRANLPQGSSFDGLF

>MdObp84

MKVFWILIFLAAADCDEIVKPNTRCSGEFLQNAIKRCAQAYGATEDDLKDVIYFKPAANHKMKCFRACVFTECKAFTNDGSLVANIPQTTAFLTSRRNASHYQIVEEIGEDCLNKLSSYDDTCELAEQYMQCIGNNTPDDVNLEGSY

>MdObp85

MQLFLLIFLAIVSVFGEELKPNSWCSDSYLENAVHKCLEEYDGILADVYDFIYLRPAANENMKCFRACVLNECYSFNGDSTFVENIPATTAFWASRRNAFHQPLVERAAKQCVTSTTDAATICDLTDAFITCLGENTSHDITFRGAFNLD

>MdObp86

MNLFCLFVIYALIGINEANDWCYGPYLKQEMDACVATYGATQADLFDLLYLNPARNFQMKCFRACAFNACRGFNLDGSFAEHVPYTLAFSVSRINAERGIAVREAAKYCIKALRSISFGHLRRGSNVCEDSDYLLQCLGMNTPPGTNFVGAF

>MdObp87NTE

GVYDGLHLIPARNFQMKCYRACTLNACRAFNIDGTFAAHAPYTMAYGLTRLHAEHWATIRDVTKYCIKVLFSFPLAYKASNICENTEQLLQCIRLNVPSGTSFVGAF

**“Plus-C” OBPs in *M. domestica***

>557755950

MNSVLSGVVKYLPVLAAVLFEIITTADAAATNGMMGAFNCSQPPKFDNFDISKCCRLPNINLGSVVDKCHKHVKSLKSHNANYPAYAHVCYPECIYRETGSFIDGDIQMETVRNFLQNNIEQRDKIIVPTIVKSFETCMTNIKNTMQARGIKSYPKIDGLGCSPYASMVYGCVNAETFLHCPPEMWQEESSCNVAKNFALQCNPLPHVPLPMI

>557760596

MNKLSIRFYLIFAFSNLCWLPLSGGQNCEDNSIITQELQDFLSCCSGRPLYTSEICIDKMIGKNKFSPNCLIDCMYREYQIYDDDVETIDLEAAKNLLNEQIVNEEFNPVYGQAFERCSKFEKSALLEVFAFVNITNQNACDDYPMFMDSCVWAYTVANCPESHALQSAECRQKTEWVNKCLFKE

>557774545

MLSHKNSVISFVVILSCCHQLVLSAVIDCQRPPQLVDPATCCKDGGRDDVTEKCALRMGITGQPTDPQPSVATATCLAQCILTESKYMNTADSIDLTAIRTDLQTKFSNDSEYANVMFEAFRKCQPNTERKLQAFKQLPMGRSILQRGCSPFAGMLLGCTYMEYFKNCPAHRWTESAECSLAKQFVTQCSLGA

>557750291

MDTKTIHLLAGVVLLSVLSHVTAEVDCNKAPAFVDPKECCAVPNLISEELVEKCKGNEPPPPPPSGEMNNEVDESEQGGPGRHHHHRHGPHGHHGRHGHHHHHCFPTCLFNETGILIDGELQEDNLDTFLSGAAAENPEVLPILKESFQTCYQKSVEIMEKIREHWSKNENSSRRPPHHHHRHHHCSPQAGIMFHCAMMNTFKQCPDSIWSDTDECNNVREYFTECMPSPDDQDDDEEEEE

>557750289

MTHFKLPNGKLLGALALLMAFVLETTFAAGIDCSMRPPMIDPLTCCPVPDIISEDIMTKCRMAMPRPPPPPPGYPYADPGLMYSDEDSSSMSNESKQPKTTGPPNRRPPHPHYGPPPPHMQACFLYCALNETGILPATPDAKLNENKLSTYLKEILANATDMIPIMESSFKTCAVKVEEMSKKFKEHFEKKAAASASSSNESKTQDRMMRPPPPLCPHAASHLMGCVFKESFINCPSSLWSNTEQCNEIRDHMKNCKANKMKNGKLDKM

>557771950

MFKIIAVLSLALLAVNAYDFSDTYFNQYLFQEYESLNSNLLSRHRRDVSEVAKDEKKSADEMKPMEEMKQMDEMKPSKECDGQFHHMMMMKKDLTCCESNKHDPSYFSMIRETKKQCAMKLRTNNPDVENFDPFNCEYMGKIKDLIVCESECVAKTLDLLDENGEIKRDAVVASFKKSMSSDSEVQHNVLEGYVDKCLAKMKGKDLKPAGKCSSAPMELHHCMFGEMVSGCPAESQVNTPRCQKIRERYSKGQTLAFGKHVLHEFLHSGRGRHHGHKDQM

**The Odorant Receptor (OR) family**

The odorant receptor (OR) family of seven-transmembrane proteins in insects mediates most of insect olfaction (e.g. [16, 17]), with additional contributions from a subset of the distantly related gustatory receptor (GR) family, for example, the carbon dioxide receptors in flies ([18-20]), and a subset of the unrelated ionotropic receptors (IRs) [21-23]. In *D. melanogaster* the family consists of 60 genes encoding 62 proteins through alternative splicing of some genes [24].

The MdOr gene numbering starts with MdOr1 as the ortholog of DpORN, a gene that was lost from *D. melanogaster* [25], to avoid any assumptions of orthology based purely on the naming numbers, and then roughly follows the *D. melanogaster* cytologically-named genes in order. The MdOr gene set consists of 84 models, as well as the OrCo gene, compared to 59 in *D. melanogaster*. Only the last of these was built as an alternatively spliced gene encoding two proteins differing in their long first exons (MdOr84A/B), like two of the DmOrs (46aA/B and 69aA/B), although even this model is questionable as there is a large gap between them that might contain the C-terminal exons for MdOr84A. There are 7 apparent pseudogenes (8%), while another 8 genes are missing parts and could be pseudogenes. The result is 78 apparently intact OR proteins. Approximately 12 gene fragments remain so short and incomplete they were not included, but some might represent intact genes.

The automated gene modeling had access to all available insect ORs in GenBank for comparative information. The REFSEQ set used as the official gene set succeeded in building at least partial gene models for all but 2 of the 78 intact genes. Unlike many other insect genome projects, more than half of these (44) were precisely correct, presumably because of the relatively close relationship of *M. domestica* and *Drosophila*. All others required at least one change, while 2 new gene models were generated (not including pseudogenes or those requiring joining across scaffolds) (Supplementary Table 6).

As expected, there is a single conserved ortholog of the DmOr83b protein, now called OrCo [26], sharing 87% amino acid identity. These were declared the out-group to root the tree (bottom of Supplementary Figure 5). There are 14 instances of simple 1:1 orthologous relationships, such as the relationship of MdOr1JOI and DpOrN near the base of the tree (Supplementary Figure 5), sharing 44% amino acid identity, which allowed for confident building of the MdOr1 model across two scaffolds. These simple orthologous genes nevertheless are sometimes extremely divergent, for example, the pair of MdOr79 and DmOr88a at the top of Supplementary Figure 5, which share only 25% amino acid identity, yet are best reciprocal BLAST matches and cluster together confidently in the tree, appear to be orthologous, although they do not share microsyntenic neighbors in the two genomes, so might conceivably have lost each ortholog from each species and hence be inappropriate comparisons of paralogs.

Most of the remaining relationships are more complicated, ranging from clear examples of gene duplication in one or both species lineages, to large expansions in one species, to apparent gene losses, all examples of the birth-and-death mode of evolution of these large ecologically-relevant gene families. For example, DmOr1a was duplicated as MdOr2/3 (Supplementary Figure 5), while DmOr94a/b are duplicates of MdOr80 (top Supplementary Figure 5). More complicated relationships where orthology is less clear are exemplified by the set of DmOr85b-d and MdOr71-75 (top Supplementary Figure 5).

There are several large species-specific expansions that are likely to reflect major changes in the chemosensory ability of each fly. The most prominent of these are nine DmOrs related to MdOr22, the expansion of MdOr24-33 related to DmOr45a, and the expansion of MdOr53-64 related to DmOr67d. In each case, while some of these duplicated genes are in tandem arrays, there appears to have been considerable gene movement in each species since these expansions, indicating that they are quite old events. For example, despite being in four scaffolds, MdOr24-30 have the potential to be in a single tandem array, but they appear to have moved from the remaining three tandemly arrayed genes (MdOr31-33), which are in microsynteny with DmOr45a. DmOr45a has been shown to mediate repulsion from aversive chemicals in larvae (Bellmann et al. 2010), so it is possible that the MdOr24-30 proteins also perceive aversive chemicals in the larval environment. The MdOr53-64 genes are of particular interest, as DmOr67d is the receptor for the male-specific pheromone 11-cis-vaccenyl acetate [27], suggesting that the elaboration of related receptors might be involved in pheromone sensing in *M. domestica*.

Finally, the existence of highly divergent genes and lineages in one species with no clear orthologous relative in the other implies that several genes and lineages have been lost from each species, specifically at least 8 from *M. domestica* and 12 from *Drosophila*. The combination of these losses and the extra gene duplications in the *M. domestica* lineage leads to the relatively larger size of the OR family in *M. domestica*.

**87 MdOr proteins in FASTA format**

>MdOrCo

MQANLQPTKYTGLVADLMPNIKLMKYSGLFMHAFTGGSALLKNVYSSIHLVLIVLQFIFILVNMALNADEVNELSGNTITALFFTHCITKFVYIAVNQKNFYRTLNIWNQPNSHPLFAESDARYHSIALAKMRKLFFLVMLTTVASAVAWITITFFGESVKFATDKETNSTITVPIPRLPIKSFYPWDASSGMFYMISFGYQAYYLLFSMVHSNLCDVLFCSWLIFACEQLQHLKGIMKPLMELSASLDTYRPNSAALFRSLSANSKSELIQNEEKEPVNDLDMSGIYSTKADWGAQFRAPSTLQTFNGINGGNPNGLTKKQEMMVRSAIKYWVERHKHVVRLVAAIGDTYGAALLLHMLTSTIKLTLLAYQATKITGVNVYAFTVIGYLGYALAQVFHFCIFGNRLIEESSSVMEAAYSCHWYDGSEEAKTFVQIVCQQCQKAMSISGAKFFTVSLDLFASVLGAVVTYFMVLVQLK

>MdOr1JOI

MKDKFKTFMRDFFPSNVEKGEIGSVKLNIWLAQITGVPIIGLKDESSLIKNLILLYGIFTTTVVTFIYTGFEMYDLYMNWHDLDSLTQNTCLSLTHVSGAIKTVNIIFHLPRLEGVIRKLKHVTKTYIKSEKQLVVFYDGEVENKLVLSIYIGIVGFTGFMGMIMLYMPEAVAGKIFPYRVILPDWMPQQLQLLYMGLSVIIFAIQIIAVDYLNVTIINQIRFQLNILNLAFDDLIVETQANSRETKSLVLYKDDPVKRMDSIVEHHCLLGELRQETEDIFSQPILWQFMTSVIIFAMTGFQATVRSSGSSAAVLIYAYCGCIFCELFVYCWFGNEVSEQSKTLGTSGFHSSWYHFDRRYGKSLLIFLTNAQRPFVFTAGGFMGLSLPSFTGILSKSYSYIALLRQIYGK

>MdOr2

MYNNVDGKTRQDLEFLDVQYRALIRVGLDIGAIRGKDFLNDRGKFLIYGIITTYLQYGLILFAVHIFGVQIDKASAALSMFNQGSLLMLKVSILIFKSNRLLKLIWDMNLLATMANEPERETWLSENRFSKVIGNIYSTACIASVILSISIPIIFMSYEHFKGLEVSLKLPFDGEFPYEHLGIPIFILNYILSVIYVYTLLCWTIGIDTLFGWLIHAVSGHFRILRLKVEMAAKKIDEHGNHLDFVQDIGAIVRYHIKTLGFVDALNEIFGQIFWAEVAFSCLQMCFLIFTLNNGSDKRMIPFNAMVFTAISIQMMIYCFGGEKIKSENEMFCFDIYSKFPWEKMYPSEKRMMLLPLQRSQQDAALRGLFFELDRNLLVYIYRTAFSYNTLLGAMKE

>MdOr3FIX

MSDTERQNLDYLPVQFGAFMVLGLDIGVTRRSALLKSGWTFLFNILCTVFMEYGFANFVINSITDIDAITSSLSMFNQGMLLTFKVLVMVFKGDEMLKLIWDMNRLARGANAKEWEIWISENRMGKWIALGYYYCCYIAATIMAVMPWLFMLYEYVQGRGVHLRLPFQLQFFFVSGNGFHISIFYYIGTLLVVRAWFNMSVGIDTLFGWYIFAVSGHFRILRHKIKETALKIDAYDNHRDFVSDVAAFVSYHNRTLKFTENLNRLYGEILWSEISMSCLQLCFLLYSLTNDENFANIPFHFFASAAITMQLMIYCFGGEKLKNENDMLCHDIYMAMPWEKMYPSEKKLMLLPLLRTQREISLKGLYFVINVNLLVFIFKTAFSFITLLGAMKEI

>MdOr4

MTNALTDNNKNIYSKLDTNVAFEYHWKVWRWTGIKPPQDMNPQLYRLYAIVLNFLATVLFPLSLIANVFFTQNLQQLCENLTITISDCQSNLKFINVFLVRHQLDRIKSILRRLDRRVQDDKEFAVLKSAIATARSSFLIFFRLYSFGTTLSVVKVALAESRSLLFPAWFGVNWDGNLSTYVVVIVYQFFGLAVQALQNVANDSYPPAYLVILSAHMRALEIRVKAVGQFRQEGMQQPLTLSAEEQAKCLKEFNECIKDYLNILKLHSIIQRIISKACLAQFACSALVQCTVGLHFMYVVDAANYEAQLMSIIFFVAVTLEAFVICYFGHMMSLQSSNLTYAFYSCGWLAQSPEFKRNLIITLMRTQRTSTIRAGSYIPVDLPTFVVLMKYAYSVFTLLIRFK

>MdOr5

MALQPMASSSSSASNKIHTWQAFRNHWILWKFCGLHPPKRNSRWFNPYLIYAIVLNVTTTLMFPITLIVDLILSQNLTELCENLYVTITDVICSLKFINIFTVRHKLLEVRWILERLDVRATTPEQRQELRHGIQTSHKWFMAFFRFYTCAVITSQLVVYLSKERVLMYPSWFPWDWKASKRNFLFAHCYQVYTVSVQTVQNLGSDTYPQAYIVVLIAHIRALGLRIKALGEALSATAAGDVSSPSSSSKKLSDDELYRELVNCVKDHQIVHELYLTIQECISKTCLAQFVATGLAQCTIGVYIIYVGSDFSRLLNSFMFFGAITIEILILCYFGDLYCRANDFLIDAIYDCNWIDKDERFKKALLLLLQRSQQADCLKAGNLIPVRLPTFVKIMKTAYSAFTVLNEVN

>MdOr6

MSVLFSPHPNTWEAFKYHWLLWKWCGLQPPSRDSKWFRPYLAYAIIFNLTTILFPLSLVLDLTLSQNLTEIFQNLYVTVTVVFSSLKFVNVFLIRRKLLEVRFLLERLDVRANTEEQQQELKNGIAMAHKCFMIFLRLYVCAITTSQLVVYFSSERVLMYPSWLPWDWRESKRYFLFAICFQIYAVSAQLSQNLGNDTYPQAYIVILIAHIRALALRIKHLGVVSTSVPAPEGKLSQEDFYRELRQCVKDHEHVHELYLTIQECLSTTCLAQFIATGLAQCIIGVYILYVGDDFSRLLNSLVFFGAVTIEILVLCYFGDLYCQANEFLIDAIYATNWMDRDGRFKKALLLVLQRAQVTNCLKAGNLTPVMLPTFVTIMKTAYSVFTVLNKVN

>MdOr7PSE

AVKISKKVATKQALTNLYICFRVVGIHVTKSNPHLYIVYAIVIHSLTTVFTPISFTTSYFRKTDQDFNVGVFLTSIQAVINVYGCAIKILLLIYYKTKLEAAEKLMDKMDQHCRAEDEIQELFNIRDLGRKIVLGYITAYWTYTTMTYISALVSGVPSYSINLFFLDWKRSKREFYLASFLEYVLVTWTCLQQVANDSYGTIYVCILRGHVRVLLLRIRKMGRKVDQTADQNLEELKSCIKDHKDLLELYNIISPVISRTIFLQFSITAVILGITLIXIAKFSFSLYTLIKQMGIKERLGL

>MdOr8

MSKQTVKVIKKVATKQALTYLYGCFRVMGIHFTKSHTHLYLIYVIVIHSLTTVFTPISFTTSYFRKTDENFNMGVFLTSVQAVINVYGCIVKIFFLVYYKKKLEAAEKLMDQMDQHCQADDEIQEIYNIRNLGRRIIIGYGIAYWIYTTMTYISALASGVPSYSLNLFLIDWRRSKLEFYVASFIEYFLTSWTCFQQVANDSYGTIYVCILRGHVRILLLRIRKMGRKVNRTADQNLEELKTCIKDHKELIELYNAISPVISRTIFLQFSITAAILGITLVNIAIFASSITAMAASAFYIVAVSVEIFPLCYYANCLLYDSDTLATEIFHSAWIGQDRRYRKMLIFFIQRTQKSMELWAGKMFAINLNTFISIAKFSFSLYTLIKQMGIKERLGL

>MdOr9

MEIPNITTVLPQQVQEDEQEPSTSSNKTLKSSHANKSDTNDDSSVQTRHGLRFLFIGFRLLGVYFPKRGRFLYFLWSLFVNLYATIYLPTGLVVGIITHRDVAIGDMLTSLQVAIDVVGCAIKIVLMYFLLPQLLQCDPVLERLDKRCTSPEEKDLVRRFISHGNRFVILFGMAYWSYASSTCISAVLFHRLPYNLYNPLLDATASKGSFVLGVFVEMMPMYLACSQQVVDDSYAVIYTQILRTHLKALVFRLQHLNDDHRNENGVISPEAEERNIENLKLCIIDHKNIIELYTRVAPVISITLFVQFTITASLLGVTLINILIFATNTASIVASCFYVLAVVVEIFPLCYYAQCLMNENDHLTEAIFHSNWIHQSKRYRQMLIFFMQRSQKSIEFTAGKLFPITLSSFLSIAKFSFSLYTLIKEMDIKTHYGLD

>MdOr10

MEHPDIGEQPALLPQQIQEEQPQPETKSNEIPKLNHENKWDLKAEPPLETRQGLRYLYNGFRFLGIYFPKRRKGLYLLWSIIVNLYVTIFLPTGFIMGIISVTDENVEIGNLLTSFQVAINVVGCSIKIILMYFLLPQLLKCEPIFERLDGRCTSREEKDLIRQFVHDGNRLVVLFTVAYWSYSSSTCISAVLFGRLPYNIYNPFIDANASRGYFILAVFMEMVPMDIACFQQVVDDSYAVIYTQILRTHLQALLIRLQHLNDDDAADLDDEAQERNVEKLKLCIIDHKSIIELYNRVAPVISITIFVQFTITASLLGSTLINILIFATNTASIVASCFYVLAVVVEVFPLCYYAQCLMDENNRLTEAIFHSNWIYQNKRYRQMLIFFMQRSQKVIEFTAGKLFPITLSSFLSIAKFSFSLYTLIKEMDLKERYGLN

>MdOr11

MFLRFLSRSNPLKEYYFYVPRICLQLMGFWPGSPRSRRILCWAVFNFIILLVGVVTELHAGFSYLNYDLEKGLDTLCPAGTSAVTVLKMILISYYRQDLEAVLKKMHQMLYGCNEKDMEHKAVYNRIIRQSSVMAARVNFAPFLAGFITCTAYNLKPLILVWIFWSKGKDLMWLTPFNMTMPKFLLEGPLYPLAYIFTAYTGYVTIFTFGGSDALYFEYCTHIATLLKMLQTDVKLLFRKFEGKLTLTPTEAAYVEEQLILIIKRHNVIIEMTDFFRKRYSIITLAHFVSASMVIGASIFEMLTYTGFGRFIYLGYTVAALSQLAVYCYGGTLVAENSIYLATVVFKCNWYICDPKLRRIILMIICRSQKSLNMSVPFFSPSMSTFASILQTSGSIIALASSFQ

>MdOr12

MFNPKPNNDLNYRIPGQCIWLKLNGSWPYNHQEANKDFYSSRYVWGWLYTVWSWYVVWSVGITIGFQTAFLINNLGDIMMTTENCCTTFMGALNFVRLLHMRLNQRQFKVVIQQFVEDIWINKKQHPHVAAVCSRNMRTFRIMTVLLSCLISMYCVLPLVVLFFDVGLDADEKPFPYKMLFPFDAHHGWRYIVTYIFTSYAGMCVVTTLFAEDSIFGFFVTYTCGKFQILHERIDNLVFDAYESVANRQNELEIQECYVKLLNRIAYDHNKLIEFAGKLENFFNPILLVNFTISSILICMVGFQLVTGKDMFIGDYVKFIVYISSSLSQLYVLCWNGDSLIQHSLETANHLYTCNWEGGQIRSYMPASKKFRQNLEIMIMCSQRPVKITALKFSTLSLQSFTAILSTSMSYFTLLKTVYDENQEDGPAN

>MdOr13

MGFWYKPNCPFDEKFSFVSDFYVHLIINGCWPTDGDPKSLSYRICNALYTLWSCQVIFSLNFTLYAECMYVYENSADLGKVVGNMCLIMIALMVSLRLLYFRGDISRMKRLLTMFAEKIWIDSEAHPKAYERAVRRTKPTFYISLSLWICLVLYLLFPIIFNLTQGKSPDSNDKPLPFPTVFPYDTQTHWAYIFTYIFLSYAGYIAVSLFYAMDAILAYFISFVAGQFEILHADIARLIPECHAEWLRRYGAGAAENGVKLNYLQEMYAKRLHGIAKRHKDIIAFCKELEKFMSFPLFANYGTSTFLICFVGFQFMIAGLKSFGDFMRFFMFFMAVTGQLFIVCKLGNLLITQSTDTAHYLFACNWEGGYLSKNSPLLLYPDIMELQELNRNLPLWKDLSYIPANRNFKLKLMFMIMRSNRPVQLSVMQFTVLSLQSFNKVVSNSLSYFALLKSFLDK

>MdOr14

MAILYKPRCGEDVNFVLPLKVRTFLMINGCWPMEDNANNTNGLWNRLLKSLYQLWFIFGVVCLFYIVCVGWVYIVANFSDVKKVVEAISTSTIGINVLIRMIYLRCRFSKFKHVLEKFTNKIWINKVTHPLIFKRCIKRTVPTFYLSITLWMVLFIYCALPIFVLITTDQTIHSNDKTFPYPMIFPYDPQKPINYILTYMTSIYTGAITVTLFYATDAILAIFISFLCGQFEILHGNIARLIPECHAEFLANYRGESTGSKKNDFIFLHNLYVKRLHELATAHDELIRFSMDLEKLFSFQLMVNVVTSTFQICTNLFQFIVAGRNSLSDFLRFFLFFFSVTGQLYVMCELGTILITRSTDTANYLFSCNWEGGILSQHSPLLRQVDYITLDSLNTKLPAWRTLEYYPTNRDFRMKLKLMIMRSQRPVHLTAMKFTVSSLESFTRILSTSMSYFTLLNSFLD

>MdOr15JOI

MFDFLKASMPIAKSFMLVPRACGRLCGVWPDPEYRWRNTLFVIFSTVVTLFGGVGELSYGFTHLNDLVDALDAFCPAVTKIISFFKATIIFINRKKFYDIMQRLRTLIMREQHDSKKMKMVQGFSSFGNICTFIIVSGGSSTNVFYNLRAIITNIIYHFQEEERKLEFPFKSLVPEFTTRFPYFPGMFLILTASGVMTVFSFSIVDGYYVCTTVFICSIFKIIQQDIGSIFDELKDCEHATDEQNHRIRQKLNAIVERHNTIIDLSADFTASFTVIIMLHFMSAAIVVCSSLLDLMLNTNSVGLFIYISYNIAAFAQLFVYCVGGTFVSDSSAAVADVLYNVEWYKCDIKTRKIILMILHRSQKATTISVPFFTPSLPAFSSIISTAGSYIALLKTFL

>MdOr16

MVPNFLKNSYPLNKQYLLIPRFALRILGFYPESEWNVWLKSWAFFNISILAYGCYAELYYGIYYLPIDIVMSLDALCPVASSIMSFIKIFFIWWYREQYKQLIEEVRRLTEDQNTLRKEKMKRWYFTIATRLTALVLFFGLCCSTSYSIRAILTNTLLYLNGKDIVYETPFKMMFPEPLLAMPIYPITFLLVHWHGYITVLSFVAGDGLFLGFCFYFSTLLKALQQDLTEVLGVIDETKKYRKLTESEKVMSLSKIIRRHNEIADLTMKLSSIMVEITLCHFITSSVIIGTSVIDLLLFAGGYGSIVYIVYTCAVLSEIFLYCLGGTAVIESSQELAVKAYTSNWYGQSVRIQKMVLLIIVRSQRHFVVKVPFFTPSLPALTAILRFTGSVIALVKSMI

>MdOr17

MQIRSIEDVPLLSTNLSIMKFWSFLLEHNWRRYFALIPYLFINTTQFLDVYFSTEPIDAIVRNAYIAVLFFNTILRAVLLCVNRFEYEGFMEKIRLLYIELMNSEDPALRKMLQECTVASRFISKVNLLMGFTSCVGFNMYPLFATSKVLPFGMYVPGVDKYESPYYQICFLFQIIITPAGCCMYIPFTNLIVSFILFGILMCKVLQHKLRNLKDVSSEKARTVIVWCIKYQLQLINFVDTINDLTTFTFLFEFMAFGAMLCAMLFLLIIVETVAQMCIICIYIFMIFAQSVIMYYFANELYDQSLKVAIAAYESNWFDFDVSTQKTIKLFILRAQKPCAILVGKVYPMNLEMLQSLLNATYSYFTLLKRVYG

>MdOr18

MLIETIEDVPLYNNSLRIMKFWSFLLRHDWRRYLSLIPYIILTSSQFVDLFFSTEPMDAIIRNAYLAVLFFNTTLRGIAVCIHQSRYEDFLERIRVLYIDMMESEDQWVREELQAITLAANNISRVNLVMGTCSVISFLIYPIFATTKVLPFGIYVPGVDKNISPYYEICFIVQTVMAPIGCCMFIPFTNMIVAIMLFAILMCRRMQRKLRHLCHVTSEEARATIIWCIKYQTELIRYVNTINDLITYTNLLEFLAFGAMLCAMMFTLVTVETVSQMCLICVYILMIFAQSTILYYYANKVFDESLNVGTAAYESEWFDVDVDTQRTLRLLILRAQKPCAILVGRVYPMNLELLQSLLNTTYTYFTLLRNVYD

>MdOr19

MKFLTERKTNKITKYSAKIKRLEDVPMLWFNVRILKFWSVLIDNNWRQYFSYIPFFFLNIFQILDLYYTEKEINDKIHDTYMTMIIFNTFLRAIVMVTNRRKFSESLEYMKDLYAELIMEYDFEIRQIIRKYSDMVLKVSKINLTMGILTGLGFSMFPIMAEEREFIFGMYVPYLNEYQTPWYEILLAVQSVLNLSGMCTFIPFAGMFVSFLVFAMAISKVLQYKLSKLSTEISSKLAERQIIECIKLHLKLISFIDKVNELCSIISLVDCILFVVILCIMLLSFILVKTVIQKCVIVVYMIMVFTQTFLLYYFSNETYHESLEISTAAYNIDWFNYDVETQKVLQLLLLRSQKPCAILIAKAYPINLVRLQAMLRVTYSVFTLLDKFYG

>MdOr20PSE

MRISMRQKDQLISKYSNKIKNIEDVPMLWFNVRILKFWSVLIDDNWRQYFTYIPFFVLNIFQLMDLCLTQKELNDKIHDIYMTMLMFNTFLRTVVMVTNRKKFCKLLEHIRQMYEELMMERDAEICRIMEDHTAMVLKISKINLIMGMLTTXEFIFGIYVPYLEEYQSPWYEILLTGQSFLNLSAMCIFIAFTAMFISYFMFAIAISKVLQYKLSRTCTEVSSKIVEEKIVECIKLHLRLISFIEQINELCGFIALMDFLLFVIVLCIMLLSFVLVKTVTQKCIITVYISMVFTQAFLLYYFSNEVFYESLQISTAAYDINWFNYDVRTQEVLKLLLLRSQKPCAILIVKSYPVNLQRLQVLVKITYSVFTLLEKVYG

>MdOr21

MAFENFYQTNSVENFKMFWFLWRLLGFRGFQNKYANIVHNLVLHVAISFWYPMHLTLGLLSLPNQGEIFKNLSITITCIVCSMKQLFLRWKIRQMHDIEMLFLELDASVESRQEYHFFTNGPRKHAQWITKLYCTCYMGANVAAITMVMLDSQRRLMYPAWFPFDWSSSSQVYWAVLMYQFMGVTTQIVQNLVNDAPAGVLLCLISGHVRLLGMRVSRIGHDSKKTENENLADLGKLFKLVEDTQSYVQLILYISGGLNICVAVVYLIFFVESLTAYLYYSAFILAITIEIYPSYYYGSSCQQEFNDLSYAIFCSNWLEQPKRFHKNMRIFVESTLPKVTMTAGGIVRMQIENFFAICKMAYSLFTLIRSIK

>MdOr22

MPSSTKHFFNSSLNTRFPVIYKVFYFSIFCRRIFHLAHMDIDAPLPKTRDATVYIFRGLNIIGYVPTETNKLAFYMWSGFVNFFVTVYLPVGFLMSFLLRLNTFSPSDFFTSLQIWVNCIGCSLKMFVFFFLHRRLIESRKFMDRLDVRIDNDEDRLVIRKIVAFSNRSLTLYSSLYLSYASSTFLVAVINSKPPYQVFNPFFLWKENVWKFTMQAGFEYMMIAFHCFQQALLDSYPVIFITIIRTHLHILTRRISRLGSISTMTSDERYEALVQCVLDHKNIMGLYSIFCPVISGTMFVQFLIIGLILGITTLHIFLFADRLAIIASLFYVASILAETFPCSFLANCLMDDSDRISLAIFHSAWHEEEPRYKQMICFFLQHTQKTLILTAMKIFPITLNSNINVVKFAFSVYTMMKQMGLGQNLQNVVGKEL

>MdOr23

MMEENRMVSINIKIWKFFAIIYPTSDKLWRLYSIQFVTILLNFMQFMFLIEMWGNLAPFILNVFYVSATFDCLLRTGVIVYNRSKFEAFLAEFDSMYSEIEENGDDYAKGKLKEATEFCRKFSLFNVLASFLDLIGTMSHPILTGTRTHPFGVALPGIDSAVSPYYEIYFILQLHCPITLSVLYMPFVSIFVTFSSFGKTALQILQHRLKDIFEIYDDDETRLEALKECAHYYNRLTRFIKVFDEMVTYVILGEFLLFGAIICSLLFCINIIDTMAQFVSIIMYVGTMLYVLFACYYSANEMLEESLKVSEAAYSIPWYEGTPQFRKTLLLFIQRTQKPLCLTVGNVYPMTLLIFQSLLNMSYSYFTMLRGLKIQ

>MdOr24

MFSVPNPPDALPPQNSLKNFFLIQRICFSVIGLDPTSLKRTMYRPWLTFIPLLSLMGLLGPMGVYAFNYLKIDLGKAITALSPFWQSMLSTIKFFVFMLNRKKIVGLVRKVWSWTLEATEEELKIIDEEIKGDARISLFYYSMVNITGVLAALAPLAISAIYTFHGRGFMETLDAPFKAEYFYDIRASYMGYILCYTWNVLGIHYILNGALSIDTLYSWIVHNIAAQFRILNLRYRQLSEKIIAHQAAGNHNEKEFLKSVVECVNYHRRIIQMSERFSEVYQGLVFIKFLVSCMQLACLSFIIPLGGEFADQSFNLSFLIAVTTQLMLYCHGGQKIQDMSTSVNLAIYEYFHWHDLSIKSQKLLMITMIRAQKPCDIRGIFFTADLSLFVWVYRTAASFMTMLMSMQDK

>MdOr25

MFNVPKAPDALQPQTSIKKFFLIQKISFAAVGLDPTSIRRTIFRPWLTFIPLVSIIAVLGPMGIYAFNYLKIDLGKAVSALSPFWQALLSIVKFFVFMLNRKKIVGLVRKVWLWTLEANEEELKIIAEENRGDAKVCTFYYSMVNITGVLATLAPVAVAAIYAWQGHDFWESLDAPFKAEYFIDIKASIVIYAACFTWNFIGIYYIVNGSLSIDTLYSWIVSNISAQFRILNLHYHQLSQNIIAHKAMGNHNEEKFLKSIIDCVKYHRRIIQMSERFSEVYKVLVFFKFLVSCLQLACLSFIIPLGGEIADQLFNLSFLMAVTTQLMLYCHGGQKIQDMSISVNWAIYESFHWHDLSIKSQKLLLLTMIRAQKPCEIRGIFFKTDLSLFVWVYRTAGSFMTMLMSMEDK

>MdOr26

MLKPPIAPDSLPSQTSIKNFVFIQRICFWAIGLDPTSIKRTIYRPWLTVIPLLAMIGLLGPMTAYAFNNLKMDLGKAISALSPFWQAILSIVKFFFFMVNRKKILQLLRDVWLWTLEATAEELEIIAEENKNDAKICGFYFAMVNISGVLAHLAPLAVASVYAWQGNGFLNSLDAPLKAEYFFNIRQSYITYIVCYLWNVISIYFIIYGSLFIDTLYSWLVHNISAQFRILSLRYRKLSLMMVTHKSSEIQNDEIFMKSIVECIQYHLRILEISKRFSEAYQHLVLIKFLISCLQLACLSFIIPLGGEMADQLFNLSFLVAATTQLILYCHGGQKIKDMSTSVNWTIYESFHWHNLSVKSQKLLLFVMMRTRKPCEINCIFFRANLNLFVWVYRTAASFVAMLMSLQNKI

>MdOr27

MFKIPRAPDALPRQPSLRKFLYIQKICFAGIGFDPTSVKRTIFSPWLTFIPLFSILGLLAPMGVYAFKYIKIDLAKTTAALSPFWQSLLSSVKFFVFMLNRKKIVESVRKVWLWTLEANEEEVEIIAEENKYDARISKFYFASVYVTGVLAVLAPLAIASVYAWQGYGFLESLDAPLKAEYFFNIRGSYQAYIFCYVWNCIGIYYVLHGALSIDTLYSWFVHNISAQFRILNLRYRQLSERTMMLRAIGEHNEEKFITAIIECVKYHRRIIQMAERFNDVYKGLVFIKFLISCLQLACLSFQIPSGGEIADLLFSLSFLISVTTQLMLYCHGGQKIQDMSTSVSLAIYEHFQWHDLSVKSKKLLLLTMLRAQKPCYVRGIFFTTDLSLFVWVYRTAGSFMTMLITMDGKK

>MdOr28

MTSDDLPPLEGVKYYFVVQKFCFTAIGVDALSARRTIVNGFLFWIPNIVQFILSQPLTLYSLQHLEDMSLVTDAMAPVWQVLMANMKMALFLWHKKEMKKLVRDLWLWNLEATPDELKILEVENRKDTMTSFSFYMTVLTTGILALTSPFFKAFYRYLKGDNYWDALETPLKGSYFIDPKETYMGYFIAYMWAFIAIYAVLNTTLAADSLFSWIVHNISAHFWILRERLKSIAATNREGSHGYGKFRKSIGDCVRYHQRIIDTIDEFNKVFMTIVFVKFLISCIQIAFLAFQFVRGGDFAGQVFHMLFLMSISIQMMLYCYGGQRIKDESASISVAIYEYFHWDLLCPKSRKLLLLPLARSQKPCKLTGVFFIADLSLFLWVYKTAGSFVTLMMSVSDTSN

>MdOr29IP

MATPSADVLPPLEGVKYYFVVQNFCFRAIGVDLLSMKRTMVSGLLFWLPNILELAICVPLARYALENLEDMSLVTDAMAPVWQVLMAILKMALFMWHKKDIKKLVWNLWLWNLEAKQEELEIIADENRXDTVKSFSFYMTVLTTGILALTAPYYVDPKGSYLGYFTVHIWTCIAIYAVLNTTLAADSLFSWIFHNISAHFAILRERLICVAFSETEGKQSYANLKKSLAEYVRYHQRILDTIDDFNEVFMMIVFVKFLISCIQIAFLAFQFVRGGDFAGQIFHMFFLTSISIQMMLYCYGGQRIKDESISIAVVIYEHFQWEVLCPKSRKLLLLPFARAQKHSELTGFFFTADLSLFLWVYKTAGSFVTLMMSVSDTSK

>MdOr30JI

MTSDDLPPLEGVKYYFVVQKFCFTAIGVDALSARRTIVNGFLFWIPNIVQFILSQPLTLYSLQHLEDMSLVTDAMAPVWQVLMANMKMALFLWHKKEMKKLVRDLWLWNLEATPDELKILEVENRKDTMTSFSFYMTVLTTGILALTSPFFKAFYRYLKGDNYWDALETPLKGYLGYFTVHVWTCIAIYAVLNTTLAADSLFSWIFHNISAHFAILRERLISVASSETEGKQSYANLKQSLAECVRYHQRILDTIDDFNEVFMMIVFVKFLISCIQIAFLAFQFVRGGDFAGQIFHMLFLTSISIQMVLYCYGGQRIKDESTSIAVAIYEHFQWEILCPKSRKLLLLPLARAQKHSELNGVFFTANLSLFLWVYKTAGSFVTLMMSVSDTSK

>MdOr31

MTRILKRYFRLQRFIFSGLGLDIAATPEKMVKRPWLMMTPLVMSILLCIANGHYVLDNASDYLEATDSLTLLCQSLISVWKVIMVIWKRKEFANMIARIERLNVKAEGEELKIVRRENTRDIIFSTTYFVLVLLTGAWSLLVPIYFAVHVYVTTGEVDLPVPHKATYFWNHEHVKGYSLVYIWDVFIIYFIACSAVSTESMFSWLVCNIIAQFRILMHRLEVASRQVMSTRPMTASHHVDDDDDNPLMGELDPQAGMVDAIIACVKFHRRTLRLTQELNSLYGAIIFVKFIVSGTQICCLAFHLVRGNNSLFNVAYLCMFLSAAALQLILYCYNGQRLKDESLLVTTKIYSIFPWSKMPVSTQRMLLIPMIRAQQFSELRGVFFTVDLSLYLWVFRTAGSLIAALKTLEEKE

>MdOr32PSE

MKIVKRYFGIQRRTLTAIGIDVNAFLPNGPERIAKHPLLLLVITVMPVLQYISIGHYAYKNSNNMVTATYSFSLSCQGVICLTKILIFLFKRRDIVKLVKMLQEDVFNAKSDELVITKEENSRDVLHCTVYGSAVFSTGFFGILHRLLRPSSSTSNMGIWCWYHHILPXYLWDYSHLPGYSLVYIWNMMRMYTLAFASVAIDSLFSWLVCNIVAHFRILMLRFQRAAWLTPGLDRPEVSVSREQERLIFDCVRFHNRTLNLVQELNLVYGGIIFVKFVVSSVQICCSAFFLNSFGASQSMAKLMYQFLLLSAVALQLMLYCYNGQRITDVSFQVATKVYSTFPWSKMPASTKRMLLPPMIRAQRFSELRGVFFTVDLSLYLWVFKTAGSLIAALKTLEEDK

>MdOr33

MKILKRYFGMQKFAFAALGVEVESMSPAGSERIFRHPIRYAVLFILTVLQYISIGHYSYVYTSDIVSAAYSIALSCQGVICITKLVIFFFKRQGIVELVRMLQTDAFNAQSEELAIIKEENRKDIRICTLYCIVIYGTTFFGMTLPFARTILGYLRNGYLVYVTPVASPSLWNYDTVHGYTLVYIITLLRLGTLCFTTIGIDTLYSWLMSNIVAQFRILTHRFQQAAWATTALDGSEISISEEQHRLINDCIRFHNRTLDLVKELNRVYGAITFVKFVVSSIQICCSVFFVSSSDSKESAFNLFYQSIFLGAVSMQLATYCYNAQRITDESELVATKVYLIFPWSKLPIPTQRMLLLPMIRAQRSCEMRGVFFRIDLSLFVWVFKTAGSLIAVLQTIDEAQ

>MdOr34

MNSREHRELLEIFYKKQSYVFRLLALWKLPDTVTERFRLLHRFYFYYILFFWVLSFDASCMIQFIANITDLNEVIKVFFIFATSLAVFAKFATIKLKNHLYAELIETIHEPAYRPVNSREVKIFRQTHRLCGTVRNFYLVISLCALNVVMLTQYIFDNSELPLSLYNPINIDTKLRYRLMYLYQYVAVSICCYMNIAFDSISASFMIHIKGQLDILCDRLEHLGMDQESRDEDITRQLKNCVKYYGDIIHIVRIAENLISFPISIQIACSVLVLVANFYAMSFLSDPGDYANFIKFLIYQLCMLSQIYILCYFPSEVTAKSEEVPYYLYCSNWVYWNRMNRKLTLLMMTRFDIPIRIRSINPTYTFNLAAFTSIVNSSYSYFALLKRINS

>MdOr35

MNSLEHREAMKTFYKKQSFIFRIFAQLKLSDTVSDRFRLLHRIYFYYILIGWVLSFDISCLIQFISNITDLNEVIKVFYIFATAMGVLAKFLAIKIKNNLYAELIEAMHEAKFRPTNSRELQLFRESQRLARTVRNFYTTISLCALNALLFTQYIIDTTQLPMSIYNPINTDTKLRFVLVYIYQYLAVSVCCYTNIAFDSISASFMIHAKGQLDILCDRLKHLGMDSETSDEEITAQLKNCVKYYGDIIHIVKIAEDLISFPISVQIACSVLVLVANFYAMSFLSDFANFIKFLIYQLCMLSQIYILLYFPSEVTSKSEEVPYHLYCSKWANWSASNRKLTLLMMTRFDIPIRIKSINPTYTFNLAAFTSIVNCSYSYYALLKRINS

>MdOr36

MFHHKRELIRTFYIRQYQLLKLFALWQLPEDASAYQRLGYRIFFWCFLIFWMLLLDCCMILQIATHLGDVDEVIKVFIIFATAFAVMGKYLYLKIYNYRFEQLFQMMHQPEYLPENPTEWQIYCQAIDLSRRVRNYYASLSVSALSALFLSQFLGDEQELPASIYYPFQLNTNWKYGLMYVYQCVSLAILCFVNVGFDSLTASFFINIKGQLDVLGMRLQTIGVGVRDQRRILKKLKDCIRNYQRILRMTHLMEELVRIPMSVQIGGSVFVLIANFYSMSMLSDNADMGIFAKLLLYQTCMLTQIFILCYFANEVSLKSSDISFNLYESNWYDWDKVNRKLVLLMMIRFDTPISIKSINRCYSFNLAAFTAIVNSSYSYFALLKRINS

>MdOr37

MAEVERYFEDFVNLPCVLLKTLGYDFLEISRPWLARWLMKLYFFLTLICCLYCTYFVTDEIFADIVSGANNLPLLLRLINDFNYNAIGILKSFYFFRNIKSKKELFRKFREIFPTSIEDRFAYRVNESYWPRWITTTLYLYFCATALILFSPLAESIIEYFVDLIKVGYADAEFTYHKLYEEQSYVVDHRNPLGYMVIYSMEVMNSHYAIVFNICPDIWLIAYAIQLCMHFDYISRNLESYEPMEKRQQKDLKVMAELVRKHQVLLELADDLKEIFSLLVLVMLFSTVATLFCAAVYVLTQGINKNVLGYMAFLPTSLGQYFMVCYYGQLIINKSLQIGEAAYSQTWYNGCQSYKKSILAILGRAQRQCEINAGGFQTTNLKGFESVMRMTFQLFTLWRTMMEPK

>MdOr38

MYSVFQQPLTVMATTERYFEDFVNMPCALLRTLGIDFLNISRSLLAKCLMQLYFVLSLLSCFYCTYFVMEMAVREIHCGSGNLPLILRLVDDIFHSLNGLLKSYYFFRIWKSNKSLFNRFCEIFPISMEDRREYRVNDYYWPRWITCMVYVQCGAIAVIIFSPFAATLKDYFLAILKFGFSDAKFSYHILYEEHTYIVDHQRPTGYIFIYSVLAMGTQYAVIFNICPDIWLVAYAIQLCMHFDYISRNLENYEPKEERSHKDLEVVAKLVKKHQILLDLANDLRKTFSILVLIMLFSTVVTLFGAAVYVLTQGINSNVLGYLAFLPTTLGQYFMVCYYGQLIINKSLRIGDAAYSQTWYNGCQSYKKSILAILGRSQSQCEINAGGFQTTNLKAFEGVIRMTFQLFAVWRTLMEPK

>MdOr39

MKVTAFSSSALKTAEKELYFDDFVKLPCVLVRTIGYDFIDKPRPLWLRALMLLYLVLCLIFCAWFTYFAWDFMMAEIAAGANDLALVLRLSVDVIYNVAAIVKSLFFFRNLKSLKSLLQRFRDIFPISREDRLAYRVNDYYWPKWITTILYMQLFALSIILFLPFVEAVYEYFGALLTVGYANAKFGYYRMYPETTYGINHYNPLGYIIVYTMDIMNGHYCTVWMMGPDVWLVAFSIQLCMHFDYVSRTLENYKPSKERAAQDLRVLAELVRKHQTVLELADDVQENFSVLILVMLFSTASILFGAAELVITQGITAHVLGYLAFVPTGVGQFYMICYYGQLIINKSLQVSEAAYNQTWYNGCQSYKKSILTIMRRAQCHSEINAGGFQTTNLMAFESVMRMTYQLFAIWSTMTSSK

>MdOr40

MTTKERTFADLAKLPCVLLKTLGYDFLDQPRPRWLRMLLTLYFVLCLMCCSYFTYFALDFAVAELAVGAKDLPLLLRLIDDIVHNVVGILKSYFFIRNSRSIKKLYKKFGDIFPISMEDRLAYRVDEYYWPKWITTILYMQLCALTIILFVPFAESIFEYVGALISLGYGNAKFGYYRMYEETSYGFGHHNFLGYVVSYSLDVMNALYSAIWMICTDIWLVAFALQLCMHFDYISRTLENYEPHKERSQDDEKVLAGLVRKHQTILELADELKINFSALILVMLFSTISMLFGAAELVLTQGITTHVIGYLAYVPTSVGQFFMVCYYGQLIINKSLRVSEAAYSQTWYNGSQSYKKSILTIMRRAQRHSEINAGGFQTTNLMAFESVMRMTYQLFAIWSTIMESK

>MdOr41

MSIVRVKKARVNFQRDFRDFCHLPNYLMRIYGRDFSERKRTKWQTLLLRLYAVVTVSSHIYCFYFISQQVFLMFLSGVPNLELFLRLLSGFNYGLFAIMKYLAFKNRITDAAAINRVLREIYPKAGRERILYRVNAFFWPKWMLTVIYFYFGAVAFIVLSPLLESVIVFVIGVGRLGWNEAQFGYIKLYDIPYSFDHRSPFAYVLTYSIELFHAQFVIICNVCGDIWLLCYAMQLCMHLDYLIKILEHYEPRVEHHLRDTQFIAGFSQKHQILLNIADDVNTVFGVQLLLILISTAATICCAGIYTLTQGVGKELLEYVAFLPCVVGQYYLICFYGQRLVSSSENVGAAAYNHAWYNGSPSYKKSVLVIMTRSQRSMKLKAYGLSSVSLGSFRMVMSESYRFFAVLKHAVFDKKN

>MdOr42

MFEDIPLIYMNVKILKFWSLLYDHNWRRYVTLIPPTFLVFTQFYYMFMTEEGIDAIIRNSYMLVLWFNTILRAYILIKDRVEYQSLLQDLEAYFYDLDKSNDVYVRNLLSHVNSNGKVMARGNLFLGLLTCIGFGLYPLLAAERVLPFGSIIPGIDEYQSPFYECWYVFQMLITPVGCCMYIPYTSLIVSFIMFGIVMCKYLQRRLATLSRFKGQPEWIYDEVIECIKYQKKIIEYCETVNRLTTFMFLLEFVAFGTLLCALLFLLIFVDSAAQAIIVCAYITMIFCQILALYWYANELKEQNLSIAAAAYETEWFTYEIPVQKLILLMIMRAQKPCTIKVGNIYPMTLELFQALLNASYSYFTLLKRVYG

>MdOr43

MAPSMEINSNEFFKINRTCWKLLGLGMLMVEGHKTNGQRKMSTNLYMVWAIVINLMATCCFPIHLFLGIFESENKTSFFDSISITITSIGASTKLLIIAIKMKKILEMQSLLRTLDARITHHEEVRHFRQDIRSRIMNIQRLYFVVYCGVGISVLGAFLFSKEQRLFYSGWFPFDWRSSLGNYAAAISYQCIPIFFQMMQTFCNDSFSPIALCVLSAHIELLYMRVVRIGQDKNGKMRETTTLQEDEEELNRCVLDQMNLYELYNTMQNIISWAMFIQFFVSVVNNCVAIVALLFFVTDVFERIYYVIYILAMGIQLFPTCYYGSDFVLLFEKLHYAVFSCNWIGQSKSFKRHMMIFTERSLRETVALAGGIFPIHLDTFFGTCKATYSLFAVVMTMK

>MdOr44

MTEEPNTKALFKTHFIAWRILGMSPPDNYRPLYWIYSILLNIFVTIGYPLHLIFGLFTSTTMYEIIQNVAINFTCSVCAMKTIAIWWRFNKVDVMFEIIQRQDQRFTSHEEIAYLRKEVYPPVRRIILLFSILCTFIGISGESAVLVTGLLGTWNLMYKAYFPFDVFASTKNYMAAHLYQFIGISYLILQNVVNDTFGASHLCLLRSQVRMLNIRVTKIGHDPKKSREENNQELLECIKVHKDLLEYRRQLEEIISIYMFFQILIAALNMCVVLVFIILFVRDIFTLAYYVSYLTSMIFEILPSCYYGTLLEDEFEDLAYALFSCNWPKQTLEFKKNLRIVAEQAKRRIYVTAWLFRINNNAFLIACKNAYTLFALVMNMK

>MdOr45

MSETKLHTKSLFWAHFACWPILGMMTPPNVKYKALYWIYSFAVVTILMIGYPLHLILGLVSSSSLKELMQSLSITLTSTVCSIKTMAIWWRLNKVTDMFTIIRRQDERVRSTEEVDYMKNVVYPQVRFVIRLFYVICGFLSLFGELSLVVAGLLGNWRIQYKAYFPFDPYANTKNYVIAHVYQLLGVNFTLVQNIVNDTFASSHLALLRGQVDMLARRVAKIGHDPQKTQRENNQQLLECIRDHEDLLEYRQILEEIISVYMFFQILLCGLNMCVILVYMVIFVRNDVITLSYYSTHLIGVMCEILPSCYYGTLLEDAFQDIAHALFSCNWMDQDLEFKKNLRIFIENSSRRIYVTAWLFRINNNAFIVACKNTYTLFALVMNLK

>MdOr46

MDKELNTKSLFRTHFKCWRILGMMPSKKYRLLYWIYSLIVNLLVTIGFPLHLILGLFQSTSLYEVIQNLAITLTSTVCSMKTFAIWWRFKDIERMFDIIRKQDEHTRHGEQLEYMKRKVYPPIRSLINLFYILCSMVALSAESSLIFNGLRGSWALMYQAYFPFDPFGSSGNYVVAHIYQFIGIIYTVTQNLVNDTFAGAHLSLLGGQVRLLGMRVAEIGHDPKKSLAENNKALLDCIHDHLDLLEYRRKVEDVISLYMFFQILFSSMNMCVVLVFMLLFVKDTFTMSYYLFYFVGMIFEVLPSCYFGTILEDEFQELSYTLFKCNWADQNVVFKKNLRIFVEQASRRIHVTAWLFRINNNSFVTAVKGSYSIFSLIMNTR

>MdOr47

MALLQNKLNTKSLFNTHFMCWRILGMLPPQNYRPLYWVYSFIVNLMVTIGYPLHLILGLLTSTSMYEVIQNLAITLTCTVCSMKTFAIWWRFQDVDRIFDIVNRQDEHTRYGEQSDYMREKVHPPIKWLIILFYILCSMVAISAEVSLVVNGLRGSWLLMYQAYFPFDPFGSSMNYAVAHIYQLIGLVYTVTQNLVNDTFAGANLSLLGGQVHLLGMRVANIGHDPNKSMEENNKELLDCIHDHLDLLEYRRKVEDVISLYMFFQILFSSMNMCVVLVFMLLFVKDPFTMIYYMFYFVGMIFEVLPSCYYGTILEEEFQDLAYSLFSCNWTEQDVVFKKNLRIFVEQASRRIEVTAWLFRINNNTFLTAVKGSYSIFSL

>MdOr48

MADELNTKALFKTHFVAWRILGMLPPTKYRPLYWMYSVFLNLAVTIGYPLHLIVGLFTTTTAYEVVQNIAINLTCAFCAMKTIAIWWRFNKLDIMFEIIQRQDERVISEEGVAYVRNVVHPPVRRIILAFTILCSVIAASGESSVLFNGLLGNWTLMHKGYFPFDISNNTRNYAIAHLYQIIGLSYMILQNVVNDTFAASHMCLLRGQVQMLNVRIAKIGHDPKKSREQNNQEFLECIKIHKDLLEYRRQLEEIISVYMFFQILVAAFNMCIILVFIILFVKDVFTLIYYILYFSAIVFEILPSCYYGTLLEDEFQDFAYALFSCNWPDQDVGFKKNLRIVAEFASRRIYVTAWLFRVNNNAFIIAVKNAYALFALVMKVK

>MdOr49

MMSEKEVQMLKKSNYNKIKELIRISFTLGVNLTSPSTLKDSLKIINIILVVSSVISFYGHWCYTIESIKDIPKIAESVCTGFQTLISVIKMVYYLFIQRRLYYLLYKAQTHEYIRKIDIFHKNFPMSERLQAKIDEILDASWKNINGQLIFYICCCAAIISNYFFMALFQNIYHTWKETPNYEFVLPFPSVYPSWKDKGMSFPYYHIQMFLGTCSCYISGMCAVSFDGVFIVLSVHGVGLVKVLNMLIENSTSADVPKERRVEYLRYCIYQYQRISDYTDELRKIYKHISLTQFLLSLLVWGIVLFQMSVGLESDLMTLVRMIMYISAAGYEIVLYCYNGQRLTSECEKIPYAFFSCDWFNESKEFQELTRMMILRSNRSFFMEISWFTTMTLPTLMAMIKTSGSYFLLLRNVAE

>MdOr50

MSQLLLDLLKEKQLENNKILNTFYRISFMTGVKIKYKTQFKDPVKLINLFLISVSLVGLCAQYCLVWNKRKEPFVESADAICTANQAWISIFKLIYLVFVQHKFYELLHTAINGSLLYDLGIFDLAIDCKQYLLQEINTILDSSWRHIKYQVNFFTFSCMMACGFYMFSCIAANYYYTNIQPQNFTLQLPMPALFPMWHDYGMTWPYYPIQYFIAGIENYICGMCAVCFDGIFIVIVVHCASLFEILHMLLEHVDDIPQSERVDYLLCCARLHVRIYNYYAKINGMYKNPSLAQCVLSMLVLCVVMFMASIGLEEDITLFVKMLCFLCAAGLQIAIYCYNGQKIITQSEKSPDAWYNCCWYNESKQFKYIIDMMIMRTNRTLYLQVSGFTTMSHMTLLSIVQTSGSYFLLLKNLNGID

>MdOr51JIP

MYLALKETKANQILKYWKWIAFTSGCNIVYKTKFMKLFKLILNMSLAISAAIGCYGQAQFFWNHRHESFDVYLEAILIFFQIVISISKLMLFTLKQQQIFEIVQDVQNGEILNDLEIFELNLINPSKILKDISAIMDQSWMSIKFQLNFFIGNVVVLCGVYLFKNLILNIHNFKNEGDRFQLAYAITFSGLFALISTHCRGLLRVLRTLITYSTTYHVLPEDRVKYLQGCIKLQQKIYKICNELNSLYRIPALAMFLVSCLVICLLTFYATVDGGNDISTIVKVILFISGAYFEVAIFCFNGQHITTESEHLPLDIYGTXNGTKRANNLRRFMIQRSNITILMDVGGFTTMSFVTFLTIFRSSLSYFLFLQECM

>MdOr52

MDTILIDISDKGGRILNPLKWIGMFSGCNIKYKSKFLHPLKILNLFLFVTSILACYGQLYYVWERRHYTFEIYIEAILIFFQSLISIWKLWMFTFSQDCLFDMMKSVENSETLQNLEIFQLELIDSANIINDITQILNESWIDIKRQLLLLRFTVFGICSWYTGHSLVSNIYYLYISDENDKEKLEFPFPASFPVWYSNVNSLWHFYLEYFVVTMQIYLATVASITCSGLFSVISVHCLTMLRVLRTLITYSTSEHVPSQHRTKYLEACVRLHQNLLSFCSRLNRVYQKPSLGLFISCCLLICLLTFKASVDLGKDISGSIKVCLYLLAAFYELLIFCLNGQRITSESERLPQAIYSSLWFDENRNFKFMIQIMIMRTNQNIRMDVGGFSRMSLETLLTITRSSVSYFLFLRNCM

>MdOr53

MAGNIQLSPSERFAKFIKVIKLFAGFCGVNSLERDYRVTWVTWLVICVVTSFFVCTFYTIYVGMAIQNNYSILLQSLCITGTGVQGYTKLLNAIFCGKHLRFAFEELTAIYEEYECKRLEYRDNLKENLEMVKRLIYGLLLINFILIAALFAVPLFYYYVRKEKIDVIPLMIPGINPSNNRIENYIYQFYHICCVIFSTFGNFASDTFMILIVVHVPMIKNIFKLKFDDMAETMKLHLRNRKKTEPLLRDIFQWHQKTILIIETMQKGFFWVIFVQIFTSMLNIIFTIVCIFLGVWPVAPVYLLYSFVILYIYCGIGNLVEISTDDITSIIYDFIWYDLTVSEQKMILIMLRESQSPPTMTIGGVMPLSMNTALQLTKSIYTIAMLLNEFVN

>MdOr54

MATKLKLTPSQRFSNFVRVVKIFAIVCGANIFRPDYRLNALTWFVIGVIATFFIFTSYTMYVGVVIDNDYTKILQLLCVTGSAIQGATKLVNGLYHASLIRSLIAEILTMYEEYECKDQRYIKYLEHTLSLIKRAVFSLLNIYSIQTIGVLAVPLFYHLLLGQQIDIIALLVPGIDKHTDFGFYTYQFYHFCVVGFASFGNFANDTLMVLLIVHVPLMKNILKLKFDALDELLKEFPRDVDRTEPLLREIFQWHQKSTMFAQNCTDTFFWVIFVQIFASTLAIICIMVCQFLGVWPAAPVYMMYCFAIMYMFCGLGNLIEISNDDLTRIIYDCNWYELTVTEQKMILLMLRKSQQAPTMTVGGFMPLSMNTALQLTKTIYTAAMILNEFVN

>MdOr55CTE

MVISVVQRNEFIVRIIRLSSKYCGCDVLNPEWQMNLLTWTVITFINMFSILTCYTVYVSIYLEGEWSHSLQALAMVGSGVHGYAKLLNAIRNKAYFRFLVDELHTIYKEYNEKKHSDYRAYLHKTMNRTVVGLKSMGIVYAIVVCSLITVVPFYRFFFNQRVFIMQFLLPGLDPKVERDFIIMNVVHFFSILFGGFGSFAADLCFVLLVFHVPQYKDILSCKFQEINEALELDEMERSGELLRDIFEWHQRYMKFISIVKENYFWVILVEMATIFLCLALSLS

>MdOr56

MTVSVVDEYEGIVRLIKLCSGVCGANVFVANYKVNVLTRIVVTFINLYFIFTGYTLYINIFIEKDWTHMLQVICFFGSALQGYCKLLNAIWNKDHLRYLVDDLREVYAEYAPKHDEYRDCLQKSINTAVKCIKLMAFFHVAITVGLIGVVPFFRFVFNERIFVMQFQLPGVDGDTEYGYLIMNCMHSICIIFGAFGNFAADLCFFTFVSHFPLFKGILSCKFHDLNDVLEGSDDAKKAECKEMLKDIFRWHQKYMRYITTVKDNYFWVLLVEMATIALSISSTLFCLLLGTWPGGQTYLSYCFIMLYIYCGLGTVVEVTNDSFTDLCYTQVIWYKLPAAERKMLLMMLMMAQKTGGLTIGAVIPLTVNTGLQLTKLIYTLTMMLINFLD

>MdOr57

MAKTVAQSYDKTILFIKISSAVCGANVLSPAYRMNILTWIVIVCINLYYVFTGYTLYVNIYVEKDWPNVLQVLCYLGSAVQGYCKLLNAIHNKESLRFLDQELREIYLEYDQKHADYRYCLKTTIDRANKFIKFMIIFQILISGSLIGVAPFYRLVFNQRIFVMQFLLPGVDPSTEYGYFVMNCMHCICIIFGSFGNFAADLFFFVVVSHVPMFKDILTCKFHDLNDLLEEEVADNENNNNNNRIKDVREDFRSLLIDIFKWHQRYLRFIAIVKENYFWVLLVEMGTVALSLASTLFCLILGTWPGGQSYLAYCFIMLYIYCGLGTVVEVTNDGFIDSCYTEIIWYKLPVSQRKMLRMMLMMAQNTDGLTIGSVIPLSMNTGLQLTKTIYTMTMMLINFLE

>MdOr58

MAKTLVQRYETIVRLIKICSAFCGANIFHPSYRMNILTWTVVIFINLYFAFTGYTLYVNIYIEKDWPNILQVLCYLGSAMQGYCKLLNAIGNKDNIRYLTDELREIYRKYDLKHTDYRCCLQKSINTVNRFIKCMAIIHFSITMSLIAVVPFHRVVFNERIFVMQFLLPGIDPNTAYGYLMMNCMHCICILFGSFGNFAADLCFFTIVSHVPLFKDLLRCKCQDLNDILEEGKDVEQEGIGDCQILLKDIFQWHQKYMIYITTVKDNYFWVLIIEMGTVALSLASTLFCLILGTWPGGLTYLAYCLMMLYIYCGLGTLVEVTNDGFIDSGYTDVIWYKLPMVERKMIQMMVMMAQNTGGLTIGSVVPLTMNTGLQLTKAIYTMTMMLINFLE

>MdOr59

MNLEDSRNANKLHRPSNRLRKIVRITRICSYICGADVFDPNYCVNIRTYFVLAVINFSILLLSYTMYSGWVEEGDWAIVLQVLTIGGGTLLQGYCKLINSIRQKDKFRFLLTEVYSIFEEYELKSCDYARHLKKGCHLLSYFMKLCAVINVMMICGLILVAAAINVIFQKRDLIVYGDVIGIDPSTTSGFYVTFMVQACFLLVGGFGLYAGDMAFFTPISQVPTLKEILRCKFKDINAAMEGDELQDSRHVSELLKDAVQFHQKYLRFLNTTQDTYYWVILTQISTYSVGIVCSMFCIFLGTWPGGYIYLLYCFVMMFVYCGVGTMVDIANEGFIDACYNDILWYKLTASDRKSLLNMLILCQNTDGITIGSVLPLSMNTGLRVTKTIYSIAMMLINFFMD

>MdOr60

MAKTHTERLLKIVRITKFCSDICGVNIYEDDYRINYRTFFVIAVIGTSFSFLSYTMYDGYGKEGDWTILVQVISLAGGTLLQGFFVLILFLTKQEKYRFLLKECIILYEKYEKMDSDYRVYLNKGIHLLANFMKVCAFINFMLVLGMTFVTIFYNLIFGTNETLVYGYCPWVSLETTGGLWTTNMVQALLIAVGGFGLYSGDMSVLTPISQIPTFKGIIQCKFRELNDLLDDDHESEMAKKIKTLAALKDILQFHQTYLRFLDVSREAVYWSVFVKVGTCFIGIAFALFCILLGSWPAGYIYMLYCFVMMQVFCGMGTLVDITNEEFIHSCYNDVRWYDLTISEKKMLNIMLMMAQNTEGLTIASIMPLSMNTGLQVTKTIYSLTMLLLTFVN

>MdOr61PSE

MAKKHSESLLKIVRITKFCADICGLNIYADDYRINYRTIFVVLIIGSSFTFLTYTMYDGYGKEGDWTILVQVISLAGGTLLQGFFVLILFLTKQDKYRFLLKECIILYEKYEXMDSDYRVYLSKGIQLLANFMKVCAFINFMLVLGMTFVTIIYNLIFGTNETLVYGYCPWVSIETTGGLWATNMVQSLMIAVGGFGLYSGDMSVLTPISQIPTFKGIIQCKFRELNNLLDDDYDSKLEKESKTLAALKDILQFHQLYLRFLDVSREAVYWSVFVKVGTCFIGIAFALFCMLLGSWPAGYIYMLYCFVMMQVFCGMGTLVDITNEEFIHSCYNDVRWYDLTISEKKMLNIMLMMAQNTEGLTIASIMPLSMNTGLQVTKTIYSLTMLLLTFVN

>MdOr62NTE

GFCVFLTFIKEQENLRFLLTECYDIYEKYERMDSDYRVYLDRGVRLLAKLMKLSAFINAMLVFGMSSFTFLYNFIYGTKATIVYAFAPGLDVATPVGFWATNFIQAGFIAVGGFGLYSGDMSVLTPISQIPTFQGILQCKFREINQLLDDDYESAEERGIKTMAALKDILEFHQKYLIFLKVSREASYWSVFAKVGTCIIGIVGALFCIMLGSWPAGYIYMLYCFVMMQVFCVMGTLVQKTNDDFIHACYNDVRWYDLTIREKKMLNIMLIMTQNTKGLSVGSVIPLSMNTGLQVTKTIYSLTMLLMNFVIENEA

>MdOr63INT

MNLEDTRNINQVYRPSNRLRKIVQITRMCSDVCGADVFGHGYRVNIRTYMVLGIINFSIIFLSYTMYSGWITEGDWTIILQVLTIGGGTLSQGYVKLVNSIRQQNNFRYLLGEVYSLFEEYELKSSDYAVYLKKGCDLLSYFMKLCAVINVIMICGLILVAAAINVIFQKRQLIVYGQIFGIDPSTSTGFFVTFSVQAGFLLVGGFGLYAGDMAFFTPISQISTLKEILRCKFKEINEAMQGDELSRPSNISELLKDAVQFHQRYLRNDGFIEACYEDILWYKLTASDRKSLLIMLILCQNTSGLTIGSVLPLSMNTGLRVTKTIYSIAMMLIRFLDKED

>MdOr64CTE

MTHRQSDRFKAIVRITKICADICGANVLEHDYRINVRTVLVFVIIILTFVFMSYTIYDGFFVQGDWKIILQVLSIGAGTLVQGFVKLLNCIQQQENFRFLIGELYDIYEEYELKHTGYQRHLNKGIHLLSYIMKLCAFIAVLLVIGMAAVTVVRSLVFDVNQVIVQCLIPGVDHTTPRGFFLTCIVQISFIAVGGFGFYAGDMAFFTPITQIVTFQGILRCKMFDLNEVLEKDGEENVKKSTEMLKEVIKFHQRYMVFLTVTQDTYFLVILVQIATYSTGIICTIFCVLLGAWPGGYVYMIYCFVMMYVYCGVGTLVEVT

>MdOr65

MAKTLVQRYETIVRLIRIFSGICGANIFNPAFKKNIITWIVIIFIYQYFVFTGYTLYVKIYIDKDRPSVLQVLCYLGSAVQGYCKLLNFLWNKDDIRYLIYELRDIYEKYDLKHADYRCCLEKNTNRVNRFIKFMATMHLVITITLIAVVPFYRVVFNERILIMQFLFPGVDPNTAYGYTIITTIHCICILFGSFGNFAADVCFFNIVSHVPLFRDLLRCKCQDLNEILEEERASEEEGFAEIELLLKDIFQWHQKYMRYITTVKENYFWVVLVEMGTVALSIASTLFCLILGKWPGGLTYLTYCFIMLYMYCDLGTIVEITNDGFIDSCYTEIIWYRLSIHQRKMLQMMLMMTQNTEGLTIGSVIPLTVNTGLQLTKSLYTMTMMLINFLE

>MdOr66

MNTHYRLQDFMVYPNIAFNLAMVQPFRLSGTLEEHQTANRCRGFMKSMLIKLWFVFGAVNLIYQNVGMLAYLLLPQLSEIFDDVEMVAKISETGGILGLTMVAVCKMFVLFWHGRRISILLQELEEIFPDEKEQFAHPTLYRVRHFAQTSERLMGRTTKFFIFAFCFYNSLPIAELLYELLLPDQEIKYRYQSNTWYPWQTKDNARTWLNFIASYVCQVQSSLTGVGFIMAGEFMLCFFITQMQMHFDYLTNALRHLDAASVRANEKLKYLIIYHTKLLRYSKEINEIFNISFLVNFITSSIAICMMACSMVMLSMAHTFKYSVGLLSFLVFTFFICYNGGEFTDASDAIMPSAFYNNWYEGDASYRRMILFFILRSCEPNVLTAYKFTTVSMPTFMAILKVSYQLFTFLQAMD

>MdOr67

MLYRPRLPDGRKVPLSWPIALFRLTNNICWPLEENASWLAVVFDRFCWYLAFILFVITNDAEFRYLRVNINNLDEMLTGVPTYLVLIEIHLRAFTLGWRKQDFRRLLEKFYRQIYIESSLHPTIFKNIRSQLMPIFVLSSLYLSALISYVILPIYFLSIGSRELMYKMIPAFDYSPLWIYLLCCLSNLWIGVIVATMMLGEATVLSTLVFHLNGRYLMMREKLMAKVDVVLEKKKRDNGNQHIAAEYNKILVETLQENVALNTFAQEIQREYSFRLFVIVAFMAASLCGLGFKVYTSPMTSIGYIFWAIGKIQEILAIGTMGSTIVTITNQISSMYYESNWELVVFQSEDSKSNARLMKLVQLAIATNSKPFCLTGLNFFTISTTTALAILQGAGSYFTCLTSFR

>MdOr68CTE

MLYTPRLVNGRPVALTWPMTFYRRFNIICWPLEDNAVWWTHIFTTVIYMVSFLIFVMHNDAEVRYLRVNFHNLDDMLTGIPTYFVLIEIHIRAFTSAFEKKSFKWMLRKFYAEIFIEESLRPDIHAGNLRSYYPVLAFSILYLCALLSYIVFTIYGLAVGEKPLPYKMIPPFDYNSWYIYTPLVLSSLWVGFIVASTIVGESYALTMFVHNLDGRYQMMGERLNMGVENILKFSSNDSEAIEKFHRILIATLKENIRLNKFAQEIQREFSFRIFIIFSFLAATLCVLVFKVYTSPVNSIPYVFWTIGKVQETIAFGQIGTTIISR

>MdOr69

MEFHRPLLPNGEIAPLSWEIRLFFVNVSWPMKANAKLFTRIYDKATLVLGFLFFCYQNEAEMHYVVNNINDIGLALEGMATYLILVETHLRIYNKGLYKSSFREFLNEFYAKIYMEKSYNIETYLDIQRKLLPTKMCSYAYMLTLVTYFLVPVLGFFSNAHLVPFKTIFHYDLDIWYFYLPTLCLTLWIGVAVVSQLAAESNLLATIILHLNARYLHLQSDLKELQTRLASDMKLSTDKVLGEYRREFIEIVKRNVEYNDFAQKFQNQYSFCIFVMMAFSAVLLCVLAFKAATLGMTTKNITFITWIIGKIVELLVFGTLGSQLIETTDKMSSCYYMANWEDIILKSPKTTDNIELMKLIILSIELNQKPFSLTGYNYFSVSLATVVTILQGAGSYFTFLYAFR

>MdOr70

MLDLFAKQRQCLLLMGHNFVRDKSELLKKWHNIKYVSVLLLVVSAQWPIMNYTIYYIDDLQLATASMSISYTNVLTVVKITTFLFYKWRFAALMEKLESMYHELQEEESKAILKTSNRYAIILVNIYGNSVGLTGLYFMVAPILKIVWSKIRNTELQLELPMPMRFPFDFESSPGYEVCYIYTGLVTLSVMTYAIAIDGLFISFTINLVGHLKTLQHFIQSKSFEQNDEDVHKQISFYIRYHNLILHLYQEVRQIYSPIVFGQFLITSLQVCVIVYQMVTHINTFLVFVINCTFLLSILLQLFIYSYGGEILKNESLMVGVSVQLSNWYNLKPRHRRMLWLLMLRSQRGAIIRGGFYEASLANFMTILKAALSYITLIQSIE

>MdOr71CTE

MKKAATFDDFFKLASFFYRTIGIEPYDEPGVEVKKSKSFAENFIFYSGVINLNYVLIMEIVYVAVAFIRGENILEAIMCLSYIGFVIVGESKMFFVFRKKPILSKFVKRLVEIFPQEFELQKTYNLSSYLRQSSRVTIGFALLYMILIWTYNLYAMTQYLLYEKWLGSRVVGQQLPYYTYAWWDWHDHWTYYLLYFIHAFAGYTSATGQIASDIMLCGFATQIIMHFHYISHVLTNYKVKVDEAKDKQAGRSQDITFLKDIIEYHNCLLELSEQLNSVFSLPLLLNFSASSFVICFVGFQMTIGVEPDALIKLF

>MdOr72PSE

MSNAAKFDDFFKLSRFFYTTIGVEPYNEPGVEVKKSKSFAANLIFYSGVINLNYLLSMEMVYVAVAFVRGENILEAIMCLSYIGFVIVGESKMFFVFLKKPILSEFVKRLVSIFPQEVKLQKSCNLASYLRQYSRVTIFFALLYMILIWTYNLYAITQYVLYEKLLKSRVVGQQLPYYTYNWWDWQGHWSYYLLYFMHAFAGYTSAAGQIASDILLCGFVTQIIMHFHNISHVLTNYKVKIDQAKNRQVGLSKDMAFLKDIIVYHKCLLDLSEQLASVFSLPLLLNFSASSFVICFVGFQMTIGVEPDTLVKLFLFLFSSTAQVYLICHYSQMZMDASLNVADAVYNQNWSIADVRYQKMLILMAERAQKPVQLRATTLVLISRGTMTELMQLSYKFFALLRTMYVKK

>MdOr73

MADHSNKVYFPRILDYVYFQTFLQLLTLLPWKMSKLISFEDFLSYANALNATIGLVAYEKPNTKPLKKLIFDVIFWLNFINLNLVLLGELVFVIESVNGRHEFLEMIMALSYIGFVALGSFKTCIIMQKKSHLTTYARDMNQIFPNASIAVQRELNVRKYLKYSKFFSIMFSTMCLAMLVFFNFEAITEWLIATELRGDQNAAQHLPYFMYAPWDWTGNHWSYYLLYGIQCWAGHTSVVAQFSSDLLLYAFIGQLIMHFEAITKDVSNYRLRSCTADMDFLRNIVFKHSILLELSERINDLFGLSLFVNFATSAVVMCFLGFQMSIGASFVNLLKLVLFLILMLTQGFLICHFGQLLTDASLSIAYAAFNQNWISSDVCCQKMLILITERAQKPVILKATTLVPVSRATMTQLLQISYKFFALLRTMYVQ

>MdOr74CTE

MLDSNKLLPFNSFEIFEFSLKTTFVTQSSLRSENVTFSLIMFKLKNFEDFFIYVDFIYATFGIESWARRELRAPCKKYLKTIIFYINITNMNVVMLAEILNLFLSTTDTVDIPDLLMSMSYIGFVINSSWKIYMIWKKRPLIESLICDFHDIFPTKLMLQQDYDVQVYLRKCHRKSKFMSLLFVFAIWFFNLLAILEFGISSRNFHHSRSQQELPYFMYIPWNWQNHWSYYLLYVMASMAGHTTAMGNVSNDMLLYSLISQLIMHFDFVANTMESYEIGSGSKGVAKMEGRENGNDLEFLKIFIEYHSHLLGLSDRLNDIFGLLLFVHFASATFVICLLGFLMTIGTSFLSLFKLSLFLFTMLIQSAEICSYGQMLMDSSLRVSTAVLTTQWLKTEVRCQKMLILMSKRAQRPAQLKATYFIWISQGTMNE

>MdOr75

MRQHQQRKSSKRNQNIKTMAPKATSNGIGLNKFLLQADILAKSIGLIPYDEENDKRSVRYEKLMKFIFILNMVNMNFVLFSEIMYVLLAMKNGNNFVEATMNLSYIGFVFVGDIKIISVLRKKPVLTILMKEIEDIYPKDGRAQKAYQVREYVWRFNLISLGFVIVHEILIWFYNLYIAVSYLIYEWWLQWRVVPRTLPYYFWVPWQWQGHWSYYVLYVSQNFAGHTCMSGQLANDLLLCVAATQIIMHFEFLAKRLREYRPTGRHVDDLKFLREHIKYHQAVIHLSALMNEVFGVSLLVNFISSSFVMCFLGFQMTIGVEADTLVMLFMFLFCSLVQILMICNYGQQLIIKSEEIGHAVYSQEWLNSDLRYRKMLIGIIARSQKPVILRATTFLNVSRSTMTELMQLSYKFFALLRTMYSK

>MdOr76

MEPIEARRDLFQFVRRTMYWAAMYPLHLDRRLPHYICGLGLFVECFFEMFLYLVSIQIAILYVCTIYLNYDSGDLELLVNCMIQTIIYVWTIVMKVYFRRVRPHHLEGMVDTINAEYRTRSAIGFTYVTMDQCLDMSNRWIKTYVYCCFIGTVFWLLLPIAYGDRSLPLACWYPLDYKEPVIYETIYFLQSVGQIQVAAAFSASSGFHMVLAILISGQYDSLFCSLKNILATVAIRMHSTKEELRKLYELQESTDSELNEFYCSEEITCDINMLVHINASPKQALMSSQEFRYHFRHAFAECVHHHWYILDSLKSMEKFYSPIWFFKTGEVILLMCLVAFVSVKSTTANSSFMKVVSLGQYLMLVAWELLIICYFGEIIFINSQRCGDAILRSPWYLQMREMKNDFLLFLLNSYRPFKLTAGKMYPLNVERFRGVITTAFSFLTLLQKMDERV

>MdOr77

MSIAIRPQLLNRMHKRHHVRDNIIRIESRDKRHDLFQFIRRTMYWAAMYPMSLEHLLPQRIRYLSSFIEVFYELFLHLVCIHIVILYLCTFYLNNNSGDLELLVNCMMQTIIYVWVIGMKLYFRRMNPRPLEELMKTMNLQYRTHSIKGFTYVTMEECLIMANKWIKTYVYSCFAGAVFWLIIPITYDDRSLPLSCWYPVDYKKPIIYEIIYFLQAVAQIQVAAAFSASSGLHMTLSILLSGQYDVLFCSLKNILANVALRMQSTEQQLRKLYKLHEITSHDTNEFYCSKEKTLDVERLFDAQQLFVETSQDFRHNFRNVFKECIVHHWFILDCLKSMERFYNPIWFLKTGQAILLLCLVAFVSVKSTTTNSSFLKNLSLGQYLFLVAWEFLVICYFGEMIFYNSQRCGEAILKSPWYLCMREIKSDLLLFLLRSYRPFKLTAGRMFALNIDWYRWVITTAFSFLTLLQNMDQRDVNVST

>MdOr78

MTPSVYFYGGDSDVLYSEHDSGREDDVFKLQLLFMKFMGQVPMQLERRLPLGWKNVAGMFAKSYCIFCVISNLHLAILYVKTTLDMLHNGELEEITDALTMAIIYSFSTFATCYWLFNAEALNSFIGDINANYRHHSMAGLTFVSAEHSIRLAYKVTLYWLIACCVGVVCWALAPLLLRSHTLPLRCWYPFDALKPVVYEVVYATQLWCQILMGCIFGNGSALFVSVVLIMLGQFDVLYCSLKNVDYNAQLLAGGDLITLRNLQRDLPRPADDELNQYALLEEHLTDLTALRVSKPNSRPSLKEALHSSLVECVLLHQFILKSCNTLEGLFNPYCLIKSLQITLQLCLLAFVGVAGERSTMRTINLVQYLALTLSELLMFTYCGELLSSHSIRVGEAFWRSGWWLNGNLIKRDIFIFLANSKRVVVVTAGKFYRMDVQRLRSVITQAFSFLTLLQKLAEKNQ

>MdOr79

MEKHRLYTLDEFLLKLQPSQRYTRIIYLDFRRENQNKPFRFESLRLLYAALTLLIVDCACNVLKIIFEIRAQRLSEAKQIGAVWSIAFLCLIRGIFVMFKHKSMLDLTNDLDKIFPRTRLLQNRMNCHKLARYLLIRHRFLFAYAVVGLSAFIGIPLLKYIVFYDPNSGEPLLDEYHQHASWFPFHLKENPTTYPYMYVSETILTLFGINCLFTWDHIYTVTVAQFIMHFEYVNTELARLNAKDTMDVEKSKKFYDDLVEIIKYHQHVLRLGNKLRNTFNLPLFLTDLISGASICFHIYLIANTDDVIAITLFIFPCFVQVAFAFDNCYQGSRIENVTTNMSQVIFEQNWYDATLEYRKFVVHFLLFASRPFTLCGYNLFSIDMVHFRGTMMIAYRMFTFLQARGSKVE

>MdOr80

MNVRLHNDGGYDRTYAVRGILRVMKILGLWKWQTEADKETPRHILWLQYVQRLVCHGPFTFVFITLMWIEALRANGLDEMGDVLYMSLTEAALIVKILNIWQHSTKASTFLHALRHNAHFALHSGDEVTFWRNAQKKFRYIIYMYSAGSVFTVISAFAGVLFVTEPQMAFAYWVPFEWQSNRRNYWLAYLYDFVSMVCTAGSNVCLDMMGCYMMFHVSLLYKVLSFRLQKLRAVKGEDVNEKFKKLILMHKSIRRMTRECEILSSKYVLSQIILSALILCFCCYRIIKLDIVANFGQFLSMLQFLAVMIFEIFLPCYFGNEITLNSSEIMLDVYRTDWLEYSVANRKLIILFREFLKRPDKVTIGGYFEVGLPIFTKVVNNAYSFFALLMNVEK

>MdOr81

MQLRQPKDVGQQLNSVYGLKYLWWNFSIIGIHPPAGVRTHPVWRFLYLVYAVVINFLAGFCLPATMLANLMLLKSLEEIIGNLSLSMTIAISMTKELAILYCRGGLLKANHYLRLLDERCSAHPRDRMTVMEAVRMCHWYYTVYISFYGFCAIGFAYIGWSNHTLVYSAWFPNIFANDQTNYLAAYIFQNLAQTFTVFQNGNNDMYPLCYITLMIYHVRALADRIQRVGGDAETSAEENVQELRNCIQDHKNVQSYFECIQPAISSTMFQQLWVAAFTLCLTAINLMAFERTFAEKIFSVVYLGVIVIQIFPACLCVNFMMSETSNLTTAMYKCNWIEQNRNFRRMLIIFMQRSQKVNVIYAGGLAPVTLQTFVAIIKFSFSMYTILSQMKIQ

>MdOr82

MVLETDNSLILFDFIRLPLKFYSAVGIKIFQWDADDIMTTKEKCIFLLLGINFIGCFLAKSLFCVFGEFVDTMQATQWILYFMFAMNGCCKTISVAIGRKKLYTVLKDIEGIFPATLKERQEFRLAHNYGYIMRHAKIMSIQHCSIAIMFIAFPLVQSTIEYLTSADSEFVTRTPYIMVYPFDATAGIGYVVGYFSQFLGGFTVSCYFVGSDMLLMCTIYLVIMQYDYICYRIENFKSRNYEEDMKELKIVLERHNLLNIVAETVNEVFSISILLNYMISILIIVMISIQITKGSEFGLDMIKFVGFFTSASTQVYYICMFGNLLMDYSSRVSESLIGQEWYWTDVRYQRMLVLAIARSQRPSHLTAFKFFTISMESYGNLMTTAYQFFTLLRTTYNNN

>MdOr83

MYYNHPLFSFNVKMWKYLGFIEFKRINQALLILIIPCLINMCQVMNIAYNWNDMSVIAIGLFMTAILFNALVRITTVMRNQSKFIEFFEMIEQWYREIEMGPDDGAWDLLKHIPRRTRLISILSFSFAAGAAVASATIPLFLEQRSLPYDMYIPFYDHLKSPMYEILYFMQGFISMPFCVLTYVPFTNLFIAWLTFGISLLQILRYKLESLPHENDEEMLKQLIELIRFHHRIMNFGQTLESLVSFVCLVELVLFTLMLCVLLASFLVMDNVMSKIATCIYIFCILYALFIPYWHANEFSWESTKIADAAYNIKWTRSNIKIRKCIAMLILRSQTPLKIKAGGIFPMTLEAFQALLNTTYTYFTMFKGMMGKEPNVHDRGQ

>MdOr84A

MTMEPRNFSKYLQITITLNQSISIVLQLMYNFTTQDEDVDVLTNMIYFNYIFVGLGKLLCMYYRRQTLAKVLETLQEIYPTQHIEEKYNLNKHFRYYSRIEKFIWSFYRLVGPVYVALPLLQSLKNIWTLGKFTLLLPLCLWKMGDPMDSNWWLTYLFYYLIGGSSSIFSGLTITGCDLCLYSLITQMCMHYDLLSQRILELQPASGEEIASKKLRGLTQQHWMITNVANEINIFSVMSSSFTLCLVAYQMLDDVSIFTIVKAFILLLYESKQVIITCYIGQKLKECSSLVNASLYAHSWYDGSTRYRRRVLYMLLCTMQPFVLNFMGIADITVITLKEVYGNAYRLFTVFKSA

>MdOr84B

MTIEPRKFSKYLKITITLNQILIIVLQIIYNLTTQDEGVDVLTNIIYINYNVVALGKLLSMYYRRQTLAKVLEILDGIYPTQRIEEKYNLNSYFRYYSRIETFIWSFYRLVGPVYVTLPLVQSLKSIWTLGKFTLILPLSLWKMGDPLDNDWWLTYLFYYLIGAFSSISSGMTITGCDLCLYSLITQLCMHYDLLSQRIMELQPAAGEENATKRLGILTRQHLIVTNVANEINIFSVMSSSFTLCLVAYQMLDDVSIFTIVKAFILLLYESKQVIITCYIGQKLKECSSLVNASLYAHSWYDGSTRYRRRVLYMLLCTMQPFVLNFMGIADITVITLKEVYGNAYRLFTVFKSA

**The Gustatory Receptor (GR) family**

The gustatory receptor (GR) family of seven-transmembrane proteins in insects mediates most of insect gustation (e.g. [16, 17]), as well as some aspects of olfaction, for example, the carbon dioxide receptors in flies [18-20]. In *D. melanogaster* the family consists of 60 genes encoding 68 proteins through alternative splicing of some genes [24]. The GR family is more ancient than the OR family, which was clearly derived from within it, and unlike the OR family is found in the crustacean *Daphnia* *pulex* [28], the tick *Ixodes scapularis* (HMR, unpublished), and many other animals (HMR, unpublished). This evolutionary history is reminiscent of the ionotropic receptors (IRs) [21, 22].

The MdGr gene set consists of 76 models, encoding 100 potential proteins through alternative splicing of seven loci. Eleven (10%) of these are apparent pseudogenes, four gene models required repair of the assembly, and four were joined across scaffolds. As is the case for some *Drosophila* GRs, as well as those of several other insects such as mosquitoes and Tribolium, at least seven genes appear to have an unusual form of alternative splicing in which multiple alternative long first exons are spliced into a shared set of C-terminal exons downstream of the last long first exon in these tandem arrays. The resultant proteins differ considerably in most of their sequence, and hence presumably bind different ligands. They are indicated with a lower case letter after the gene name. As a result, the number of apparently intact GR proteins is 89.

The automated gene modeling performed by the NCBI using GNOMON had access to all available insect GRs in GenBank for comparative information. Given the relative closeness to *Drosophila*, automated gene modeling might be expected to be successful for conserved proteins like the carbon dioxide receptors, with perhaps less success for some of the more highly divergent bitter taste receptors, and indeed succeeded in building at least partial gene models for most genes, with 20 precisely correct. All others required at least one change, while 16 new gene models were generated (not including pseudogenes or those requiring repair of the assembly) (Supplementary Table 7). Most of the new models are indeed candidate bitter taste receptors.

As expected from its relatively close relationship, the GR repertoire largely resembles that of *D. melanogaster*, however as expected from the birth-and-death model of evolution these large environmentally relevant gene families experience, there is considerable gene gain and loss, as well as some interesting complementary evolutionary history. Overall the *M. domestica* GR family shows an expansion compared to the 60 *Drosophila* genes encoding 68 proteins, to 76 genes encoding 100 proteins, and most of this expansion is in lineages implicated in perception of bitter tastants. Approximately equal numbers of gene lineages have been lost from each species, again mostly candidate bitter receptors. Several instances are noted where a tandem array of genes in one species has been independently achieved in the other via alternative splicing, remarkably complementary ways of expanding the sensory repertoire. The *D. melanogaster* receptors were named for their chromosomal locations, which is obviously not relevant for *M. domestica*, plus the extensive gene family evolution largely precludes naming them for their *Drosophila* orthologs, hence a numbering system is employed starting with the conserved carbon dioxide and sugar receptors. Detailed accounts of the major GR subfamilies and lineages are provided below.

The carbon dioxide receptors are known to be highly conserved within most of the holometabolous insects, except the Hymenoptera to date, with two proteins represented by DmGr21a/AgGr22 and DmGr63a/AgGr24 constituting the functional receptor (e.g. [29]). *Drosophila* species have, however, lost a third member of this subfamily, first recognized as AgGr23, which is present in Tribolium, Bombyx, mosquitoes, and tsetse flies, and known in those species as Gr2 [29]. This gene is an ancient paralog of the DmGr21 or Gr1 lineage. *M. domestica* does not have this Gr2 lineage, so it appears to have been lost before the *M. domestica*/*Drosophila* split. The importance of this protein is debated, with Lu et al. [20] finding that it enhanced perception of carbon dioxide, while Erdelyan et al. [30] found it did not. Unusually, *M. domestica* has a recent duplication of the Gr1 lineage (DmGr21a), and in a probably futile effort to maintain the naming convention proposed by Robertson and Kent [29], these are called MdGr1.1 and 1.2, while the DmGr63a ortholog is called MdGr3 (Supplementary Table 7 and Supplementary Figure 6). The only other known recent duplication of a carbon dioxide receptor gene is the Gr2 lineage in tsetse fly.

The sugar receptor subfamily is a larger set of 8 genes in *D. melanogaster* (e.g. [31, 32]), and study of these in other available insect genomes indicates that they represent four major lineages that duplicated in basal Diptera [33]. One lineage, represented by AgGr16, was lost from *Drosophila* and *M. domestica* does not have it either, so this might be an old loss. The other three lineages are each represented by 2 or 3 paralogs, specifically DmGr61a and 64a, Gr64b/c/d, and 64e/f and 5a, all of which are proposed to have once been in a large tandem array, with the terminal 61a and 5a genes moving from that array. The *M. domestica* orthologs for 61a (MdGr4) and 5a (MdGr5) similarly appear not to be in the array (confirmed for MdGr4, which is in a 38kb scaffold that contains other genes microsyntenic with DmGr61a, but only suspected for MdGr5, which is in a 38kb scaffold with no flanking genes), so their movement out of the array is old. The genome assembly for the remaining genes in the array is rather fragmented, however it was possible to connect most of them in an array, albeit now with the DmGr64a/b/c/d orthologs (MdGr6/7/8/9) in inverted orientation to the DmGr64e/f orthologs (MdGr10/11) (Supplementary Table 7). The DmGr64a ortholog (MdGr6) is only represented by the final exon in the assembly, however it was partially manually built from raw reads, and unfortunately the MdGr7/8/9 genes are only represented by the first 4-5 exons each encoding the N-terminal half of the protein, hence their phylogenetic relationships in the tree are not accurately resolved (Supplementary Figure 6).

The highly conserved DmGr43a lineage has recently been shown to be a fructose receptor [34] that also serves as a nutrient receptor in the brain [35]. *M. domestica* has a duplication of this lineage (MdGr12/13) (Supplementary Figure 6). Duplications of this lineage in other available insect genomes are not common, however the hessian fly *Mayetiola destructor* and the silkmoth *Bombyx mori* each have duplicated it, and *Tribolium castaneum* has 10 paralogs.

Most of the remaining *Drosophila* GRs are implicated in perception of bitter tastants or have not yet been functionally characterized. A naming system for the MdGr orthologs and duplicates is not obvious, so they are named consecutively, but starting with some of the best known and most conserved ones, and keeping sets in tandem arrays or phylogenetic clusters in consecutive number series. There are quite a few interesting differential evolutionary paths these gene lineages have taken. Some have simple orthologs, for example, DmGr2a/MdGr16, DmGr10a/MdGr42, DmGr33a/MdGr38, DmGr47b/MdGr65, DmGr57a/MdGr66, DmGr58c/MdGr67, DmGr59f/MdGr71, DmGr77a/MdGr72, DmGr89a/MdGr73, DmGr93a/MdGr74. The highly conserved DmGr66a protein required for detection of caffeine and many other bitter tastants (e.g. [36]), also has a simple conserved ortholog (MdGr36), however there is also an older duplicate of this gene in *M. domestica* (MdGr37) that was lost from *Drosophila*, and presumably was also involved in detection of bitter tastants. Other examples that illuminate evolution in *Drosophila* are DmGr2a/MdGr16, which are simple orthologs, but then MdGr17 is an adjacent gene with no simple *Drosophila* ortholog, and MdGr18 is the DmGr23a ortholog, indicating that DmGr2a and 23a were once in a small tandem array.

A far more complicated scenario is offered by the set of MdGr42-64 genes, all of which are in two large arrays about 100 kb apart in the same scaffold. The first gene, MdGr42, is the simple ortholog of DmGr10a, MdGr43 has no *Drosophila* ortholog, then the apparently alternatively spliced MdGr44/45 are related to DmGr59a/b, while Md46/47 and 49/50 and 53-64 are a large *M. domestica*-specific grouping with no *Drosophila* ortholog, and finally MdGr48/51 and 52a-k are related to the DmGr36a/b/c and 59c/d genes. It was apparent from analysis of the *Drosophila* Grs that their dispersion across the genome in mostly singletons and a few small tandem arrays might be a derived state [24], and indeed most other insects have multiple examples of large tandem arrays of chemoreceptors that provide a simple explanation for their origins through unequal crossing over. This and other examples confirm that many of the *Drosophila* Gr genes also originated in large tandem arrays, which have subsequently been split up by the high levels of chromosomal rearrangement seen in this genus.

DmGr32a is a particularly interesting candidate bitter taste receptor (e.g. [37]), that is also involved in courtship through expression in a small set of gustatory receptor neurons on the male foreleg [38], and recently was implicated in mediating rejection of non-conspecific females as targets of male courtship [39]. While it has a simple ortholog in *M. domestica* (MdGr14) that might play a similar role in species recognition in *M. domestica*, the ortholog of the related *Drosophila* gene DmGr68a appears to have been lost. Furthermore, the related alternatively-spliced DmGr39a gene has a similarly alternatively-spliced *M. domestica* ortholog MdGr15 (middle of Supplementary Figure 6).

There are some additional interesting examples of gene subfamily evolution. For example, MdGr75 is an apparently alternatively-spliced gene with two protein products, but its ortholog in *Drosophila* was duplicated into DmGr94a and 97a (top of Supplementary Figure 6). An even more extreme example is provided by the DmGr39a-c and 59c/d genes, whose expanded relatives in *M. domestica* include the genes Gr51 and 54 and the alternatively spliced Gr55 in a separate tandem array noted above. There is one other possibly alternatively-spliced locus, but for now they are included instead as missing their C-termini; these are the set of MdGr22-26 (top of Supplementary Figure 6). These genes are in sets of otherwise fine tandemly-oriented genes (Supplementary Table 7), so it is likely that their C-terminal exons are simply missing from the genome assembly. For them to be alternatively spliced would require more complicated models than those for all other insect GR loci that are alternatively spliced, in that there are multiple exons before the potential alternative splice, instead of one first exon each.

The DmGr28b alternatively-spliced locus is another interesting problem. The various splice forms of this gene are expressed in both gustatory cells and in the brain and elsewhere [40]. In *M. domestica*, this locus is split across two different scaffolds (with additional assembly problems probably involving multiple haplotypes). It encodes seven proteins compared with the five in *Drosophila*, because two adjacent first long exons have been duplicated in the *M. domestica* lineage (39c/d and f/g) (middle Supplementary Figure 6).

Finally, there are 11 DmGr lineages totaling 18 proteins with no apparent MdGr orthologs (Grs 9a, 10b, 22a-f, 23aB, 39b, 59c, 68a, 77a, 89a, 93b/c/d, and 98a). Similarly, there are 9 MdGr lineages totaling 26 proteins with no apparent *Drosophila* orthologs (Grs 17/19, 20/21, 29a-c, 37, 41, 69, 76, and the complicated subfamily of Grs 43, 46/47, 49/50, and 53-64 described above) (Supplementary Figure 6). It is possible that some of these genes have simply diverged too much for phylogenetic analysis to reveal their relationships, and indeed for at least one pair of the above (DmGr39b/MdGr19), microsynteny analysis suggests that they are in fact orthologs despite not clustering together in the tree (Supplementary Figure 6 top). Presumably the orthologs of most of these genes or gene lineages were lost from the other species, and eventually identification of their ligands, along with those of the duplicated genes in each lineage, will provide insight into how the gustatory capabilities of these two flies have diverged.

**100 MdGr proteins in FASTA format.**

>MdGr1.1

MAFWATVNSGNPSTPKIVPVLNPNQRQFLQDEITYQNKIKFLAENDGANLTDFYVRKEEVFDDPELLDKHDSFYHNTKSLLVLFQIMGVMPLHRNPPIQGIPRTGYSWISKQFFWALFVYTVQTCVVVMVLRERVIHFKEGPDKRFDQAIYNVIFISLLFTNFLLPVASWRHGPQVAIFKNMWTNYQLKFFKVTGTPIVFPNLYPLTWGLCIFSWVLSILINLSQYFLQPDFKFWYTFAYYPLIAMLNCFCSLWYINCTAFGIASKALSESLRKTLRGEKPAEKLSEYRYLWVDLSHMMQQLGRAYSNMYGMYCLVVFFTTIIATYGSFSEILDHGATYKEVGLFVIVFYCMSLLYIICNEAHHASQKVGFDFQTQLLNINLTAVDTATQREVEMFLVAIAKNPPTMNLDGYATINRELITSNVSVMATYLVVLLQFKITEQRGLRTQQAAIS

>MdGr1.2

MAFWATVASREVASPRVMPALTPSQKQFLHDELRYREKLNFLADNDDVNLSDYYVPKEETVDDPELLDKHDSFYHTTKSLLVLFQIMGVMPIHRNPPKPNLPRTGYSWTSKQVLWAMFVYVIQTTVVIFVLQERVNKFVTNSETRFDEAIYNVIFISLLFTNFLLPVASWRHGPQVAIFKNMWTNYQLKFLKVTGTPIVFPNLYPLTWGLCIFSWTLSILINLSQYFLQPDFEFWYTFAYYPLIAMLNCFCSLWYINCNAFGTASRALSESLQKTLRSEKPAQKLTEYRYLWVDLSHMMQQLGRAYSNMYGMYCLVVFFTTIIATYGSLSEIIDHGATYKEVGLFVIVFYCMSLLYIICNEAHYASQRVGLDFQTQLLNVNLTAVDSATQKEVEMFLVAISKNPPIMNLDGYANINRELITSNVSFMATYLVVLLQFKITEQRGLRSQQAIAMDP

>MdGr3

MASNYTRKKKKDAVFLNVKPIMNGDISVRKYSNGIMDQMHNGFRKQVYERANIRPSLATISSTNQQFIPNVFYQNVAPIKWFLSVLGVLPIIRSGPGTTRFVARSLPFVYCVVIFICLSAYVAYVTNQRIMIVTSLSGPFEEAVIAYLFLVNILPIFTVPIMWWETRKVCTLFNDWDDFEILYYQISGHSVPLNLRRRAQNIVLVLPILSILSVIVTHITMADFSFIQVIPYCILDNLTAMLGAWWYLICEALSRTAYILAERFQKALRHIGPAAMVADHRALWLRLSKLTRDTGTATCYTFTFLNLYLFFIITLSIYGLMSQLSEGFGIKDIGLAITALWNICLLFFICDQAHNASLYVRTNFQKKLLMVELNWMNSDAQTEINMFLRATEMNPSNINCGGFFDVNRNLFKGLLTTMVTYLVVLLQFQISIPNVIQGINSNMTLIEAITMMITDSDYSGESEEATTTTTTALPKTTKIISTGTRGRKG

>MdGr4JFI

MPLSKYHWKVWTNLKLRKREQKQILNKFAQLHHRQDFGNLDTFHRAMRPGLLLAQIFGLMPLVNSMGCNPYRLAFKIPCLTFTTTVLFLFFGSWKTLHVSDSLLKVGLNPKNIFFNAAFAVTWNFMDFFIMAVSLGIATRFQQFAERIELLEGNYVPDALWNQIRQHHILLCEFMEKVNEHLSAIVLLSSINNMYFICNQLLNIFTKLRYPISTVYFWMSLAFLLGRTCGVFMFASRIRDASLLPLKTLYLVPSGCWTEEVQRFLAQILDEPLGLTGKYFYTVTRQGFFGMMSTIVTYEFMLLQLDAKSREGDLPDLCT

>MdGr5NI

LVMAQCFCLMPVRGVLSKSVKGLSFRWLSFRTSYCLVYMALTVADSLLTLNLVRRAELDVRNIEPMVFHTTIFLASIGFLRLASKWPKLMRRWQQVERQLPAYRSWQERGELAKRIKTVTFVLITMSLTEHLLSTISAIHFANYCPATSDPIESFFLTVVDQVFLVFNYSPWLAWLGKIENILLTFGWTYMDVFVLIIGIGLSSMFKRIKRQMEQHKGQAMPESFWCEIRRQYMLICDLIEEVDEAVSGIIMLSFANNLYFVCIQCLKSINAYHVEVERFAMEINSMSVTMTGLRYFDITRKLVLTVAGTIVTYELVLIQYHEDQKLWYCGNE

>MdGr6FI

VLFLGQCFSILPVRGIRRSNPKQLRLKSIQVLITLFFMCCSSILTLTTLKHLLKIGINAKNFVGLAFFGCVQCSCVLFALLAPHWPRLMRYWSFNNYILKNYDYVFQILPHNMFIGVFILNGLCTFIWNYMDMFIMMISKGIAYRFEQITTRIEEVPETVFIEIREHYVKLCELLDCVDEDLSGIILLSCINNLYGRTAFVFLSAASINDESKGGLAVLRRVSSRTWCFQMTTQTVALSGKKFYFLTRRLLFGMAGTIVTYELVLLQFDEPNRAKGLPDLCG

>MdGr7CTE

MEQQTFHQSVRKILFISQCFGLLPVSNLWQKNVNKLKFKWVSIPSIYSGVILVLDIMEFGVVIYYIWQTGVNFHTSGTVSLFFVCIWEHIIFWRLALKWPKLMRQWRQVEELFLQVPYQLYVTFNMKFWIWFWYLLIMFGGSCEHVLLVFNSFQKSDLERRQCNLNVSYWETLYGRERPHLSMVIPFQYWTLPIYEWLNLTLAYPRSFTDVFIIIISIGLAARFHQLHLRMKAVQGK

>MdGr8CTE

MFNYTLDETIRNTLLFISQIFGLFPISNVYHGSISRLRYKWLSLPVAYASAIMILNVLEFVVVIYYNFVTGINFHSLGTIALFLVCLLEHYFFWRLSSKWPKLMKQWRQAEEVFFRAPYPNYLTFNMKFWLWLWYTVIMCGGLMEHCLLVFNSIQKADLERTQCNLNVSNWEILYGRGRPYLRLVIPFQYWMLPLLEWLDLTLAYPRNFTDAFIALVSVGLATRFRQIYLRIRHVQGK

>MdGr9CTE

MVQVKVQSYEEQSTRSITNTLHHALGPFLVLSRFFGTMPVLGVWPRADIALVRFKWCSLPVLVTLTLCLFATMDLFLSLKVVTEMGIMLTTTGPLSFSIGCLTGFIVFLWLSRKWPNLIKSTRRLEVIFLRGPYAACPESQMLSRRIRLTGTLFLVSSVVEHLCYVGSGIYSNHLQIKECNLTAGFWKNYYMRERWQFFSLIDYTVWLVPLLQWITISMTFIWNFVDIFLILVSQSLAVRFNQFKWHVQCHQKKHMSNDFWLGVRKDFLALTDLLWLYDTDLSGLVMLSCAQNLYFLAVQTFHVFLYRDNFMSEIYFWFSLLHVAIRTFYMMWSAAAINETAYGILSTIYEIPTAYWCLE

>MdGr10JIN

SIFFHMKSANGKQSQRKSLMMKLKHQILRRGRKEDYMHVGSFQEAIRPVLLMAQIFALMPVEGITSNSSDDLRFSWTSVRTWYSFIATVLIGICSAFNIAYAFRGVFNFDSVEHILSMLTIIYYVNRCPRFQNQPLNSFLFTNFSQFFYFFEYTTFAGICGKVINILSTFAWSFNDVFVMCLCVSMTAKFRQLNDYMAKYSKKPTTRSFWIERRKTYRMLCHLCEAVDDTIAVATLLCLTNNLYFICNKILKSLQKKPSIAHTLYFWYSLIFVIFRTFLFALFAAAVHDESKRPLVIFRNVKREYWCSELKRFSEEVNADCTALSGMKFFHLTRSMVLSVAGTILTYELVLLQLTKTEVVSDCH

>MdGr11

MKLPVTRPQALRMQIISDSDHYHSYFTSRTDVPNNEEYLEKPTKFLQKATKDNFMYEGHFHEAVGKILLIAQCFAMMPVRGVTSSHPRYLSFSWTHIRTIYCLIFITCSAVDSIIAVYKVLNAPITFNTIEPMIFRIAILIVCVSALNLARKWPELMVQWHSLEQQLPEYSSQKEKRRMADKIRMVFFVGMMLSLAEHLLSVTQAIYFAARCGATDDPVKNFLLIASDHLFYIFPYSYLLGWYGKLLNVMSTFIWNYMDVFVMIMSIGLTYMFKRVNENLEKFKNKQMPAVFWAERRVQYRNVCILCEKVDNAISMITMVSFSNNLYFICVQLLKSRNNMSPAVSMVYFYFSLVFLFLRSLAVSLYSAAIYDESRKPLRVLRSVPKESWCLEVKRFASEISSDLVALSGMKFFHLTRKLVLSVAGTIVTYELVLIQFYEPTDLWDCKSLLKNFEHQKLLASGK

>MdGr12

MEISESSRCIYIVSKILGLAPFSVKKSDKGTYLVEKSIPFIIYASVLTSAMSFLTYRGLLFDATSKIPLRQSFRMKSVTSKAVTTMDVSVIVMAVTAGALCGIFGYNPTKELNVRLQKVDASINGDRKRDSLKAIMLLILPVISITILMFFDIWTWLSFAQTANTEGENTDLNALWYIPFYGLYYILICLHVTFANTTLSLSRRFKTLNITLIMSFLTTESKKEIQMQNIPKITPVLPTKGHEPPLHISFTKITSELHQAPEKNKSLLLKMLAECHESLGKCVELVSSSYGMAVLFILLSCFLHLVATSYFLFMEFLEKNSGGFSWLQVMWITFHTSRLLLVVEPCHRISAESSKTIHIICEIERGIHDSILAEEVNKFWQQQLVFKDRFSACGLVIVDRSLLTSIFSAIATYLVIIIQFQKSDG

>MdGr13

MEISEPSISILFLSKVFILAPYSLQRNAKGIYIIDKSVPFIFYSSSIILLLVFLTYRGLLYDANSNVPIRMKTATSKIVTALDVSVVVLASVAGVFCGIVGLNTTRELNGRLRKVDETINGFKDVKRERTKALILLIVPMLSITILLGLDIGTWLRKAASMKVHEDDETDMNIKWYIPFYSLYLVLTVLHISFANTTFGLWKRFKGLNRLLRTSFLPHVRVKEPQMTKNPKITTVKANSTVSSSSSLASESYQQNGKTKSLLLKLMAETHESLGKCVKLVSSYYGMAILIILVSCFLHLLATAYFLLIELFSNKDSGYVWLQVMWIIFHALRLLAVVEPCHRLTVESTQTIHIICEIERTIHDSILAEEVKKFWQQLLVYEPRFSACGLCMVDRNILAAMFSGIATYLVILIQFQKTNG

>MdGr14

MCPGPLSVSMKGSKIKQSPTLQRENDEIVLDDSMGSSPKSKTFLNDITSILVILKATGLMPLYVTLTAYELGPPKILNRIYSIAIHFMVHAMTIFNMYMLFTGGSNQLFYSYRETDNINYWIEILLCIVTYTTTVVVCSKNSKAFLKILNETLKVDEEIQQQFSATIVNDCGFAVKFIILILIFQWYIVLLKILLINEPLTVTSYVIISVYSIQNALSSIFIVYSSILLRLLSVRFAYLNSIINGYTYKEQQKTRRFRTRIPTKDQATLPPPMSSFPEDSLFAFRMYNKLLRLYKSVNESCSLILVVYMGYSFYSITTTTYNLFVQITTQLEMSLNILQICFALLFSHTAMLALLSRCSGEATDQANLTSQILARVYEKSKEYQNIVDKFLTKSIKQEMQFTAYGFFVIDNSTLFKIFSAVTTYLVILIQFKQLEESKLDDSGGQTTTTTPALAPTAAMNETIQ

>MdGr15a

MSEEFEVFYKLLRLSGLTAVPFDGSKSCEKVRNCLIYYFFPMGIQLTLVSSVVLAYLIRESLLLADFMATEYYYNYILVESTFVTNIILRFWLISNQNINLQILELCKRWITSHCHATVHSKKMLAAFFVAALVYFANLLVLFYELWFNGVISVKLCLFWTLFTYCYVTTVLILCLWCAIVIAISNVFKSIAKQLEDILLHADVMFPDTDIVLLQALVHTIGEIIQVVSKDVSKVHGISLLLCMVVTINESIWNFFQMMAPNLASNHLIEFLMSMWMLPILILLAIGLPNNNVQEEANKTAKILARYSRSNTGADKMIDKFLLKNLRHKPILTAYGFFSLDKSTLFKLFTTVFTYMVVLVQFKELESSTKTLH

>MdGr15b

MTDKYICVFYKLLKYSGLMAVQFDADNLCYFIRGSIGYYVFHTGIQLALVASFVATYLNRGYILVGDFIDTEHYYNYLSMQTTFLSHTVLRLWLICNQHNNLRLLESCRKKWWNGMDDSTVDGGIFDDYTRNLLMAFAVSAVIYFVNLIIMLSLNSDGLNGSSLLIWTGFTYCWLTITLILYVYIFIVITISRVLKSMAHRCEMMMLHRTIDFNNCTDLRHLQYLFNLYDDITYAVWQDVNCVYGIAILFSTITLINESIWDVYELAMSNSETNYFNQLQTTMWMVPICIFFIVGLWNSNVPEEANKTAKILARYSRSNTGADKMIDKFLLKNLRHKPILTAYGFFSLDKSTLFKLFTTVFTYMVVLVQFKELESSTKTLH

>MdGr15c

MFGDLDLKSFIGTLNVLGLLSCCFTNPDSGSHIQRTLAHKVRSFFAMALMQTTCGLLFLYWLLFPEQFDFESYNSTGNIYVTLNYVSGSAVISVIYLYFFICQTCLLQTIESVLSYQQTFLQFHCKGWNLRHWFGVYILLAITNFVNNYRVFSKIKVGHVAGPCYQFMNNLIFLLFGIILLTYVSVIKIVESCMQHINDDICRMMSAEKESHGESFDLIELMAKRKKLIDLCERELGERFGPVFLVIVTFMVFSAPSGPFYFISIITSMRFDSIWVLAVGAAGTMYWILPWLVIFVAVMSCAFDDQANKTAKILARYSRSNTGADKMIDKFLLKNLRHKPILTAYGFFSLDKSTLFKLFTTVFTYMVVLVQFKELESSTKTLH

>MdGr15d

MFGDLDLKPFLWTLNLYGLFNCDFVDSYDEDGYFRRTLHNRVHSVAVLLLVQALCAFLFLYWLLFRKQFDIDAYNSTGNIYLNIYYAFGCVLVSVIYFYFFTGQLCFMQTLDTVLRYQEEFSQYRCSNWNLRHWFWIYVFLATTEILNNYRAFESTNVATLANICFQLMSNLVFLLCGIIILLYVAVIKIVKSCLRHVNKEIHRLLLGKKSKGKNRNLKEWMESRKKLLDFCQNELSERFGIILLVILAFMVFSAPSGPFYFISVTLKLGFEYNWAFVCNAIMVFYWSVPWVIVFIAVMSCTVEEQANKTAKILARYSRSNTGADKMIDKFLLKNLRHKPILTAYGFFSLDKSTLFKLFTTVFTYMVVLVQFKELESSTKTLH

>MdGr16

MEIMDSLIVFQIIYQFTNLTPWSINRKGWIFQRSRILEAYCVAVILVSVVVLLYGLFSKNAITTINSNDIGKTVDFIQLVGIRVAHIVSIAEALIRREEQKKFYQQLIEIDKIFEKSLNIDLNNGKFHSSTAKSGLLILCVYIISEVFILIAHLISYENENFQIYWIFYLVPLLICGLRYFQTFTSIRLIQKRLNELIKLLNEINLHKPLLELSLHKRQEMENTDMKKLLIVRDLYNRLFLLTEIFNRYFGVSMLINLGNDFISITSNCYWIFINFKTFASTTKNFLQIAGSTVWFIPHVLNVLVLAILCDKTMGCTTNMALGLHRIHIDTFNDNHNSVIQQFSLQLLHQKIIITAAGFFTIDCSLLYAIVGATTTYLIILIQFHLNEELDS

>MdGr17

MDEDLKFVLNCCTAFGIYIPQTKYGSRRWKIGCTIYTSFLMVILSSICVLGVFMSPLENDYIISWFVSAFVFVSQIFSHLVMMWECLAKQREHTEFLRLLDEIEVAFKLKLRTDIGRDLLAQKLRRILFSLAAISILGLIIFGIHTSLMDDQGYFWWALFAILAMRMRFLQLQMYVELLNHYLWSLNRKLQQVVCLKTEEEAQLLDVDYKQLETLEYLNHIKELYSSIYEAFHCLNEFGQASMFAVTASYFLDCTCHIYWCLLALDKLFPSASIVLSISTIIPLSLNSYKFCYTCQLVKQECRLTALLVTRLNVSDSNHNCLELQKNYKSLVHDFSLQLLHQRIVVTGKRFFNFDLQCIFGICVLIVTHLIILIQFTKSDNNSGNVNQTEIQETMVN

>MdGr18

METLGSIVRAALMFLILICGLYPITRFPWVTLLLRIGLLVWLFVNVFLMFYLRMNGRDTSIGGLVGTASFVCNGITNIIIILESMLHDNHAKIMHQLEDEIFYIFKRHFHKRIEELEKLRHKMSKEILAIFCIELICIGFKLWINAISSIQPVFWQAFPTSVSLRVRYIQIIAIVIKFNGHGEIFKHYLKLSTTDKTPSNAVGLWQPYKDEEYAQLNARRLIYLRIWEMFKSLNDAFGWSILYLFITSFFDIVCNCYWTFTAGYKGQVFHKYVFNGATSISLSSLVITLFYYTDTSYKNSRYIGCLISKLVKQPLGNKRYNDLVSEFSVQTLHQRFIVTAKEFFALNLGLLGSMVAAIVTYLVILIQFMFTEKSNGDSKISSSKLETTTIANTLLFTTSAVNSTILDMFENNN

>MdGr19FIX

MVNELHLNFLKFFVIFGLAPYTRRRQNQQRRRRRQCQCRSQCQHLSHTYPHNVNYHNKQHYFVHHHHDDNDDVLRGGPNHHLRWQQIYTGALIILNLLLTLYGVVVMPFEDKTVISDLVSVIVFVIQMAVIFVVLIETALSYGEHYRFIENIHRIQSLMQRLLQTQLCSVTLRQRQRRKYFIFIAVVYGSLLLVMLVIFFVHYYGYFWHAILAVLIIRTRCLQMLVALDYVCFYLELMNRKLQALISCKNSQNYHCLDVNYEHLESYEYLENFKLIYDEIYILHSIYNRIFGVSLVGILTVIVLDIIIHVYWSLLTIMGYYESYFIAITGATLLPLSTIFVVLCATGDQCEKECNSILISLKSLLRTSSKSFNPHAVEYNSLLQGFIMQILHNPIRISANDYFTLNLKFVMAIAANIVTYIVILLQFRQNSPNLTNGNLNMTTNCTKDLLFTNSTNFNRTYVY

>MdGr20

MYRTKVNISLYKNSLWPLRVLMHICNALPWQFDELNSFDHNGLSCNIICWRLLHQLLVILLVGWLSMLRINHFEDVYYEKTDIFSIGMDAIRYGILTAIHLIVYWENTWKALTYVELFKNFETILQKFRLYLKFEVNTTYLFLYVALLYSMLTLNILITFFVIYLRYLKSLNAIRLLLEQYSETILKFKLLEYALFLVIIVTIQRHLNAFAAHYLRTTVLRLRTMPEGKEQTDILNIIGILQDIHNLLVTNVNHIENYFNWSLPMLILRMFTEIVLTSYWMYYITDYELSRLYHLYGYSSIILQLMFLFVICALCSQTEKLDTQLANILHMSRHQRHNPLLNGLLNEMSLQLYHQQIKFTAGGFVDVNYKVFGKFIFATVCYVVILIQFHMLI

>MdGr21

MYRTKVGNFLYKKTLWPLRILMYICAALPYEINEFHTPSCRVICWRFMHQVIVTVFVGWLSVLRFNRFQDVLYKKSDIFSVGMDSMGYGLLLLIHLIVYCENTWKSLYYIEIFNNFEMILQKFQLNLKFKLNISRLHLYTVVLYSMLALNITITFFVIYLRYIKSLNAIRLLLAQYSEFILKLKLTEYALFLVIIIVIQLHLNVFTKHYVRHTIPRLKCMPEGREQDEILHVIGILQDIHYLLISNVNNLEHYFAWSLPMLILKMFSEIVLTSYWIYFSVDFKINLYFQLYGYSVIVLHIIIVFVISCLCSKCEKLDAEFSNIFHMTKHERYNPFLNALLKEMSLQIFHQKIRFTAGGFIDVNYKLFGKFLFATVCYVVILIQFHMSV

>MdGr22PC

MKWQITPLLQWHIRIFQIFGFCVLSSREDDNPQFFINEHLLRLWSFLLLATSNCVAFTAIFGHDPFLHQEDLFGRFNDILKISCANLAITCSHLEDFFQRNHFRQFWMAYSKLQQFHMESNDTNNKKDKIVWSEIVKNHRFVVIFYTSTIMELFAIAMFCKFQTFNYHLILFXIPLTFTVHLRNMQFIFHIELIRQELHRLRDDLSLLVDYSRYHAYGTGFKGFENFLRSKISEKQMHFQLIYEMYANFQNSFGFSIVTVLLMVYVRVLVDSYFGYYNVYLERYVMEIIMLIPSVIQIPVFLIISKNCMDVLKFITLNLHSIISQFNGQNVSISIQ

>MdGr23CTE

MEEKISPLLKCHIHIFRLFGLCTLTFGRHPIEESFRRQRWLRLWSLFLLVTFNIVTMAVLFINDSILFSGDKFGFFNNVLIFVFSDVALTSSFLEAVFKRQSHYEFWRLYSELQDPPQENNSTQLLWLKEIRKNLRFVVMFYTFLISEIFVIGAFLMLENLLPNTIYFWLTFWPYLMVVHLRNMQFIFYIELIRQQLQRLRNDLHLMVEYSRFHAYGTGFRGFEEFLRCRIVEKQRVYQRIYEMYDHFQNSFGVSIVAVLLVIYIRIVVDCYFCYFNVYRDRLKMGVYLVLPAFFQLPMFLLTSKCCMDVVKYITLYLHSIISQYNNHDTDISKQ

>MdGr24CTE

MNEEISFLLRCHIRIFQAFGFCTQHFSDDRQKSTCIEKCLRLWAIFLLTFFNVITLVVLSCYEEFLFTTDMFGFFNDVLKIVFGNIAVTISYLETILCRNPVRKFWIVYGKLQKPQHYNPTTKHEMLNDFMKNRRFIIMFYTIVIMEIIVLGIFAANQEKQRQVVLFWSVFTPFIYVVHLRNMQFVFHIEIIRQELLRLKDDLGLMADYAIFQGTGGGLGGGFEEFLRSKMAEKQKTYELIYEMYEHFQNSFGFSMLAVPLMIYVRVLVDSYFGYYCHYREIQECETVLLTPALLQFPMFLLTSKSCMDVIKFITLNLHRIVSQFKKDNSVLSAQ

>MdGr25CTE

MKVQLSPLLRWHIRLFQIFGFYTMSFNENPQKALITEQCLRLWSLLLLVAFNSVAAIALFTNNNILYNDDKFGFFNDVLKFVFGDLAITTIYLESIFKQNDAHQFWLVYTKLQNHQFGNQCWQRSTWQKDFRKHIRFLISFYGVVFLEILFMIVFIIFQHKNRQLVLLWCTYGPFIYTVHLCNLKFIFQIELIRLELLKLQQDLQLLVDCTQQKIFEHAFWNFEEHLRSKLLEKQYMYQRIYEMYEYFQNSCSLSIVAVLLVIYFRILVDTYFAYYSHYIGWEKYATILLMPALLQIPLFLVTSKCCMEMIESITLNLHQIVSQYNSNRNVVSIQ

>MdGr26PC

MKVQISPLLKFHIRIFQIFGFCSLSFNGNYQKSLIVERWLRLWSLALVMIFNIASFLISYKNKEALYASDMFSFFNNILVIVVADMAVTVSYLETVLKQSYSQEFWKIIVKQSHSNQNYILKKELRKHHRFVAMFYTVVFSEIILLCIYILFQQRDLHLKLFWGLFLPLSYTAHMRNMHFIFHIELIRLELQKLREDLRLMVDFSHFQANGRGFMGFNEFLRSKLSEKQRIYLSIYELYDNFQNSCGLSIIAVVTMVFVRILVNTYFSYYNFYRDSMDYGTLLLLPQLLQCPMFLITSKCCMDVIKHLTLNLHCIISQYENHSTIISLQ

>MdGr27

MAGQLSLVLKFHIRIFQAFGFCTVSFGNRTIVEERLLCMWSFFLLIAFNVVTFTALINHRYFLFFEDKFGFFNDVLKIMCGNVAVTISYSETLLQRSHSYKFWGIYLRQQESSANKSHQNSWRNWFTELHTHRRFLVLFYTVVAAEIYVMYICFSITVVDFQAVLFWCIYTPFIYVIHLRNLQFIFFVELIRLKLVAVQTNLRKLMDFTNCGISKMDCEENLHSKIANTQQSYQLTFEMFLHFHNSFGFSMVAVILVIYVRIVVNTYFSYYSDNKGWEYYGFILLIPSLLQCPMFLIASKCCMNTIQDITQNLHCIDSQFGNDKNEISIQLQNFSLQILHQNISINGIGITRMDGYMLTRLIGSITTYMIFFIQFMPKFTNI

>MdGr28

MVKELSLLLRFHIKIFRAFGYCTLSFENKHKRHLDLRLWSCTLMIAFNVISYVALFGNDDFLFNGDKFGYFNDSLKIIFGDMAVTCSYLESILQRVSVHQFWVIYGELQNLHPNCSRKTTQDFWLQEIKKNRRFLVTFYTIVVIEIGVMLIFFSLQDMTRHLVLFWSVFVPFIFTVHMRNLQFIFYIELIRQELVKLQQDLSLMVDYSRFQAYGSGFRGFECFLRTKIAEKQKTYQLIYDMYEQFQNSFGFSIIAVLLMIYVRVLVDSYFGYYSVYRGWNPIELVLLIPAFMQIPMFLIFSKSCMDVVKFITLNLHSIISQFNNENTSVSLQLQNFSLQILHQHICINGVGIARMDGYMLTRAVGSITTYMIFFIQFMPKFTNN

>MdGr29a

METNKTLFQKLDLQTPLRIPLRMFYVMGLSIFDGRQHCCTTFEKVKRAKFCILNIFIIFFVIIAISIYNYDPPYGDNFGKFNDKLKLGVVIAAHLVILCESVIAGGYTNGFFQIYSKIHLKSSTDHHWKSEMKLYWKLFSYLGGSIAFILSVEITYLMQVLDKDDWLVYFTSYTPCVFICRCRLLQFILSLELIRVELEQLNRELLQSAKGTGKVQMKFYEKFICNVLPQWMKRYEDIFEMSHSLSKSMPISPLVVFIAAYIKILSDCYWAYWVNYAKFKINEIFECSLLLPSVLNILLVLVVSKNCMRTAKLIPQSLHSIRHSVGNLCLSRKIQHFSLQICHQQIIFKAFGCFTIDCYIASGILGSIATYMMFYIQFMPKFNYL

>MdGr29bPSE

MNFKNDLRINQNPLWILLRIFHYMGLSTCVSHQIPPNZQKLKLVKLYLVHLLIISVIMMITIFRYKFEPQYHNNFGKFFDILKFVVIFLVHLMTVFEAIVTGENVYNFFRLYNKLYTKWSKQSVLWKTGLRTYWKLFFYFGFSVVLTVSIEVNYIIQIRKKTEWLVFFCSYTPSIFICRCRILQLVMYMELIRVELSQLNLKILQSAKGTKKVQMVFFEEFIRSDLTQWAKQYDDIHELTHLVAKSMPLSILAIFVATYIKILSDCYWSYWMIYAKFEIHEIFECSLLLPSVLNILLVLVVSKNCMRTAKLIPQSLHSIRHSVGNLCLSRKIQHFSLQICHQQIIFKAFGCFTIDCYIASGILGSIATYMMFYIQFMPKFNYL

>MdGr29c

MKYKILCNPLRILLHVFYYMGLSTYISYKVSPKWQRLRPVKLLVIHLLIIAFLIIMILQYKYDPPYHDNFGKFYDILKFVVLFAVHLLTLLETIITGQHVCEFFHLYYKLNKSWFKSSRPLETIRRTYWKLFCYLGFSLVLTVAIEINYLVQIRKKRDWLVFFSSYTPSIFVCRCRIPQLVMYLELIRMKLLQLNQKIQQYANGTKKVQLKFFENFIYNDLTQWAKVYEDIYETSVLVSRSMPVSILAIFVATYVKILSDCYWSYWVIYAKFELHEIFECSLLLPSVLNILLVLVVSKNCMRTAKLIPQSLHSIRHSVGNLCLSRKIQHFSLQICHQQIIFKAFGCFTIDCYIASGILGSIATYMMFYIQFMPKFNYL

>MdGr30JOI

MESPWKTAAEESSQLLKALFPWQWFYGLCGMALPPCLIWDQKLSQKVWVLAWFLYLLYVGFLNFLVAELVWESNTILDMFVRDYVLDEVTKILSALQTYDIICVQLAVLWSMVGGRKTLRQIQLLVGQLERDIYAYQFSLEDKCDLFKERCSSFARRLFWQCAVFLIIHSILLGYAKFPLLWFTFSTFKKLWVLLSFHLMHAKCSEYRTILHLLDELISALQYGLRNLKYEIRRHELLGATETTLHEKLRSHQFLLSRYWYLVQLVEDYFSLPMLIFFLYNGLNIIHSINWIYVRTFLHLELDTKHPHRVTYIILLFANIMWNCWLSQICIDKYNHIASILHGIKIPAQDITLAQRLREYSLQLRHQKIIFSCWGLFDMNMKYFGLMSFAILTYVFILLQFKMQEQTDKVKRL

>MdGr31

MGFLFQFYFNSAMENSQAKPSVPENSKLVKAILPFQWFYAFCGIALPPILLRNPSNGGRFSKLLSASVWILYVLHVICLNLLVFWMVWDNNVIVELVVQRYVLDGVTKILSIAQNYDVICVQLAMALSTFIGRKTLQRIHEMVAQLEKDISCYEKSLEDKSAEFEKRCSAFGRRLLLQCGFFFVLHSVVLGYAKFPMIWDNLWYRNKLLTLFSFHLMHGKCSEYRVMMHLLDELIEALQNTLKNLKYEIARHDLLGSEGAMESRLYRKLRTHQFLVSRLWYLVQLVEQYFALPMLVLFLYNGINITHIINWIYVKSFRRNEKDTIHPYRFTYIMLLFANMMWTCWLSQICIDKYNHLCSILHAIKINAHDSALVQRLREYSLQLRHQKILFTCWGLFDMNMKYYGLLSFTILTYVFILLQFKLQVETEKAIRL

>MdGr32

MEYNFEPRIVEKSHFLRATIIYQWIYAFFGLALPPPLAQNVTSSSIGRLLLWPFFILYVAVLIVLVIWMVYVNNLVVYTYVDHYALDSITSVLSIVQNIAVAFVQITMHLVAFVGRQRSERIQKTIAQLERDIGWYSRDFSNHFGVFREEDINFRQKVMAFHRKLFLRCGLFLLVHCTLLSYVNYPLISDILSLRDRILTVLSFQLIQTKYSEYCASILIVNEFVSSLQQSLRVLRYEIIRGQRLEGNFPAYGKLMANQFLLSRVWILVQYIEDYFGLPMLILFLYNGVAITHTINWMYVRSFALDEKDSLEGFRFYFILLVFICMFWACWLTQECTDKYSQISSILSSFKIPPRDVALKNRQREYSLQLLHQKLEFSCWGFFDMNLKYFGLMALAVTTYVFILIQFKLQAETEKGNLRL

>MdGr33

MVIYTSHFSGAKLLGTMSGTNSYTNSVYIKSIKIYLWIFSLFGQTLPPVLIDKNNHKFWLYSLMFGIYLIYCILLAILALYTSHVHHQFILNNSVQYDLDVITKILSYAQNFLLVGVQIFIEIKTFFNGNTLRDLLELLADLEHELDEQCQDLFTKSSLKWKLLKISGLSFMTLVGLLLYLGQFLTQDTMDIPFRIGILFFMAAMQMKCIEYTVYLQVVYEFLEALWRNLVMIIEKIEHQPSNFEMINRLLKNQLILNRILFFVNRLGEYFAWPMLMVFFYNGEAVLNIFNSAYIKHLNQKQDEYVLFRILYMFIMLTSLFIVSALAQRCIRKYNSIGALIHNVNISSDECDLFMRLREYSMQMMHQKLVFTCNGYMDIDFKCYGKILLLISSYVIILVQFKMEESSKGSVIAPQRMFGKSI

>MdGr34

MNKFRNPEYINLLQFYQWIFCIFGSNLPPILYRQDFQGFQRQLFMAFYGFYAILLFAVAIFANCLHNTLAHTFTMMNRLDCITELLSYGHNTGLIFAYGTMEASMFWQRNRLREILRDIQEMENELMSMNEVAKTRVYLKWKLFRISGIWLIFVSGNFLYLTYFLTGGSLMPLSFKIIISLFVVAIQLKFVEYGVYVQIINDIMEHLYNSLEGIKCNVEDFPRPVHGDLPHLVSHQLLRNQQLLRQLWLLVHKINRYFALPLGLMFYQNGVAILFTVNWSYVRSLFESDDTNQIFRFVYIIMLLMNLFHICYFTEKCMDKYNHMSTLLYNFKLKFHDVEVMFRLREYSLQLMHQKLKFSCSDFLDIDLKTLGKMILAVTSFMIILIQFKMTNGTAGAIIATRKIFGISKMKL

>MdGr35

MWSMENHTRNSTTKYINLIKLYQWIFVIFGINLPPNMYYGNLSLIKRKLFHLIYGIYCGILFGLALFTVHTHNCIVEGAVERHKLDNITEIISYLHHGWIVVLMGCIEIKTLFGNRQLGEIFKLLQELENEICSRTLKTRNSLSLKWKLLWNSGMWTFFLISSITYLSHEIIASGMPTLGKIFNSFFLTALQVKSVEYMLYLQIIYDIIHEIHESLENLQSQMALVNRYMAHDLELCGIIVQNLIKSQQNLNKLWFLVEKVDGYFAATIFLLFIHNGLCIVYTVNWAYLRVIYEPKYTTQAFRYSYILILLLNIFLMCYFAEKCIGRYRSIADLLNNFKLTLHQPKQLRIRIREFSLQLLHQNLKFTCNSFLDIDFKNFGEMVLIVFAFIVILIQFKMEDVSLGALYATQKLFGKWN

>MdGr36

MSQQQTVQTILLHFSELFLLCKVMGIYPQNWKVFQRYHDLKKSNVGVLFVIFVMLAIVVLYNLLIFSFSEEDSTLKASQSTLTFVIGIFLTYIGLIMMITDQLSAIRNQKHLGEIYDRIRKVDERLYRIGCVVNNSVLELRIRIMIALTFVCEITIMIAAYIVLLDHTKWNSLLWIFSCLPTLYNSLDKIWFSTTLYALQQRFAVINRALEDMVQVHERYKAMMAHRKRSGSNNMVKNKNVINDILFDLGHEESLKLNYLQNELRGSGLAGKLGKNRVKPVITVANSMNNFNQFQSIKKQPTKSAINIHYESELSNVSRVEDKLNDFCQLHDEICEIGKKLNELWSYSILVLMAYGFLIFTAQLYFLYCATQDQPIPSLFLSAKNALITATFLLYTAGKCVYIIYLSWRTSLESKHTGICLHKCGVVADDNNLYEIINHLSLKLLNHSVDFTACGFFSLDMETLYGVAGGITSYLIILIQFNLAAQQAKDAASSHGTNDPSQISNGADNSSEDVNDYSTALTTLMTSTASTIITASSSALN

>MdGr37

MTRKPETLKPINRKQQYPPRPLLEEFSILFYIGKVMGINPQDLREFRKYRRLERSQTGDFYSIVVIVSVVMNFNLMVWVFHDPEYSVEKDNLTVAIGFVLTYFSLFIYLSDRITGLRNQDKFIELFENLQELEEELMEQGIRCNNNIIKYRVIFFIIMAAISETLVFVFTFAFLVDRDSWSAWLWTFTAVPTFCNSLDKIWFFGILLAIKKRFEALNNEFDNIAKKIENNLPLQKERKPIISAFRENKANKFNRKIHVQPVSKTDIPGIYLGEVIRNHAAFAPTTPSVRELKSSSPKPSIINEFGVLEDKFVKLCQLHNDLCMLAVDLNDLWAFPILALMGYGFFIITAQLYFFYCSNASQVIPSLFRPASNLAITIIYLFYVAVKCISILLLSWLTTVESKKTGVCIHRCALAADKNEVYELVNHLSLKLFNHVTSFNACGYFSLDMDTLFGFCGGISTYLIILIQFNIEQQQVKSASGSSKSTSPELIRNESLSNTLPHNITSLFVRLFQLDGDFTTDTLY

>MdGr38NTE

LYGIMPFDRLNARSNIFDYIQMYIIPTFYIICYGLINFGSFGIAHNPSCDSVCRLGNALIVHLGCFLYLSMHALNLWRRKKFFIVFENSLQDIDENLRRCQAVSGGDMDGKPKKKRKYLFYGTWIVVILAFTASFCYDVKELVHYYHEYFFITLMVSNFPYSAASVMLGQFIYFVSEISQRFEKLNELFEKINAESDRKHIPLMIFDIETDAKKDMPPNQQLRQRHLTATNEESDELNDDLESFYDTETIPDDGPTSESNLPELFKLHDKILSLSVITNAEYGPQSVPYMAVCFVITIFGIFLLTKVFFVVGGKSRLLDYVIILFMVWSLTTMVVAYLVLRLCCNANSFSKQSAMIVHEIMQKKPAFMLGNDLYYNKMKSFTLQFLHWEGYFQFNGIGLFTLDYTFIFSTVSAATSYLIVLLQFDMSSILKSEGLL

>MdGr39a

MSFAVPVQKTPWHKRLFRKLCTSPNYYKSMQPMFWTTFVSGVTPFRIASLPNGAKYLKTSCFGYLNLFVHFILMAYCYAYTMLHNESVVGYLLSTKVSKYGNYLHVCIGVMGATILPVAAIIRKKTLEKSFNIYLEVDRHFDQIHVGLDYSQILRYVLFVLSLVAIFDCTITVICIYCLNSISVYPSPCLIFIAVAEVLGISVTISLFCAMVRSAQRRLRRLNWVLKNLSHQWDTRNIKAITQKQRSLQCLDSFSMYTIVSKNPSEIIQESMEIHQLICEAASTANKYFTYQLLTIISIAFLIIVFDAYYVLETLLGKSKRESKFKTVEFVTFFSCQMILYLIAIISIVEGSNRAIKKSEKTGGIVHALLNKAKTPEVKEKLQQFSMQLLHLKINFTAAGLFNIDRTLYFTISGALTTYLIILLQFTSSSPPAVQAACETANSTNIANLTQH

>MdGr39b

MNSASRLERLRQCFISHQVFEALQPLFLITFLYGLTPFRVAKNNKGVTTVQMSFFGFINIALYILLYGACYIVSLLQDETVVGYFFRTKISNVGNTLQICNGLITGAVIYISAVTQRRKLLRVCEILYNLDENFANIGIKVKYSRIYRFSIVMIIFKILVIGCYFAGVLHLLKSLGITPSFSVCVTFFLQHSVLSIAICLFCFVARSFERRLVIVNKVLKNLSHQWDTRNIKAITQKQRSLQCLDSFSMYTIVSKNPSEIIQESMEIHQLICEAASTANKYFTYQLLTIISIAFLIIVFDAYYVLETLLGKSKRESKFKTVEFVTFFSCQMILYLIAIISIVEGSNRAIKKSEKTGGIVHALLNKAKTPEVKEKLQQFSMQLLHLKINFTAAGLFNIDRTLYFTISGALTTYLIILLQFTSSSPPAVQAACETANSTNIANLTQH

>MdGr39c

MDTEEVVERIPVESPLKNRLRRLFSASQMYECIQPLMFLLYWHGLSPFYIANDKNGKKELKESMWGYINVGVHILVYGACYILTLTNDHETVAGHFFQTEISFFGDFMQILSGFIGVTVIYLSAILPKQYVQHSLAIIQFMDDQLRELGVRIRYTKIIRFNYVFLASMILANLCYTIGCIFILRSGERIPSFSLHVTFVMQHTVVLYVVTVFGCFTRMLDMRFHMMQKVLKNLSHQWDTRNIKAITQKQRSLQCLDSFSMYTIVSKNPSEIIQESMEIHQLICEAASTANKYFTYQLLTIISIAFLIIVFDAYYVLETLLGKSKRESKFKTVEFVTFFSCQMILYLIAIISIVEGSNRAIKKSEKTGGIVHALLNKAKTPEVKEKLQQFSMQLLHLKINFTAAGLFNIDRTLYFTISGALTTYLIILLQFTSSSPPAVQAACETANSTNIANLTQH

>MdGr39d

MDVELQETPELEHPVIGRFRRFFTAKQFFECLQPLFFLLYWHGLVPFYIDSDANGEKRMKQSAWGYVNVALHIVVYAACYTMTLLNDFETVAGYFFSSHISHFGDFMQILSGFLGVMVIYLTAIIPKQYVQHSMAVTQEMDHLLRGMGIKIMYSKILRFSYIYILTMVTANLAYTTGSFRLLRKINERPSWSLHVTFILQHTVVLSAVAMFSCFTRMIEMRFNMMNQVLKNLSHQWDTRNIKAITQKQRSLQCLDSFSMYTIVSKNPSEIIQESMEIHQLICEAASTANKYFTYQLLTIISIAFLIIVFDAYYVLETLLGKSKRESKFKTVEFVTFFSCQMILYLIAIISIVEGSNRAIKKSEKTGGIVHALLNKAKTPEVKEKLQQFSMQLLHLKINFTAAGLFNIDRTLYFTISGALTTYLIILLQFTSSSPPAVQAACETANSTNIANLTQH

>MdGr39e

MKYVNHLNPPSTIKAKGYEMKFHSIFLNRTTMAFWLDFLNPQDTYAAEKTLLFVTFILGVTPLRIAGPFGRRRIYISRLGLAITLLQSTFFVYCFLHSFLLEESIVRFFFKTEISKVGDILQKFIGLAGMLILFGMSLRHSRDLVEMYTTVAQIDWRFRNLGVEFKYRYIMNFRHTKLVMMVVVCGSYMTSCMWILFHNQIWPSFQAVGAFFLPHVFILSVVVLNVSFAMRFGQQFDLLNRVLKNLSHQWDTRNIKAITQKQRSLQCLDSFSMYTIVSKNPSEIIQESMEIHQLICEAASTANKYFTYQLLTIISIAFLIIVFDAYYVLETLLGKSKRESKFKTVEFVTFFSCQMILYLIAIISIVEGSNRAIKKSEKTGGIVHALLNKAKTPEVKEKLQQFSMQLLHLKINFTAAGLFNIDRTLYFTISGALTTYLIILLQFTSSSPPAVQAACETANSTNIANLTQH

>MdGr39fPSE

MSSAILLPRSVQIFWRDVQKPGDIYGSLRILFLITFLGGVLPLEYRSKPKNHLKPTIPSYCYAICIFVFFVFIFLYVKTTGESVMEHFHESNVSRFTDNMRKFNGMIGLLIALGLGLZRGRVFVKLLQQLEDLEIRLSHLGLAFHQRNNALWINLVIVSLSCANLAFILYGSIVFTLSEIFVSPWAWISFYSPHLIVSCIVMLFNAIMQKVTMYFKSFNKVLKNLSHQWDTRNIKAITQKQRSLQCLDSFSMYTIVSKNPSEIIQESMEIHQLICEAASTANKYFTYQLLTIISIAFLIIVFDAYYVLETLLGKSKRESKFKTVEFVTFFSCQMILYLIAIISIVEGSNRAIKKSEKTGGIVHALLNKAKTPEVKEKLQQFSMQLLHLKINFTAAGLFNIDRTLYFTISGALTTYLIILLQFTSSSPPAVQAACETANSTNIANLTQH

>MdGr39g

MSFLPFALRVFLHDLHRPGDVYACYRLMFLLTFMVGLAPFEFHSHPRRHLSNTLFGYGNTLVRIVFYVLVFGYTMGHEQSLLSHFFETEVSRLTDNLQKFNGMSCILMILLCSWVQSKYLMRLMEQFEWIELRLSRLGVKFLQKNCSAWINLRILLTLSANVGFILYGSVGVFWRNGVAISPVTTVAFYSPHLVVSTVVVLFSSVLKKLKPYLRANNKVLKNLSHQWDTRNIKAITQKQRSLQCLDSFSMYTIVSKNPSEIIQESMEIHQLICEAASTANKYFTYQLLTIISIAFLIIVFDAYYVLETLLGKSKRESKFKTVEFVTFFSCQMILYLIAIISIVEGSNRAIKKSEKTGGIVHALLNKAKTPEVKEKLQQFSMQLLHLKINFTAAGLFNIDRTLYFTISGALTTYLIILLQFTSSSPPAVQAACETANSTNIANLTQH

>MdGr40

MGIKIWERFTKADNIFQSLRPLTYISIIGLAPFHLKSQNEVRTSALSFVAGIAHFLFFVLCFFMSRRENGSIIGYFFQTNITKLGDATLSLTGVIAMFTIFGFAIFKRDRLIGIIQNNLVVDEIFVRLGMKLNYRKIYWYSFAMSFGMLLFNFIYLCVSYMLLRSAEITPSFVVFTTFALPHINISIMVFKFMCTTHLAKSRFHMLNEILQDILDSHIEDSHAVELSPLHSVVRINRSVPRRRPTTISMASNQQQPQRYSVASIIRQNPELALRQVTNIHNLLCDICNTIEEYFTYPLLAIIAISFLFILFDDFYILEVKLNPNCVEGFEADEFFAFFITQMFWYVIIIVLIVEGSSRTIKESGKCAAIVHKILNITDDGDIRDRLLRLSLQLQHRRVRFTAAELFNLDRTLIFTMTGAATCYLIILVQFRTTHHTDPNANATNCAS

>MdGr41

MAERLLLQLHSLYFRFLGLTCYSEKYYLQIILQIFNVSIVLFEINELRKYFQNLTLDGVTSVMTITWMCIYIVYHVAHIVNCIRGMFTKSEEKAIHHLFQDIEDNFQLRLYQQTKGSPRVRKNHDFWKIFFTLFDISWFVGALILVYWRKMSTEFVSFVIFYLYIIEAVWNAFLQMAFAVMEVEEDFENLHECLQVHNRPWSGQGYRHIKGLTTENDTKSKFRPFQLKRISAMKRIYQQMHGISLKCSSLYGPKVFLANIVTGCDFTLCCYLIITNIIQIEIDWGTILIHMYSITPSIIKFLFLCRYCGRCTKKTSAILSKLTSSPMKSPLLDDFILQIRQNPIKFTAYDFYELNSETLTQVSVIVFDLMLFLLQIFSLTNIA

>MdGr42

MTTSFWEKYKDKIYIFGHIYANLYGLVVINYIPTIPTKSFRHYLALVYSHVLMFVVIVVLPLYFVYSIQDLVETKDRRWQLQLVVNFSNTLIKYCMVVVTYIANFIHYKDIRSITKHRQYLEDEFNRSSVGMDETPRKRFEFMLLFKFGLINAMMIVQISQILHAYFGDGHPVRVYFQIYTFFLWNYTENMADYFYFINCSALKFYRQLKQQLCQMVEENRLLLAYCQRRQRAGLLGHLCCVMSDRVQEFCRRYWQIYDLYRDSIRLHQFQILGLIFTTLISNLTNLFTLFNLLFKHKTFAVTGIVLNFIFAIIFYIDTYIVTMICDQIENEVKAIKKTLKEFAELPALDWRLEETLENVSLSLITFDGRFRICGLFYLDRHLTFLTAATGLSYFITLVQFDINWNNFK

>MdGr43

MNLLKYFEYWNLGFGINLNLFEISETHWLAKRRLYKIYKFLLGTLALALMPIYNVYGYSYMDAYMEKPLLPLLNRLNVQIQSLLVLMTVFAKLKTSPEQHEKLCFQFGLLNLSTKEKQKNQAMLWLKSIGFISHFLIVFMGIKFGMERKKHNFLEIFLLVYFYVVQYILQVKLFEFFYLLVKILDRMDDLLPNVEELFAKLPLNEWKLKWLLNNLSLIDEVCPGLMNFYQFFIMALLLSFFISNTIFIYMVFLEAQQLTHRNFVTMFTVFMAFLRYFDIYLSIICCEKIQGKRLECIQYLRGCEEDSQVILNYLLKLSISRFSFNIYGMFDLKKPLAFMILATVVMHGVIVIQFDYILKK

>MdGr44a

MKSRFIKMQKRFKNIRQYFLIYMGLTSYWYDQEKGVYERNSVSGTLATAVNIMGAIVLVQVLIDYLEVFENIEQRHRLMVIMSSFKYLQGLLVLNAIVHIWRTDGSYTAIKRQIEKLEEQSRSNFASSKKIDGQFKKLLYFKYSIMAYLYLSVLITSYTVLSRGMNFWTIFRIVLLANVQYLTYLILFQNFQMFWKTCRIYSYIELYISCLAEEAILELPSRNDLKERHLCYKLSWLLQLHSNLGSCLRRLQILCKSQIFQCRYNVNVNDIIAVYYAFLYPEYIKDDVAFLILVVSTNVFNNIDLYLNDNIIDMTSQHFTDLNLALKKFTGVRSYARDLERQCEEFAIYICNRKLNLKLAGALNMDRKSWFSMMSRLVMFSIILIQSHMYIDRQK

>MdGr44b

MFFNLKHSMKSFCRKFRNYFQYFVIVQGLTAYWYDESQNGFQRNALSRVVVFLAHSVGLVFLVYILIDSLELFENTGNLNPLMVIMSGYKYVQGVMIVYTIIHIWRYDMACFELKSWILLLEKEANHNMNECQGWKYKFEYLMYLKYGILFYIYWANMLLSYNSLPWQFSLWDVPLIVCFANLQMLPYLVLYQYFEVFFKICRCFCHIEMNVVSMAEKKLLGVENDTTSRLCELQRLHSKLCRVLGELKSIFQLELLVCRSNIIMCNLTAAYFTFLFILYIRETMAILGLVAVTYFFNTLDLYINDYMCDMTSSSFGDLNTGLKGFNVLQSVTGSVEKACEEFAIYICNRKLNLKLAGALNMDRKSWFSMMSRLVMFSIILIQSHMYIDRQK

>MdGr45c

MFFNVNPSLMWCFRKFQNYFQYFYIIQGLTAHWYDGRQERFKRNTLSRMAVFAAHSVGLALLTRVLYDSLALFKELEKMNPLMAIVSGYKYVQGVMIVYTIIHIWRYDAAYTKLKSLILVLEKETIHNMETLKGWKYKFEYLKFLKYVILSYIYVVNMLIGYGSLACDCFIWNPIFIVCYANLQMLPFLVLYQYFQMIWKICRCFCYIDVTIVAMAQESDNWPSGAFGYHSRLYHLLQLHSKLCRFLMQLKIIFKWQLLICRANIILFNLIAAYSIFLFLEFIKNAVELLSLIGLTYFCYILYLYITDYMSEMTSSSFGDLNMGLKEFNVLRNVSGNIEKAILNYLLKLSISRFSFNIYGMFDLKKPLAFMILATVVMHGVIVIQFDYILKK

>MdGr45b

MKSSFIKFLKKIQNIPQYLPIIMGLTSYWYMEDKGVYRRNNISGTIAVAANIMGVLCLLQDLINFLQIYENIEGQHRLVIIMSSFRYIQGLLVLCAIVHIWHKDTTYTAIKWQIEKLEEQSLNYFPKCKGIEGRFKKLSYLKYFVLTYLYLAVLIARCSQLSETMVFWTAWKIIFLTNVQFLSNLIYFQYFQMFWKTCRIYSYIEHHTAYLADEPLRDIPTNNPFVESHLCFKLSSLLELHSNLGSCLRRLQILFKSQIFRCRYTVIVYNIIAVYYIFLFQEYMKDSLLHLILVVTSYIFNNFDLYLNDNMIDMTSQYFGNLNLSLRQFNGIRNSAKSLERQCEEFAIYICNRKLNLKLAGALNMDRKSWFSMMSRLVMYSIILIQSHMYIDRQK

>MdGr45a

MNRFVKSFCGNVQNYFQYFFIAQGLTAYWYDESRGKFQRNILSRATVFLAHSVGVALLLHLLFDSFELFEGFDDLNPLLIVVSCYKYVQGVLVIFTVIHIWRYDEAYTKLKQRIFHLERDNGSKLCSSNRIESKFRCLAYLKYGIISYLYLAILLISYGSVSYDNYFLDIPLKFCYTHTQILPYMVLLQYFQMIWKLCRCFHNLDITIAFIAQEAVKSPCHMVTFDSSLYELLQLHTKLCRCLIQLQQIFKLEMFVCRSNIIISNTIAAYFIFIFTIYIPEAVIVISVASVTYFFHTLDLYINDYMGDMNSYSFEDIILKLREFNGGKNLGRKLEKVCEEFAIYICNRKLNLKLAGALNMDRKSWFSMMSRLVMYSIILIQSHMYIDRQK

>MdGr46

MNWNRLLMKFMVFFSIYLGSTLLRVDFERRQLQAANFFIKFYVTCNCLTFVLYMPYTVLYTVQQAQYYVANPVAKYANFLTLVMRLVIMYVYSLTRPHRDRELRQWFESVLDIQSSYFDRLRDLPRHTGHRKWLYVNGVLTFVHLTTLVVDIQRSTIRRQYRKTIQLYPLLGMLGVQHLFMLQHAILLCYLRECLSQINCQLLSNYQDPKLTLIYAQLRQKFLQLNKIYNPSILCILLCLVISNSMVGYAIYMIFLVPGQNLHRYDYLFGDSFYLCILVHMYLYFMICEWVMCTLKETQGILKDYINLGSQEEEEELEKVNLSCCLNSAEIKIFGMVAINVGSLFSIIAQTVLYTTILIQTEIGSYRQKGHIN

>MdGr47

MNWNRFLMQFMVFFSIYLGSTLLRVDFERRQLKTPNFIIKVYVIFECLSFVIYIPYTVLFTIQQVQVYVTNPVAKYANLLTLVMRLVIMYVYTLTLPRRDREIRQWFESILNIQSSYFDRLRDLPKNTGHRKWLYVNGCLTFVHLTTVVVDIQRSVFRRQYQKAIQLYPLLGMLGVQHLFMLQHASLLCYLRECLSQIHYQMLANYQDPKLSLIYSQLRQKIMQLNEIYSPSILCILLCLIISNSMVGYAIFMIFLVPRLNIHRYDYLFGDSFYLCVLLHMYLYFMICEWVMTSLKETQSILYEHINSGSEEVEEEIKKVSLSCCIYTAEINIFGMVPINLRALFSIIAQTVLYTTFLIQTEMENYRIKAN

>MdGr48PSE

MYRAARFATVMAYLYSILFGVIAFTYDLETGYVTKKTPLTTYCLLINFLTVSCVIYFGRNMELKMESSDKPDLHNKILVALTFIRILGVSLTLVNNWWRRDEFIHNLNTFKAFRERFLRKHSTNKRYEEYFNQQIVLKFGIGALCEVIMFYGSVRIMRQIFSVRNPMVITVXGLMSTVLNLMACHYFFIALSVRILFCIIADELRRLLTTMENLFADFHTKCIGPGLLSVKSCQLADEFDDLSGMHTELQVLSEKINSMFYVQGCCVFLILYLNNICVLYIYYMLAKQVELGPQFSHAILYFLPLALLLYYADGYMLIDLVLRYMDAIEMPAQLLKDCAAWLPILDRRLEESVKLFSLKMAAFPVSRSLLYLFDVTRPMVFATITSTITNAIVLVQYDYQYNET

>MdGr49

MNWNRFLMQFMVFFSIYAGSTVLRIDFQRRQLKTANFFIKLYVNFEGLTFLLCTPYTLVYTALQAPHYVANPVAKYANFLTLIMRLVIMYVYTLSRPRRDRELRQWFETILDIQSSYFDRLRDLPRHTGHRKWLFVNGVLMYAHFTTVIIGSYRNAIRGQLKKTLELYPLIGMLGVQHMIMLQHATLLCYLRECLCQINHQLLQGYQDPKLSLIFSQLRQKIIHLNEIYSPSILCTLLCLIISNSMGGYAIYMIFLVPGQSVHRYDYLFGDSFYLCILLHMYLYFMICEWVMATLKETQRILYKYNQSRDWDEQEELEKVTLSCCLNTAEINIFGMFPINLASIFSIIAQTVLYSTILIQTEMGSFRRKSKIN

>MdGr50

MSFTSSNRNYLGVLNSQPHSSVAMNWNRLQMKFVIFFSIYLGATLLRVDIERREVRPTNLFLKIYATLGGLIFLLWIPYTVVYTADQAQHYATNPVAKYANLLTLVTRLLLIFVFGLSRPQRDRKLGQWLESILDIQKSYFDRFRDLPKHTGHRKWLYLNSGLTYFHITSVAVDIYRCVFEGQYRQAIKLYPLFGMLGVQHLIMLQHAILLGYLRERLSQINYQLLACNQDPKLALIYFQLRQKLLQLNNIYNPSILCTLLCLIISNSMVGYAIFMLFLVPEQNSHRYDYLFGDSLYFCILLHMYLYFMICERVMNTLKETQAILYNYTRSLSQEEEAELEKVTLSCCLNNPDINMFGMVPINLGSVFSITAQTVLYITILIQTEMENYRKKSNIN

>MdGr51PSE

MRRAARFVTAMTYLYSTLLGVIAFTYDLETGHVTKKTSLTIYCLLMNMLALLCVAYFGLNMELKXESSGKPNLHIKILVALTLIRIVGVSLILVTNWWRRDEFIHNLNTFKAFRERFLRKHKNHKKYEDYFNQQIVFKFSIGIMCEVFMFFGSVRIMRNIFAVRDPVILTVFGLMSTVLNLMACHYFYIALSVRILFCIMADELTRMLKTMENLFSQCHSHCIGPGLLSIKSCHLADEFDDLTRHHSELQKLCENINSMFYVQGCCVFLILYLNNICVLYIYYMMAQHVDMGPHFNQNALYFLPVALLCYYADGYMIIDLILQYIDIIDRPAQLFKDCAAWLPILDRRLEESVELFSLKMAAFPASRSLLYLFDITRPMVFATITSTITNTIVLVQYDYKYKEVV

>MdGr52a

MRRSTRWMLAYFYYSSQIFAIFPFGYDSERREIYTSPTLTIYSTIFNICLVGFVPLLWSVEINPENMYDKDLHVVITAISSVVNILAVLITAMLVWLRRREFMKVLQEFLELRFRIFSNWPCNEHLQAKYEKAIRSKFFWCISAHICVVFGYVEFYRQQFKFDGMLLFVGIMIYNIYMEIILTNSYVFLVNVNILLEVLNGELNKILECSALLSHFEYLKEAHRSDFEDQCRKLAAELDVVAKFQYQLQQIVNRMTQLCGVQMVSDMLMIYLGNVGTIYMTYMMIQHSYMREMYQASLPPTLISLFVYYMDLRQFAFSVFDLEERFEEPGQILRLREMSEANLNDTLENSFKNFSLQLAKFPIEMKLVGLFKFNRAMVFSIFGSTISNAIVLIQYDYKNNYNE

>MdGr52b

MRRSTNFMVLVFLVAGHLMGTISFFYNHRTGEIYTSTWLTIYSAVVSLAMFGALPMLRHITINPKYFHAKIHFLIFLIRIASVLVTVVFNWTKRQEFMRALAQLIRLKKAFLNKRPLSSRLEEKYENLIRSKFCWGFASSLCLMLGSLKFFKHQFTFDNIMVILSLYVLDNVLNLVVTSYFFCILHINILLAAINEELLAILLKSEHLVHLQRLGQAPAGFFITQCCKFADEVDELARYQIDLRKIAGRINRMYEVQGACVLLTIYLNSISVIYLIYCSANIPWEDYSPWIVVWMPIALIMYYVDVGIFLYSMLSFQDLITRSGQYLKENQTCVNNLDVRLEESFKNFSLQLAKFPIEMKLVGLFKFNRAMVFSIFGSTISNAIVLIQYDYKNNYNE

>MdGr52c

MRKSTRLMVKVTLATAHVLGILSFFYNHRTGEIYTTPWLTIYTAVISVAMFGVVPMLRNINISGKRIHVKINFSIFVIRITAVLVTMIFNWTKRCAFMEYLRNLKKLRIEFEKKWPLSQNMEEKFDRTLRRKFCWGVSSSLVVFVGFMGYLKIELNINNVWMILFLALMTNILNVVLTSYFFCILRINIFLAAINEEVTRILKKSENLAYLRSRGQTHAGFFITQCCKFADDLDELARFQLEFRKLARNINGMYEVQGSCVLLSVYLNSISVIYDAYISLHILWDEYTKIRLVFTTIALFLYFMDLNVFLFTMLEYQDLLIDCGRILKEHQTCLTNLDVRLEESFKNFSLQLAKFPIEMKLVGLFKFNRAMVFSIFGSTISNAIVLIQYDYKNNYNE

>MdGr52d

MRWSTNFMILVFLAAAHLVGTISFCYNHRTGEIYTATWLTIYCALVSLAMFAALPMMRHFAVNPKYFHAKINFLIFVIRTAAILVTVIFNWTKRQEFMGILGALISLKKEFTSKWPLSRKLEDKYEQLIRSKFSWGCASSLGLLFGSFEFFKHQFNLDNIPALLGLAIMSNVINLVITSYFFCFVHINIILAAINEELSTILQKSEHLSHLQHLGQVHAGFFITQCCKFADEVDELARYQIDLRKLARRVNDMYEVQGVCVLLTTYLNSISVIYMMYCTANIPWEAYSPWVKVWMPIALTMYFVDLAILLYAMLGFEDLITQNGQLLRENQTCFTNLDVRLEESFKNFSLQLAKFPIEMKLVGLFKFNRAMVFSIFGSTISNAIVLIQYDYKNNYNE

>MdGr52ePSE

MKRSTLWMLGVYYYASQLMGVLSFHYDTNSGEIYTSPSLTIYCAVVSILTFTALPLVLRVDLNLQTMNAPDLHIRIVGAICSIRIVVILLTMTMNWTKRHTFMTTLRRFVKLRQKFLRKWQLSSGVENKFETAVRLKFLWGSLSDIGLILGSLEYFRHQFRLENPILSLALGVYCSILNIAIFHYYFLILNINILLRTINEELQRIMEQALKENPTKLCIQLSKDLDELAYFHFQLHTLVIRINDMYGLQGISATLCVYLNNVAMIYMNYMAWQYTYMREFYSLWTEVTVFAMICYYVELTICFGCMMDLLVLYDHPGZMIKEWENIGRPLDARLVETVFKNFSLQLAKFPIEMKLVGLFKFNRAMVFSIFGSTISNAIVLIQYDYKNNYNE

>MdGr52fPSE

MSRYKIIYDKLSVFLHAAHMKRSTIWMLWFCYFASQLMGLLTFHYDYRSGEVYTSKLLTMYSAVLGIVMLAVLPLTLQLDFDFKNVRAPDLHLRISAIFFVFNVGVILTLILLNWTRRQCFMQTLRDFEGMRRSFLLKWPLSPAVAEKWESEFRTKFLWGCLSGVLIVMGANGYFTVLFRRQNIWVYLPLNLFLQIFSVSMFHYYILLLNINTIQLAINEELENILKISRNHSLSWGXTGKWVRDLDLLAVTQYSVQGIVKRINRMYDLQMICVIFTVSLDFLTLIYMSYMSWYHPKVRDYFSAWTKIALVLGMLFYHVDVKNCTICMFRVRDYGEHAGFLLKQRDEFEAPLDQGLEEGFKNFSLQLAKFPIEMKLVGLFKFNRAMVFSIFGSTISNAIVLIQYDYKNNYNE

>MdGr52g

MRHSTILFLRFSYFASQLLGALSFNYDYRTGEVYTSPLLTTYCVVINLSTLAVIPLLFRLDFGPETLNAPELHIQITSITFLLRPLTIFVTLIFNWTKRQGFLQTLRDLERLRRNFHTKWPLRPRVEEKFEQDLRAKCLWGILTSLFMIMGSREYFQKIYKVENIWLYLTYALFCQIFNILLFHYYFLLWNINAMQASIKEELMEILQDSRKATSNLTGKVSDKRPSSKIVDNLAEAHYALEQLVKRINCMYDMPVLCLFLTVYLNNVALMYMAYMHWYHTYMQEVYSLGTMTLMNFGVLSYHVDLKLFLKCMFGVHDNWENIRMVLRQWQDISPQLDSILEESFKNFSLQLAKFPIEMKLVGLFKFNRAMVFSIFGSTISNAIVLIQYDYKNNYNE

>MdGr52h

MSRSIKWVIAVSYYFSILFGVLSFCYDQKTGEFYTSTWLTVYSAILSVGMFYVLRALMRMDFNPTISNGHDLHIRITGVIYIIRIAVILLTVVINWIQRHRHVAILREFQSVYRSYCQKWQCHEKLQEKLESKIKWKFCLSLISNLGLFVVSWEFLQVHFKLESLFEIYVVDTLCIILNLIIFHYYCCMVNIAFLLGSIYEELKRILELTKTLVRLHMMGHLGSGPYGRHCWRLSSDLDDLMAVQLQVQLLATRINRIYSIQGACCLTNMYMNNVTTFYMFYMLTEHEYIIRSYSCWTVIVLWITLVSYNLDLKMFLYSMFDYVDFYKDIRELLRERQPCQSLQNKRLEESFKNFSLQLAKFPIEMKLVGLFKFNRAMVFSIFGSTISNAIVLIQYDYKNNYNE

>MdGr52i

MRRSTSWLVALTYFTSLVLGLVGFCVNRKTGEFYTSPLMTVYSGLMGASMFSVLPILLRMDFNPTTSKGHDLHIRISGVIYLTRIAVILISVVINWSKRHQYVAILGEFQEFHRAFCKRWSCNEKLEEKMENDIKWKFYLGFVTNLGLFVVSWNFLDIYFKLRNSFEICLVDVLCVILNLIMFHYYCCMVNLNYLLGSIYEEVKRILELTHNLWSIEMRGHLVAGACERLARDLDELMRAQFQVQSLGNRINNMYQLQGGCCLANTYMHTVTVIYMAYMVLQHEYILQIYSRWAVIIIWCTLIFFHMDLKIFFQSMFDFVDFQQKFQELLRDRQTHLPLKSEPLEESFKNFSLQLAKFPIEMKLVGLFKFNRAMVFSIFGSTISNAIVLIQYDYKNNYNE

>MdGr52j

MANITHLLISLCYYSSKMLGLLAFSYDTKSRRFSTNPLSTWYCAFIRLVVVAIIPRLVVDDLYQRNVSISELHQQVWLAIYVIRIASVLISVVFNWSQREKFMQTFNDLEAIREYFHKKWPKWNEGLESEYNRSIQTKFLWSFLANMGYALEHLAIWRTQHHMVALFVMTFLNGVISVIMTHYFIALANVSTLLIAINKELQGILDDCDHLVRLRSFHKIGCGFLMTRSCQFSDEIDELARIQYQMQLLFERITNLFDIQVVMVLLTVYMNNIAVYYILYVWANDEHLWRVYSHWSLYLVPLVIFCYYMDIQMSRKNMLQIEEQFVETARLLKERALWWPMLDSRLEESFKNFSLQLAKFPIEMKLVGLFKFNRAMVFSIFGSTISNAIVLIQYDYKNNYNE

>MdGr52k

MKTFHLLVSSILQVSMKRPSRWILAICYYFSLLLGILSFGYDLKTGKVYTSRILSIYCGIINVAMCGILPLIFTQLHLSPGNFLKLHFPLKVRIIVCCIRMVAILLTIFLNWTKRQEFMRTLNYLQDMRSEFRKMWPLSDRVEHYFDRAIVLKFVVGLIANFCISMESSAVGHPNIQWSQFWIGVIDSLAIILSVIMTHYYNTISNVSVMQMVIREELREILLKSQMLSHCRNRNLIKHGIFIRQSEKLAKLLNELASSQYRLEQLVHRINAMYDIQGVCLLITIYLNNMVFVFIWYLLLGKMYVMIQWNQWAALFVPFTFGVIYADLLIFRFGLLRPVDLARETGQLLRDGKLMCLRLDKSLEESFKNFSLQLAKFPIEMKLVGLFKFNRAMVFSIFGSTISNAIVLIQYDYKNNYNE

>MdGr53

MLGRLPLWWFHFINCVCIFTSTAIYSIDFEKRQLRKPGLCLKIFVYLPCFLCTCLLPLAIIDSLSQDRLFFQNIVAIYVNWMTIFVRFLLYGVFVLGLCHRNGRIGHWLEKVLELQASYFDGHAEVPKDMQHRKWLYFNSILACTHYGLESYLNGDSDRDDEILTDWAQVSLFVMVNVQHFYMLQHATLLCYLRECFSQLRHQLATKEITTRLNLIYNQLRNHYEELNDIFGPLISIILLCSFLTNSMVGYVMLMYLKLPNFQIDLYLYLFGNGLYFWLLLHWYIYVMLCDRVESAIKDIDWVINEYTTEKESQREIELIVFSRCLRWPGTNICQLIDINRGYLFCCLAQTLSYIITLIQCDYVNLI

>MdGr54

MLRTLKHLAFYAMMWTSYINFINGWWLNLDKRQVKRLKLSIRILMWLPTVFMLLALPYGTLLAMSRDRLYATNPVAIYANYTVIMARTLLYYIYAWTWNRRDQQVLQWLEKMLRLQREYFDWHQAIKRSLRPWLYVNCLLTMVHMWGISFGIFSDDIEEVGRDQWVSYPLYVMIVVQHFHMLYHGGFLCWLQEYFSIINQQISEQKLNPQLNLIYWQLHGMKEELTDIYCPVMLFIIFSLLISNSMVGYITLMKLMLPELHTQSYAYHFGNLFYILLIIHWYSYFTIGQRMEETIRDTELLLYDYVTEPWLCADTYERELEMLIMSRSLNTAEVQIVGINLNWSSLFAILAQTVSYIITLIQLDYVNLI

>MdGr55

MLQRWWFQDIVIFCIVVSSLTLYLDLAKRQVKHLRTWLKTLVYMPIIVIAILMPFSLEETFKMSHQYLSNPVIIYANNTTAMAKMVLFLVFALTMQGRDRNLEKWLETMIEIQTSYFDRYPSRGVAKDMSHRKWLYLSSGIAVLHYVLESIRSSIKNFATEDANIACFTFFLLLTLQHTLMLVHGTLLCHLRECFSVLNIQMAGKSHDPQLPFIYNRLRCQYRELNRLYGPSMLGVIVCLLLYNSMVGYVALVILLIPDVDGDSFRYLFGSLFYGFLLLHWYIYFMLCQKVETTIRDIDVILCEYAIDEGQESGKQLELLVFCRSLHQASVNFCGIIDINWSSLFCILAQTIAYIITLIQLDYVNLI

>MdGr56

MLQHLWFKAIVICCCIAGTLDLRLDLKQRMVKPLRLWLKVIVYGPAYFGVCIVPWSLWNSLEISDSHLTNPVVRSANIVTILVRIALFFTFVRGIYKRNRKLEKWLRRALAMQKAYFDGLPERETTGRSISHRKWLYLTSLITCLHYAMETCSELDSNEARSLLYFMVTIQHFFMLTHGALVCFLRECFSVVYHELRMEICRFPASRVYSLLHGLHRDLNEMHGPIMLCVLLSLLLSNSMVGYIGLLQLLMPNFNGAHFDYLFGNALYGLLLIHWYIYFMLCQRMETTIKQIDMTLYEYEDHEDTKKEIELLVFNRSLNESSVCFCQLIRVNWNSLFCILAQTVSYIITLIQLDYVNLI

>MdGr57PSE

MLQRFWLQTVVAFCIFSSGINFGLDLKKRRLRNPCLYIKIYVYIGVLYAIVVVPWIIPETVGKSHLYLRNSVAIAANNTNAVLRLGLLLSVGLTMHRRNSNLKEWLEKILKIQIDYFDCLPQDGRXRDVPRIFPHRKWLYLSSLITFLHYGIESVKMYMNSSDMPEFAVYPFFLLLSTQHTFMLIHSGLLGYLRECFSILNFRLAEQQLDPQMCRVYSQLRSMHEELNRIHGFSMLWLILCLLLSNSMVGYIGLVMLLIPDMDGDSYRYLFGSVFYCFLLVHWYIYFMLCQEVETTIKEIDLILYGCISASEDNTNEKEFELLIFSRCLHQPAVNFCGIIDINWSSLFCISAQTLSYIITLIQLDFVNLI

>MdGr58

MHEIRRVYLVLMQPPKMLQHLWFNIIVICCIITSTLDFRLDLKRRLVKPLRPWLRAIIYIPIYCNLLFAPLSLWEGLRMSLSHLENPVAKSANIATILVRIVMFLLFGLSIYIRNRKLEKWLERAAEMQTNYFDKQSGDEAREKSISHRKWLYFNSAVACLHYITDSCGVLNANFSRLGLFYMVTIQHFYMLVHGALVCYLRECFALLYQELRKKNTVFPLSSIYNQLHCLHRDLNAMHGPTMLCVLLSILLSNSVVGYIGLVKLMLPDFSGDRYEYLFGNIFYGLLLIHWYIYFMLCQHMETTIKDIDIILYEYVVYGYAGNSHIEIELLIYSRSLHESSVSFCHLIKVNWSSLFCILAQTVSYIITLIQLDYVNLI

>MdGr59PSE

MLQHLWFNIIVICCIISCTLDIRVDLKRRIVKPLGRRLRVIFYAPVYACLFLVPFSLWNGLEVSHTHLTNPVAQSANIVTILIRVFMYAIFAASFYGRNRKLEKWLRRALEMQTNYFDKLPENEVSGRCISHRKWLYLSSVIACLHYATETYFEVISNDARTTFYFMIIIQHFYMLEHGGLVCYLRECFSILHLEMRQKSARFPVGHIYNQLHCLHSDLNAMHGPTMLCVLLSLLLSNSMVSYIGLLHLLLPNFNGARFDYLFGMGSMVSCWSIGIFTLCLVKIWRQQSKKLIXILYEYVVHGDCRYSQNEIELLVYSRSLHDGTVDFCQLIQVNWSSLFCILAQTVSYIITLIQLDYVNLI

>MdGr60

MLPRWNHLFFYLLLLISIGNCVTMLWINLEKRRIRKIPYVLRLLVWFTLALLLFLLTIGCGLTLTRDKLYETNPVALYANYAVMLTRTLLYHVYVWTMRGRDRNLQEWLEAMFRLQGDYFDNFENYLSPNRSSQRRWLYFNSCLVVVHAVEAYKNMYNNSYSQGGYQKIIVYPLYGMIVIQHFYMLHHGGLLTWLAESFALINQQLRQKSFNPQMFGVYRELLVLKDELNAIYGTILLWVLLCLLLSNSMVGYIALMQLMLPQLHSPSYAYLFGSKFYILLLVHLYSYYTICHRVERTIGEIHFILYEYTTETWSTTNGNYERDIEMLVWSQRLHGSTIQIAGIAINWSSLFCILAQTVSYIITLIQLDYVNLI

>MdGr61PSE

MLQRWWFQAMGIFCIITSSLTLRLDLRQRQVRHLRPWLKILVYVPIISAIVLIPFTLVETFENAHQYLSNPVIIYANNITALARIVLFLIVVLTMHRRDENLTKWLEEMFEIQTNYFDRLSTAPKDISHRKWLYLGSVIAVVHYTNESTNSGTNNTKAGKVDFKWHSFFFLLSTQHTLMLVQSVTWHVSQVESNVWPQYAGCNCLPAPLQFHGGLCWPCYVADAQCGRWQFSLPIWQHFLLFANAALVHLFYALPKGGDHHXDIDMILCEYVTAEDKGNEKEFERLVFCRCLNPASVNFCGIIDINWSSLFCILAQTISYIITTIQLDYVKLNLNEMY

>MdGr62PSE

MWIFYGWNHLFCYLLLLLRIGNCVSMLWINLEKHRIMKIPYVLRLWVZFNLVLLLLLLTIGCGLTLTRYKLYETNPVALCANYAVMLTSTLLFHVYVWMMRGRDRNLQQWLEAMFPLZGDYFDNFKNNLPHKRSSQRRWLYFNSCLVVVHAVEAYKNMCNNSYSQDGYKKMIVYLLYGMIVIQHFYMLTTGCWHGYRNPLPSLINNSGRNPSILKCLGSTVSYWFLKDQLNGIYAPILLWVLLCLLLSISVXIAINWSSLFCILAQTVSYITTLIQLDYVNLI

>MdGr63PSE

MWQHLWFNIVLIFCIASSTLSVRVDLRQRIVKHIWLLIRIILYIPVYGSFITSPLALWNGLEVSHSYLSNPVAQSANILAILVRILLFTLFASTLHIRNRKLENWLRQCXTYFDKLPGDVISGRATSHRKWLYLNSALACLHYVAETYSELNSDDVRNNFYYTIVIQHFFMLIHGALVCYLRECFSILHRALRTKPTGFPVNRIYNQLHCLHSDLNALHGPTMLCVLLSILLSNSMVGYIALLRLMMPNFDGARFDFVFGNVFYGLLLVHWYIYFKLGQDMEATIKKTDLILYEFVNPEEGGDSQKEIELLVYSRSLHEPTVDFCQLIQVNWSSLFCILAQTVSYIITLIQLDYVNLI

>MdGr64

MLQRLWFKTVVVFCILVSGINFGLDLNRRQLRRPCLCIKIYVYVPLIIILTLVPRIIRETVGKSHSYLTNPVAIAANNTSEVLRLGLLLMVVLTMHRRNRNLAKWLEKIFEIQINYFDCLSEGVTRGGGGGAGYPKDISHRKWLYLSSDLTIIYYCIETVKLNLNSSERPELAVYPLFILLSTQHTYMLIHSSLLGYLRECFSILNFRLAEKRIDPQMTRVYNQLRGLFEELNGIHGLSMLWVILCLLLSNSMVGYIVFVMLLIPDMDTDGYRFLFGSIFYGFLLVHWYIYFMLCQEVQTTIQDIDVILSGCTTTEDNANDRELELLVFSHCLHQPTVNFCGILDINWSSLFCILAQTLTYIITLIQLDYVNLI

>MdGr65

MQKTNKMRSFFQSKSVIQCFQLMFFFLFHTGCLCFRLKNGVRLYYTKLSLIYTYSVRLILLACFMGGVVVKLTTEEYYSAMIGRLSPIITFVMCFESIVSVFTYLAVTFGLDRTRKEHLKAWNRLQSIDDEVVKSFPNVNWNYQKNCRKYTRLTAFIYSYFSIIAFGFVFNLANCSCGYFSSFLISFAYACITASSGLASFLFAVQMDMLRLRFRLLHKLVNLNFVSCSNGQRNDTRLLRKFKILEYFFKEYNALIHRLNRVFNVVSSASMFYDFAILTNMGFLVCSKAIESNTHWKEYVFIAFFTLPRIYKVIICSVYGHMRKNCWQEFVRIENYFNKSFVIRDDVECFFHWRMHNNYNFTVGKTIRFNLGLLFMIFNSIANYIIVLIQLQFQQNMIRRTLYGAPSGDIEMIEM

>MdGr66

MFRLHRFWKKTQSIYDCCRLLCQIQFVLGCSGIRSRSDKYVCDWISLSYTALAMGCVLSTLGLAAFVKFQDPYLVEMDSLIKSIIYLELGMSLFMYVTTATTMVAEAKTHLKLYKQINDLDLVLIREFGCKMNYKALVKKNLQLLGFTASIYIIIIALGISRAKDLRNIVLNLLSALAYICITGGPNLNFYIQMNFAEILAIRFRLLQKLLQAKPPRLEEAKLVERFQKLIDLVEQYHDCIRLTNQIFAKSLIIIMLHDFTLTTSELYLIFGGLTSSGSSALIYFVLLGLVLPIYKMTVGPVYSENAIKEEAKCFKIIQDLDFQYNGSRKIRDMVAICLTWRWDNIVEFKSGSMPLNMETIAGVYVEIFNYILILIQFRMTQEMGDQIEKQKNTIQDWIGVDYV

>MdGr67

MSQPTSLTLNPILKFCFYVVVFITQLFGLLNLPFNFKTKRFSQKGIYNRIYCGILHLLYCGFLPFAATSPVTDNAAYKKASFYVILNYAITILRLPALFFTLWGVWWHSKNLYCVIQDFEKLRLENFHNLKESKRYQILKKNDRLVWSKILTTMSVMIMFYFRIFMFAKEPSLSFILLSIYFGCLECLTIYTINFFFCGICYANCALHYVKEILDELEGDSISFRIHRLSQVFGDICKTTKNLFIIFQWQILSIMLAAMIALIALFFNLIILWFTSPRIFQIPVIILTLQAAFINMGEIFVTAYVLNDMKECLKDIQRVLMELTWKCDFFNNKELDNVMDMFSLHLCVRAPQANLCGFFDFDMRIAIKFLQAMLIHLILLVQFHFRHMV

>MdGr68

MSLRINERLEKMVWWINYYHALVLGLMPGLYNKDTRNLKSPKIYIAYSVIIQCVFMLLTPMATPFMASREEQEDYYMNRKLILRWTYHIGKTARILVNIVMSLEIWFKRGRMIRLYEDYWKFVRKYQQFCAYHDMEPYMEQELATVRTNTIYKFGVCHANAIIMFILFIRMQKERSWTYMLMILVNLLQSQFLLQVNVTFDLILFRMHLHFVFINKVLQHTSQRSTRGELFWSYWTLYNMHYECYHLSQRFLRIWQDITFFWMIKIFTTNIALLYHAVQFTNGSIESDNTQDLIGTMTIVLFYWDTTLTMKAIDGILSSCNQTNEVLRIYANEKGERGHLHQQSQFLKMITQFHQYLACHKLQFNIYGLFPLNKATCFRYFFFALVHLIVLLQFDLKSKM

>MdGr69

MVEFLTIYYYTSLVVGLTNLRYDGGTQIVELYHWPTIVYSAVLNLVFIMLQPISMLHSSRVSLNCDEFGALVVIKLLSGIAYFLAYFSIMCMSWLKRKKIHQLYYKYLALTRRYFTETMLLDNYEAVQRAQRIFLKKFCSSVCKAIVVYVNIYHYYTESPCEYVKSLPYVAISLYYGLLNVQQLIVDVNVILGLLLIDLCLSMLSHTLEEIERDIWLMAKARKVQENIFLKNQQMLHRKWRGNLNRTVEAIAAEVMHLQSLTHEHLDIYEIPVLFLLLAVFISLITMMFNIMAYVADFGNVQPLKLSFYVFILLANISNVMIFYNICESLQRTYARMVNQVYRIGIYASLGSGGYVERDTLVLSGRIKKVALCKHIFKIFIPSTIGWRVGFIQFY

>MdGr70

MFSSRYRIKSVATPPGQVQQMTNKDFNKIKFMEFLKVYINVFEVFGILPYAGTDCCLRYGQRCWCVVLLLGIWIMCMAEVCAIDAKLTSMEKFLFFCELFLYAILCCVIYFNTFFNNNALKDVGLRIAKNSERLKACYKMLGDGIVMENMYLRIKREVQVLAVCLSLFQILCITINMLYRPTFKWGLIRPLLAYNIPNILINFNLCLYWLLLRFIAHQLQCINGILKYLPQVRSGVEEESSSILYPTSLWFQKEFYGSKSYRKTMPHNVHGIFLKLQKINADLYGVLTAIVEIFRIVLVLNFLTSFVVLTIEFFSLYKYFDNPSLNELILVIFKFVWLFLHTSRIFFVLLTNYAITKKKCQTLYILNGTPLEIFESENDISKFLLQIMVRNHTETACGIVDLDLMFLLGIINALAMYIIFLIQSDLGNASLNETLFNTTTT

>MdGr71

MSIKMKKDFKLYSNGTTLKFQNKNKVHDDGDDVRKHQYLKKQLYGTTKMLLRISQIFLCAPMGVQKPKSQETTKERLIYYIHFLWCTGLYLGLVVCVYDEYTSSNIELPTVQKPLYFSEYLVYLMHLFVILLSIFGGRETFWKFYEFILDLDRLLWQRGIPVNYKGLQWFIRQHFLLITAHLVATVIVGYFYSFGVWLNFVRTSTVYVIPNIIIHISLVQYYTLLYLTAERSDWSYDLLQQLLGNPSSTKSFQELRLELHFIRSLYAKLEQFTRDVNDAFSYSIILVYVGSFINISINIFLLFKYLGNWETSNLAWTAYSVVWTCMHIGKMSLILYYNENIQSKKTRATHLLSTYRYENMALEPAFRHFILQLMSDTRSNVICGLAALNLNFVTSLLVAISTLFIFLVQYDITYEALTKTFNSARPTIA

>MdGr72

MKSHSFGGNFRTQHQSTASTWLSTINGFFLCTLSIASYGLCRVLGILCLRYNFRETRVENTALTFAYSVVMLVIAVFYTPIALQILYSDMVFLRQNDLLTYVGYIRYGVMLTCALATLFMQVIFRSAIISSVNQMLHLSGLLLDRPSFVNGYVTWKVVSKCLTVVLQALWTVFLIENDNAVSNMWYLATLVFVHYCLMVLQMTLNMLYFGVLLITLLIKQVNANLVGLLLNLRTLPHHRGGVNAQSRDKLCKDVGQLMHFHYMLVKLSTTYVNLYGWQLLSFLMSVIMECVTQIFIMYFVPAEMARRERKSNDAEARPPPIPINPFALMYVIGLLWDMFLIVVMLDDMRLQFFHTRHLYTSSIWLRALASPANVRLEGCLSHFNLYLLHAQPRISYSACGLFTFDKTLILVTLERIFLYLVLLIQFDLITN

>MdGr73

MDDNKMSPRQLRYCAESPSDDPPPTLMAKLRHLWNKIFFAVIRVMIFCDQLTLLGPFVVERKKSSSGSSRLHFRTHRVFTGVAVSFCVGLIVVTPFLAKIIPDLYDTSRKDQDTLFKRIAQFTMLTDVIGTLLIMSAQIWHRNKLVEILNSFVDITEKMRFYEHDFINFKTFLALMVKVGLTCYDLLMCLPFLFTGASRLSGTDICAFVALVAMQHLTSIFGLAIFTAILGLLTMSLQLERQLTHFENIASNLKMLRLITLQNALQRLISLFVNTLQFGIFIMMLIKFITILCNIYAFLDYYVTTDRVYTTFIMYLVSVSLELYSIILMAYLCDRSQRKMPQIFIMVESSVLWPQIEKFSILNLFILHNEFALFLLAYSINFLVIILEFEITKAGKRL

>MdGr74

MNDFESIAKAKPQEPERPQRYTKWILLGLFNYGRFLDIINCQWDAKQLQMRPVNKVYKTVTSILRVFIVIVYWDVVPDVLKSFLNERRGFVNLFSMFQVTSVVAFSVGLFLMKVRDSFKIIQLINRFVRLNIKVAQLSQNSFSLCKKSISLFFLKSIITLLGYINEMPHMLEVQGLNINSSVNIVIGVYLWLGSMYVLDACYLGFLMLTLMYGNLGSHLQKMLNNMKHVEGGSLVGSSLTTYNRMKLLCDYSEKLDELSAVYTNLYNITKDFVHIFQWNILYYIYYNFMVIFLLLNHCIWQYIRSNFIDFTEIMFVFVKIANLVLMIMCANDTVEKSEMVNQLNLDIVCSDIDARWDTSVETFLSQRKVENLEIKVFGFFTLNNEFILMMLSAIITYLFFIIQFGMSGGFGTSSMGGES

>MdGr75a

MNGAKVAKFFITLFTAFLIGVGLLDLWYSFRRKRFAISPFLIIWSFAIIAVFVFVYGRRLYEEFKTDQIDMKNAVSIYYYLNIVCAMVNYFSQLIQVRKLLQFYNAIPLFKCLNYFNINHCSVKSSAMLIVIKNILFPIIVEVNLILRELRKGEDANLLATLYNLYPMVIANFLPNCLFGGFVVCRECIKALNVRLKLIEKEANFYQNTKQMMLHTIFHRMQIYCELSDKLDELTEKYTQICYYTLAYMDLNSLPLLCSLLSNLFGITAGCFQQYYAIADTMINEETYDVFDAMTNGVFLAVSFSEIALLNMVVNDCIGKVHETSIILKRIQINNCDIRFRQSVEKFSLQIFVENFKIQPLGMLEINVGLLHDVLSAVTSFLLILIQSDLTLRFSLK

>MdGr75b

MAVDKSIFKMVLALLFGIAYSFGLLSCAYSRRERRFYINNLLMIWCIGMTITVTIGSAKQLYAAYNDDKINLANAETLYYYISIVGVVLSYICQLVQTTELREFLSNVPLFEILDYFELKRSVVKSSIQIILVKTVVFPIILEINLLIRQSRNEPEESLLKTFYTLFPTVVSNFLPNCAFSSIVVCYHTMRALNLRLEKIEKEANFYQDVKQIILHKRFYRMQKFCNLADTLDELSQKYTLICGYTLRYVDINSVAIMATLLCNLFAITGGLFQEYNALADTFINKENYDVFDALTNGVFLSIAVADIALYGSMANDCLEAVHETSIILKRIQINNCDIRFRQSVEKFSLQIFVENFKIQPLGMLEINVGLLHDVLSAVTSFLLILIQSDLTLRFSLK

>MdGr76

MTTSAEMASRKFYEYLLKVRAYFLGNFSSSELGYVVFPFLKIFKLFGFMPIRLDQSYLFNERSKMVWDLWAILWSFLGSVIYVGGFVMGVCHIASSKGIERLHEYIVIAYFTTWGQLLSLFILGGFGVLHNWLNMQQLQLLLSRIARIDEQLDRATGRAVNYACMRKKLLMQFVVVFVLTASMSMINCIIIYSDSDNLIFSSSCFWFVCFFPILLLTFKEFQFYNMIFLVKSKFEIINEELTRYGSNSQSQRDRMPNDLLEIFPKSKCSEDDLKQLLHIYVNLSDCVDLLLRIFAWHLVSLTSVSFGVITIQGYNLFAALIVRVLHMSSYHLTVTIGWIFLQIGVICINVSVCSATDRAIFSMEVVQRRNSFTAAGFFNMDYKLITSIIAAVTTYLLIIIQFHTSMGNPIVPSV

**The Ionotropic Receptor (IR) family**

In addition to the OR and GR families in the insect chemoreceptor superfamily or seven transmembrane proteins [24], there is a second completely different family of olfactory and gustatory receptors in insects, the ionotropic receptors [21], which clearly evolved from the ionotropic glutamate receptors involved in synaptic transmission [22]. These proteins are somewhat larger than the ORs and GRs, and have a single transmembrane domain at their C-terminus. They function as obligate heterodimers, usually two and sometimes three different proteins. While some of these IRs are highly conserved, and have been implicated in olfaction, others are highly divergent and most of these are implicated in gustation. Like the ORs, and probably many GRs, the divergent IRs function in complexes with some of the conserved proteins, specifically IR8a and/or IR25a [23, 41, 42].

Naming and numbering of the *M. domestica* IRs is complicated. Following the example of the Benton group (Croset et al. 2010), the conserved orthologs of most IRs in *Drosophila* are given those names, even though they have no cytological meaning in *M. domestica* (like the OBPs, ORs, and GRs, they were named in *Drosophila* for their cytological location). When *M. domestica* has multiple paralogs related to a single or multiple *Drosophila* proteins, these are indicated with a numeral, e.g. MdIR76a1. There are some *M. domestica* IRs with no clear simple orthologous relationship with *Drosophila* IRs, either because the latter was lost, or they are simply too divergent, and these were numbered from MdIR101, which avoids confusion with any of the DmIRs, because the latter only go up to DmIR100a.

The MdIR gene set consists of 110 models, which is a considerable expansion from the 65 in *Drosophila*. The automated gene modeling for the OGS as REFSEQ had access to all available insect IRs in GenBank for comparative information. It succeeded in building at least partial gene models for 100 of these 110 genes, and 5 of the missing ones are pseudogenes. Some of these are large gene models that concatenate two genes. 39 models were precisely correct. The others required at least one change, and 5 new gene models were generated (Supplementary Table 8).

The IR family contains several conserved orthologous genes shared across insects. The co-receptor IR8a and 25a genes are unusually highly conserved and because in larger trees they cluster confidently with the ionotropic glutamate receptors from which they clearly evolved [22], they were declared as the out-group to root the tree (bottom of Supplementary Figure 7). Many of the other *Drosophila* IRs have simple single orthologs in *M. domestica*, presumably serving similar roles in chemoreception, e.g. 10a, 21a, 31a, 40a, 41a, 60a, 64a, 68a, 68b, 75d, 76b, 85a, 87a, 92a, 93a, 94e, and 100a. There are several simple instances of recent duplication of genes in the *M. domestica* lineage, for example, IR84a has two paralogs in *M. domestica* while IR76a has three (Supplementary Figure 7). Most of these genes are also those that show the highest levels of conservation and one-one orthologs across the *Drosophila* species, and are implicated in olfaction [22].

Many other relationships are rather more complicated, and hence simple orthologous naming was not employed even though some orthology is implied. For example, the set of DmIR7a-g (and IR11a which is a *Drosophila*-specific duplicate of IR7a) are vastly expanded to 26 genes in *M. domestica* (MdIR101-126), including 3 or 4 lineages that were lost from *Drosophila* (top of Supplementary Figure 7). All but IR101-103 appear to be in a large array, albeit not all in tandem, in scaffolds 18656, 7398, 19274, which are inferred to be adjacent in the genome (Supplementary Table 8). Similarly, DmIR56a-d and the related IR62a have multiple relatives in *M. domestica* (middle of Supplementary Figure 7) and are in two arrays in the genome (Supplementary Table 8). The relationships of the remaining genes at the top and bottom of Supplementary Figure 7 are less clear, with evidence of duplications in either species, although the largest of these is clearly in *M. domestica*, the set of MdIR163-178, which might be related to the DmIR52a-d genes, although this is not revealed in the tree. These genes are mostly in small scaffolds (Supplementary Table 8), so might in fact mostly be in a large array in the genome. These genes, and those below, are similarly divergent across *Drosophila* species, and are mostly implicated in gustation [22].

Finally, there are multiple implied losses of IR gene lineages in each species, approximately 8 in *M. domestica* and 11 in *Drosophila*. The combination of more gene losses in *Drosophila*, and far greater gene expansion and retention in *M. domestica*, leads to the considerably larger gene repertoire in *M. domestica*. Only four of the *Drosophila* genes are pseudogenes, however, while at least nine of the *M. domestica* genes are, and some of the incomplete gene models might in fact be pseudogenes. Thus the intact set of *M. domestica* proteins is probably ~100, compared with 60 in *Drosophila*. Interestingly, like the ORs and GRs, the *M. domestica* IR pseudogenes are all relatively young, with only one or two obvious pseudogenizing mutations each, which is in contrast with most other insects examined to date, for example, the ant *Pogonomyrmex barbatus*, and even with *Drosophila* which usually lose pseudogenes rapidly (e.g. [24]).

In conclusion, the IR family has undergone considerable expansion in the *M. domestica* lineage. Most of the expanded lineages are highly divergent lineages, and most are implicated in gustation versus olfaction.

**110 MdIRs in FASTA format**

>MdIR8a

MDFIQITVIVWFIVPAIFANDLNIAFWIDPVQKDIYGDIAATLKEIEGLHLETKIVDTVMVIEPGDDDDDEVDSMEISERNMRTFCDILSVSGISIILDFTYLPWHQGLDYVQAHGIPYMKVDRILRPFMQMFSAFLQQKDATEVVMLLQNERDKREAIEEMIRGLPFRTLILNAGDSNRTDFVKILRDLRPSPGYYGIFAKGSNMNSIFDKILKGNVFARPAEWHFIFLDTRDRVFKYKKQAENGNKFAVNPKAVCKSLQMKDVYCQSGFTFQRALLLEIFRALIDIRQSRWLEPILMDCNVTTSETMEYLKDFDILDHFKLNDFMTFSPVNPENTFDDERPEMIPPLSYSVNVSINFYSSEHEAVTDLAVWQNGEMKKINHTISPAKRFFRIGTTEAIPWSYYRKNPNTGELLLDANGQPMWEGFCIDMIESLAEKMNFDYEIVTPKKGKFGRRDPVTHEWDGLVGDLVSGETDFVVAALKMYSEREEYIDFLAPYFEQTGITIVMRKPVKQTSLFKFMTVLRLEVWMSIVGALVSTAVAIWLLDTYSPYSAKNNKKAYPYPCRDFTLRESFWFALTSFTPQGGGEAPKAISARILVAAYWLFVVLMLATFTANLAAFLTVERMQTPVQSLEQLARQSRINYTVVEGSDTHHYFINMDFAEKTLYRMWKELALNASRDFHKFRVWDYPIKEQYGRILLAINSSMPVADAEEGFRKVNEREGADYAFIHDSSEIKYEITMNCNLTEVGEVFAEQPYAVAVQQGSHLADPISFAILELQKDRYFEELKAKYWNRSRSNCPLSEEEQGITLESLGGVFIATLCGLGLAMISLVFEVLWNKRKQKKIAGDIVQVKPVDVKDPPVEVWHSEAKLTPPPSFETATFRGRKIPSGITLGSEFKPGRVGLNRRLLSRRPDEDTPPKDELPAYME

>MdIR10a1

MLFIKAHVFAFLYFCKSIATHHPNPQRVRGKKSNFHINFVIFVLFFKTFRQKRRRFFTLIFSSIAMPKQVNRSIKIFQMALIIALILNIFILTKSHRITPDFIGERLKEPLKYMHSLQMKIRLQHAGEDLENPYIKWFLRYGDITKSLNTYNIEDNNLKPLIHRDNYVICTDMRRLQLTIDLFGRAVGSFFFIMDGGDVNVEALLPYFRSTFYEHLIFPIYLLIREDILIYDPFALDASGRHGQIMPYNGESDPQHRLFRDMRGYPLKVLLFKSVFVRPIYDAATKKVKDYSGVDARVAYLLQEHLNFTLELQEPVGDPYGGRLPNGSFSGALGMILDKKTDICFTGFFVKDYHTSDIAFSAAMYDDRLCIYSRKAKRVPYYLLPIWAVNHNAWIGFIGLAFFSAFMWMVFRTLTWKMEIYSHDENKSLKWQYLIILKDTWVLWVRVNVNHLPVMSTEKVFVGVLCFVSVIFGAIFECSLASVNIKPLYFKDMKTLQEFDDSGMHIVIRYISMADDLFAPDTSALFDRLRNKTTFNADVKHNLMQDILQNGNVAGVKRWRSLTLDNLELAFTKQIWMIPDCPKVYHISYVWLRYAPWEEPINYYLLQYLQFGLIQGFEQAMRHEAYVQIIKKGLNVSREAFKKLRIEDFQLAFYVVLAGNVVGSIVFLLEKIWALRNSRNCQ

>MdIR10a2

MLLRFIIALIIFLFQISKIATKNKSENENQDIKGDLIQTWLNIPLKNVVSLNLLLRESNFEEDMENPFIQWFMKYSQLPYILTSYGKETENGIKIGRSSSYVIVCNMEHLKSNVKHHAQRGATNFIVINDMKLDLKAIQEAASFLWNQFRILNVFYLTLYGVYIYAAFSLDDNGNYGSMTAYKGENTLNKILFHNMNHYPLRIQIFQSVHSRPILNRMTKKVDHVHGLDGRVAQNLQIRMNFTMDLLDPDPNYFGERLPNGTYTGAIGSILDHSVDICFTGFFIKDYLTRDIEFSVAMYDDQLCIYTRKAERVPDYLIPIFAIKLSVWISFIGIGFLASVVWICLRIVLISLKIHRRKFRNKDLQRPLKWQYLLILKDSWVIWVRQSVNYYPAFEAEKVWLISLCLVSMVFGAIIESSLASSHIEPLYFQDIRSLADLDKSGLPIVYRHASMKDDLFVGNQTSELYNRLDNKTRYMPNRNVSILDEIAKYGKATVVNRYNSLMLESLDVLVKKQIWIIPEFPKHYSIAYVWLRDAPWKDAINMWLLKFQQAGIISKFQRDMKIEAKLDVMKKHLYENAVGLRILTIRDLQLAFYVVIYGNILALLLCLLECCIFKSK

>MdIR10a3IP

MIFPFFIFIFGFLIQTSQSFQGILRIMELQQNEEFTIEQINKWLEKPLNDIPYLDVMLRENNYTKDMDNGYIEWFLKQTRISFTLNTYSIGDKRKFAGLKMGEASENAIKHYVIVTSFKEFQQTSLYFAQHSGIYFFVILDEFRLRELREICQMLWTKHQIFKSFLLTNRGVLVFDPFAWNNRTGKYGKIIQYTGEKSLERTIFYNMRGYPLRVQQFSSVYSKPMLNPITKKLHVHGVDGRVSDVLQESLNFTRVLLDPDPHYFGQRLPNGTYNGAIGSILDHSVDICLTGFFIKDYLARDIEFSGAMCDDQLCIYTRKAEFGAIIESSLASSHIEPLYFQDIRSLADLDKSDLPIVYRHASMKDDLFVGDQTSELYNSLDNKTRYMPNRNISILGEIVKHGKAAGVNRYNSLMLESLDVLVKKQIWIIPEFPKHYSIAYVWLRDAPKDAINMWLLKFQQVGITSKFQHDMKIEAERNVMKKHLYETAVGLRILTIRDLQLAFYVVIYGNILALLLCLVECCIFKCK

>MdIR10a4IP

MILPLFILFFGFGIKTGKSLQGIMELQQKEEFTMEQINKWLEKPINDIPYLDVMLRENNYTKDMDNSYIEWFLKQTRISLTLNTYNIGDKRKFPGLKMGEASENAIKHYVIVTSFKDFQQTSLYFAQHAGIYFFVILDEFRLRELREICQMLWTKHQIFKSFLLTNRGVLIFDPFAWNKRTGKYGKIIQYTGEKSLESTIFYNMRGYPLRMQQFSSVYSKPMLNPITKKLQHVHGVDGRVSDVLQESLNFTRVLLDPDPHYFGQRLPNGTYNGAIGSILDHSVDICLTGFFIKDYLTRDIEFISLCLVSMVFGAIIESSLASSHIEPLYFQDIRSLADLDKSDLPIVYRHASMKDDLFVGDQTSELYNSLDNKTRYMPNRNISILGEIVKHGKAAGVNRYNSLMLESLDVLVKKQIWIIPEFPKHYSIAYVWLRDAPKDAINMWLLKFQQVGITSKFQHDMKIEAERNVMKKHLYETAVGLRILTIRDLQLAFYVVIYGNILALLLCLVECCIFKCK

>MdIR10a5

MILPLFIFFFGFGLKTSKSLQGIMDLQQKEEFTMEQINKWLEKPINDIPYLDVMLREKNYTKDVDNSYIEWFLKQTRISFTLNIYSIGDKRKFPGLKMGEASENAIKHYVIVTSFKDFEQTSLYFAKHAGIYFFVILDEFRLRELREICHMLWTKHQIFKSFLLTNRGVLIFDPFVWNNRTGKYGKIIQYTGEKSLERTIFYNMRGYPLRVQQFRSVYSKPMLNPITKKLQHVYGVDGRVSDVLQESLNFTRVLLDPDPHYFGERSPNGTYNGAIGSIIDNKLDLCLTGFFVKDYMVPEMEFSVAVYDDKLCIYTPKAKQIPESILPILSVGYDLWLVFIFSAFVCGFIWVLLRYLNLRLKLWSRLQTEPTINGKLDKPYKWQVVRIFIDTWVVWVRVNINHYPPFNSEKIFIASLCLVSVIFGAIFESSLATVYIHPLYYKDVQTMEDLDKTGLFVIYKYTSMGDDLFFSETSPLFASLNKKLKHVKDLNADILKDVVEIGGMAGVTRLTTLLLEYLSYIRAKRVWIVPECPKYYTISYVWHKNAPWEETVNQLLLRMQSAGLFDKFIDDMQTDVDIKLSTDQTLAQQKEEFKVLTVEDLQLSFYVILLGSLMAFVSLLFERRKKRKLTGVEQTLSG

>MdIR21a

MSEKIIKRYQFNTDIYQSCESREAQALHNRKPRRVEPIFRGKPKPRRDVLATKFHLNLDNRQTASLVSLVNKIATEYLSKCPPIIYYDSFVEKSESLLLELLFKTFPFTYYHGEINSRYVAHNRRLKNSIDSNCQSYILFLSDPLMTRSIIGPQTENRVLVISRSTQWKLKDFLSSEKSSNIVNLLVVGESLTADPNKERPYVLYTHKLYADGLGSNKPVVLTSWLRGGLTRPHINLYPKKFQNGFAGHRFQVMAVNQPPYIYRIKTLDFTGVTQVHWDGIEYRLLQMMGQKLNFSIDILDNPNTGRNERPWELLEYNVAQRLVDVGMGGMYVSNDKLESVDFSVGHSKDCAAFITLASKALPKYRAIMGPFQWPVWVALICIYLGAIFPIVFTDRLTLSHLLGNWGEIENMFWYVFGMFTNSLTFSGKYSWANTQKVSTRILIGSYWIFTIIITACYTGSIIAFVTLPAFPDTVDSVMDLLGLFFRVGTIDNGGWEYWFQNSSHEPTFRLFQKMEYVSSVEEGIGNVTQSFFWNYAFLGSRAQLEYLVQANFSNENMSRRSALHLSEECFALFYIGYMFPKNSVYKQKLNSLILLAQQAGLINKIESEVKWAMQRSSAGKLLQASSSSPLRETIQEERQLTTADTEGMFLLMGIGYAIGAIALVSEIVGGITNKCRQIIKRSRQSISSGWSSRRESVVVLPGNEAKKKMHHKTREKKGFGWRQLNLTRTTLKELYGDNHGEVQQEHKIKSSHKSQWGGYHNMDTENNSDDAASLKSTVNEFILNERPSKHNKHGDIIEQVVDRFLKEELENTLKTFDQTLATYQEDEEENEERLSAVTHPEDAEEIFGSFVSSFLDENAKVLDNLQLFKDPNSGSEHEAPQEQEREENTQK

>MdIR25a

MILPRLKFIHIVLLFLKILSRRYLLVSSQTSQNINVLFINELDNDPASKAIDIVQTYLKKNSNYGLSVQIDKIEANKTDAKALLESICIKYAESIENKQPPHVVFDTTKSGIASETVKSFTQALGLPTVSASYGQEGDLRQWRDMEESKQKYLLQVMPPADIIPEVVRSIVRKMNITNAAILYDNTFVMDHKYKSLLQNIQTRHVITAVAEGDSARADQIERLRNLDINNFFILGSLKTIGQVLESVKPAFFERNFAWHAITQNEGEVSSKRDNATIMFLKPIVYTQNRERLGQLRTTYNLNEEPQIMSVFYFDLALRTFLAVKDMLQSGAWPANMEYLGCDDFQGGNTPERNIDLRQAFVQVTEPASYGDFDLVTQPGKPFNGYSFFKFDMDVNVVQIRGGNSVNSKSIGRWTAGLDSPLVVNDEEAMKNLTADTVYRIFTVVQAPFIMRDETAPKGYKGYCIDLINEIAEIVHFDYTIEEVEDGKFGNMDEKGEWNGIVKKLIDKKADIGLGSMSVMAEREIVIDFTVPYYDLVGITIMMQRPSTPSSLFKFLTVLETNVWLCILAAYFFTSFLMWVFDRWSPYSYQNNREKYKDDDEKREFNLKECLWFCMTSLTPQGGGEAPKNLSGRLVAATWWLFGFIIIASYTANLAAFLTVSRLDTPVESLDDLAKQYKILYAPLNGSSAMVYFERMANIEQMFYEIWKDLSLNDSLSPLERSRLAVWDYPVSDKYTKMWQAMQEAQLPATLDEAVARVRNSTTATGFAFLGDATDIRYLVMTNCDLQVVGEEFSRKPYAIAVQQGSHLKDQFNNAILTLLNKRQLEKLKEKWWKNDEAQAKCDKPEDQSDGISIENIGGVFIVIFVGIGMACITLVFEYWWYKYRKNPRIIDVAEAASTPPGKDVKLAEGIILGQTGKEYEKANAALRPRFNQYPHNFKPRF

>MdIR31aNJ

AAKGIKQLSSFNTFMKIVNLKSSKCMEALFTPKVHAKTSIFIDCRCIEAGDVLHKGSNGMFFNKTYQWMLWDEANKCLPLLYKLKNIGPNAQLIKVHRQNSTFVVSDCHSKGRHLNAALEFIQLANFFSNGSSTILDYIDRTQNIYCRDNFNGLLLKAATVIDQDNITSNIEIEDILSRSHKESGVAAFAKYHYALFCILRERFNFTVKFRNARGWAGKLGNSSLRLGYIGIMQRNEADVGASASYNRINRFDFFDILHQGWKLETAFIYRLTPNIGYKNLKGDFFAPFHIYVWFIMGGICLLLTVVWMCIEYMVSKKTDQFTAVNVIPVNVVGAICQQGMDPSPMGISSRIISLTTFVFSLIFYNYYTSSVVGGLLGNTVEGPSTIDAIISSELKVSFEDIGSYKILFQYNKTPRIRKLLEKKVLPHRGPKDLPVYTHLEDALPYVKKGGHAFHCEVVDAYPEIAKQFDVSEICDLRVVFGLLESELLNFVIHKNSPFTEIFRIVMRRAVETGLDKRILKQRQPEKPPCSNLYTVYPVDLTGTFSAFIFLAGVKYSQETPCVEFQNIGGIVMWGYTISWTNVAHLRT

>MdIR40aNJ

CPDENLEIDPDLRVHVDEFILRLHQLYFKSVIFYDTELFFRFVEASLAGSIESVNLIFRHPDELTSMILDRKLAHRLGLFIFYWGAKHPPKRSEINFREPMRAVVITRPRKKAFRIYYNQAHPDGNGHLSLVSWYDGDNLGLSKEPLLPPASQVYSNFHGRIFRVPVFHSPPWFWVNYENDTAANSTMDSLNSDESYANGEDEGEGDMELSEVNVTGGRDHRLLQLLAKHMNFEFVYIDTPGRTQGSLVNETFTGGIGLLRNGLGDFFLGDVSLSWERRKAIEFSFFTLADSGAFATHAPRRLNEALAILRPFKADVWPYLILTVIVSGPVFYFIIYIPFRWQADFRERQMKKKIKRTAFHMVYIQEITRMDNRVARRFAKAEGLSRRSQKAEDELPDNLFNKCIWFTVQLFLKQSCQELYHGYRAKFLMIVYWIAATYVLADVYSAQLTSQFARPAREPPINTLHRLQKAMIQDGYLLFVERESSSLEMLENGTEIFRQLYALMKLQSPDEEGYLIDSVEAGMHLIADGLENKAVLGGRETLYFNIQQYGSKTFQLSQKLYTRYSAVAVQIGCPFLDSLNNVLIHLFEGGILDKMTTAEYETQSRMISKDMKNKNRNNNNNKNEQNKNLKSHQGDPAEMSPLGDETNNPNESQGKSADNAEMKKPQAATTIIQPLNLRMLQGAFIVLVVGYTLAGGKRE

>MdIR41a

MGGKTVVEMLSAPAMVINWSPIINVIMQIYLQNSTICVLWPQDGELQLDTKFEKFPYSIINIDATNSDEKLMENEVQNIKEKFMEDNPLTLMLTLAIEKSHCESFVAFENDILKFIESFANASRYSVWRSKRNYFVFGSSDRSLEYSLERQRFFEDQPNILMVSGDKATPGIFELKTNKFVGRRADGPGNLCLLDRFYVNTMNFEKGANLFPYKLGNLQGREIIVPGMDYRPYLVINYVQDKNNSYDLAFDGSAEGNVQIDGTEARVILTFCEIFNCTVLIDSTEADDWGEVYSNLSGIGSIGMVAKGMAEITIGAMYSWDTDYIYLDMSMYLVRSGITCLVPAPRRLASWILPLEPFQFTLWLAVVVYLFVEVASLALAYRFESHFISMMADSWPESLKFGVVTTLKLFVSQSGSKKVISQTVRVLLFTCFLNDLIITSIYGGGLASILTVPSYDEAADTLDRLWSQKLQWAANSEAWVSAIRNAEDDRINGILENFFIYPDEKLEQLASSRSGFGFTVERLPFGHFAIGDYLTTESINHLKIMQEDLYFQYTVAFTSRCWPMLSAFDNLLYWWHSAGLDSYWEWRAVADNMNVQKQKQVEATVYSNIEDMGPVKLGMANFVGILLLWMLGVTISFLVFLYEVLRDYVERKNKE

>MdIR60a

MCLLHWTCSIVNPDKESASMVIYLQKPSSLGPRTWLAGVNCLDQITRLFFRKQESLTRSPNMVMTVAKNMSTPAAQIQEGFLKIMMEAVSELDPVHKRYQMRIVSDAQPYLWYKMNQPELVLADYYVIVVDSLMRLANLLQNYVSHMLSWNPGAHFLILYNNAKNRNNADTTAETVFQVMLDQFYIHRVGLLYATTDTRYVFKVLDNFNSSSCRKLKVKHFAECQEGSVVTKNFGALQRSLDRFLSSLTLTNCTFYMCASISAPFVEADCVFGLEMRIIGFIKRRLNFNIIQQCEHESRGVQEEAGNWTGLLGRLNEKSCDFIMGGFYPDNEIISNFWVSDTYLEDSYTWYVKLADPRPAWMALYSIFEDLTWLAFIVMLLITWLTWFVLVYFLPEPPETREWSLTGINSMAVSICVSVNERPLCMASRFFFISLALYGLNVTSTYTSKLISVFSNPGYLHQIDTLPEVVEAGIPFGGYEESRDWFDNDEDYWVFDKYNDSSDFEPHTRNLVWVERGKRVILSRRMYIMQSALADNIYAFPVNVFSSPMQMIMKPGFPFLYDFNLMIRYMRDFGFLNKIHRDFVYNNTYLNRIAKMRPDFKEKVIVLRMDHLQGAFSILSVGVCVSVGLFLAELLVFHVGGRCSSKSGHKKRQRKRDKTRKRKRNKSSEEIVIYWNEIHVQKDMGSTPLKRRIVHKSADE

>MdIR64a

MVKDQLKENNNNVHGEPKITVDCQDNINDMECYSNVENVMNENENKNGINQNTKANESKTQFQAKLIRQFALTHKKMSRINLFTCQVTGNHNRDGSPKESYRNLMERKKETAQLLDQLFTGGKSLDKLESDNRGLILKIIQIDHLIPKKRTDSGPANQRGRFERTNTRNVGGPNSRNSLSNTNWLDQILRPEYYSQLVVVDLACGEASRKLLEMASNKALFNSLYHWLLMEDYTFNGQTGINDADDDMKNKKNSDSKTETGTATGTRARNTNDDDDVAAAAGDMENIENFLEKLNININTELILAKRRMDYYYLLYDVWSPGRQYGGKLNTSEIGEFSASQGLELVDWYKGSSFIMRRLNMHLARIRCLVVVTHKNGSNSLHEYLISHIDTHLDSMNRFNFALLSHVRDLFNFSFVLSKTATWGYLKNGKFDGMIGALVRKQADIGGSPIFFRIERAKVIDYTTRTWVARPCFIFRHPRSTKKDRIVFLQPFSNDVWILLAGCGVATILLLWLLTTLETDGRPVSAVIPTKSFPHGSFKKRLVRWGGLLCGYDIRDDNSATQRVGMFLESILFYVGSICQQGLTFSTRSFSGRCIVTTSLLFSFAIYQFYSASIVGTLLMEKPKTIRTLRDLIHSSLEIGIEDIVYNRDYFLRTKDPDAQELYAKKVTSMPTADGTGFVDAPPDNVVLPTSIIPMTEAQKAKAYRDILHSHETGAHAKTNEASNWYEPEYGVAKIKKGHFAFHVDVATAYKIMADTFTEKEICDLTEIQLFPPQKMVSIVQKGSPLRKPITYGLRRVTEVGLMDYEHKIWHSPRPRCVKQLHTDDLRVDMQTFTSALLVLMFGILVSGLILSLEIMHHRMWQQYTTTTTTLTMPITTTLTRTTTE

>MdIR68a

MESLRLLLAQILVVSRIERCFVVIADDWYDPVYNKAFFQYFHEPLTHFYIKIKDSEDLKAPNYQTVRVLKQIKVFNCDIHFITLLNGGQVKRLLMFLEKYRVLNTKRKFVFIYDERFITEDMLHVWSNMISSIFVKPLEEDGSFVISTIAYPNILNGIVVTKRLIEWPKGGHIRKIQLFPNTSTDLKGYQLPIAVYQHIPMVVASEGESGKSFNGLEVEIIKSLAKVMNFQPDFYESRDTETERWGTKLPNGSYSGLIGQISSYSAVMVIGDLHMFTAYSAVLDFSRPHSYECLTFLTPESSQDNSWKTFIQPFSSSMWTGVMLSLFLVGTVFYFLSFLHALLMRKKSSKLNAKSFFAPFRKRHSISHMNIQRFRDVKFRRYLNQMTVAQRQEDLFDNFSNCILLTYSMLMYVSMPRVPRNWPLRVLTGWYWLYCILITVSYRASFTAILANPAPRITIDTLDELLQSHLTLSVGSIENKKLFDNAFDQVLKELGTSTDVLTDITGVTEKIAKGGYAYYDNQYFLQHLRLMSTESSDDDAVLHIMKDCVVKMPVALGLTRNSPLKPHIDKYLERLMEAGLINKWLQDTVKHFPNDELAPAEAIIDLRKFWSSFVPLVFGYFCGFVALLLEHVHFRKVVMPHPLYDKANTRLYYNFKRKFPNN

>MdIR68b

MKNHQIGLIIGLFSTWLRIGATSLWKIENNDNFDSGLQEEEKLKFALKICDVVQQRDAKINILYRNPTREHMETIYRRINVDALHQCMTEFPLTIRNLHSYAMEPERLMGSLNIYFIATRTIARQVANFLNAHQRWKPGHRYLFVWLLEEDKSDEVLHEFFQQIWQKNILHAVAILDSQRVYTFEPFSPEGFRIKLLDENQNYFYDKLKNFHHFEIRITMFIDPVRAIPLPNYATEGYKRIDGRVANAMVKYLNATARYITPADNETYGSLINGTFTGALKDVHSGLTHIGFNLRYTLDHVKQHIEELYPYQRRFLYLVVPAAQMRPEYLIFVKAFSYSLWRLLLLHFALVLLLFKLLQHLVGRLPAQHIGSCVTQKWHWYELLEMFWKTQLGEPVEGFSRISSLRQFLIAWILFSYVLTSMYFAKVESNFVQPAYEPEIDSLEQLPQLNLPIYAFDIVFEAVKVSLNPKYYEWINAHGVRVPPNIRVEQFAFAVTQKNAEVALMLHDEMAKELLAHSYNDVTKRPSYHIVKEYLRSLTSSYILTKGSPFIHKFQSVISAFHEFGLMRHWLQLESQPNTYTHNSEEFFEDLDDDFDLYYDEDGAGNVGGGGGGGTTTTSASLQSHKKVVLNLDILQGAFYLWLVGIFISCMGFAAEWLTYWWSLRREENKYQVDFYENQ

>MdIR75a

MLHIHLINMILYNFVDLKLSCVVVFQCWPQEFLSQFSMAASQQHLYGQYVSLDDPTALVDMAYAYLRYRRPKIGVFLDMNCNQTERAIEKTSQIRFFNQHHYWLIYDERSDMSRFYKLFQDANLSVDTHLTFMVPREDQMGMRNLSRSFYMAFDVYNNGWLIGGKLNISSNFELSCGKEGCYKSKFLTDLHKRSLTGNREALRDVVMRVAVVVTRYDLDAPPEEINNFLLTQEDFHIDPLARLGFQVLRLLQESLYTNVSYTYYDRWTDVEYTGGIVGSLVNETADLTSAPFFMSANRFRFLSSLAATGDFRSVCMFRTPRNSGMHGGVFLEPFSTKVWILFGCILILAGVLLWLAFFMEYHEMERYIRTYIPSLLTTCLISFGSACAQSSFLIPHSWGGRMAFISLSIITFIMYNYYTSVVVSSLLGSPVKSKIRSLRELADSDLEVGLEPLPYTYTYLNFSSLPDVQYFVRTKITSKKNSESLWYSASDGIIKMRLKPGFVFVFETSTGYNLIERMYDAHEICDLNEILFRSDTLLATHLHRNSSYKEIVRMKIIRILETGVHSKHRRQWVRTHLNCFSNNFVINVGLEYTAPLFLMLLCGYGLVLILLLFEIVWNRWEMKAERLSSYEPVS

>MdIR75b

MVNLSLLNFVLYNFLANRLKWVLIFNCWNGNAQVKLSDLLLKENIYAQFWKINDVQESEVMAESYFKHLSPLVGVYFDFNCLKSEEFLRKVSEQKLFRQHFHWLIYDEKSDFAKFHSLFENFNMAVDADVTYAFPNPAIVNGPQNMSYLTYDVYNNGLYLGGKLNMTGDEEVNCSPKGCERKRYLSTLHEKTRNENRWLLGDITMRVATVTTYLPLTTPPGKILDFLASDDNKNRDAIARFGYAYIMILKDNMGCQYTHNYTNTWSVTEATGGVVGQLGMDQSADISSSPFLISKLRLHYVKPTMPLGNFRQVCIFRTPRNAGIRGEVYLEPFSGRVWLIFSGIILLIGFVLWITFVVEYHQLRLYLNFLPSLLSSCLLALGSACCQGSFLVPKSTGGRMTFFSLSLLTFIIYNYYTSIVVAILLGSPVKSNIKSLAQLAESNLDMALEPIPYTKAYLNFSKLPEIRSLVRNKIQTKKDPKSIWLPITDGVRRVRDEPGFVYVTESYSSYSLIENTYTAKEICDLNEILFRPQEILHEHVNRNSSYIEFIRYKQVRIFESGVHRRLQGIWVRTRLPCYLSSGALVQVGLEYTAPLFIMLACVYGLVFMLLVLEVLWHKYLDNMGLMARIRGAAMGNE

>MdIR75d

MKFAILFVGIIFCRFIAPLGGKKATSDHHQINKIILEYFKFHGVRTMNFIKCPRNESGPHHLEPKKLLPYLIRENMPVRVWSGMDYLKKDPIAPLYGPPITFQRNGSIGRIPLNIKMETIAHKTGIIVDNFNTPCALNVLSWCGASEQNYFTTNRFWLLLGTNEGDLELLEDPGIFLPPDSEVKVLLRNENKTYALLDVYKVAADKELKVREVLGNFTEVKEMLKGLQKYGSPISYRENLEGITFKTGLVIAFPDMFTDINDISLRHIDTISKVNNRLTLELANKLNMKYNTHQMDNYGWHQPNGSFDGFMGRMQRYELDFGQMAIFMRLDRIALCDFVAETFRIRAGVMFRQPPLSAVANIFAMPFENDVWISILILIFFTIFVFTLELVFSPHAHEMDFWDGVVFVWGAMCQQGFYFSFGNRSGRMIIFTTFVATLFLFTSFSANIVALLQSPSEAIHNLKDLSQSPLEIGVQDTVYNKIYFNESTDPVTNLLYHKKIAPKGDSIYMRPMIGMEKMRTGLFAYQVELQAGYQIISNTFSEPEKCGLKELEPFQLPMLAVPTRKNFPYKELFRRQLRWQREVGLMNREELKWFPQKPKCEGGVGGFVSIGLTECRYALAMFGYGVLLAIIIFCCEIILKIMHRMGKRMNAYYKGDRFPPGVNAE

>MdIR76a1

MLAAEASHWSTVINIILQLYFSDLTTTCVLWNKDFDLHGTSFVNFNVVLIINPWNLNDTFSKDIYNFEKQDNQLSNDGIDFDDWIKKFVAAISHTHCEGFVVFQDDIPRFAHTYRKASVYSLWRSFEPKFLFAYTKEKLTEDYFQDLLFKIIETDFKNATQFYIKTNKFVGSLFENPNELIDVSVFNAIEGTFEPTVDLYPRNKLQNLQGREIIVGAFDYRPFVVVDFNRLPLYYDHAEDNPRHLVHIDGTEMRIVHTFCELYNCSVQVDTTEKEEWGTPYPNYTSDGMIGTIIDGKTHMGMGAMYAWYMAYKSIDQTTFLGRSGVTCLVPAPSRKTRWTLPIRPFPYSLWLAVIFCLCWETVALCLTRFFEDRVVVRQNNASIWSSIQFAYVTTLKLFISQSSRYVVRSHTVRTILFACYMIDIIVSSIYAGGLSAILTIPDLTEAPDSVARLYSHNLTWTSTSYAWITSIVDEGDGPKDPIFHRILANYRINSMDEMRSKAKTENMGFALERMAFGHFGNGDFFTPEALQHLKLMVEDIYYSFTVAMVPRMWPHLPKYNDLILAWHSSGLSKYWEWKIVAEYMNANEQNRVQASMYNHIDVGPVQLDVDNFAGFIGLWIAGIFMSILVFIGEWICYWWNRNQI

>MdIR76a2

MIVTETSYWSSVINVILHSYFSNLTTTCVLRHKEYDLLWTSAAINSNVYLQINPWTLNESFSKDIHNFDEQDKQYTDDGIYYDDWVKKYVAAIAQANCEGFLVFQDDIPRFAQTYRMASVYSIWRSYEAKFLFVYTNETQCEDFFQDLFFKNNANILIIEAEYKNSTKFNIKTNKFVGSFFENPHELLQISQYDALNETFEPNVDLFSRHKLQNLQGREIIVGAFDYRPFVVVDFQRLPQYHDYAEDNPRHLVHIDGTEMRVVHTFCEIYNCSVQADTSEKSEWGMLYPNYTADGLIGMIVEGKTHMGLGAFFVWYIAYRSIDQTSFLGRSGVTCLVPAPTRMSTWALPITPFKYTLWLAVILCLFAEALALFLARLFEEHLIEEQENLDIMSSVEFAYSTTLKLFISQGSDYVVNSHTVRTVLFACYVIDIIVTSVYGGGLSSILTLPDLSEAADSVERLYSHNLTWTATSYDWVALLEDEIEPLYQRLVTNYRISSREEMRSRAKTENMGFALERMAYGHFGNGHFITSEALDRLKLMVDDIYFVFTVAMVPRMWAHLTKYNDLILAWHSSGLSKYWEWKIVADYMNANEQNQVQASIYTQIDTGPVKLDMSNFVGLIAPWIVGIILSIVVFIGELIYYRWRQGKENRQIIPDE

>MdIR76a3

MISTENNHWATVINIILQSYFTDLTTTCVLRHKDYDNAWLPTEDSNVYLLINPWNLNDSFSDDIYNFTHQDLTFNRNGIYYDNWTRKYVAAIKQTHCEGFVAFQDDIPKFAETYRKASVYSIWRSIKAKFLFAYTKEGQRKNYFQDLLFKKLQQITTFDALTKRFEPNVDLFSKNKLQNLHGREIIVGAFDYRPFMVVDFQRSPEYYDHAADNPKHRAHVDGTEMHIVHTFCEIYNCSVHVDTSEKEEWGMVFPNYTANGLMGMIIDGKTHMGMGAMYLWDLAYKSIDQTIFLGRSGVTCLVPAPTRITSWSLPISPFQLTLWLGVFLCLFWETVALFLTRYFENQVVEQRENSTIWSSLQFGYVTTLKLFVSQGSDYVVTSHTVRTILFACYMIDIIVTSIYAGGLSAILTLPALEEVADSVERLYRHNLTWTATSYDWIVSITDKEQEETDPIYRRLLDNYRVNSMDDMRRKAKTENMGFVLERMAFGHFGNGDFITPEALARLKLMVDDIYYQFTVAMVPRMWAHLPKFNNLILAWHSSGLSQYWEWKISADYMNVNEQNQVQASMYTQQDTGPVKLDMKNFAGLILPWFIGIILSILAFIGEWIYYWWDKKMGTKVIKLRD

>MdIR76b

MATGIELILASALCLSCANETITYPQGLLMVDQNYEVVSEAPIGDVLDTSLDDAPAETLNTFLEKAEKLTKLKSWLNGRHLKIATLEDYPLSYTETQPDGSKKGMGVSFILLDFLKEKFNFTYEVLVPKGNIIGSKSDFDGSLIQMLNTSVTDMAAAFLPLLSEQRSFLFYSTTTLDEGEWIMVMQRPRESASGSGLLAPFEFWVWILILVSLLAVGPIIYFLIILRNKLTGDNSQKPYSLGHCAWFVYGALMKQGSILSPVADSTRLLFATWWIFITILTSFYTANLTAFLTLSQFTLPFNTVNDILAKNKHFVSQRGSGIEYAIKLTNESLSMLSGMATRNLAVFTGDTNDTLNLRKYVEKYGYVFVRDRPAITHVLYEDYLYRKTISYDNEKIHCPFAKAKEPFLKKKRSFAYPRNSNLSDLFDRELLNLVESGIIKHLSAKDLPNAEICPQNLGGTERQLRNGDLMMTYYIMFAGFATSIVVFSTEMLFRYLNNRRESNQWATHGVGRTPNGGLLKPSKWFWRRSSESNKQLLGSSHSNNITPPPPYQSIFNNGKGFQENTSMRRWHHAANYGANGAGGFGVLRPVGNYYGNDAGAGSSTNSAALESTGLRKFINGREYMVYRTPDGLNQLVPVRVPSAALFQYTYTE

>MdIR84a1

MIAAGNDGINAYGYRGFHEKGVDGPTDVEQWMRSWMFSFVHAFEHKLWSADDHQIALGQLACCGKSCELLAFKEYLDFQHLKQAIVIYGGDEAKAYAAEMGRMNNSFLKFFNTNQLKENTDFYQLLRGNSYTVGILMSHASRNAVQDQLLILNYSPSLNMTMSEYLNDNKRYLQNDFMQRKTYQLMTITQDVFNYSFNLKIEDSWGVYNNGTWTGVIGLINSNDAEFSLSPLRYMTERLHVVSYTPVVHVELVRFLLRHPKRTSIRNIFFEPLAVNVWWCVLALIITTGFLLGIHVYTEYHLYWKMKMLQPNETAATFYQLGPEHKVDFVVLTILETAFMQGPSPEQFHANSTRLLLTSVSVFAILLMQFYGGYIVGSLLSETPRTITNLDALYSSSMEIGMEDISYNYDIFNLTSNRVAQKMYKNRICKNGKRNIVTLEEGLQRIAKGSFALHVSLNRAYQLLTDMLTESQFCELQEITFNNPFVTAIGAAKTTPYLKYIKSAVLKFREAGIMKYNDLVWKLPKIDCAALAKDDVEVDLEHFAPVLVFLAFSIMISVWILMLEFLYKRIEKMLHINYENMCRTIRKCLKN

>MdIR84a2

MSWFIVLDDKYHNNHVEKMQQAFGNLNVLLNSDVSVGLKNKSCFIDIYDIYKICQRCKNEKLTIEYKGNWSQTSVLKIEDRFRLPFALRRRNFNNSPVKVATAILDYSPSLNMTIPEYLNDNKHYLYNDFLQRKTYQLLTRTQEVYNYSFQLTIEKDWGSFSNGTWSGVLALLKNHDIEFSVNPLRYMSERFHILSYTPEVHVELVRFLLRHPKQRGIRNIFLEPLANTVWWSVLALIVITGIMLAIHVHAEFQIYWELKRLQPHESAATTLKQLVPEHNLDFIVLTTLEAVFMQGPTPEQFHSNSIRLLLTSVSVFALLLVQFYAAYIVSSLLSEPPRTITTLDALYNSSLEIGMEKARYNYDLFNGTTNRLVDNIFKYRICKNGKQNIVTLEQGVQRIARGGFALHVTLNRAYQLLEDKLSESQFCELQEIIFTNAFTTGIGMAKTTPYSIYLKSAILKFRETGILNYNDLAWKLAKIDCAALAKDDVEVDLEHFAPVLVILALGIVIAIWVFILEHLYKRVANRLRVNYEIVCKTMRKYLPK

>MdIR85a

MWLALWLFGLVIGSSTSELQQHKSLNFYSMAFNSSWMDPKRSHLIEFVGEVFCKSHLKVVHVYYETDISLRYSGQILRDLNRCGISFVALRNDQQNSHLKTISDDGILLHLVIILRDIDQTLDLSIIRKKSAAKHLTYIMLLIQDAHNVTEKWLLSTFKNFWKMWILNVVVVFTDPKQGYIELYRYDPFAKILRHRIALGPDTYNLDELYPKDILNMRGNPLQICLYQDNIRTIFESSGNILGTDGLMSSFLVERLNATPLVRRIRTYGNDSVSQDLCFKETFDELDDMATNIRFLSMESFYGRVESTIVLNRDDLCVLIPKAKIASSFWNLFRSFSISVWVLISVSLAMAYIFCSLIYRNIFAGDKLLLDLLSCIISTPRARLQRSRMSARLFFYVWLVYGLLISAAFKGNLTSYLVDREYLPDVNTLQELAESKYPLATLPRHIKHLNRYLDLNNPYESMLRQKIIPLPDAFFNELIEHNNLSYAYLQKYHISVFRANSRKHSLNGKPCFHAMAQCIVPFHAVYIVPYGSPYLGYINKLIRNAQEYGYLHYWDSMMSAVFRRSRRNGQLQRSDDSEPEVLQLFHFQAVYCFWAMGLLIATVCFAGELINARISF

>MdIR87a

MKYLWIHLLVCFGLKYGSAQGFGMNLMKVAEDDPGQIVCTVALLEKYFHSGEALSGAVLHYTITSASLHLQKSLLQALHSLPKNPWSIVVRESNKRGDSDVPNFILHEKPQCYFMIIDNMDDEDMDEIFENWKLSINWNPLAQFVVYLSSVEETAEEMTDIMIEVLLNFMNKKIYNVNVIGQNEEETYYYGKSVFPYHPDNNCGNRVITIETLDLCDYQDTDKFDEDEDEEEDEEEEGEGEEEEEGEENEGEEEDHSKAEDESGSDDDDDSGKEGGEEDEEEKNTSESEDSNEMPEDGNMEGKEPKFYIEEMYRALFLDKFPKDLSGCPLVAAYRPWEPFIFNEALHDAIPSNNKNEKPLESEDNNAEDDYGDDENYMEGDDNAVESDYKSYEDDLTAIGVEVRLNGIEYKMIQTIAERLHISIDMQVENTNVYHLFQQLIDGDIEMVIGGIDEDPSISQYVSSTIPYLQDDLTWCVAKARRSHNLFNFMSTFDAKAWLLTLTFILTASLSIAMSQKFLKLRLHIMKSYFSINIYVMGVVLSQAVNLPRIPTSLQLCFGTTFFMGLIFSNVYQSFLISTLTTPKSSYQISHIEEIYANRMNVMGSVDNVRHLSKEGETFRYVREHFHMCYNIEECLHRAAVDPKLAVAVSRQHSFYNPRIPRDNLYCFDRNENIYVYLVTMLLPKKFHLLHKINPVIQHIIESGHLHKWARDLDMRRKIVEEIQRAHEEPFKSLTLDQVIGPFALHFILLLFALFVFGVELLVHWLVVQRRTRLKIAKCLHRKFL

>MdIR92aJIN

GFITILTNTSSFLHARYFATRYARLRLKDKIYLFLCENEDPAELLASELLQKYVGAEGNLDAMHLDTFQAENMAFAKNVELYPNKLRDLQERQFPRWRTNIMPFSTELWICLIPTLVLCSLLFHFVKYTGYSCMKGGRSRKRHGLKSFEKAMLEVFAVFIQQPSVDTVLKRTASRVFLAFLLCATITLENTYSGQLKSILTSPLFYEPIDTVEKWSATDWKWAAPSIVWVETILGSNITKEQRMAASFEIRDHDYMYNARFRNDYGFGVERLYSGFLNVGKYITIPAVESKVILKDDIYIDWTRAASIRGWPLMPVLDQHIIFCLETGLYIHWERLANYRFMDRKLQDVLVKIASNEKPKSPPQKLSIDHISGPLFILLFGYLTAFVVFVMEVISSHLKKQLNKI

>MdIR93a

MEKLKRKLTELMTMGSVTKEYNDYSSFISANATLAVVVDQDYMQQQNVNILSHFQKILSDTIRENLKNGGLNVKYFSWSGIRLKKDFLAAMTVMDCENTMKFFKSTRANSVLLIAITDADCPRLPLDQTLMIPLVGRGEEFPQMILDAKVQNILPWKTAVVIMDENLVNENTKLVESVVHESTKNNVVPISLYLYSINERLRSQRKRQAIREALLPFQRHPRESNQFIVFSKFYEDIIEMADNMDMYHVNNQWLFFVLEENTENFDAMAVTQNLAEGANIAFVLNETLPSCETSLNCTLQEISMAFVLSISKLIAEEQSIYGEISDEEWEALRYTKKEKQDDILQTMKEYLKNHSRCSTCSKWRLTTALSWGKSQEHNKPRRGLSENRNKYFEFVNIGYWTSVLGFVTHELAFPHVKHYFRNITLDIITMHRPPWQILKKDQRGEIIQHSGIVMEILKELSRMLNFSYILHDASSLDANEDMVNLNDTDQLLGSLTYIIPYQVAEMLQANKFFIAALAATVDDPDKKPFNYTIPISIQKYSFISRRPDEVSRIYLFTAPFTLETWASLVGVIVITSPVLFIINRFVPVEHLKVKGFATIKNCFWYIYGALLQQGGMYLPQADSGRLVIGFWWIVVIVIVTTYCGNLVAFLTFPKFQPGLDYFFQLYNHKEYEQFGLRNGTYFEKYAATSTRNEFTKYLEKATIYNNLREENIEAVKRGERVNIDWRINLQLIIQKHFEKDKECKFALGKENFLDEQISMLMPSNSPYLILLNEQITRLNQMGFIERWHQTNLPSMDKCNGRGVMRQITNHKVNLDDMQGCFLVLLLGSLGALFVMLLEFLHRRWQLKYADKTKQTIFSN

>MdIR94e

MIMAEARIDTAKAEPSVNGGNDYTVIEFLKDLKDLHNYDNVLLMHNQNTTIATKFYTNTNTMAYGNGSSTAAAGNISFIEKTLNVDASGRSTSLPFVAHLMQQVQVPVLQLNEWQHFNLKLRVPDNLLAIVQIDINGGGGGAGDGGGDGIKITLDHHAGLLQNLSKCLWRMKVAKVLFLINGPAMMNDEMLASDRGNEDVHYALVEQLFQHCWRQKLLNVAAIMANYQKTKLLYRFNPFPEFQMETVPLAIGRTQQQEEIYPQRLDNLLGYNMNVVIGGSDPRIIPYEKNGKLFVGGFVGHFVLAFAKRYNCTLQEPLPYNPKIPLPSQELMRAVRNGTVEWSSGVTFPEIPFRGYTYPYEIINFCLMIPVEADIPGYEFFTSVFKGETYVFFIVTLVIISMVLSAALFIHGYRPDLFDIICHDDCLRGMLGQSFSELRNPPGIVRAIYLEICILGILLTTTYNAYFSTYVTKAPKTAPINTLDDIMASGLKNIVWEPEYNEILSRVPEFKRYAPMFLVEPNYRKYLELRESFNTHYGYIVPTTKWTIVTEQQKIFTTPLFKQRPSFCFYNNIPMCFPIHENSLFIELMYKLMLEVSQSGLMNMWMEHGFLELIQADKLQRSDLSQKKEFEAMTVDDLLYIFIFLAVMFVFVVLVFIGEFVVFHREKVWKGL

>MdIR100a

MSARTTIKVLLLLMYNHITTSMDWQNIQEIVNSLDCLYINVATVAEDNRIYEEIYTTFEIPLENSKGNFKDTKCPNKMLEIYNVEMLSTLLATEASREGFLFLLFINEITDLWPVVLNESKNYWSWQRIYKVVYITPTHKRFFHPFVRDVNGNFGSLVDIEEYNIQKLFHNMNGYPMKVYIFDSVFSSLTADAEGKRLTGVKGTDGKIAHFLESYLNYSMQLQWPDDEFFGSRLDNGSFNGALGRLMRNETDIVLTGFFVKDYLANDIAFSSSVYMDQLCCYVMKAKRIPASILPLHAVDESIWLAYTIVGILASFFWVLLRQANLKLNPNEMRNLRGADCRWYTVFIDAWALWGRMIILRFPPSNAERMFAISLCLVSVIIGALFDSSLATVFIKPLYYKDITTLEQLNKANVRIFYKHPAIKDDLFTGHSSPIYQSLDQRMLLVGEPEERLISIMAKRGKFAAVTRAYSLSLVDIYYFITKKVYMIPECPKAYHIAFPMQKHSPFEEEINVALLKLLAGGFINHWIEMQQYVARSRIHLFEDYAGESEHIWKILNINDLQLAFYVLSVGLIASFFLYICEHIYYKCKLRRRRT

>MdIR101

MIRKFFGLLILWHSLKSVRGDSHPLVKGDVGVSAELLEYTHVALNLTKLYISSHTNALVIMEKCSGLVCRRQTLNHDFLLEYFLRNLSCDISVQLEFGRPDVRPWDYNLFVIDSAKAFEALRLQLPGPSKNRQFYFFILLTCSSAHPTYVKQQMYKIFKACLQIGVKNAVIMHRYSAGAYISFYTYYAFGRFHCWDDITIREINRFENGSLSGNYLFPKQLRNYHGCTIMVSAHLMAPLLSFNGDFTNEQHLRDKSRIAGIEGDILKTVADTLNMNLKFRFPLNLNKKFMFSNRTDSLVDLTENRSEIAIGGLSPILPDTQQFTYSSVYHTTPGVFVVKRGLSFGPLKQLLKPLDTNIWILIILQWLVAVVLIQLVQRFGNLALWNFIFGPHNRHPMRNMFMSNLGYPIPTAAVPGRNFARFLLMAWLLLTFELRNAYQGKMYDSLRLAKRLPVPRTIDGLIRHDYTLLSPEFNDFYPHNKTRIMSNAFMRLHRINSSHHKLTAMALLDYLADFNARNLHNTSLTYVEEDIYSFQCVMMFRRYSVLPESINPKLKLLTDAGITDHIAKRYVRWQKQRGNRRGAVPTGIQEITNHKLRGVYKGYGVLCACAVLVFFLEMLTFKFGILKRIMDYLN

>MdIR102

MQKQIILLSLSALCCIGLTIAYSTSSSRDLILDDIDDPQNVLMEYGQIALYMVMRFISPRTNTLIIMENCLFYCDHHRLYHSTVLKFFLNNLNYTMATQLYFGQPDERPWDYNMFVVPTWREFEALQVNIPKTFYDRQYYFFIVFTWFMPYRDLYFENMRKIFEICRKMNVKNVVIMMHPFAEKSISFYTYSLYTGEYCNTELMIREINRYRNGKLQNPFLFPDHMRNFHGCKLTVCGHIIAPLLTFNGDRNNETHLKEMHRLAGIEGQILKIVASTMNIILEYRFTSDDYHVEGDANFTGCLADLYENRVDMAIGGLGALIPNSQKFSISFTHHFSPYVFVVRGGRPFGPMTQLMNPLQLNAWQALLAQLLIIIALIYWLEKRGKRSCRNFILGAHNKYSIHHLFVTLLGSPVPSYAVPRRNFARFLFVAWLLWSLELRNFYQGKMFDTLRLAKRLPTPKTIHELIDKDYILLSSHYKNFYPENKTIIIPQNSKPLTILNGMENGAFTTTAILDFMANHNMINFKSSTLTYVDEIIYLYHSAVFFPKHSILLPSFNRKFKLLSDAGITSYVARKHVHPYFHNTKDHINTGDVRQITHKNLIGLYYIFIVMNGMALLLFLVEIGTKRSKVLKCFIERLN

>MdIR103

MQKSFALITCTSLLCLALSYSFETHFYSRKLLLKDIEDPQTTLMEYGQVALYMVMRYISPRTNTLIIMEHCLHNCDDHRLYHSTVLKFFLNNLNYSMATQLYFGQPEERPWDYNLFLVPTWKEFEALQVSIPKTLHDREYFFFIVITWFWPYQDFFDNDMMKIFEICRKMNVKNVVIMTKPLVGKVISFYTYSLYNGDYCNTELAMKEINRYENGRFQNDFLFPDFMKNFHGCKLTVCARIIPPMLTFNGDRSNESHLKEMHRLAGIEGEILKLVASTMDIKLEYRFTQSYFNPGRNDSFTGCIADLYENRADMAIGGMGALMPNGHYFSASYTHHTSPYVFVVRGGRPFGPITKLLNPLQMNVWQVILAQLFIIIIFIAWIERRGWWTLRNFILGSHNKYSIHNLFVTLLGSPLPNYAVPRRNFARFILVAWLLWTLELRNFYQGKMFDTLRQAKRQPTPKTIHELIDKDYTLLSSIYRDYFPHNKTIIISNTVERLHVVNSLDMPFTTTEVLDFMSYYNMINWKSSTLTYVDEVIYMYHCVVYFPKHSILLPSFNRKLKLLSDAGITSFVARQYIHPYYRNLKGQINTGEVKQITHKQLIGLYYIYVALNGVAVMVFILELGWKKIGTLKRVIHRFNKIKC

>MdIR104

MNPLMELSLNSSQSNKNLPLILATVWIIREYFATYTVSAVIIGQYAISDQGRQLQSDIMDEVLRTISNPEVIIKYLVEGEMYPQDDSEATMSAAEIREKFFRYYSNPEKSIWFLDSIQAYNKFEANLLNPNHRYHRNGYFIIVYTGSEATRLANIKEIFQRLFWIYVTNVNVLMMVGKHAFVYTYYPFAPDKCHSSQPEYLMSFYDIEKKPNFTTAIKLFPSKVKNMHRCKLSVATWNFPPYIFLNDDEKEMELTFLRGIEGFVITLLAERMNFSIEIKQPNPIGRGVIYPNGTSTLAAKMILDREVNITISAYTHNAQRADIMLASTSYLTSTFVLAIPDGQPLSPFERLIKPFRYIIWSCFSSSFLFAILLIYFIRLLGRSDLMDFIYGQDNRKPITNLIAALFGVGLVNKLPYRNFARYLLTVWMLYTFVLRSAYSGELFKILQDGSSRNVMSSIEEVVVNNYTIYAFATLEKVIKESVPEAKVEMVNTTEEELLLRISRGSADDKIVLCSLDLTIQYFNQLHPHARVRILREPVLTAPLIFYMPRHSYIKLRTGNLILDLIQSGLMKRYRRMILYSSTKIHKDHAEPTKLSIHLLFGVFCTYGAGLVFSTIVFVLEMFSKRCRSLAVIIDFLNM

>MdIR105

MAPFVKILENNNQSSSSLNLPLILATVWIVRNDFDVHTASTVTIGQYAITTHGRQLQNDLIDGVIKGTMSPCPVIMCWVHSEMQIMNDEEKIREFYRSYMNRERSIWFLDSMEAFRKLEKNLLNPYFRYQRNGLYILVYTGLESKRFFTIRNIFERLFYLYITNVNVIMMVEQYAYIYTYYPFTPNRCHSPQPEYVMSYEDIESNENFTLSEGGLFPNRVTNMHGCPVSVVTWTYKPYTYVKRDRKTGAFMGLYGIEGSVVTLLSKHMNFTIVIKQPNPLEPGELFPNGTATGATRMILEHEGNITVMSYILYSERSKRLQPSGSYLRQFYVLVMPLARPLTPFERLLKPFQCLVWFCFDTSFCFAIGFIFYIKLLGKSNLMSFVFGKGNRIPFTNLLNTLFGGVMNSGNMPQKNFARYLLILWMMYTFILRSAYSGELFNIHQDGTGQNNLQTLSEVVANNYTIYTFGVLNSVMRNAIPGGHIKNFNKVETMDKLLRTIGEPESRDKIALAVLDTTANYYNQKNPRRRVHVLKERVIPAPLVFYMPRYSYLRGEASRIVHKIVESGLVRHYTALNLYATESFDKRRESADLSLGVLVGIFSLHATLLLICCLIFALEMLSTKYKRIKKIVDFLNS

>MdIR106

MSPLEKVLLNSSQPTSNVLPLVLAAVWIVRNDFAVYTVSSVTIGQYASRPRNLYIQNDLINHVLRDTMNPFGIIKYLVEGEIYPHEEYDESVSHEEAMERFFKYYANREKSIWFLDSLEAYLKFEENLLNPNRGYHRNGFFILIYTGFEPERLVTIRNIFRRLFFLYVVNVNVMMMVGKYAYVYTYYPFTARKCHSPQPELLLSFRGIESNPNFVLKKGLFGSKVANMHGCPLSVVSWDYPPFIFVKKDPKTGAFRTIHGIEGSVISLLSEQMNFSIYIKEPNPREAGEVFSNGTATGAARMILQQEANITAIAYIYSPERSEKLLPSDSYLTLTVVLAMPLGRPMTPFERLIKPFRYIIWSCFSSSFLFAILCIYYIKFLGRSRLMIFIYGQGNRIPFTNLLSTLFGGVVFGQMPQRNFARYILSIWLLYTFVLRSAYSGALFQILQDGRGKNNLQTLDQVVEHNYTIYTSRVMESVMKFALPKATVRQYDEVNTLQNLLETISEPDSKDKIALCLFDLTVKYYYQLNPTRRVHILKQPIMSTPIIFYMPRHSYMQLHTSGIILRLVQSGLIKRFVKFNVYASSRDNVRKSEYVALSLDVLIGLYWVYGFLIFLCILIFILEILARKSGKLRKVMDFLNL

>MdIR107NTE

NFYERVKHSGSQNLSYTIMYISSITTLDVATISKVFKIIFPMSLLNVGLVIPLSKDNIIMVTYFPFTPTECYSVAPVTINSYDTVKQEWRNKNYFPKKSKSFYRCPVTCATYEEMPYLGLSLNRTTKRVNSYRGFEGELVKYSASNLNFTTIVYLMNEEEINESFDERGLVFEKIFSKSADFAIGAFYYRPHLNESSPYSQTLYYYLSHTYLVTNVFNIYSMYEKIAYPFHLGLWYLIGLILALSSLLIFTCESGRRWRKQRNFIIGENNRTPQYHLFVLALGATVSSTQLPRYNFARFLLMCWLLGSLVIRSAYQSGMYEMLRDNKHRNPPQTIADVLKQGYVVLLRGYHKSLLNILPDMKNVRELNVSILQAFPQLATASERTAVFSQYEYYGYFGKTNLATWQKLHLVNERIYTQQLAMYVRLQSYLVTELNAQIANAQYFGFINHWVNKYYGRPVAAGGHGHQGEESQTNILSMNELGAVFMILLWLHLAAFGVFVMELLWHRYGRKGRTMCH

>MdIR108

MQNFPDPELPVLLQHAIAACCSIVADYFAAKSNSFMLSTNIEEKILQPHIRDFINNVLLCLDSIKVEVENLHGERGRPSFNRKYNLIVVDSVEALRRLDPGHSTRDYDIQEVYLVYLMNASRFPNLEIQLRDIFAYFWQNYIVNVTVVIVNTRTGSVEALTYYPFYNNVSCKLVHVQQINSFLGVWVKPLHENIFPEKIANLHQCPLTVAVWETPPYLSYRPADNGFYEIDYFEADLLLVLEEKMNFTLDLKEPPNNEQRGKVLENGTSTGALRMLQERTADFSLGSFRYTLERSQLMTAALPYYQTWQIYGFMRTAQPYTSLEILVFAFDDKTWLCLILSIQIVMAIGYLLQFQYRKFTLVRIILGHPRPTTPVTNIVKLFFGQGLEILPRSNFTRFVLVLWDVYGLLMRTAYQSMLFQLLKGNLYHDPPQSLSDLIDKGCKLVTTEGTFDSIGTVPRIEQGLIEVIKIKNTSEQSTFFYMEENTREGNCLSGISPMDFLTYHATREKKRGVFFALPEKIFTQHITMYFSKHSFLINRINFLLMSLRSMGLIDFWARQSLDTSYFDAPNDVHFVAVEFAKVKGVFVTYLALMLVASIVFCLEVILFYFKKML

>MdIR109NTE

LQERTADFSLGSFRYTLERSQLMTAALPYYQTWQIYGFMRTAQPYTSLEILVFAFDDKTWLCLILSIQIVMAIGYLLQFQYRKCTLVRIILGHPRPTTPVTNIVKLFFGQGLEILPRSNFTRFVLVLWDVYGLLMRTAYQSMLFQLLKGNLYHDPPQSLSDLIDKGCKLVTTEGTFDSIGTVPRIEQGLIEVIKIKNTSEQSTFFYMEKNTREGNCLSGISPMDFLTYHATRENKRGVFFALPEKIFTQHITMYFSKHSFLINRINFLLMSLRSMGLIDFWARQSLDTSYFDAPNDVHFVAVEFAKVKGVFVTYLALMLVASIVFCLEVILFNFKKML

>MdIR110PSE

MNYLHFQLMVIVVGICFAAKGGEGQTNITLLSEIIENIFKNIFYPNGISVNIVNDFPNNYDFQQFNIDLIEEILKRNSIPISLNSQVVTELDKYFRLRCIIVQSSKDVGYDLKHSIEYSRSLKTIQTTGMKFLIILNNKKRHSQQLHEMEKIFELLFNAYILDVIIITPGLQNVQMYSYFPFTQHHCSNTKPVLLFDIGGLVGHSQLGYNDLFPKKISNFHQCPMNVVVWNIPPYIEIRKSEEGVVTLDGFDASILRIIAEALNFSVLLTPNEPPDLISVHILPNGSAVGVFKMXAVPIRPRPLCIGCIACDLTRMQITSGSYAYFIPKFVIILKNSIVIKSRELLVRPFTKSTWRLVICISVLKLTLLPIIRRRNTKLYSIFLMAWLYILFALRIGYEGVMFHVITNPPFQPLPMTLEELFNHNFTLFTDYSTNRLLELMPSLKAISQIVNCTPMELLEQMDELPSKSAILSTTAYVSYYMKDRMZNISQYSLLHEKLLNGLNCIYYPRGSFLAGEIDGVLKNVISSGIRNKFVREMGLHNLPNATGNWQHMESNHDDPNFTLNFHFLQVTFKALLQLHFIAIVLFLGEILTHKFIARCKIFENLKIKFSKI

>MdIR111

MNYLQFMLIIVIQVGKGCESQPDFQLLSAVIQNIVIGALEPNAITLEIITNYSNSENQKLNTDLVEEILNINTKTEKPIPIILNPPWDSLMDDRLKVRLLMVQRAEDVRLDLDGSVKYYRSLKSVLTTTPKYVVIFTSYQFNEMERIFQLLFDAYILDVIIVMPHTNQVQIFTYFPYHSNRGCSNVLPVLVYTTDGFEKQQPITYDTIFPRKTLNFHQCPIRVVVWNTPPYIDIIGDPTGTVSLQGFDASILDILSKELNFSVEVVPNDPPKIISGVVYPNGTAAGVFKMLQQQRLNLTIGWISCMLNRLEVSTGSNAYFTDHYVIILKNNIMVTPNELLLRPFTKSTWRVLICVAIVKIALLRMIRKHKIKFHSILLLAWLYFLFFFRVGYEGVMNHVITHPPYQPLPQTIEEFIEQNFTLFADDSTNRILDFIPHLKEISHVIHCNPLELLEQLDSLPPKFGIISTEAYIGHFMKMHMENRSDYSILEEIILTGVNCIYYPRGSFLAPVVDDILDWLSNSGIRDKLVKEIGMENVPHSTAVLRQFVYGGKHFKLNFNFLQIVFKTLVIWHCIAGGIFLGEIVTYKFLSKCMLYKKVEIKLQN

>MdIR112

MSLLLNTTLDNIEPTFNLPLIMAIVWIINKDYAIYTTSPVTIGQYAINWRNRRFQSDLIDEVLRRAQTPEAHIRYQVEGEIYPKDTTEEHMSAEELLERFYKSYANREKSIWFLDSLEAYKKFERDLIDPKQHYHRSGYFVLVYTGLEADRLSNIKEMFRRLFNIYVTNVNVMLMVGKYPYLYTYFPFAPNKCHSSSPGYFASFKGIEKNANFTLGKNLFPTKVENMHGCSLSVITWTYLPYIVVERDEKTGELISLHGIEGSVISLLAERMNFTIKIKEPKAKDRGDIYPNGTATGAAKMILEKEANITIISYLYNKERADVMSASASYLNLPYLLAIPQGRPLTAFQRLIKPFRYIIWSCFTSSFFFAILFIYYIRFLGKSKLMDFIYGQGNRLPFTNLLSTLLGGSVYSQLPYRNFARFLLTVWILYTMVLRTAYSGELFNILQDGKARNNFQKLQEIVERNYTIYAFPAVETVLKFLDPQPSTGTVDSVNSVPVLFEKISNPNTKEKIALCLLEYSIRSYNQRNPSRRVEILPETVVTSPIVFYMPHHSYIRAQTGVLIMQMLQAGIMKRFESIYLYVAWKPQRSQGEPTRLSFHLLLGIFVVYGVLLIFCVLVFLLELSSARVGWLQAVVNFLNL

>MdIR113

MSTVYCSNVPKLRLFIIFSTLISHATAIGCPQVLELNHQEIAHNISEALVEMIDSFFLEKYGRRSFNVHIKVQNPQNRHFFNDIVRAMWSLLDGRISIYLSNDIPIPVSNQIHFSVLLVDSTESLEYLYTNIIKYHLFIEGSYFIVLYTLPSPNHYYDELYRSLQTCLDAGISHANVLVYAGLNSILLFHDEPFSEFHCNANVPVVNNKFISGQWNHTGFYISKASNLYGCPLVCATWEDMPYFEVLSNETSAKNQRFKGLEGRMLDYLSERMNFTVAIRWMNDEEINRTLYDESGMLEELFSTGTDFVIGAFHDKPTSFYDTFTPTTNYFLSSFYFVVSAKTDPYDPFVKLLLPFKTEIWFILILLLVIGNVILFSITQVDRQIKYLVLGRKKQRPIYNMVIISLGGPVARDPKVPFSRFLLMVWLLASFVLRTIYQGFMYHFLRHDIHKPPPKSIQQLREENYTILMSEVVYQGIKHLKALYDVAVVLNDSEVESFAILNEPEKYGFDRKTAILTAYEYYGYFKYLNQNNNDFYLVPEIFFTQQLSIYMMKNSMFLNRFNMYITSYTNEGLMHRWEKYLIFKNTFRKLQADDQPSAMDLYQLCGALNLLGICLLGCVGVFVAEVVVHRVSVWARKKRRRWWGPKRPRKNQWINEGSEF

>MdIR114

MSTIYYFILISFLVNIRNSIATNCPNLSQNQEIAHNISEALVEIIEKFYISKYRLRSFSLHIKVQSPRNSYFFEDVVDSMWKLLNGRIEMILSNGIPIPVSSNIQYCILLVDSKESLEYLYRNIITHHLYIEGSYFIVLYPWLAPYHYYDELYTASQLCLDAGISHANILVYAGQNTILLFHDLPFTEFHCWANVPVIDNKFSHGQWEHMEFYIPKVNNLYGCPLVCATWEEMPYLEILPESTSTEHFRGLEGRMLDYIANRMNFTVKMRWMTEDEINRTLYDERGILKELFAEGADFVIGGFHYKPTSFDDIYTPTTTYFLSTFYFVISAYTDPYDPFSKLLLPFRSKVWLILIWMLVLGNALVIGVMKTKCHLKYVLFGRHPHSPIYNTFVISLGGGISRDPKIPFSRFLFMVWMLASFVLRTIYQGLMYHFLRHDVHKSPPKTIDALLRENYTIFISEYIYNSVEHVKKLRERAVVLNTTELESFPMVNEPKKYGFEKLAILTTHEYFGYFRWFHRNNQGYYLVPEVLFTQQLSIYMMKDSIFLNRFNMYIKSFINEGLMHRWEKHLLTKNTFRKMSSDEQPKALGIYELYGAWNLWMICLAICFGVFVGEILVHYLGLWVKRRRRRWRKSQMKYQWID

>MdIR115

MTTISYLIVSSLLLLFLVVSLKRNSSDAANCPLYSLQLNHHEMARNISEALVEIIEKFFIGKYRRRSFNLHIKVQSRRNSYFFEDVVDSMWRLLNGRIEMILSNGVPIPLSSNIQFCILLVDSTESLEYLYTNILKYHLYIEGSYFIALYTWPLPNHYYNDLYTSSQLCLDAGIAHANILVYAGQNSILLFHDLPFTKFHCMANVPVIDNKFANGHWEHTKFYVAKANNLYGCPLVCATWEDMPYFEVLPESKTPSRDHYRGLEGRSRMNFTVKMRWMTESEINRTVYDERGMLKELFDEGADFVLGGFHYKPTSFFDIYTPTTTYYMSTYYFVISANTEPYDPFVKLLLPFRIKVWLVLILMLVIGNVIVFAAIQTNCQLKYLLFGRKPQRPLYNTFVISLGGPISRDPKIPFARFLLIVWLLTSFVLRTLYQGLMFHFLRHDYHKLPPKTINQLRRENYTILMPEYIYNGVEHLKKLHEKALIMNGSELESFPMLNHPEKYGYEKLAVLTNHERFGYFKWFQRNNQAYYLVPEVLFTQRLSIYMMKNSIFLNRFNMYIKSYINEGLMHRWEKYLLTKNTFRKVRSDDQPKAMGINELYGALDILLICLAGCILVFVGEICVYRMGCWWKRLRRRWRRRQMKYQWVD

>MdIR116

MFLISVATVLNLLLIEQIFGKLLPINQVDDRDVDEMARCVRHFNAEVFLGQTSQVAVVKSVESSAANGYFSELLSEILKPWNDMKIRLSDVGVDYRHEYDYFNILLIDSYRSFEKIQPGPIAKTKDFSEYYLIIYHANSSTSQNEMQKIFEYCWRYYMVNVAVLLKLENQTISLYTYYPFTLRQCHKPQIVTLSHGKSIRNLTRLELYPEKFNNFHNCTIMAALWNVPPYLMLPKAGTSFHGMEGMEGWLLKVLAELFNFHLDYKTPPNNEQRGLVKKDGSVTGAIKMLNDHIADLSLGSFRCTLERSTALSPSATFYQTMQVFTVLARRQPFQSFEILTYPFDIYIWTMWLMLTLLLLVFTFIFERIHIPTLHFIYDVRCTSSININIIATSLGQPAFNTLQPQRNFARYFTTMWALMTFLLRSTYQSSLYDFLNSDKTVQPPNTAAELAARKFTLIVNVATSDSFSGIPILRNKQLDLKIMNITDAGGYPILEANPDKNYATGTPRDFLVDYVNSYHKYGVFHVLEETIFSQQLCVYFSKHSYLLPSFDRVLLNLRSFGLIDHWARQVFDDRFLEQTGEERIPLALGISQLWSIFKTCLIIDLLAVMVFVVEIVYYKCSHRKLNSSK

>MdIR117

MNVTNLFNFGQTRLEESQINQYDMNAVVAQSLCRIIQNFFMEMTTSFMIVISTRRRRTFYFFLNVLEFIFDMIPDLNAQLVFVDHKNPQRIEGPRFYNLLLIDSYEAFLDIDPIAYTKQYDTSEYYHMFLMQNDLIILEEMEKIFRYCWQNQIVNCNIQIQNRKSELHLYTYFPFGWGTCNSTRPQHINQFVDGKWLRRPYFHAKTNNFYGCPLIGVVRCTRPYVYYDENGEFTGFEVAIVKEFARVLNFTLILKEAEDDDRNYPALRGGLLMLANRTADFVFGYYRKRSLTADLYTNTAPHYQSSIAAVINLRAHIFNTFEVLAYPFRLYTWSAIIGCGASILLVTRLVRFQRPKSMRTFSMLTSAFGLPVRETIKHRHSYFMLGPWIWGTFLLRSIYSGLLYYLFSNDIYHKLPLNLGDATNQNYISVLNRFTFYDVANIPFYHDRSRHNLQPIILNSSDELAAIKYVEENLSRNLYAVISKEFLMHYAQESGKVALFYVIPETVMKQQITIYFTKHTILAYRFEKMIMDLKSSGLQRYYIKRYFDSKTMMNSYKEDDEMIEQKDLLGIYVICGALQLLAVLTFLLELLSQKITKLRVLFD

>MdIR118

MVGACLLHIIGYYFVRWSKSFILIISVEREDSSAFYNDVLDRTFANWKQYSLQIVNVHRGQKRRVRGTRDYNMVLIDSYESFVSADLVAHTKNNNHNEYYYIFLKRSDAMLWPVMQQIFEYCWQNHLINCVIQIQTDRGELQLYTYYPFTRWQCGKAQIVRITGLNASGKMSREMLFPSKLKNFYGCPLRVAIWHIPPFMSLSTDAEGNVQLDGGCESRLLKMLSDRYNFSLDLRVFDDDTRGNVFPNGSTTGVLKMLNDRELDFGIGSYHQNALRNSVATSTVNYYQSIISVVMLRSALRLSDSKALIYPFQPNTWLILFVVTIAVILGVYIFRHIRHTTVVKPFTDVFISMLGMPFVHMPPFKELRVFALSWIFFTLIIRSAYLGFLFHIIRSHLLSNPPTDLNTLISRNFGIIVSERVNHIIANISELKQLNHTILQKKPETYTLEYLLNLPPEEGNHVMGISAVDFLQYQIRARRLRDVVKIMPYDLLGFKICIYLAKHSYLSDQFNELLIWVRDSGLIEYWKKTQLDSGYVNGKWQAEDELFDMAELKTAFMAVGIGDVIAILIFLVEVFYHKYFDHDDDNDVLVFIN

>MdIR119

MNYSAFLVGHDEGLFRQDESINRFVAKALRFLIQNVFETLTSTYAVFIASRDQPTLHWMNYIMMELFSITTAMTVQIVQINAGQKVKFEVSGRKYCNILLVQSYRDLLDIGLESINSAYDGMEYYLIFLQARDAMIPREMQLILQYCLDNYWLHCNVMIQTAKGEVLMYTYFPYTAQDCYKAKPQFIDYFDGERFQNAPLFPDKLNNLHKCQLTASTWPQPPYVAMTYLDDGNLHYSGMDINLLYGLSAHMNFSLKFEYKDDERIKFVIRDRQVNMSMSYTRRSLELDRIGSSTVTVYHTTLVAVVIQNPYPLSSLKTLVFPFEITVWICLLCSLLMTIAINQTQRHTNPFTNLNFAEILLGLSTLYRPQLKWHSLSVLTWLWSSLLLRSLYQSMIFFLYNFDIFENLPKSLDTLAEQGFTLICSRKTMTFVRKIPQVEENMLRTIVLNSTNEMYQLFYLDKISEGNYAAIVDKEIARFFIDNMAPKNNLKILPFTVNSIQTTIYLPKHSFLIEAINANILRFFAAGFQVVRKLHNRSLENPNNEDSQRTISEMSFMHVISVLEMTAILYFLSFVIFLLELYSKKSTFLQKCFEKVL

>MdIR120

MNYSAILLSSDEGLFKQDECINQFVVKALKFLIRNVFESLTSTYAVFISSRDEPTLRWMNHIMVELFSLTTAMTVQIVQINVKRKMALEMHGRKQCNILLVDSYQALLDIGLASSNAFVDGLEYYLIFLQARDNEIPREMKLILQYCLDNYWLHCNVMIQTAKGEILMYTYFPYTADHCYKAKPKLIDFFDGERFKNPPFLPDKLYNLHKCPLSVNTWSQLPYVAIENLPNGTLHYSGMDIQLLKALSDRMNFTLKVKYRDVEKLISAISERKVNMTVSYTRRSLTLDRIVSSTVTTFHTTLVAVVIRNPYPLSSMRTLVFPYKANVWICLLCCLLVMICINQMRRQTNPMTNLQFLEILLGLSTSYRPQFKWQSLSVLTWLWSSLLLRSLYQSMLYYLYNFDIFENLPQSLDALAQQGFTLICSRNTMRYLEKIQQVEENLLPVIVMNTSNEMHTLTYLDNCSKGNYAAIVDKEIAKYFLNNMESKNSLEILPFTVNNIQTIIYLPKHSFLIETINDYILRFFASGFQLAWKIHYTGVDHPNSDESQRPISEMSFMHVISVLEMTSILYFVSFVIFLLELYSRKSKFVQNVFDKLL

>MdIR121

MNHSAFLLSYEQRQFMVKQDDRINQFVAKALRFLIQNVFETLTSTYAVFISSRDLPSLHWLNYIMMELFSLTTAMTVQIVLINVKQKATFEMRGRKYCNIILVDSYQSLLDIGLASNNANFDGLEYYLIFLQARDNVIPREMELIFQYCLDNYWLHCNVMIQTAKGEVLMYTYFPYTAEECYKAKPLLIDYFDGVQFQNSPLFPHKLYNLHKCPLVVNTWPQPPYVGMQYFENGTLHYFGMDINLLNALSEEMNFTLKFEMRDVERILYAIDERKVNMSTSYTRRSVILDRSGSSTVTTYHTTLVAVIIRSPYPLTSIRTLVFPFDTDAWICLLCTLLAMITINQMRRHSNSMTNLQFIEIFLGLSTLYRPRLKWHSLSFLIWLWCSFLLRSIYQSMIFYLYNFDIFQNPPKSLDALVEHGFTLICTRKTLQFVENIPQVANNMLRKIIFDSSDEMQQLVHLDRLSERNYAAIVDKEIAKFYINNMKPKNILQILPFTVNNIPTTIYLPKHSFLIETINDNILRIFGAGLHETWTLYNGAEDYPKNDEPQRTMFDMSFIHVFSVLELSLILYFVSFVIFLLELCSRKLKCLQKLFEKVH

>MdIR122NTE

LQRRETNITAGFFRRTPERDDLATSTYVTFSVPLAAVVVRRESGHESLNVLIFPFDMPTWVLLIISSLILIIINYFRQKNVRSASTWQIIESLLGLPSVRIPERLSPRVTFIIWMLSTFVLRLVYQSILFFVYRTQFYRQPPTTVIDFAVSGYRAVCTQPTAPLLTYIPQFMDNSLPLIVLNTTDEMAPLRYIDKNSHENLVAITVKDFVFYYVHTESSRSRVFILMRMSLNDQKITFYVPKHSYLAERLENCILGYHQMGFMELWHKLTYESFRISQSSYSTKYEAALLVNLRQIMSFIYLVVFLQSASIVIFVLELLSKKFDFLKKLF

>MdIR123PSE

MNLGKFTIFLCNCYSIFKSVTSEICLKQWGIQLNIANQSFEKVVADYFRTKTHILGVAVYLEYSZDWMHEIERYITLAFAGRDSMKFETSSQGNKQSLLYGYNLWYVDSYKAFCALLPAIGRNEYVQKLGQYMVVMKTAKHLNGDQELQRIFQHAYEHRILDITVAMYRKRFIFTLYSYDVFHPSKCRHVVIQKINTFEGGHLTRSDIFPIKLQNLHKCPIDVFVHLTEPYFNYSLDVVNGELTNFWGLEAWILRIMAKKLNFKLRLQQSRGATIGLVFENGTITGPFLAMTQGKIDVLVGYYHSKVRARRFGVSMPYLLTPLVLVIPKREKRTFEGGWLLVPFQNDVWLLLLVSLMFGLTTFLVLRYTPNNAVVAQNSWLDVVGLALGSSRNIRYHSMGTKYFVMLWTFGFVIVWGAFQGKLYGAFHIDVVPPAHTVEDLVADNYTFHIRRYFRGDLIEALNIPPTQIVFTDVSETQSDFIAQLQAPHPYAIMTDYWLFQNFLKTHNMHDRFEIIPHILIWNQMCAYFRPKSFLIEPFDRIFDALHSGGLIKKWLEEVGEQIQVSVSVKNVPNTEPEPLSLTKMLIIFKGLLVLHVVSLLVFMGEIVLSKKNLNRKKVLRKIRNNIKNKIINH

>MdIR124

MCRHKFATFLWLLPSILQAVALLNSLEQWGIQFDETNRSLEKVIADYFRAKTPLLGVAVNLQNTSDWVYKIEHYLSLAFVGKVLMKFETSTEGKKQSFLFAYNLWYVDSYKAFSALLPVTENNEYVHKMGNYMVIMKTADQLNSGDELKKIFEKAYERNIIHITVALHVESFTFNFYTYSIFQPGKCRNVALQLISQFKDGRLTKAELFPMKLQNFHKCPIDIFVRVSDPFFNYSLNGAGKEIKHFWGLEAKMLQVVAEKLNFKIRLQKSRDWTIGRLYPNGTATGAFLAMSRGKFDVLAGFYHSATRARIFAVSIPYMLTSSVVVYPKRKQSLAEGSWLLAPFQGSVWLLVLISMIFALTSFLALRCAFRKTNVIQNSWLDVVGLVLGNARNIRYSIIGCFATLWTFGFVIVWTAFQAKLYGAFHIQAISRPYSVDNLIANNYTFHIRRYFNGDLIEAMQIPPAQIVYTDKRENHSDFFDHLHNPHPHAIMTDYLLFQNFLKTYHLYDRFEIVPQIVVLNQLCVYFKPQSIFMEPFDRILNAMHCAGLIKKWLSDIFGHPHGPTSNKMVPKTVPIPLSLAKMSVVFKGLLILHAVSFLVFLCEIFMHTYFRRIEILKK

>MdIR125PSE

SLAVPINGMEKFDIKIIEGNRSLEHVIARYFQNKTKLLGVSIHLEYSZDWLYDINYYLTEAFRGIDFMNLEISINDDHQRLLHQYNLWYVDSCRAFGKILQLIGKLPNVKYMGQYMVVLKTVQQLEDYNEMQRIFQQAIRRNIIDITLAMYLENSKFDFYTYYIFQPNLCRQLKIQQFVKFKNGQITTNQLFPRKMKNFCKCPITVFVHPIEPLFNYTEEGGQKEITDVWGLEARILRCIANKLNFKLHFQSSKGGDIGLVHENATVTGPFFELSERKFDILMGYYHSLSGARFFSVSMPYLLQPAVFVVRKRESSLLKGGWLLAPFEGTVWLLLCMLFVIVYITLFVLPRMVMKKNSPICMWLDIIGLALGNSRTISHRRPGSRFFVAAWTFGFLIILRTFEGKLYGAFHSQINTPPNTVKNLIRDNYTFYMRRFFQGDLIRPFNIPSSQIVYTNVPENHSHFQAQLHSPHRLVILTDYWTIHHLLRVHQLHDYFEILPNIVVLNPVCAYMRPSSILIEPFNRILYDLHSGGFIKKWLMDVTGKFPMSIAMAERRRKYSKLEPVPLCVAKMQIIFHGLWAMHLMSLLVFFVEILWHTFLKKRIKKFRQ

>MdIR126

MKVLKFTLIFFSLQFVHTSGYDASLKKWKLFLYRANRSIENTIRAYFQRRTRLLSVAVNVESCPSWEIVIYDYLTSYLKGTTSLKYEISTKRYHQNDHIYSYNLWYVDSYRAFSSLLPIIDDYYVEKMGKFLVIMQTAKHLQRQREVQRIFEKAFKRNIVDIVVAIYAGNSTFHWYTYDVFRPGHCRQVMPRKFNTFRKGILQSKEIFPNKLKNFHQCPLNIAMRPPVPVLGRSSYMTHALDEKYWGMQGETICLLAKKLNFQTIRHPINESLVSEVYKNGTVTGVFYDLKQKKFDILMGYYKYLTRTRYFGSSSVYFLTPTVVVTTKRWQLDGEWLLAPFGPRVWLLYIFALTLQVMVVQLLRCISKIFKLTWLDILGLALGNSRNIQYQFQSTRYFVMLIAFAFMVINGSFQGKLYAAFHLKSNRGLNTVSELIAKNYTFLKKKFILQELLDALQVPRAQIKELNYTDDFESYEQMLEFNYPVAMLTNYWQHQAFIRSRRLYDDFNTLPGIVVLNQVCAYTRPQSYLLEPFNRIVDNLNSAGILKKWMMDFLGIFDSQEELKNNDGNMNITPIALSLEKIRLVFIGLLVMHLIGVLVLVGEIIFKTILKK

>MdIR127

MALNSTIALLMLSSGSSMSNTMSNETQAVPQLVNSSFLIDIVSSIHDIYKFHNFVFFISERLTIDTDTAADFFQDFWDTFPTVPVLIMIDNAQVMDGYLSTPSLCLVLTTERDDPVMDVAADSMRGIRYFKTLFILFPIEESDDFYQTFEDYNRFYETIRMLYDWVWMKQFINTALITVKNNVFILDPYPTPTLVNITETWQPESFFINYGSDLKGYVINTPIRYDLPRVFYMKRPRIGARTKHQVTGVSGKIFTAFISTINATFNESWTDGLESEPVDINNIIKMVEEKHLEISMHTYTGLIGDGRATSYPIGINDWCIMVPYRNRSPEHMYLQNGFQQYTWLLICFSILYITIGIWLCSPSQSRDLSLSFLQAICSTILIVPLRVLMAPTLRMHFIFILLFLMGFFITNLYVSKMASFLTTTTEQPQINSVQDVIDAGLKIMIMDYEYDILVSNNFPEPFMDLLVKANKQVMDDHRDRFNTSYGYSIQSDRWNFLNIQQRYLKKPLFRLSQICLGPYYHVFPLQSDSHLAAPLQSFIMFASQLGLIKCWKNEAFADALYLGYVRMMLVNESLPPLSTNFFRSLWYVWWLGLIVSGITFCLEIKRITWLRVRDWCVKSYRRVCEELE

>MdIR128

MILNTSIVGLLSILVKDPANISENSKGITPEIVNHTFIWNIVHDLHKRTPFNDLVFFISENLMTDPVRGEFFHNFWQNLPQIPLTIKINNSQKLNGYLSLPSMCLVFTSGEEDAIMETTAASMKGIRSIQTIFVLYSVSQTEDTFEVVSAIFSWAWKKQFMNTLLITLWNNIFIYDPYPRGRVVNITDNWSLENLFNFMERKDFKGYVIHTPVQRDFPRVFYMGRQRASRRNTTKLSGISGSLFRAFMKSINATLNYTSSPEEPKNIFQIIELVGNKSLEISVNSYTAMFKTISGLSYPVGINDWCLMVPYLNRTKANHFLSRSFHPSTWALIGFSCLYISLGIWLCSPPRQKDLSKSFLQAICSLMLIAPLKVLQLRLWRMRLLFVLLFVFGFLLTNIYLSKMASLLTAYSEPQQINSIEDIIGAQLPIMMMDYEYEVLLSYNFPQQFMDLIITVNKSQMDEHRDRLNTSYAYSSQTDRWKFLDMQQRFLKTPLFRLSQICIGPFFHVFPVQKDSHLDKPLKDFIILASDMGLLAHWKKVSFADALFLGYVQMIKIDEGLMPLNLHFFRSIWIIWSMGLVLSAVVFLVELNWALFCKIIDLIVDFLKKLRNKIYEV

>MdIR129

MALNSTIALLILTSSHVIPSSNGATNHSHPQMVNSSLLIEIVRSLHNLNDFNNFVFFISDRLTKDTHTAAEFFHDFWHVFPTIPITIKVDNTENMDGFLSIPSLCLILTTAPNDPIMRVASIGLKGLRYVRTLFILFPFAGEDSNEELYETICQIYHWVWRKQFINTALLTIRNGIFIHDPYPEPSIANLTHNWTAEQFFVTSNMDFKGYEIRTPIHHDLPRVFYMTKPRRFITKNHFVSGVSGKLFTSFVEFINATFNETTTNELGTQPVNLSDIIRKVGGKRLEISLHSYTDMLPKKSGTSYPIGINDWCIMVPYHNRTPDHRFIKSSFRTYAWYLVIFSIAYITLGIWFCTPTPQRDLSLSFIQAICSLLLIPPLRVLTLPCCRMRFIFIILFVLGFITTNWYVSKMASFLTASKEPPQINTIADVIAAKLPIMIMSYEYQVLKEYNFPQAFMDLIINATKPQMDMHRDRLNTTYGYSTQTDRWNFINIQQRYLRKPIFRLSDICIGPYYHIYPIQKDSHLARPLQTFILLASNVGLIDHWEKEAFADALHLGYVHMMTVDEILPPLSLSFFRSIWLLWAMGVLLSIAVFAFEFHGQATCRKLRQACKLHATKFCNKFNGT

>MdIR130

MDILRQNANTTWDTAFLMGYILPLVAYIKIPEVIWFISERLNGEHHENLENFMLTLHARTFVPQKVLTNTLSDRIMQTDSKRNYLGVVLTTSADDPILDVHNLVLKGRHGYLNFVVIVDRIDDFRIAEETLYVLERNNFELSLLYVGYRNGSSDLYGITSFPEIHIAKRTNFFSSFSSTMSKVSSGGWDAYGYKYKTPLRQDVPYVFSSHDGQGNVVQRGISFSILNVFLEYVNASMEVYEMPKDPLGGDVIDMRAALNLIRNGEVIILSHAYALFSTDDNLDISYPIMVVRWCIMVPQWNRESTFYYALKPFTNAIWYAVLGTFVILCIIDGLWIYVQSLGKRTVVKRNVLIWLIRDSVLENFCFIINIAASRTIRNPSIMRFLFYAAVWFHGFFLTANYTSLLGSILTVTIFRGQINTMEDLIRANISVMIIDYEYDFLMSGGLNLSQDFLRLIRQVDSATFAQHQLRLNKSYAYFVTDDVWKFLAMQQGHLKQGLFKLTDICFGSFYLGFPMEPDTPVKRSMEYFIRNMYSSGLLEYFENNAFDHALQAGLVQHFNTDKEYTSAHMEHVMIVFVVLFVVYIVSVLTFFMECGYAWLKGKRGK

>MdIR131

MELKILACVLCGCLSLATSWDFQYLYGFLDSYINRTSVSEIVWFISQALDGDQREEVDGFMKGLSLRNYLTQFVWTEWMDVRMVEIGCKRVSTAVVISTGLEDPIMKVHNDLLIERHFYIGLLLYTHKVGDVETIENLARDLSQRNFVNTFIFFDSMEGVANLFGFKQFPEFELKNYTDYDSWFAREFKQLALAALDTGGYKWYTPLEQDIPGVFSYLTANGERWIQGTSYTILKSFMDSINGRLVEYPKNKSANDVVNMQNVMQLVSSRRIHISAHAYALFQKDKLLEKSYPLLVVKWCIMVPLRNELSTNLYALQPFEWQVWLLVVLVLFLLCSMDFLLWRVKQKYLHFLDLWLNDVCFLLNISPTFPLPNPGCWQRFLYYFAIFFFGFFITSLYCSYLGSALTVSLFREQINTLEDIIRLQLPIMIIDYELEFLESQGFPLQPEFLQLLLVVNSSSFYAHQLSMNVSFGYFTTEDRWHFLQYSQKHLKQRHFKFSQICFGSYHLAYVMEKDSPIWRNLEYFIFRIHSSGLYQVYEQKALYHAVKSGQLRIIRAAGEYQAVVMDHLVVIFALMLGIYVLGGLCLILEIICHSKKRRMN

>MdIR132

MPRNRQVIQFTMNIRRLAVAVISLALFPNPALQWNEDFIFNFIMQFYPMLQSMGNIWFLSPRMTTEHTEHLDSFIKRIQDATGEPQYVWTNRSDVRMIRTAAKRNCMVVVFTTDAEDPIMYTHNRVMTGRHFGLSLIIYAPKVSSIKDIERLCYVLYKGNFVNGMVYFQQTNGRNELFGHEQFPEFVMENRTDFMAYVRRQYKKALAASQDVGGYKFYTPLRQDLPHVIKYKDKGGRAQMQGTTFRILDDFVESLNGTIVEYQMPPDNYGGDVVNMKAVLELVRSRKIDLAAHAYALYHTDDDLDKSYPIMVVKWCLMVPLQNSISTFLYVLQPFEWKVWLVIIVVLFALQIMDLFKITLETIILRAQRKEYFSRVIEAWLDDYCVVLGITTPKPIQVPGLKRFLFYITLFYFSFFLSANYTSYLGSFLTVSLFRAQINTMEDLIQAQLPVMIIDYELEFLLSEGFQMPEEFGKLIRPVDSHTFVTHRIQFNKSFAYFVTDDTWHFLDEAQKHLKQKVFKFSDICFGSYHLAYPIQMDSPVWRDLEYFLFRTHSNGLLQKYEHETFQYAVSAGYIQRLAEAHDTTAAGIEHLRLLFIIWALMCSAAWLCLFLEIAVYKCRKARISKGKKMKIWH

>MdIR133

MNVRKLIIAILGLSCFPSPSLPWNDTFIANFILKLYPVLKAQGNIWFLSQLMTTQHTEHLDRFIKRIQDGSGEAQYVWNNRSDVRIIRTASKRNSVAFVLTTGPDDPVMHVHSRVMTGRHFYFSVFIYVPKVHDFREIERLAHLLYLGSFANSMVYYQQPNGQNELAGTEQFPHFQMINLTDFTVYVRRQFQKVMSANQDVAGYKFYTPLRQDLPHVIKYHDKMGRLQLQGTAFRILEDFIDSLNGSIAEYEMPRDNYGYEVVNMKEVLDLVRSRKIDLAAHAYALYHTDDDLDKSYPIMVVKWCLMVPLQNSISTFLYILQPFGWKVWCILLLVFSGLLSLDFLRIFLDSLIFKELRMKFPSQLSDAWLEDFCHIICITAPKAIKVATLKRFLYYATLFFFSFFLSANYTSYLGSFLTVSLFRAQINTMEDLIQAQLPVMIIDYELEFLLSEGFHMPEEFGKLIRPVDSHTFVAHQIQFNKSFAYFVTDDTWHFLDEAQKHLKQKVFKFSDICFGSYHLAYPIQMDSAVWRDLEYFLFRTHSNGLLQKYEHETFQYAVAAGFIKRLAETQEHSSAGLEHLRLLFIIWGLLCGMGLFCLVIEICIHKYRHYKILKF

>MdIR134

MSKIKILSFLLFTLANCWNIDYVGKRLALPIAMGTQETLFCACIECQDEKLALLMKWIQLATMHPQLVISQPSDYVLRNHDVKRNLLTIMILRDLDDPIVEIHRNLLRGRHFYVNIWILYEPQWNFTLIENILEYLYVKKFVNSDLYYVNASNGGDEVFGFATFPEFTVENKTHLVGNIKMFYHRIIEKTDLKGYRFETPLLMDAPKVIRYYNNNGELRIQGVTYNIMEMCLEYLNGTLIESQMEYSSDGVVNMKNVLEGVRQHKVELAAHGYALFHNDDEVQKSYPLLVVNWCLMVPITNKVFTMFYPLSPFQATVWLNFLVAFVLINIVSHFFLKFHDLDNHNFILINFCKFINAAPPITSHGSDMPWFEMVINGFIYVQGFFLAAHYTSMLGSFLAVTVIKSDINSIHDVIHQHLPVMIIDYELEFLEEEVLNLPPKFMDLLHAVNTSVFYEHQLNLNHSYAYFATYDTWHFLNLQQAHLRPPIFRYTDICFGDYHLAFPMVAESPIWRDIEHLMFRIHSSGLYYYHEKKSFEYALRAGVVSHLVEDPSFHTVGFTHLRMVVGFLVIGFLMALVSFLHELWQSRRERERAKRVDLEEQEADVANSVS

>MdIR135

MVKTLSSFNHFDNIIFYGTPNRVYDALLNVSTLLTNIGLEVHKTLGDIVAEFMFTNENSRPVMIFAIAPWNYVNSSESIPNLGKILTNRNLAVILINDIFNRELKEFVSTSLGASMETKLIFLLIGHELTEVLPFIRQEKLLRFFRWCWSKNILNAILMYQEKHVVANVDDLKMEIYSYSPFPRPIKLIKLTNVHPFFFFFDRTLDVRGYEFKTPVFNDKPSVFKAKRLDDDDDDDNDEEVSGIAGHLYLEFVKSINGKFVELETPESHPMMLDYEYELLVARNIDIAIHPFSNLLPHGYYGSYPITNTNSCVLVPVIPEIFVGHYIPRIMNLNMWLQVLVLFTGFQVAYFLIDKFNGGKWYPWKSISLTLKGMLNMSLGEINVSETFGIFSRSRILLIHMLVLLSGMLYSLSFIAGLTSALSATIFGKKLETLEDLRRANISIMMLDYMYFMYNYMDIIPNSFESNVLIADVETVSHHLNSLNTSFAYAVYNEEWQVLQSLQKKLWKPKFRIASKLCIANVYLSFPIQFNSAFYHPLKNFILRIRETGLELKWTSDILKDIRETTSGVNLMNHQEEHPVPLTIDHLRVIWTGWFLGMLLATMVFLVELYLKRIKRIIRRRNKQSKENKSFTQMLK

>MdIR136

MEPVALLIILINSLKASAAYEIPLSSENNQKNIDFISNYTEFLYERHNFDTFLIFCDNCSHSSESAIGETNLPQYLMRDLQIPLIIWGMERRVRFKHQLGLNNLVFIYIRQLRDPLLRVASETLTDLHFTAMMVILKTETIPDSRAIKDFFENCWAFNIINVVLIIFHGKETIEDLKIFRYSPFPWLAFEEVEEVVFDERMNFILEYINDTRGYVFDTPIFMNPPSVFLTPYEQHHNNISYPYITGTAGRIFHEFTHYVNGTINVVLNCNVSFYSYHKETLLLAALKVIDIGVHPYSGLLPYANETSGSYPIGYTNACIIVPVIPEIHPSDYIYRSLQPTILGILFLELLSLFLVELLRLRTFDFGEGVIYAFGTLLFQAMEPQRFQQRKVLLRVLHLLVVTVNVLVESTFCASLTSLLSTTVYGDQIDTPEDLLRSGLQIMVNRYEKEIYFDSELLPAVLRPRLFQVNATFSSWNKNKLNTNYAYVATSMEWRKLNLQQQLLWKPKFRLASAGDMCTASYFLRFPMQWDSPFHSALIKFFFVIQESGLLKVWEDRSIYHAISLNLLYYMDNDKIPGQYFDWTYLQKPLLIYGLMMLGAIVCFLVELWLYQRRN

>MdIR137

MQTLRTPFNQEIPRAFSYQDRNGKKQFGGENLQLLTDFARLHGMQLELVPLLDYNIAKVQGDIEKGIYNLSVHRSTFYNPLANITFSYPLEKSKICIMVPAEAELPRYWYLVWPFDVYIWLLYILAVPYVALCLSCVRKPKRNFGANCLASWALLLFNSNSHLRLVNSSTHLQVVFILSTFLAFILYGYYICYLTSYNMRPVFQPYLSTVSALIEANMEILTPKHISTELDNNPHVDLSSVHALMVNASYRDVAKLLSSQTRSYAFIVTHDDWLFLEKIQRHLIQPVYQITDICSCDIFTSYPVRRDSSFAAALDFFILLVHEAGLWQQWQERAFQALTNSKQFQVLKDSYPVNPLDIFYYRIGWILFGFGHLVAAICFVGEILIFRWKK

>MdIR138

MHFRILTIFLVFSFSIAITAIRVYNLQKILDYKSHNPEKYKFQPMELGMLIGSIVEYWNMTSVYIIYNSRMSQSNLLLEVLNDLNARNSYLANIPRMTLQSENIGKPLYNIADIGPNALVLSLMHSVYDSVLEATARALRKRRMCFTMYLLHTFNYAEDHRYLFKKLWKYQLRRPLVIANGKDLLTMDPYPVLKVVNVTLEPMSKWFPLADGIRDFKGYTLNMPVQNDFPATYFYKDEKTQKYQADGLAAWMVKELMARLNVTLKVHTLTVNNSYIFDYWKIFELLRKGNIELSPQLMLSIFREDGFDFSYPYISTSRCIMIPQSRKNTIVFLPFLDWKLCLVLAIFLMVYEILFKLYPLYRSRVGGVYEWRQQRSFYIPWIILGIPVPLIKFHPSLGRLRFSVFLRLLIVYFLIAFSGNYVSQIFSSNLTSLLTENYVKETSIKLRDVLSAKVPIIMRYFDTDSFVRFHKIANVDLRYFVNSTKEDLHKHRSKLNAKFMYFLTSEEYDVIDEQQRYLSPKRFRFSNICHGPYPLQFQFQADSHFLDLFHLFILRVHEAGLYEYQKRYLFERAKRYAKLDYVYESDLEKSKITFTTLSAMVYVLLVGYTSSIFVFILELYGQKIMRLFERKGILKGRRN

>MdIR139

MFIKDAFEPREMVKFINTIAEFWNMTSVYIIYNSRISQQNFMSEVLDELHSKNTYLNRLPQMTVGSKDVEKPLYDIVNISRNALVLTLMHSVYDPVLEATAKALRKRKLCFTIYLLHTFTYDEDHRYLFDKLWKYQLRRPLVIANGRDLLTMDPYPILKIVNVTKDLMWKWFPIVDGIKDFKGYTVNMPVQTDLPATYFYLDKQTQKYKADGLIAWYIRELMTRLNVTLEVYPLNNNETYFLDFRKIFSLHRNGDIEINPNVMTVHAHVENIDFSYPYIATSRCIMVPRRKKITIDFLSFIDWKLCVFLAVVVTFYQILWKLYPRYQSVVKRDYNWLKVPPYYILWLMLGIPVPHLNSLPTLGRLRTFAFLRFLVVFFMIAFNGNYISQIFSTNLTSFLTANYLKGTSSQLKDIIAANIPIVLGKVDAQPFLSYNHVGKKSLEKFIYIPYADVQRYRNRLNSSLSYLIAREEYPIIDEQQRYLDSKRFILSHVCHGPYPRQFPLRADSHFLELFHFFILRIHEAGLYQHQKRNLFQRIKNHGQLDYIRESDEEKCKITFTTLTAMFYVLSVGYASSITAFIFELYGKRIMRYFRESKIWKLRWSLGICNRRENVFISQNGKS

>MdIR140

MYLGISLFLLVFFYSIPMPTIAVEKFDEILQYETQTTRRTTFDPRKVAELINSIAEFWNMTSMYIIYNSKMPQNNLLLEVLNDLQTKDNYLEKLPRMVLRGEDVEIPLYKIIDVNRNALILTLMNSAYDPVLEATAKALRKRKVCFTIYLIHSFSYPEDHRFLFEKLWKYQLRRPLVIANGKDLLNMDPYPLLKIVNVTREPMLKWFPTVDGINDFMGYTLNMPVQNEIPGTYFYWDEKTQKYKADGLAVWFIDELMTRLNITLVANPLKINNCYAFNYRGIFDKLRGGDIELSPHLMLTIGLEEDLDFSYPFKTDSRCIMRPRPKKFILNFVSLIDWKLVVFVTAFIVIYEILWKLYPLYCAVVKSNYNWMHLPPFYMLWLFLGTPIPNSKSLPAVGQLRPMTYLRLFIVSFVIAFNSNYISQIFSTNLTSFLTANYIKGSASHLSDIFSANVPIIMRSFDARSFARYHNVEKVDLKNFINLSYEDVLKYRNQLNTSYMHLLSTEKYQLIDEQQRYLNPKLFRLSKICHGPYPMQFQFRADSHLLDVFHLFTLRVNEGGLYEYQKGQLFYRIKSRGQLDYIRESNPQRPEIAFTTLTAMFYVGIVGYTSSIIVFVFELYGEGLMRVFKGKTVLKFIRN

>MdIR141

MNWKTFLILAIFYPFAELLPLRENLERILQSQTLLADQEVFEPREMAKLINGIGEYWNMSSLFIIYKWKLTNNRLAQALLGELNQKNGYFELLPRMTMRDVDVEKSLYEIADVDANALVLTLMHSAYDRVLKATARATRSHRSCFTIYLLHTFTYDDDHRYIFEQLWKYQLRRPLLIANGRDLLTMDPYPVLQIVNVTSQPMASWFPIVDGIADFKGYTLNMPVQNDFPSTVFYLDAATQKYVADGFAAGVVTELMARLNVSVNVYPLNVNKSFALNYYEISELLRKGEIELSPHLLSIVDFDPDEDYSYPFVSTSRCIMTAQPHRTVLIFVDFINWKLCLVLVIIIAIYELVWHLYPLVFPNRSNRQQGWQRYRPCYVICILLSIPVPVLPLPALKRIHPLKFVRMLMLYLVITSSGLYISQLFSCNLTSYLTANYLKGPPSRLKDVLAENIPIMMIPFDVESFSNFYKIKLIDSQQLVATSYENVYQHLSNLNRSFMYLVSKEEFVVFDQQQRYLYPKRFSLSSVCHGPYPLQFQLRADSHFRDLFHFFILRMREGGLYEHQKKTLFQRIKNHINVDYIREEDSVKSTDFNIALNTVSAMIFVLSIGYTCSIIAFVVEWNFDRIVQWLEGMKRVFA

>MdIR142

MIWKVLLIFTTVYLQTEPIPVEENFEQILKIQTPIAAREVFQPKKVAKLINNIASYWNMTSLFLIYSSKMSYNHLAQEVLRELYKNEDYFHELPRMRLRDVDVEKPLYDIADVDRNTLVLTLMHTAYDGVVKATANATRNRRSCFTIYLLHTITYPAGHRYLFETLWRYQLRRPLVIAGDNCLLTIDPYPTLRIINVTMAPMAEWFPIVEDIKDFQGYTVNMPIQTDIPSSYFYKDEKSGKFVADGLSAWIINELMARLNITLNVYPLNVNNSYFLNSLKIVELLRKGEIEISPHLLSIVKYEPDIDYSYPFMATSRCILMPQPRKHTIAFLRFMNWKLSASLLVFLIFYEIVWTFYPLYCSNIPKHIFHLQHYRPLYIICVLLGIPMPALALPSWKRLGILAFVRILLLYFLIAFAGHYSSQLFSSNLTSFLTSNHFKSPPPEFQEILEDSKPIMMRPFDAQSFTEYFKIDVIASEHFVLATYKEVYRHRSQFNASYMYLVTQLEYEVMDEQQRYLQSKRFILSNVCHGPYPLQFQLAADSQFLDLLHLFILRLQESGINKYERQSLVERAKKHGKLGYIRDVDAEKSLQLNVTLNTLSAIIFVLAVGYTTSIIVFIMELHFKRIICAFKK

>MdIR143

MNWHFVLIFLFLNTNNISTNDVDKFNKILNYKTNTQRGQAISQATKMANLIDQIAEFWNMTSVYIIYNSKIQDNNLLRDFLSKLHQKENSYLHGLPHLSLRERDIASPLYDIANMGHKDMVLTIMHSVYDTVLKATANATRHHRSCFTIYLLYGNSNPKDLHYLFGQLWKYQLRRPLVIGNGKDLLTMDPYPELKIVNVTLEPMATWFPIANGIKDFKGYIVHMPVQTDIPSTYFYRDEKTRKFKADGSAAWIINELMSRVNVTLKVYPLKFNHSYVVRPARHFELLRSGEIELSPQLLTVLRKENDVDFSYPFVSTSRCIMMPRPRKVTIGFYRFITWKLYAFLGVFMVLYEVLWKFYPRYCRKVNRGYSWLHYRPFYAIGVLFGIPIPQLPLPSFVHLRSFAFLRLLIVYFIIAFSGNCISQMFSCHFTSLLTASYVKGAANIRLEDIFSARIPIMTRGYDVELFAKLYKIDDFDRDKIRTTSYEDIQAHRSQLNSSFMYLVTKEEYVFLEQQQRYLHPKLFRLTNICHGPYTLQFPLRADSHFLELFYFFILRLRESGIYEHHKRSLFQRAKSHGQSDYLQEGDIKGTSELDITFNTLRAMMFVLSVGYSSSIVVFILELYGKRIVCWLRGFKF

>MdIR144

MSKLTLSFILIFTVHIQSESIYHVVEKLQEEFDIYTLLHFASNGTVDAFNSPNIPQVVIGNETATDLRGSQGQRVLSFIRLDEIGLGELNEIIKPSLLNLHLADILFYTNTTWSEANEWQWLFEWCWVEGFWRILLMNEVDQFLSMDCIPEMSIKSVTLNEYLAMRKHRVKNLQGYPVKVAVGHSPPRVSAFFDDEGILQLGGFYGTIVNMFIEQFNASMDYVLMPNMSTYSVLSCIDSILEQSSDICSDAILYGNGIETTRPLYVVSSHLVVPFDKPLENYNYFRKPFTIDVWFCILITFVSTVVLLMLIEYKEYGHLRLVNSIFTTFSSLIGGSFSVEHFSDKYHYGLETILIFSGFMLSNYYLAVLSSLLLTKIYEREIESIQDVLSHNLTIMTTEFQQYVLEVTKAPEQIRQQTVVFSEEEAVDNMRKLNTEYVYFGINAEIDFFLYQQKYLSRPRMKKLADEAVTTDIGEIPMRAYWPLQDLLMSHMENVFCSGIIMYLETETFEEGIRRGDISFIPNRDLYVEPLSLEYFVLCGLLLAGGYSLSVLCFVVEIIVYKYRGKK

>MdIR145

MMKYILLLILIRIEDIQTASIVQVIEKIRKEFDIYTLLLFVSHDATSEALDSPSLPQLVVVNETAKDLRRSQGQRVLSFIRLETTGLAELNEIIKPSLINLHLADILFYTNSTWSEETEWQWLFEWCWSEGFWRVLLMNDAAQLLSMDCIPEMSITSVTLEEYFAKRKHRLVNLQGYPVKVAVGHKPPRVSAFFDDEGNLQMGGYYGHIVNMFVEQFNATMDYIIMPNMSSYSVLSCIDSILEQTSDICGDAILFGNGIETTRPLHVVSSHLVVPFDKPLENYNYFRKPFTLDVWICIAISFVSSVVLLLIIEYKEYGRLRLVNSIFTTFSSFICSSFSVEHLSPKYHYGLETILIFSGFMLSNYYLAVLSSLLLTKIYEREIDSIQDVLDHNLTIVTTEFQQYVLEVTKASPQIRRQTVVFSEEEAVANMRKLNTEYVYFGLNAEIDFFLYQQKFLSRPRMKKLADEAVTTDIGEIPMRAYWPLQELLMSHMENIFSSGIVMYLETETFEDGIRQGDIAFIPNKDLSVAPLSLEYFVLCGLLLAGGYSLSFMCFITEIIVYKYCGRK

>MdIR146

MQCYSVAKSNMKPLLLYTLLLSLLFSDALAEPLPQVLEKFCKNFDIYTLLVFGGNGSFDYWDNSPNSISLPRVVVGHAVAKDLRESQGERVMSFVNLDTNSVDYLEEILKPSLLFLHLRDVLFYTNTTWVRGEEWLWLFEWLWDQGFWRVLLMNEADQYLGMECLPKMQMRVLTLEGYFAMRERRYLDLQGFTIKTAVGNNPPRVNAYFDEEGRLQVSGFYGNTLKIFAEIYNASLEYVVMPNMSHYSVLDCIQSVRDHEVDVCMDVILWGTGIETTRPFYIVISHLMVPYDTPLEKYEYFRMSFGREVWILIFFTFCCTVVLLIVVEYKEYRRLSLINNIFTTFQSFICASFSLQHFSQNYRYGLEAILIFSGFMISNYYLSILSSILLTKIYKREINSVADVVSHNLSILTTDFQQWILEVTKASPLIRQQTVVVSEEFAVRNQRLLNPDYIYFGLDEKLDFFLYQQKFLTRPRLKKLGDEAVTTDIGEIPMRSYWPFQDVLESYCDNLFSGGVHAYIDEETYQDGIRLKQIAFIPNEDLSVEPLSLEDFVLCGLILAAGYLLGLLCFVIEIFVFKKIGRK

>MdIR147CTE

MKLTLVYFLIFLKNSQAESPLEIISKFYQEFDIYTLLIFANNGTRKLLQDFQQPQLVIAGEDGGNGTFKDLRQTQGERVLSFVSLDDMEFSYLEEVFKPVLVNLHLANIIFYTNGNWSTEEEEGGGGEEAEEEQWLWLFERCWQQGFWHVLLASGGAEVENKYLSMDSIPQMKMKSVSLEEYFEMKRNRVVDLQGYPIKVAVGNNPPRVTAFFDEEENLQLGGFYGNTILMFTEANATLEYIIMPNMSQYSILSCIESIVTQTSDICSDAILFGTGVETTRPQIIVTSRLVVPFDRPLENYNYFRMPFTEEVWILIAITFVSTLILLMLVEYKEYGELRIINSLFTTFQGIICAGFSVEHFSLPYHYGVESILIFSGFMLSNYYLAILSALLLTKIYKHEIDSIADVISHNLTIVTTGFQQYVLEITDAPAEIRRQTVLFTEEEAVANMRALNPEYVYFGIDAEVDFFLYQQKFLLRPRMKKLGQEAVTTDIGEIPMRFYWPLHDHLMSFMENMFCSGLIMYRQLETFEEGIRRGDIAFIPNEDLSVEPLSLEYFVMCGLILAA

>MdIR148PSE

MALTFLATLTAVDIPLRNFSTLMDLKLRLDIETFVVFDYENRGNLVNVLQREEGRRFALPNIPLVIVSQNIVWLLKNNFSRNFLPLVAVQSRREKNAEILKILLTAMQRZHLRLVVFVAMESLEPLEKWRFLWQWCHKYGFFKSVLINFQAMEEVVIFQHYNNKEQEQVTALSSGGEFWNFYVENGKNTNGYPIRVTLGNNPPRSLLWWSHEEAENHSRPHLHISGYYATILEIFAQQYNASLEYLIINPHKEYYNELDCLDRIRENRTDVCADAMIMGQGYIVTQPEEISHSYLMVPYDTPMDRFYYFVKPFQPQVWLWGELTSCYVTLMLSLVNRLQRGHWNFPQNYLNSMMASANLPFHLPLILGWRRKFLEIFMFICGFVLANWYLSLLSSLLSSRLYDRYISCLEDLQKHNLSIILSEYEYLFLKTSQLSPLISQQLQIVDNEFLLENRRNLRPEYAYYSQYDKNLFYLRQQIYMEKPKMRELTEYPINPVYGGIPMRPNWPLEDKLNNLMGYMLESGVFIRILDDTFYDALRIGHLHYFPMDGNSVEPLSLDYFRMPGTLLLVGYSMALVSFLMZILIKKFKKYLLN

>MdIR149

MIVSICSFTVWLLLHQAHVAGSLGMSIEDVIYQLNEQRGIEINVFLNLQKTGNGSYAIFMDNILVKNRKPLPRLLYSNYTVIQNLKGIFSENCLTVAWINWENLSITLLAVDKLLKALYFTDLLIVYETKDPQQLNSQLMHIYEKCWQLGISSVMVWTNHQIYIYHPYPSITVKRLENFHEFTNRSYLENFQGYNIKIPTVDFPPRCFNYTNRQGQLIYAGYIYKLISVFFAHHNGTTEYFFADMWSKNFSMEWGLTKCKPVGCAFLPIMLDAHNLFVASWAPFLAKIVLLVPAAKEISESLYLLIPFDGIVWAIVLITGFIYFLLMFGVARRWKSNVDIGLILMEAFKTIIFVALATPKRRNIQHFMLCLLFLFTGLFITNFYTSSLWSLYTSKVYESEMKLLTDIGNTNLKLFIYSLDKGYFDVIANNLPPIIRQRLYTGDDDQFTSYRQNLNMSYIYPAIEDLADYLLLQQMYLRRPKAIKLAEPTYHRSFFISMQLRTPFLQQFNRYFSRLFESGIFNKFMLDAQWDGLTSGKYKLLKDDRSTNEALTMSYFQFAFIMLGCGLGMAVIVFVVEFLWGIKIMLNMSRGRKRNNVLP

>MdIR150

MNIIQALRTIVLFSILKSSETLASPEKGVSNRQLIKLINEIYYGIRAESLLVFKTPSSEGENIQTLLLKLQQPKILLNWKNSQELRGKFNSQILLLVFMEEGTLEWSEEQWLNFTRHLNRWTHRDIVFITLTENISNASHEFLMQCWNKGFCRIMLTSIQGENLFRVKFIPHLRLEPISPAIYIKERKVIPDMQGYPLKISAGNNPPRAFVYPNQNNEMIYSGIVPRLIKIFARHFNFTLQWMLVPNYQSSSLRDCMAFLLENKIDLCGDFMHFNDKLYAIAAPVFINYGYIQVPFSQTIPKYQYFLQPFENSLWYSICILLAVHTLVLSLIHRYKSNFWSLGKFFLLSLQSILFVSTYNLPPWRGHLKYFLYLLLTLTGFIISTLYVTFLSSILTTNVYEPQIDSVEELKQRKIPILTNDLDIEVLKYFDSLDKISGNYLNVDITTYGKHRSRLNPQYAYITFEDKCDFYLYQQKFLQRPRLRLMTKPSIALWANIPMHHNWPFLDLMHRYMLRIFETGLLTHIQELTKEEGIVLGHIRFLKTSNLDSLPLDIHYFEMPAILLGIGYCSALLCFIGEVVLYRFRRIKFCMKSGLKK

>MdIR151

MNFKNLLILEILAFSSLFVKNIAPNGFIEDLQKEQQTNDEIKYQLKRLFNKVDQESRFDSCLLLGREDNIRDTLVAEVLQEDMGKTLLVQTFTRTFCSSCLKLNQNFILFIFWRYGDEDTYSRLLSQFLEYKRSNRIILLAPVHLSALFADIVSSYMFEKCVQNNLLNVIILLGNFYETMAFFAFELYPQFTLVNRSFGHDATEVEVFPNKMSNLHGHRIRTYPDQMIPRSVVYRDQYGLPKMKGYVVSFLETFAKSINGTLWWPLNLQDDKPVFYQNIFDMAKADRFDIPTALVPALYGNSSKIMSRSYEERPWCIMVPVEKPHSYKEFIYRSLNPLFFLRFVLSMMVLSLILEFSTKIMYSKLNQDYKITLDKIILNTKIFRGIFGCSFKLQPNSTKSLKILYTILFIRGLLITVQFSAILQSFLTHPLIVPVKNLKDLQANNLKIVIFKNDLDFIKNSRILNYKRFLPELKVFEDYYKYRELHSSFNTSYAYPVHHSQWYIYRTQQSSLPKPLFRLSNMCLNNMGNLGFVLPQNSLYLQPLNKFIIRANELGLPEYWMRLSYIELKKIGKIKNIPNPILEIEHRTMGPEEFNFIFEIMLKCYIFAFILLIAEIVCSKIYCKIECGLKK

>MdIR152

MYRQYLLTTNKCNSVHRQPLTMELKNQTFLQILALLLLLIQMAASNKLTENLQKELQKSNEIKFQLKQFLYRVDRESRFDSCVLMGREDNIKDTLVAEVLQKDMGKTLLSYSFPHSFCNSCIKLNKNFLVFIFWRYGDEEKYGKYLSMVLEYKRQNRIILLAPESLSNLYAGIVARHMFAACPKFKMINVVILLGNYYETKLFYAFEMFPHVEMVKKSFSHDAETVQIFPNKFLNLHGHKIRTFPDQMIPRSVVYRDQKGLPRIKGYLVSFLETFAKSINGSLWWPMYIKDDKPVFYQNIFDMAKTDSFDIPAALVPALYANSSKIMSHSYEVRAWCIMVPVENPQSYEEFIYRSLTLTFYIRFLLFTMVLSLILELATKLMYLKRNQDYAITLDKIILNTKILRSIFGCSFKLQPNSTKSLKMLYTVFFIRGLLVTVQFSAILQSFLTHPLIVPLKSFKDLQANNIKIVIFKNDLDFIKNSRSLNYKYFLPGLEIFDDYYKYRELHSSFNTSYAYPVHHSQWYIYNSQQSSLPKPLFRLSGLCLNNMGTLGFVLPQNSVYMQALNKFIIRVNELGLPEYWLRLSYIELKKIGIIKNIPNPILKIEHRTMGPEEFNFIFKIMIECYICAFILLLIEILCSKIYCKIELGLQRADKK

>MdIR153

MGYHEKRKNVIFIIILTLQNVSTMQLYEILQHNVALAQAEQGLWLKLLRQIDQEENFEIALVVGEMKKDFLEILLELQLDKSVLINEDFAEDFNMDSMNSKFITIVVLPMTMEISEFTESLANKLDLRRNNPVIVILEQHRGNVEQNDIELLFRQFIFYKMLNVLVLLQDFAMTQMLYTFKVFPEFKLQIQKLENFENLLPSKMDNVYGKVLRTIPDQVMPRSVVYTDQCGKLQVTGYIAQFIRMFARYINSTLKFPDDMIPGNTLFYRDFVNWTQMELLDLPCSITPLMSGETVSRMSYTYEVLSWCLMIPEEEPLTYQDFLKGFLTLQMLVGIFVMDVIFTTLLTLSQQLMYYRKYHTFDVEISNILINPQVILAHLGSSFKLNAYPGLSLRIIYVALFVSGLLYTTAFSVQLNAFLTRPTVQSITSLEDMLKYRITILTAKNEYTTLMKLSGDHFIPYLSLFKVIESYTEFADMRTAFNRSYAYPVTSSVWHVYATRQKLFSQPMFRRTDACFKSLDLMAFVLPRNSIYKQKLDILIARVSDMGLISFWLKNNFYDLVKIGKFSFEDLSKSEAVTSYIKMEDFYYVVTSLIKAFSFSFIVFVMELSWFYGSVIVRIKFCKVRIKDEIE

>MdIR154PSE

MKLKIIYHSLLHLGDFPNVINRCIIGHQKKELENDINKYFVCETRLARTMVVVSNVTTVFGLLPIWILQEQLSAAIVDILRDIWKERYYNTIIYIRHRDKLAGDRYDVDNVAKVLNIPMIQLEGNMSFYLWPNSNRELVAVVEMSGEDGEDKKLLKILWKSLRMLHKTRLLLLFKEGDKENYLEEIIKFCCNHKAVNVVAIRDNILQLPQFYSPKIFPSFKLKVYGPNHSLFCPNHVRNMYGTPLRLSLKRHSTKSYILKEVNGTYFLGGHVGHFFDEFAKYHNAIIEFPTGYLNALSFDGFLDNDTLDISQQLSVNRYESDRAYSDCYTYLDWCIMVPTASLIQKYMFYGIIFDIRIMGLILATILLLAMVIAFTFWLEGKATHLLDTVFNIYIFNGMLGQPYQMEPHFSGVRSILYMLICLGGIMINTTYVTYLQSFNANPPTEKPAXDILHHRKKILMYEDEYQTMHEIFTQSEIYPKIIKVIPTFLDFYTLRDGYDTRYLYPVPAVQWSQYDEQQNFFTKRKFRLSDICFFRMLPHMIPMQANSIFEDAINEMIGITTQAGLTNRWMKLAFLEAIQRKRLSLTDTSHKDTFEPMQVEDTKWFMXIKLGIASISFIFELIWHGLSIKPLIIIK

>MdIR155

MVILINVTTLLGLLQISSNSYELNVKIVNMLSKVRRERNYRTIIYMSSVDGVPEEGYNIDEVARLMETPMIQLKGNTSFYLWSKFNRELLAVVPMNGDERKDKLLLESLWRNLRKLLKTRLLLIFKAATDEEYMEDIIRFCSNHKAINVMAIKEDVASLDLCYVPNLFPSFVLRSHNMSGSSNFRFYPNHVRNMNKSPLRLSLKKGSNKSYILKEVNGIYSLGGHVGHFFDEFARFHNATITFPTGYHDAFVFDAFLDNDTLDMSQQLALNNYKSDRVHSDCYSLTDWCIMVPTASPIPDYMFYAMIFDLKILTLILVTIFVLTFAIDLTFWFEGRTTNPWNILFNIYTFNGMLGQPYQMEANYSGWRSVLYVLTCFGDVMVNTTYVTYLQSFNASPPTERPINTLEDALANPKKILMYEDEFSKMNNEIINDYDLFVKIIEVVPTFLEFYTLRDSYDTRYLYPVPEVQWSLYEQQQEFFAKRKFRLSNICLVKMYGQMVPMQADSPFEEAVNEMIGIAHQAGLTNHWEQMAFMEAVQRKRINLTDSSSKVRFEPMELQDTKWFMLLYLILNSVAFLCFMCEIVFYKLRNKSLIIIKI

>MdIR156

MLLWTNVTTLLGLLEIWGNSIEFNLKIVDILGEIWKESYYHTIIYICHEDRLLLESYDMDEVAKHFGLPMIQLKGNTTFYVWPKMNRKILAVVPMNGDEWADKSLLDALWVSLRRLVKSRLLLLFRAEEDEEYVEEIMKYCCSHKAVNVVAIRDNIAQLPEYYSPRIFPSFEMRTQQLNGFSKLYPDQVRNMHKAPLRLNLKRDSNKSYILKEVNGTYFLGGHVGHFFDEFARFHNATITFPTGYLDAFWYDMFLDNETLDMSQQLVLNDYSSDRTHSNCYTLLEWCIMVPTASPIQDYMLYAMVFDIGILWLILVTMMLLSSALALTYWLEGKITNLWNIFFNVYIFNGMLGQPYQMEPNYLGWRSILYVLTCLGGIIINTTYASYLQSFNASPPTEKPINTLQDVLQRNKKILMYDDEFKKMTNELFADYDMYLKVVKVIPTFLEFYTLRDSFDTRYLYPVPEVQWAQYDEQQKFFAKRKFILSDICLLKMYGQMVAMQANSPFEDAVNEMIGISAQAGLTNHWKQLAFLEAVQRKRIKLTDTSKKTTVEPMKVEDIKWLMFLYLGLNCMALLCAIVEILWYRFFVSINIVNRVE

>MdIR157

MSNVTNLIGFLQLCSVQNDLSLALVDILKTAREQKYFHTIIYARHADMSAGEIEELYNVDDVAKGFGLPMIQLRGKAPLYLWPVYNRQLLALLPMSGNEEKDKHMLESLWQVLRRSVKTRLLLVFKQSAEDDFIGNILKFCMRNKAINVLAVKESLPSTGVLFTIQIYPQFKVTQIELSWPIKIILYKDQVKNLYGQPLRLNINKGSTKLYILNRVNNTYRLGGHVGHFMQEFAHTHNASIMFPNLGDENNTFITDVEMMLDNGTFDISTEPSFNLYNSDRVYSNIFDYMDWCVMVPVEKPIPAFMYYSQVVDDNVWLLLLGTVVILSLLITLTIWLKSPVGTRLRLNFFNIYIFSGILGQSFKMETNFKGVRSALYMLTCIAGIIMNTSYTTYLQTFNALSPRDKMITNLDDLHETGMRLLMYNDEYNMIKAYGQEHIFRSVVTLTSFEEFMTLRNNFDTRYVYPVPSAQWLLYQQQQKFFTKPRYRLSDICFVKMIGLMIPLQANSRFEDDINRMIGQVEESGLLSHWKFLTFLESVQLKRINLLDNSPITGFEPMKVEDTRLVMYSIIIMAFVSFCCFVFEFLWFRRRTFWHKIKRIRECK

>MdIR158

MSPYANASLIMGFLQLISMENEMSLSVVEILRSIREQSYYHSIIFIGHENTSSSTGAGLYNMEKVSQSINVPVFQFRGNTSFYLWPKFNRDLLAIVPLCGQEDQDMDLLQTLWKSLRRIVKTRLLLLYQNNEDEDYIRNIMEFCKDNKAVNTMAINGNLLKSQEFLSPQIFPQFDVRIKKITPGNETTFYPNHVKNLHGHSLRLGINNESTKAYVMRETSSNKFTLSGHVGSFFKVFADFHNASITLPHFRQKIEVTHVLLEAMVNNGTYDMSMELSINQYANDMVYSYTYDFLDWCIMVPMENPVPAYLFYVRIFDILSTVIVFCAVFVLTLLVALTFWLQDYTVYWFDTFFNVYIFNGILGHPFKMEINFTGVRSFLYLLTCVGGIIINSSFATYLQTYKARPPTEKPIMTLNDIRSSKLKIAFYKEEYKFMQANNLSRPYDDVAFLIDTYRDYYTLRDSYDTRFVYPVPSPQWSQYEQQQKFFAKPKFRLSDICIFKMIGVMIPMQPNSPFEDTVNQMIGIVNQAGLIQYWKSMEFLESLQRKRLTLFDGSTAISFEVMKFQDTKLLGYLFIIMISVSSLCFIAELYWPRRGRLARRLWKMRKLCSCFKKPVN

>MdIR159

MSMATNVSLLLTFMEMSTYKSDYGQSLAELTKAIKKQSNFHLLILGRHDWDLESDFLWTILMENLEMPIINIRGNSDVKNPIISNLYQFVIVALSGIEAMDKQVLKSVWQQMKRIFLSKFLLIAKRNESNDYIRIILKFSVVNKALNMMVIKESFVEFREIFVPQIYPNFEMKRMIIENISAFEFYPDPVKNLHGYQLNVGIKQPNYRSYISKTCETHVHLGGYLGLLFTEFARQHNATLRKPNRGERVQFFYISKDLHKLLENQTYEMLAELSLDIFQTNTDFSVTYNFLDWCLMLPMEQPLGSYNFYAIAFEKKIILAMLGFLFVFSMLLEITSCRRFSPRNIILNIYVFNGLLGQPFPMQPNPLTIQMFLYSLIFLQGLIFNTAFVTQLQTLKATPTTEKAIRTVADMEKANLKFGIVQDEVDILKSQGIFDEFQSVSQIMEPLEFFRRRDGFDSRYAYSVPFDRWVIYEEQQRYFQMPKFRISNICLVKTMGMAIPLQANSPYKNAIDTMIGRLSNGGIINYWKSMAFWEAVKRKQMPLSDTSQKFKFTAMKLEDMQLIIPLFAIMLAFILMCFLAELYWYYRGWKHFFYFKIKILTKSK

>MdIR160

MSSALNVTLLLTFVQLSTFESDFGKSLAELTKVIKKQNNFHSLILCRHEHDLDSNFLWKILMENLEIPTINFRGNTNTKSPIINHLYQFVIVALSGTEAKDEQLLKFLWQQMKRISVSKFLLIAKQQERNDYIRKILQFSVENKALNTMVVKESFVELREIFLPQIYPKFKMKRMVIENMAGFEFYPDPVKNMHQYQLQVAIVNASYRAYISKMFENHVHLSGYLGSIFTEFARKHNATIKKLNMTLGHEHYYLNKDIHNIIEDESYEMLAEISVDIFKLNIDFSTMYDFLDWCLMVPMEQPLNSYEYYIITFDKQILILILCSLISLSVLLGMTSNIKRVKSLTFPDLFFNIYVFNGLLGQSFKTEPTPATIRMFLYSLIFLQGTIFNTAFVTHLQTFKATPTLQKPILTLDDMRKANLKFALINAEENLIRTQHLLSGYETACKIMESEEFYHRRNAFDTRYAYAVPSDRWNMYKEQQRYFLQPKFRMSDICFVKMIRITIPLQLNSPYKNAIDAMIRRLTDGGIIKYWKSLAFWEAVKKKEMSLMDTSQKFSFIPMKLEDMQLLWLLYAYMLALIGICFMCEIFWYYKGSKLCRYLKYKILNTF

>MdIR161

MSTAMNVTFLLSLVQLSTYKIDFGKSLAELTRVIQKQNNFNSLILGRHEQDLERDYLWKLLMENLEIPIINFRGNTNTKSPIINHLYQFVIVALSGTEAKDEQLLKFLWQQMKRISVSKFLLIAKQQERNDYIRKILQFSVENKALNTMVVKESFVELREIFLPQIYPKFKMKRMVIENMAGFEFYPDPVKNMHQYQLQVAIVNASYRAYISKMFENHVHLSGYLGSIFTEFARKHNATIKKLNMTLGHEHYYLNKDIHNIIEDESYEMLAEISVDIFKLNIDFSTMYDFLDWCLMVPMEQPLEAYEYYILTFENKILVVMLILLFLLSVLLGITSSSRGMEFSISNLFVNINVFNGLLGQSFKMEPTPTTIRMFLYLLLFIQGTIFNTAFVTYLQTLKATPTLKNPILTLDDMREANLKFALIKEEEDLIKTQYLLSGYETVCQSMEADEFYHRRNGFDTQYAYTVPSDRWNVYKEQQRYFLKPKFRMTGICFAKMVGVTIPLQLNSPYKNAIDAMIGRLNEGGIIEYWKSLAFWEAVKKKEMSLIDTSQRFTFVPMKLEDMRLLWLLFGYMLSLMGLCFVFEIFWYYKGLRLCCYLKNKILNKF

>MdIR162

MRQTLLILLIGLNLSGCKILRLSDELKKNSTTIYGKLFVKLMKDKRYESLLLYGEETNWKFGCFNILDTLQTMEIPAIIISSKVNMKISEKFNNEVVAWICLHSLQNRTEFSDLASALDHMRYTRVIVQVMVRSSRNELNNFYEFSTKLQMLNVMVCFEDFPQTAVYFLYGLFSPSKLEEHVFNPVQDQVIFPQRIQNMQGYPLRTLVEHTMPQAIEYYDAEGKRRLAGQLGRFVTSLAEKWNATLTFPYYVPHNVAINYRKFLPLMRNYSLDVPATSSAVFWRDDFREFSYXFELSYACLLLPVEHPWEFRYILLHLMESFFMAIMFGLIAAFSVIFYCQRIVQTSASLSYNWPIYVINTDVIQGVFGFSVNYKSQKRFSLNLLYITLFVCGLTNSSLFNSKIYSYITHPSPSKPIRNYEDLENSALKVAIAQVEFDYFNYVSNISSNLSRSKFYIMENITDFYKLRDTFDNRYCYFTFAAKMFHYKSFQQVKGVKMFRLSKEMCPNPLMLLHIPLSPNSYFRQAINFAILEVWQSGLLYYWQSISNADVVKTGNKIRVNLTTSDSVSLMRTKDMNVIFLIYFVIMLIAFIVFALEIIVFQIYNKR

>MdIR163

MRFSVKVNIFPFISLLFSQGSANFLQPLLGENNTNRLIFSNLIQKIYKEEKLDSLVVLHPEGIPKSMAMEGLYDSELPKLLLSRRADFLYKDFYNSEILFIFYGMSWEQEWQNFVEAMAELLDFMRHSRILIIVENLKFFEIHGEDLKHHLERFKMTNVLVMVLSQEMQPFVMKKIQPYPEYHWIDWRPNSTPQFPPLWLDLYNKTLMSFVEQTSSRSFVYADAKGNFHMNGFVARLILLFAEHYNASLEMLYPLKVGNKTHYTVINQLVADNKLDLPMAMIPAIFEEEWRHVSDTYDINEIMLMVPLSEALTMPEIFGALLDGKFFACYLSLALIFSLIHGLVEFCREHLENSWDFLLHPRIWPGVLGQAFTMAPHPAMSLKLLYLLLGFYGLYMATQFSADINTYFTRPPHHPEINSYKDLLGSPKKILINSADAQEIHDWLDPYRKSMIFTNNTTLVHELRRRLNTSYCYYATTASYQLAWRQQKYSSRQLFHTPKSMAFFSMLPWGFRLQHNSPYKQALNHLIHQVHAAGLVDAWTDSLFWDMLRLKQVSIRDVNPPAERKVLSVCDLFWVWMIVVIGLSGSGVVFLGEVYWGKWRGKKLGN

>MdIR164

MFFLPGILLILLSCQEVLAEDLLSPKQENVHLTFYSHLLENVYKEESFDSILLVYKRDPYFPEEILKDIYRLNIPTLCLSKDQGKLVAKTNFNRQIVAVLLFSKSLDLGLLQIMANSLDYMRQNRIIVVAVDIPKGGEKEEGEDFRKLLLESCEKYFFTNVLVIFTNKDLDTHEAIHLNPFPNYHWTKQGDPLRPYFQDHWRNMHNKTLLTYMDRAPPKSLYYKDPQGNLKINGFVARFVMLFAERHNAHLEMAFPLSFEEPTHFSLIVESMVRPNLIDIPMVMDTSPFIDKWYNMTNTLHHDKGLVAVPCAQALSKQEVYGILLNEVFFGYVILCTIGLSLVQSLIDYLFNGQLHMSRLLFSELIFPGILGQTFSIGNFSQISAKIIYFMLFLGGLYLNTIFSVNVSTHFTHPPKHRQIETMTDLLNSPLKILLYDLEATVILDHGMAYRPVFITTSNFNHLQELRNNLNISYGYYFSSSAWRMISLKQHFFENKILCTYDNLTLFPNLRWAIPLPHNSPYKEGLNELIELVNAYGLMEAWNADTFSDMLELKEMTISDPYRDSYGPPKALTIGDMFWIWMICLMGLGAAVGVFVIEQIWYRKSQKKGKKK

>MdIR165

MYTMSRRVVIVCGLLFLFENSQSVKNFPILEKFETEKNIQNFEKLCHTTLAAIERERPFYSLLLYQASVGELENMEIFNGCPWIRSIPHLILREGNVVPFSDLYNSEILSIIFMPRRVNEKLMESVAQSLENMRQSRIVIATPALESGQEFREEILKLCEKYKMTNVLLSHLGAADNHDFILQPYPKYKWAQVSLETPGGIFYPPNWRNMHNKTLLTQPDDSLPNSLVFHDDHGQVQVSGFVANLVFFFAEYFNAHLQMYRPLEVDAPVHFTEITQMVKAELLDIPMTLDAGGRGNWWHITKFVLLNKVAIMVPLSSQLNVDEVFHLLLDRRFFAIIYTASLIFSTILSLIEWIFNKIPWNWNFLMSDEVYPGVLGQAFKERLNPIVGQRLIYFCIALIGLILSTEFSAKVNSYFTSAPYHRQLERLEDLVDSPIKILLHPADALIMGQWLKKWHHIAVIAQNSTDFQVNRHNFNTSYGYVVQTPLWDIYDHRQKYFRRNVFHIPPAMDLHELMVWGIPLPRNSPYREALNAIIHLVHERGLMTAWIAFTYKHMVQLKLVPLKDPNTEEPLHALGVEDLHWAWLLNVIGWFMSGGIFCIELLVERLKKMGKK

>MdIR166

MLENSVIMETRKIVVIAVLLGISLVGRSQMELEFQDKEYQGLYELILQDIFRESPFDSILFVGNERPASEVIQGIEVPKLIFSPGHQRTFLYKDFYNSEILVVILLRFRIEEEVLQRAAEILDYMRQCRILLLAEDVEDSQLLRDTLRTLCEDYKMTNVFLVIFSEVAPHPSFWSLQNFPKFSWQEWCPRKHAAYYPIQWYDFQNISLRTYVDQDSARTFVYEDAKGNIKMNGFVAKFIILFARTHNATLEMPLPLEVGKETHFTIINQMVADNKLDLPMAMIPGMYASEWRDVSDTYDLNQIILMVPLSHKLSMQEIFGLLLNGHFFCCFFVSSLVLSFCHGFIDHWRQEWQQFWDWIITERVWPGLLGQSFHVRRQPLASLKIIYLVIGFSGLYIATRFSANMNMYLNKPPYHPQIRNYHDLRESKMKILVDVADSRDSEDLREFIVYTSNTTYLHENRRKLNTSFCYYATTATYQVLLRQQRYSSHHVFHTPKSMAYFSMLPWGFRLQHNSPYKEALNEFIHNVHSAGLIHAWHNSLFWDMLKLKKVSIRYRPVDTDQRVLTASDLYWVWMIIPTGLGGSCVVFFLEVLWVRKKVFKRKISLPQ

>MdIR167

MRPHRLIVTAIGFAFFLSKSNVADVTNIRDQVNFYGNLLKDIHQERGYDTLVIVHEDVNVDLRLKEIYGFPHPKIFLSKHFEFFYKQDFNSEILVIIIMSGALDLELMGIAARSLNYIRQSRILIIARNVSNEEEFMVECLALLEEYPMTNVLLHFLKNSFEIPLDYQQLKPFPEFHWQKRNFNEKDLKYYPQHWRNLYGTNITTHTDQSLPSSVTYIDERGNLKLNGHVARLVLLFAEYFNGTLRMYRPLEINGFTHFTVVADMATKRLIDIAMCLHVISTPGHSTWTYASDVYEIGRGMIIVPCSQPLSIGDMFEILLNEYFFGMVIICTLLFSILHFVIEYYFDGEFSYTDLILNNRIIGGVLGLSFSGRNSPWRGLKLIYILLFFAGLNINAQFSARMNTLFTSPPMHKQIETLWDIRNSNLKINVLRGDLAIMGGIMLDIFRSLIITEDVAEYSRMRFNFNTTTGYYATLAQWKLFSLKQKYHSHKTYCTYENLTLHKFIPWNILLQPNSQYKDAFNYLIHRVHEAGLSDAWYASAFNDLLKLKRLSLADPNPEGGPSTMTVDDLRWAWLVIIIGLAVGGGIFLLECWYHHHHNRYSD

>MdIR168

MIVTALGSVLLLLFFVWSVVASSTTTHGQVDFYTNLLRNIHQERSYDTLVVLHEDGFNDLRLKAIYSFPHPKIFLSKHFEFFYKKVFNSEIFVIILMSAALDLELMEIAARSLNYIRQSRILLIARNISNEEEFMTDCLPLLEDYSMTNVLLQFLRNSPEIPLDYQQLKPFPEFHWQKRNFKEIDLTYYPQHWRNLYGANVTTYTDQSLPSSVTYYDGQGNLKLNGNVASLVVLFAEHFNGTLRMYKPIVERGFTHFTVVAGMAIKRLIDIAMCLHVTGIAEHSTWIYASDVYEIGSASIIVPCSSPLSIRDMFKILLNEYFFGMVVVCTVLFSVFHFVIDCYFDQHFSYMDLVFNNRIIGGVLGLSFTGRNSPWRCLKLIYILLFFAGLNINTQFSARMNTLFTSPPRYKQIETLEDIRNLNFKINVLKGDLAIMGDVMLPISRSVIITEDIAEYSSMRYNYNTSTGYYVASAQWKFLNLKQKYHSRKTYCTYENLTLHKFIPWNILLQPNSPYKEPFNYVLHRANQAGLPDAWYNGAFLDLLRLKRLSLTDPNPERGPSIMTANDLEWAWLVLVIGLSVGAVIFLLELWHHHRTRYSDNNK

>MdIR169CTE

MLFHILFFMATLWTLACSPMKIRETLEEIPQKEKDMPENVLQKLLRDIYLGREFLSLLVVREENQKSLEPIIQDLFELSWPITLLTKSQGDFLYRMHHNREVVAVLLLTQKMQEEVMKILADALNFLRETRIVVVAVDVWDQPEFRGELLTSCKVHNMTNVLLSFGYSAKNPGNSESTLFYALKPYPEYYWTSLSPGDVEQENLKYFPQHWLNFYNKTLLTYSDRASLRSLYYLDEEGQLKINGFVPRFVMLFAEHFNATLKSAFPLDMQNPKHYATIMKEMVETNLLDIPMTLDTNPHYDRWFNMTDAYHHDRGLLIVPCAQALSIREVYVIILNWTFLGSVILCTVIFSLLQSLIDYLFDGLLDLSRLLLSERIFPAVLGQDFTPPPKEPRKILKFVYLLLFIAGLFLNTLFSVNVSTLLTSPPKHRQIENPQDILESSLPILLHDAEAYAMRYRIEDYVRAVITTNNYSYLEGLRDNFNTSYSYYSSSTSWYKDLMRQQYLARKIFCTYDDLILFPYMPWGIPLQQNSPYREGLNYLLHWVHAFGFVEYWSDSTFWDLLKLKQVSIRDPYLQPGPLALTANDLFWTWMILIGGLVA

>MdIR170

MGACCLSMAYKFEYFSKWKTFSVVYLNCVEMRNFWKTILIWCLIPRIYGNAISEVILETQNNLNRDLLQQIYEERQFDSLLLAYNPDLLKVAPNVGILQEILSFEIPKLLITESMPNFVVKKKYNSEVMCVLLMQGSKDMYLLNIIAPILDYIRQSRILILTKRIENLEDFEKELLELCQHHSMTNVLLMVIQEEEEKEESVIFMLKPYPEYHWLAWNSTPFYPQHWRNFEKKYLLTFTDQTPPRALLFRDPSTREMRLSGFIPRMVILFARHFNAKLKMFEGLEVGRPVHYTVINDMLETEQLDIPMVLDTGPEEKWMNMTYPLDVAQGIFMVPCAQPRNIREVFNILLGWKFFGCIGICTVTLSLMHSLYDHLFFGNYTPFNLLLNERILPGVLGQSFVARKTHLQGLKMVYFLLFLAGLNVSTQFSARVQTLFTHPTYHQQIEDMEQLRQSPLKILLDAIEAQYILPHIEAVKSSLIITQNSTFFQENRQNFNTRYGYYSSSTLWQMYRRKQQYFTHKVFCTSDGLTVFRLLPWGFRLQFNSPYQEPLNYLIHQVHAAGLVQAWHSSTFSDMLRLKLISIRDPNPERAAKVLVVNDFYWTWMIVAIGSGLGLMVFLGELWWHRRGSVFFK

>MdIR171

MQNFYANIVSLLLAVKGFSASTELWNFLEEETPNFSQNHLRDLLSEIYKERSYNGLLVLQENVCPNGVFDMDVPKIINVGRHIFVFKEHFNSDIVAVFLMRGRINKDLMAQGARILNHMRQVRVVIFMEDSRREEELEVKEEVLKISGKYKMTNVLLSFEGATFYQLRPYPEYHWLERRFADNLPYFPQHWRNMSQKHILTYTDQTAPRTVITAVDTTMPQQGVPKMNGFVARLVLLFAELFNARLEMCCNFHWKNVTPYPVINQMVDREQLDIPMSLDPVLKGNYSYRSQVYDVGKAFLMVPCSEAFSLQEISKMLLKRNFFGYIFICGFLLTALHSLTDYLLDGQFDWRDLLINERILPGVLAQASVARKSPWLVIKITYVLLFLAGLNISAQFSARMNTLFTRPPHHRDIESLQDIEDSSMKILLESSEAKTIEKVLKPISKSLVLTNQTARYLQMRRDLNTSYGYAVNTAVWKMLRRKQNYFPHKVFCTSDKLTIFPFIHWTIRLQANSEYKEPLDYLILRVHELGLMKAWHGSTFVDMLRLKQITLANPNAKEEFFILREQDLMWIWVMEMVGLTAACAVFLLELMWPRVGDGRRLIGKCCKILLSKFCQVV

>MdIR172

MQQRQIEISLLTVLFIHVLAVSLEVENLEIFDLKTFLQQIYAEKEFETLLVISDRESLKDNRVWWEVVQEIPLPKVLVTYGAAYEFVRSFNSEIFVIFVFHGELKEQLMATGAEVLNFMRQTRILVLVEEGDRGFQLELLKLCELYKMTNVLLKGVAAKNETIQQLKPYPRYQWSQWRGPPYYPQHWRNLENKTVIYFTDTTMTLSFPYEDGQGGVRLNGYIARLVLLFGEVFHGHMQMYASHEVAARTSLTQVNQMAEDNLIDIPMTLSHYNDGKWLYKSAVYDFLEGLVMIPTAQALSTTEVYGVLLNRYFFGCVLICTLLFALFQSLVDYCLDHSFQAVDLVLNYRFFSGILGQSFTPKESPWRSLKLLYFLVFVAGLNISTQFSATMNTLITSPPNHAQIQSFEDLKHSSLKILAITSDIEAIEDAADAIRDSLLLTDSIAFYEENRNNFNTSMGYFLVSLSWKVLRRKQQYFSHNIFNVYPKMTLFRMPCALQLQKHSQYKEPLDHLINRVHEVGLPAYWYANTFGDMLRTKRVTISSPPVSAEARAFEVKDLFWAWIIVVGGEVLGSVVFFAELLFYRLHKKTSIDLK

>MdIR173

MFTLPSLIQFGSKIFFFIFISKINDIPYIRKGKLIVVPYGKYKYITYQNPEKKGLFELVIDKMSSTKELLLLLAMFVSGTWSEHLIDYLTTQQHSKDDEFMLTYENLLWDIWQEQPFEGLLIVQHNDIKEVELLKTLCGFPLTKIIITKDVVFEYKHKIGNSNILAVILVSGLMDEKIMEAMAQTLNYMRQVRILWLVEGVSDKEGFLDTILNKSRLYKMTNVILNFIESQPGQLYFLKPYPNYHWITSENKEDDLYYHQHWRNLENTTLVTYADQISPHSFLYEDNNEKKNIRINGLVARMVLLFAEHFNASLQMYQPLQVGNQIPHFSIINDMVDANLLDIPMALDSAYDDRWFNMSDVYELSAIMIMVPLSKQLELREIFSVLLDPYFFGCLVASSLLLSSVHCLIDFCVDGFWQYLNLLLNDKVLPGVLGQSCEMRSKHQWASHRIIYLLVGFVGLHISTQFSARMNSLFTSPPYHRQIRTFEDQRGSPVKLLVDTADAYKLSYYYDEKNIDAIFTNTSHMLEVRASLNTSYCYLARSSSWNVINQVQSHFANKLFYTPEEMYILSMTIWGFKLQFNSPFREPLNDLIHWVHAYGFRQAWYRSAFSDLLKLKWISLRNLNPMVELKVFTAHDLFWIWMLLVIGLSCGGVVFMLELWFGRKQRI

>MdIR174

MLSSALANNLTAYFPFHNDNEVVTHEELLWNIWQEQPYEGLLVIQHNRIEDLGLNLLLRIPQIKIILTKDSRLEYKHKIGNSNILTIIMVSGYLNMEIMEATAVTLNYMRQVRILWLVENVTDTEGFKDLVLQQSQYHKMTNVILHFVELRDVYHFIKPYPKYHWTSGSVKDNNGAYYPQHWWNLQNMTLLTDVDLISPRALIYEDQNKNIKLNGLVVRMVLLFAEYFNATLKMYHPLEVGKKLTHFSVILEMVDENLLDIPMAVDGSYDDRWFNMTDPYEIDQIMIMVPLSPQYTLHEIFGVLMDPLFFACLMGFSLLLSLAHVVIDYYADGVWRLMDILMSEKVFPGLMDQAFLIRTSEWWSHRIIFFLIGFTGLNLYAQFSGRMNSLFTSPPYHPQVRTFAELKRSQIKLLVDIADARKLGYFYSERNINAIYTNTSNFIEVRASLDNTYCYFVRTDSWSIFDEIQKYFSKKIFHVPDDLVLFSLAMWGFKLQFNSPFKEPLNYLIHQVRSYGLREAWRRSVVSDLLKLKEISLWEPNPTVERMYLTVDDLFWVWVMVAVGCSGGAVVFFVELCISRKRWRIFTCS

>MdIR175PSE

MSTIFIDIQLLWDIWQEKPFEGLLIVQHQRPEDLRLQFLYRIPLTKIILTXYKHLIGNCNILTVVITPHAVDLKIMETTTVTLNYMRDVRILWLVEKTTSIEDFKDIVLQQCQLYKMTNVILHFVELKEQYYFLKPYPKYHWHIANVKENDHIYYPWHWKNLQNITVITFVDLVPPRALAYEDVKKNIKLNGFVARMVLLFAEYFNATLKMYEPLKIDKDLPHFGIINDMVDANILDIPMSLESSFDDRWLNMSYPYEIDQIMTMVPLSSPYTLHEIFGILLDSYFFVSLLTSYFLLSLALCIVDFYVDGFWLHLNWLFNEQVFPGVLRQSFQVRPTQLLSHRIVYTLVGFIGLNIGTQFSARINSLFTTPPYHPQIRTFDDLRQSNIILLVDFGDVEKLKFYFYEKKLTAILTNTSHFLEVRGSFNSSYCYFVRTASWNIFNQVQNYFSYKIFLYWIWMLIVIGWSCSMFVFFMELYLVWKR

>MdIR176

MAVGRRRFEPLYGFPAMKIVEIQQETSFPLRREFGVDILVLLLLTSGEELGSFERLSKTLNDMRQVRILICGLLRVGESENLFKENVLKLCQFYKMTNVLMKLFLLENEDGGEISPDYYELKPYPTYGWFEKNLLLNHGIFYPQHWRNLQNITLITCTSQITPGNLVFEDENGGIHINGLTARLILLFAEKFNATLKMLKPLKVGEIIHYGLINEWTFQNKLDIGMVLASGDGETYMRYLSDSYDMTHVMLMLPCSGKLNLLEVFGILLNLTFFACLFICTYLLSLVQYLVDYIFDGVQNHLELLLNLKIFPSILSQSYDLKPSKWKGLNIIYFLAFVAGLNISVQFSAEMNTFFTSPPQKHQISTFEELSRQSALKILLDARDVAEYREWLPTIGKAYISSANSTFVVEKRNSLDTTYGYLVTHNRWHFVEQQQNYFSHKLFCTNEQLFLPKPPFSIALQENSPYREALNYLIHRVHERGLYIPWYSSTFVDMVKLKMIALNDLNPEERLKVLTAKDLFWLWMILFIGLGLSVVVFICELYWENRNRMEIISK

>MdIR177

MLVIMDSWAKVVVILLAIKATVNGIPFEKFHLDKDSNGHFYEEILKEIENEKVIESLLVLQENIAMDVSLRIFHESPIPKVIISKNQNFQFMEKFNTQLLTIFLMAEKFNPELLAMGARILDYQRQTRIFIVARKIAKYQGEEAFKNELLKDLENYKMTSVLLCFEEDKRLYVLKAYPKYHWLEKNLEDKYYPPYWQNLQNKTLITLNGQDPPTGLVYLDNEGRLQMNGYMARLIMLFAERFNASLQLHKSFKFGKSTAFRDINDFSFRGELDIPMSLAYQADPTYPQNLKTMYYEIIKPLMMVPCPTQLTYRELFGLLLNEKFLGLLIACYLQLSLIHCCIDYFFDHFWNPIDFVVNDKIFPGLLGQSFVTRTSSCRILRIIYLLLSLIGIYITVLFGANIKTLFTQPPYHKYIETNEDLKESPTKIFSDPTYAVDLLAFYNQDSVVVAPNDAEYLKQKSKFNNSYAYIISSTEWEALFSRRQQFYTKKQFCIAYKINLQDFLLYSMVLPKNSPYREPLNEHIMRVQELGFMEAWQSSTFVDMLRLGNISLFGGYDIVGDKKILSADDLFWIWMIIVVGAVMGILAFGCELYLGKRSKSRCIRKNKKNRK

>MdIR178PSE

MQNMTTELTLLVIFISRLPNPLIMKFPANHNLVCMEFYLKAVAVLLAFGKSTWGFSAENFQLEQSPGDNFYGEILKDIAKERAVESLLILQQNSTMPGGLRIFHDVPIPKVIFSKPQNFFFVENMEVVAILLMADTFDADLLAVGAKILDYRHQARILMVAMDIRENWEVESFKNDLLKDLKNYRMTNGLFHFZRKTEGEGTPTSRLYALRPYPDYHWTKKCEEEKYYPPHWKNMRNTTLITLNGQDPPTALAYLDREGNLKMNGYMARLIMLFAERFNASLQQHKSFQIGKTTPYRNINELSVKGELDIPMSMAHNTNSSHPQILKTVYYEIITPLVMVPCPTRLRKQEQIALLLNGYFFAWVLVSSLLLSIFHSLVDYIFNNFWDLKNILINDHILPGILGQSFLISRTPWRSLKIIYILVAFIGLNITVNFAAKFSTTFTQPPYHRAIETIQDLQESPIKLLTDYAYGPSLSRTYGKDKVHILADSSQYLKHKSHFNNSYAYVMTSTEWQALFSRRQQFYSXKQFCLSSQVPFPNLYIYNIILAKNSQYREPLDELIMRVHELGFMEAWQASTFMDMLKLKNISLFRGYTIAEEGKILRVQDLFWMWMILVVGLAIGVVAFLAELGVNKIKRNKN

>MdIR179

MALELFLNFTLTSWHLRELNYIISGAHYNHDIQTLVFFGSTFEVERYIRAAEIWTTPKIVITEHTGEIHLKNDAGVNNNIFFVAIGNLRRRSFWNHMNNALSEMQSRVRGVFITNPPNGKRAQLNVGEHFEWCWQRGLINTLILVDNELPREERFEIFNYNPFLKNSILDKSNATSFNLFPDKFRNLHGYQIKATAQYDPPRVFERRECKGKKRQPLSGYVANIFRAFLKEYNASLYLPYYYPNKTLDIVEILQQINQGDVELSINPYVPHKGVQLSYPIRMLRRCIVVPAAKELEKYKYFIMPFDVDVWLCFLGSWVWLSLARLLNWTSTHRRCKCNRLDVGRTFLEVFRLLAFLPVPGNCCSRSVRWYHLLWFMLIVPLAFILSNLYLASLTSFFSGLTFRPQIRTLDELVKRNLAVETIDYDIPSILDNRGLPKGFVDLLKPRSPSELIADILDLRSTLTFSALIDRIQFVLNQEKHLLKPTKHIVEECINTVPFGFVMPPHSQFELPLNRFLLRCAAAGLIEKWARDSIEDAYCSGMLTLKKTEFQVARPLLLEHFQFGWYVLGGGYTLSLLAFVLENLKRILRRFRIFIIYY

**References**

1. Larkin MA, Blackshields G, Brown NP, Chenna R, McGettigan PA, McWilliam H, Valentin F, Wallace IM, Wilm A, Lopez R, et al: **Clustal W and Clustal X version 2.0.** *Bioinformatics* 2007, **23:**2947-2948.

2. Schmidt HA, Strimmer K, Vingron M, von Haeseler A: **TREE-PUZZLE: maximum likelihood phylogenetic analysis using quartets and parallel computing.** *Bioinformatics* 2002, **18:**502-504.

3. Swofford DL: **PAUP*. Phylogenetic Analysis Using Parsimony (*and Other Methods).** 4 edition. Sunderland, MA: Sinauer Associates; 2002.

4. Pelosi P, Zhou JJ, Ban LP, Calvello M: **Soluble proteins in insect chemical communication.** *Cell Mol Life Sci* 2006, **63:**1658-1676.

5. Forêt S, Maleszka R: **Function and evolution of a gene family encoding odorant binding-like proteins in a social insect, the honey bee (*Apis mellifera*).** *Genome Res* 2006, **16:**1404-1413.

6. Gomez-Diaz C, Reina JH, Cambillau C, Benton R: **Ligands for pheromone-sensing neurons are not conformationally activated odorant binding proteins.** *PLoS Biol* 2013, **11:**e1001546.

7. Vieira FG, Rozas J: **Comparative genomics of the odorant-binding and chemosensory protein gene families across the Arthropoda: origin and evolutionary history of the chemosensory system.** *Genome Biol Evol* 2011, **3:**476-490.

8. Hekmat-Scafe DS, Scafe CR, McKinney AJ, Tanouye MA: **Genome-wide analysis of the odorant-binding protein gene family in *Drosophila melanogaster*.** *Genome Res* 2002, **12:**1357-1369.

9. Vogt RG, Rogers ME, Franco MD, Sun M: **A comparative study of odorant binding protein genes: differential expression of the PBP1-GOBP2 gene cluster in *Manduca sexta* (Lepidoptera) and the organization of OBP genes in *Drosophila melanogaster* (Diptera).** *J Exp Biol* 2002, **205:**719-744.

10. Graham LA, Davies PL: **The odorant-binding proteins of *Drosophila melanogaster*: annotation and characterization of a divergent gene family.** *Gene* 2002, **292:**43-55.

11. Zhu BB, Jiang Y, Niu CY, Zhang CY, Lei CL: **Construction of a cDNA library of the antenna of housefly, *M. domestica* domestica.** *Zoological Research* 2005, **26:**203-208.

12. Gotzek D, Robertson HM, Wurm Y, Shoemaker D: **Odorant binding proteins of the red imported fire ant, *Solenopsis invicta*: an example of the problems facing the analysis of widely divergent proteins.** *PLoS One* 2011, **6:**e16289.

13. Kim MS, Repp A, Smith DP: **LUSH odorant-binding protein mediates chemosensory responses to alcohols in *Drosophila melanogaster*.** *Genetics* 1998, **150:**711-721.

14. Jeong YT, Shim J, Oh SR, Yoon HI, Kim CH, Moon SJ, Montell C: **An odorant-binding protein required for suppression of sweet taste by bitter chemicals.** *Neuron* 2013, **79:**725-737.

15. Wu DD, Irwin DM, Zhang YP: **Correlated evolution among six gene families in Drosophila revealed by parallel change of gene numbers.** *Genome Biol Evol* 2011, **3:**396-400.

16. Su CY, Menuz K, Carlson JR: **Olfactory perception: receptors, cells, and circuits.** *Cell* 2009, **139:**45-59.

17. Touhara K, Vosshall LB: **Sensing odorants and pheromones with chemosensory receptors.** *Annu Rev Physiol* 2009, **71:**307-332.

18. Jones WD, Cayirlioglu P, Kadow IG, Vosshall LB: **Two chemosensory receptors together mediate carbon dioxide detection in *Drosophila*.** *Nature* 2007, **445:**86-90.

19. Kwon JY, Dahanukar A, Weiss LA, Carlson JR: **The molecular basis of CO2 reception in *Drosophila*.** *Proc Natl Acad Sci USA* 2007, **104:**3574-3578.

20. Lu T, Qiu YT, Wang G, Kwon JY, Rutzler M, Kwon HW, Pitts RJ, van Loon JJ, Takken W, Carlson JR, Zwiebel LJ: **Odor coding in the maxillary palp of the malaria vector mosquito *Anopheles gambiae*.** *Curr Biol* 2007, **17:**1533-1544.

21. Benton R, Vannice KS, Gomez-Diaz C, Vosshall LB: **Variant ionotropic glutamate receptors as chemosensory receptors in *Drosophila*** *Cell* 2009, **136:**149-162.

22. Croset V, Rytz R, Cummins SF, Budd A, Brawand D, Kaessmann H, Gibson TJ, Benton R: **Ancient protostome origin of chemosensory ionotropic glutamate receptors and the evolution of insect taste and olfaction** *PLoS Genet* 2010, **6:**e1001064.

23. Abuin L, Bargeton B, Ulbrich MH, Isacoff EY, Kellenberger S, Benton R: **Functional architecture of olfactory ionotropic glutamate receptors.** *Neuron* 2011, **69:**44-60.

24. Robertson HM, Warr CG, Carlson JR: **Molecular evolution of the insect chemoreceptor gene superfamily in *Drosophila melanogaster*.** *Proc Nat Acad Sci* 2003, **100:**14537-14542.

25. Robertson HM: **The insect chemoreceptor superfamily in *Drosophila pseudoobscura*: molecular evolution of ecologically-relevant genes over 25 million years.** *J Insect Sci* 2009, **9:**e18.

26. Vosshall LB, Hansson BS: **A unified nomenclature system for the insect olfactory coreceptor.** *Chem Senses* 2011, **36:**497-498.

27. Kurtovic A, Widmer A, Dickson BJ: **A single class of olfactory neurons mediates behavioural responses to a Drosophila sex pheromone.** *Nature* 2007, **446:**542-546.

28. Penalva-Arana DC, Lynch M, Robertson HM: **The chemoreceptor genes of the waterflea *Daphnia pulex*: many Grs but no Ors.** *BMC Evol Biol* 2009, **9:**e79.

29. Robertson HM, Kent LB: **Evolution of the gene lineage encoding the carbon dioxide heterodimeric receptor in insects.** *J Insect Sci* 2009, **9:**e19.

30. Erdelyan CN, Mahood TH, Bader TS, Whyard S: **Functional validation of the carbon dioxide receptor genes in *Aedes aegypti* mosquitoes using RNA interference.** *Insect Mol Biol* 2012, **21:**119-127.

31. Slone J, Daniels J, Amrein H: **Sugar receptors in *Drosophila*.** *Curr Biol* 2007, **17:**1809-1816.

32. Mishra D, Miyamoto T, Rezenom YH, Broussard A, Yavuz A, Slone J, Russell DH, Amrein H: **The molecular basis of sugar sensing in *Drosophila* larvae.** *Curr Biol* 2013, **23:**1466-1471.

33. Kent LB, Robertson HM: **Evolution of the sugar receptors in insects.** *BMC Evol Biol* 2009, **9:**e41.

34. Sato K, Tanaka K, Touhara K: **Sugar-regulated cation channel formed by an insect gustatory receptor.** *Proc Natl Acad Sci USA* 2011, **108:**11680-11685.

35. Miyamoto T, Slone J, Song X, Amrein H: **A fructose receptor functions as a nutrient sensor in the Drosophila brain.** *Cell* 2012, **151:**1113-1125.

36. Lee Y, Moon SJ, Montell C: **Multiple gustatory receptors required for the caffeine response in *Drosophila*.** *Proc Natl Acad Sci USA* 2009, **106:**4495-4500.

37. Moon SJ, Lee Y, Jiao Y, Montell C: **A *Drosophila* gustatory receptor essential for aversive taste and inhibiting male-to-male courtship.** *Curr Biol* 2009, **19:**1623-1627.

38. Miyamoto T, Amrein H: **Suppression of male courtship by a *Drosophila* pheromone receptor.** *Nat Neurosci* 2008, **11:**874-876.

39. Fan P, Manoli DS, Ahmed OM, Chen Y, Agarwal N, Kwong S, Cai AG, Neitz J, Renslo A, Baker BS, Shah NM: **Genetic and neural mechanisms that inhibit *Drosophila* from mating with other species.** *Cell* 2013, **154:**89-102.

40. Thorne N, Amrein H: **Atypical expression of *Drosophila* gustatory receptor genes in sensory and central neurons.** *J Comp Neurol* 2008, **506:**548-568.

41. Rytz R, Croset V, Benton R: **Ionotropic receptors (IRs): chemosensory ionotropic glutamate receptors in *Drosophila* and beyond.** *Insect Biochem Mol Biol* 2013, **43:**888-897.

42. Grosjean Y, Rytz R, Farine J-P, Abuin L, Cortot J, Jefferis GSXE, Benton R: **An olfactory receptor for food-derived odours promotes male courtship in *Drosophila*.** *Nature* 2011, **478:**236-240.
