# Supplementary figures and images for: Genome of the house fly, Musca domestica L., a global vector of diseases with adaptations to a septic environment
Source: Genome Biol. 2014 Oct 14;15:466. doi: 10.1186/s13059-014-0466-3 (PMC4195910; doi:10.1186/s13059-014-0466-3)

10% corrected distance

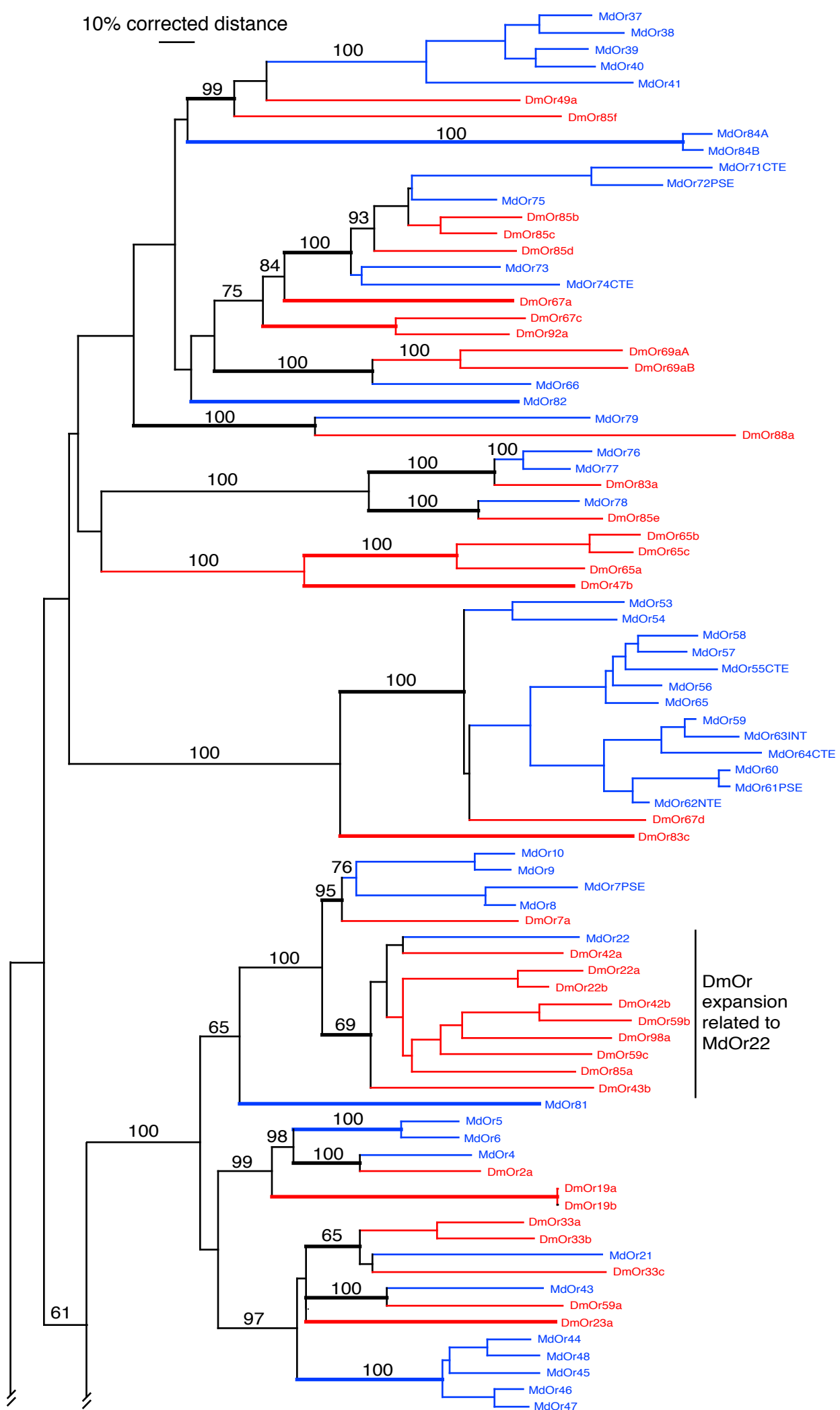

MdOr  
expansion  
related to  
DmOr67d

DmOr  
expansion  
related to  
MdOr22

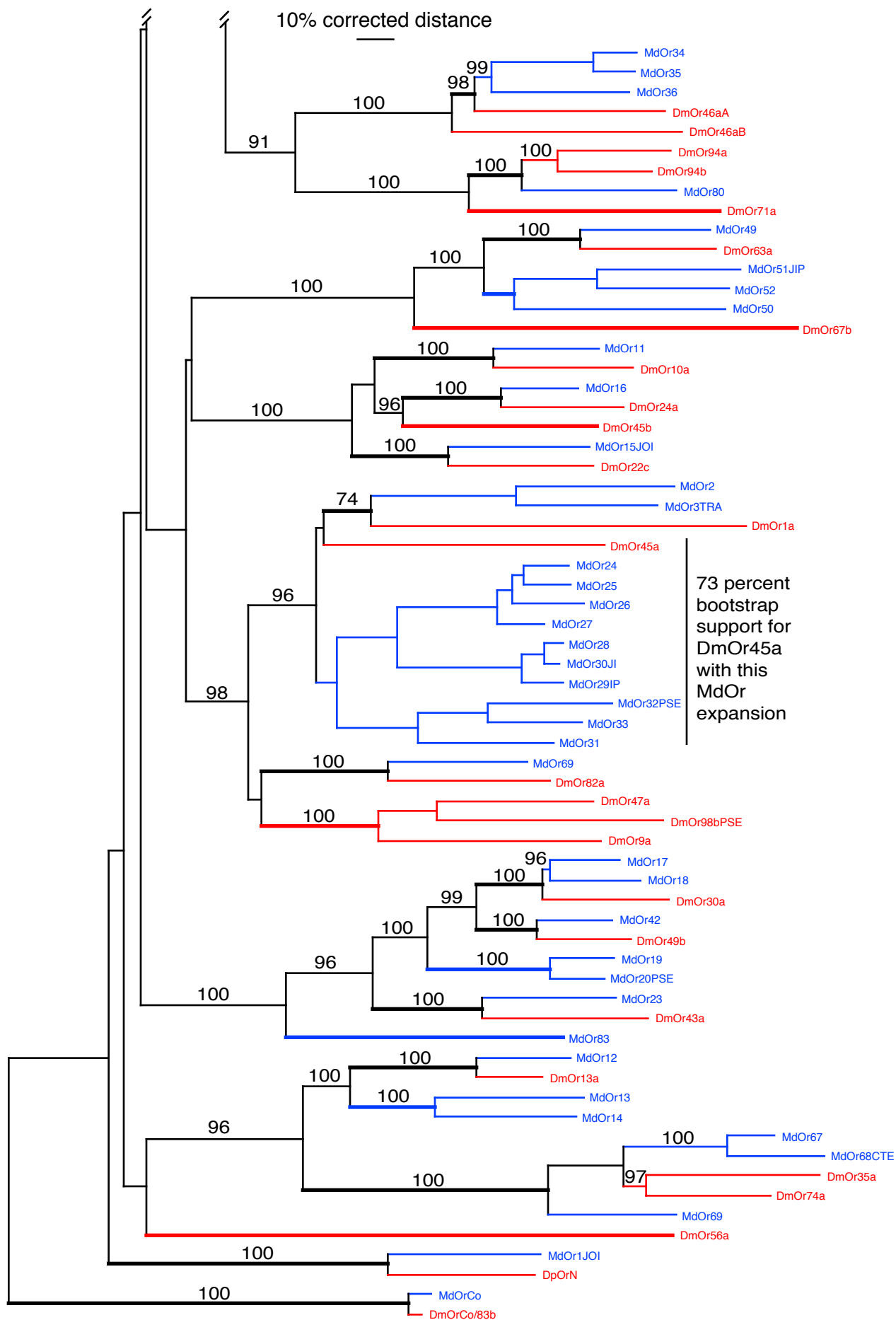

Supplement: Additional file 17: Figure S3. — Phylogenetic tree of the M. domestica and D. melanogaster ORs. This is a corrected distance tree with the OrCo orthologs as the out-group to root the tree. The OrCo orthologs were declared as the out-group to root the tree, based on the basal position of this gene in the OR family in analysis of the entire chemoreceptor superfamily in D. melanogaster [87]. Comments on major gene lineages are on the right. Suffixes after the gene/protein names include: FIX, sequence fixed with raw reads; INT, internal sequence missing; JOI, gene model joined across scaffolds; multiple suffixes are abbreviated to single letters. The M. domestica and D. melanogaster gene/protein names are highlighted in blue and red, respectively, as are the branches leading to them to emphasize gene lineages. Bootstrap support level in percentage of 10,000 replications of uncorrected distance analysis is shown above major branches. Inferred ancestral and orthologous lineages are highlighted in double thickness. Suffixes after the gene/protein names are: NTE, amino terminus missing; CTE, carboxyl terminus missing; PSE, pseudogene. [file 13059_2014_466_MOESM17_ESM.pdf]

50% corrected distance

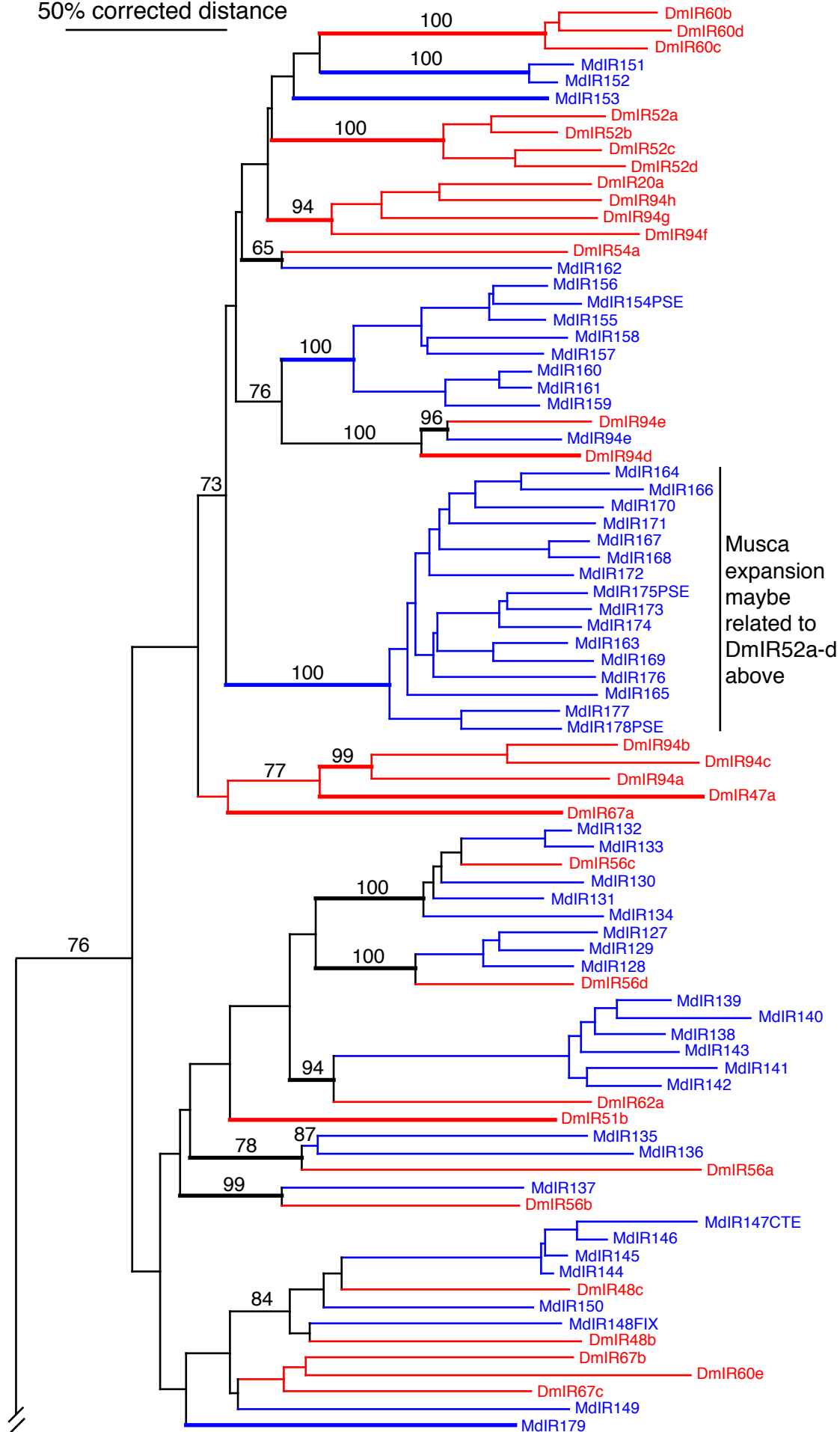

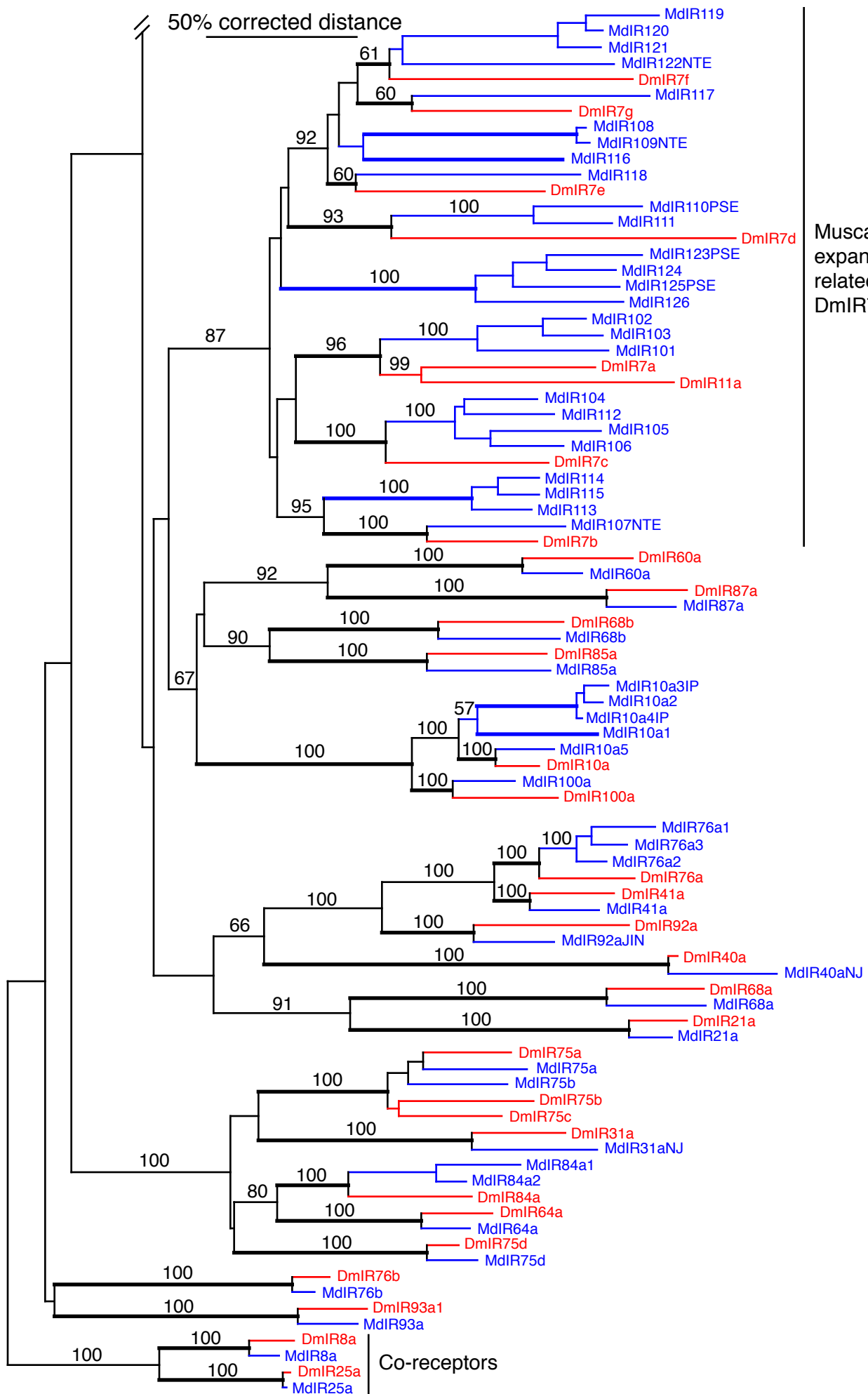

Supplement: Additional file 19: Figure S5. — Phylogenetic tree of the M. domestica and D. melanogaster IRs. This is a corrected distance tree rooted with IR8a/25a as the out-group, based on their highly conserved sequences and ancestral position in the family [134-136]. See Additional file 17 legend for other details. [file 13059_2014_466_MOESM19_ESM.pdf]

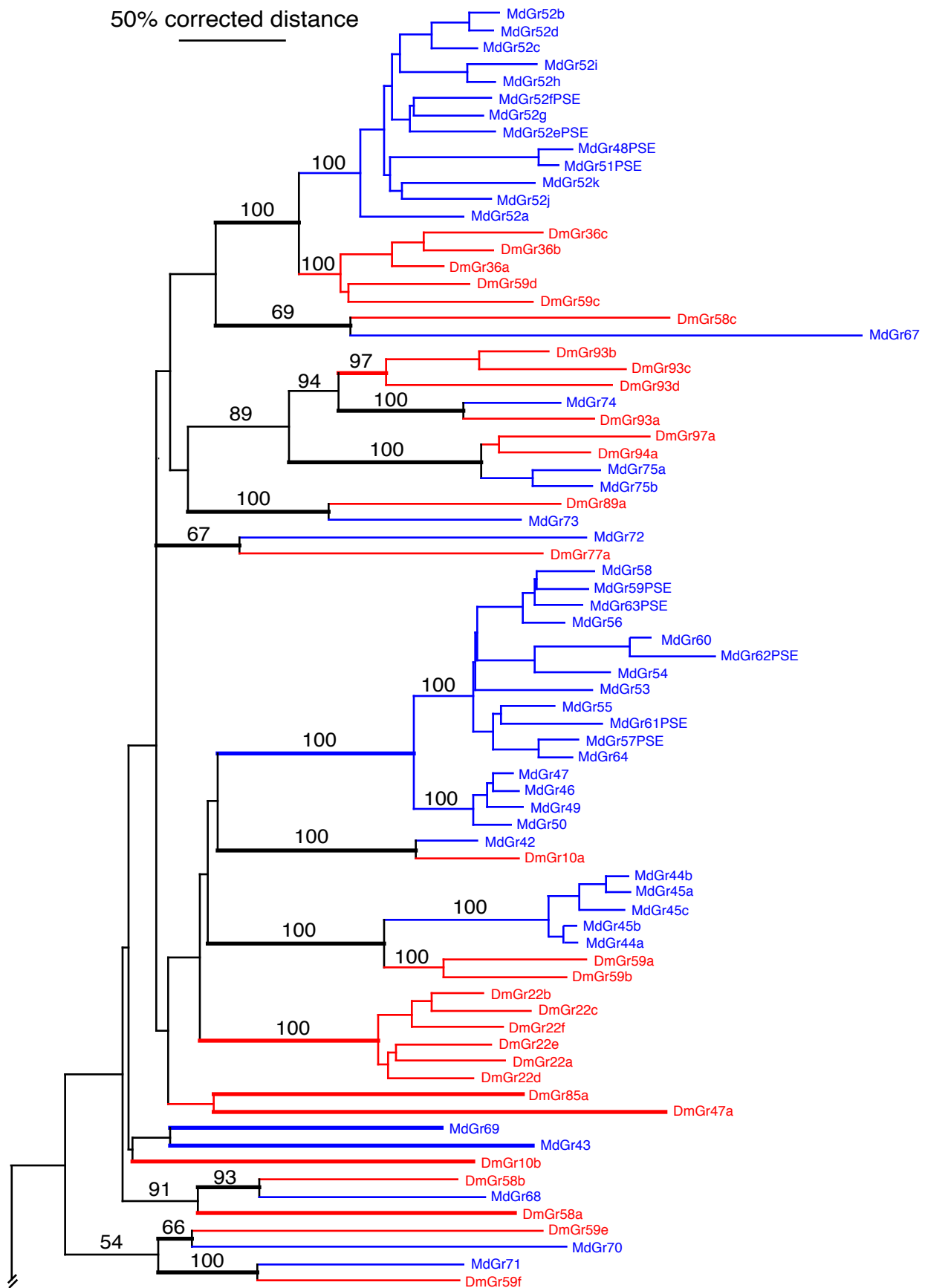

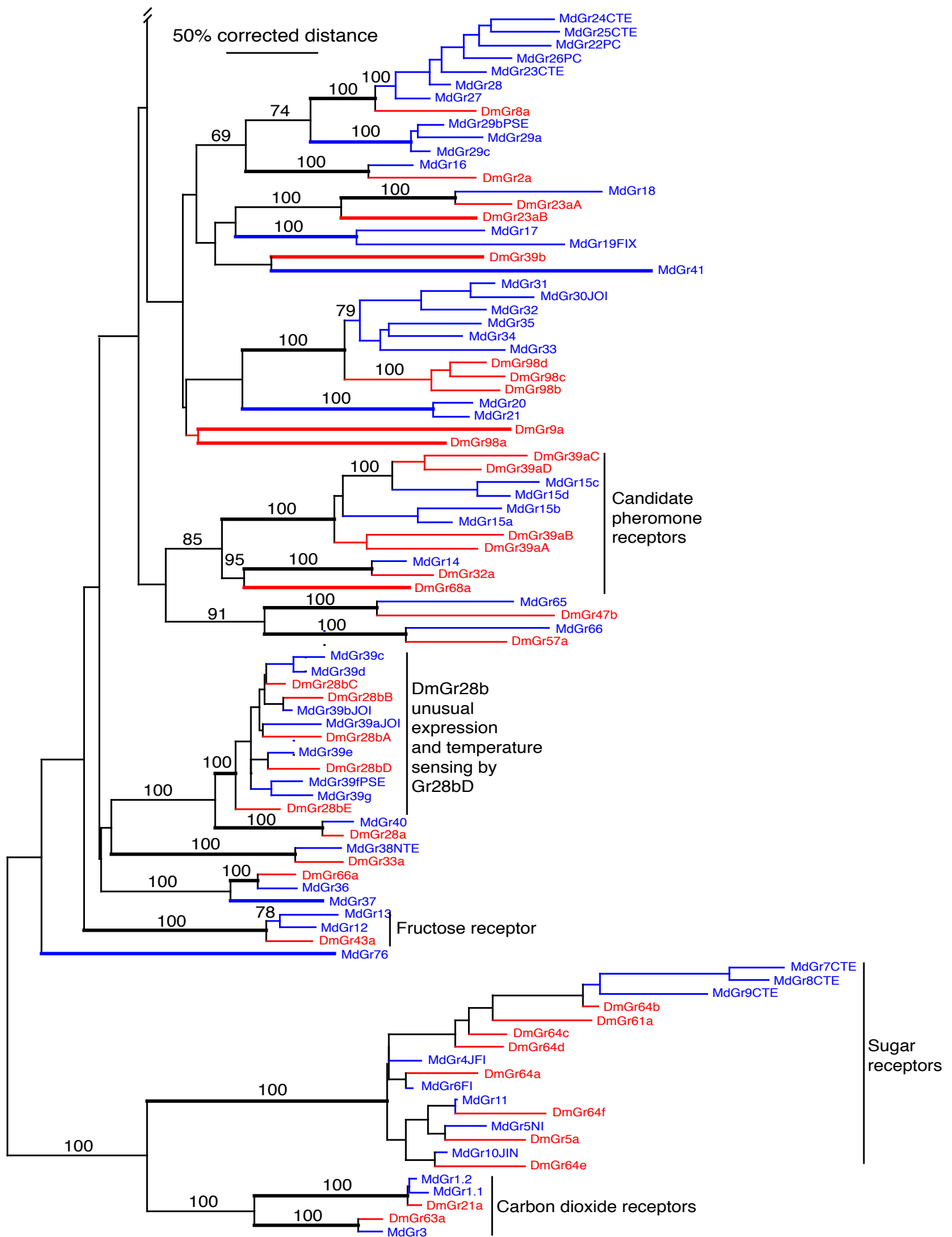

Supplement: Additional file 20: Figure S6. — Phylogenetic tree of the M. domestica and D. melanogaster GRs. This is a corrected distance tree rooted by declaring the distantly related and divergent carbon dioxide and sugar receptor subfamilies as the out-groups. The relationships within the sugar receptor subfamily are not accurate in this tree because many of these genes in M. domestica are only partially assembled. See Additional file 17 legend for other details. [file 13059_2014_466_MOESM20_ESM.pdf]
