# Supplementary material for: Genome of the house fly, Musca domestica L., a global vector of diseases with adaptations to a septic environment
Source: Genome Biol. 2014 Oct 14;15:466. doi: 10.1186/s13059-014-0466-3 (PMC4195910; doi:10.1186/s13059-014-0466-3)
Supplement: Additional file 18: Figure S4. — Phylogenetic tree of the M. domestica and D. melanogaster OBPs. This is a corrected distance tree and was rooted at the midpoint in the absence of a simple obvious out-group. See Additional file 17 legend for other details. [file 13059_2014_466_MOESM18_ESM.pdf]

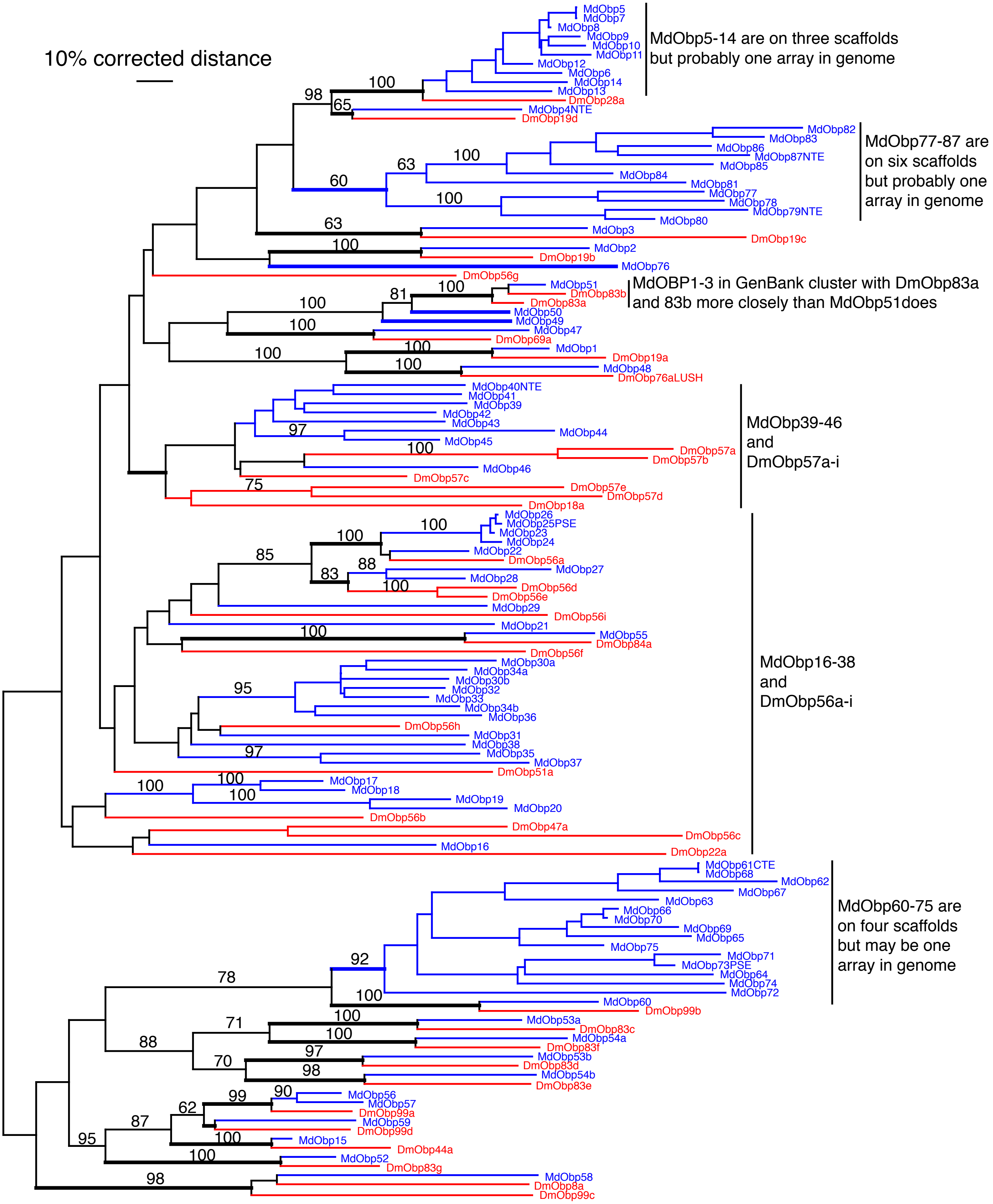

10% corrected distance
MdObp5
MdObp7
MdObp8
MdObp9
MdObp10
MdObp11
MdObp12
MdObp6
MdObp14
MdObp13
DmObp28a
MdObp4NTE
DmObp19d
MdObp5-14 are on three scaffolds but probably one array in genome
MdObp82
MdObp83
MdObp86
MdObp87NTE
MdObp85
MdObp84
MdObp81
MdObp77
MdObp78
MdObp79NTE
MdObp80
MdObp77-87 are on six scaffolds but probably one array in genome
MdObp3
DmObp19c
MdObp2
DmObp19b
MdObp76
DmObp56g
MdObp51
DmObp83b
DmObp83a
MdObp50
MdObp49
MdOBP1-3 in GenBank cluster with DmObp83a and 83b more closely than MdObp51does
MdObp47
DmObp69a
MdObp1
DmObp19a
MdObp48
DmObp76aLUSH
MdObp40NTE
MdObp41
MdObp39
MdObp42
MdObp43
MdObp44
MdObp45
DmObp57a
DmObp57b
MdObp46
DmObp57c
DmObp57e
DmObp57d
DmObp18a
MdObp39-46 and DmObp57a-i
MdObp26
MdObp25PSE
MdObp23
MdObp24
MdObp22
DmObp56a
MdObp27
MdObp28
DmObp56d
DmObp56e
MdObp29
DmObp56i
MdObp21
MdObp55
DmObp84a
DmObp56f
MdObp30a
MdObp34a
MdObp30b
MdObp32
MdObp33
MdObp34b
MdObp36
DmObp56h
MdObp31
MdObp38
MdObp35
MdObp37
DmObp51a
MdObp17
MdObp18
MdObp19
MdObp20
DmObp56b
DmObp47a
DmObp56c
MdObp16
DmObp22a
MdObp16-38 and DmObp56a-i
MdObp61CTE
MdObp68
MdObp62
MdObp67
MdObp63
MdObp66
MdObp70
MdObp69
MdObp65
MdObp75
MdObp71
MdObp73PSE
MdObp64
MdObp74
MdObp72
MdObp60
DmObp99b
MdObp60-75 are on four scaffolds but may be one array in genome
MdObp53a
DmObp83c
MdObp54a
DmObp83f
MdObp53b
DmObp83d
MdObp54b
DmObp83e
MdObp56
MdObp57
DmObp99a
MdObp59
DmObp99d
MdObp15
DmObp44a
MdObp52
DmObp83g
MdObp58
DmObp8a
DmObp99c
