# Supplementary material for: Genome of the house fly, Musca domestica L., a global vector of diseases with adaptations to a septic environment
Source: Genome Biol. 2014 Oct 14;15:466. doi: 10.1186/s13059-014-0466-3 (PMC4195910; doi:10.1186/s13059-014-0466-3)
Supplement: Additional file 22: Table S15. — Locations of putative microRNA and their predicted precursor hairpins from the M. domestica genome. [file 13059_2014_466_MOESM22_ESM.docx]

**Table S15 Locations of putative microRNA and their predicted precursor hairpins from the *M. domestica* genome**

| **miRNA with the same seed^†^** | **Supercontig:position:strand** | **consensus mature sequence (5' to 3')** | **consensus star sequence (5' to 3')** | **consensus precursor sequence (5' to 3')** |
| --- | --- | --- | --- | --- |
| dme-bantam-3p | NW_004768998:4924-4981:+ | ugagaucauuuugaaagcugau | cgguuuucgauuugauuugacu | cgguuuucgauuugauuugacuuauuuuuacaaagugagaucauuuugaaagcugau |
| dme-let-7-5p | NW_004764666:411807-411871:- | ugagguaguagguuguauagu | uauacaacgugcuagcuuucu | ugagguaguagguuguauaguagucucacuaaaauuaacauacuauacaacgugcuagcuuucu |
| dme-miR-1-5p | NW_004769106:51098-51160:- | uggaauguaaagaaguauggag | ccaugcuuccuugcauucaaua | ccaugcuuccuugcauucaauaguauuuaauucaaucauauggaauguaaagaaguauggag |
|  | NW_004769106:55600-55662:- | uggaauguaaagaaguauggag | ccaugcuuccuugcauucaaua | ccaugcuuccuugcauucaauaguauuuaauucaaucauauggaauguaaagaaguauggag |
| dme-miR-10-5p | NW_004764481:26778-26838:+ | acccuguagauccgaauuuguu | caaauucgguucuagagagguuu | acccuguagauccgaauuuguucugauauuugaaugacaaauucgguucuagagagguuu |
| dme-miR-100-5p | NW_004764666:412113-412174:- | aacccguaaauccgaacuugug | caagaccggcauuaugggaguc | aacccguaaauccgaacuugugcuauucuauugagguuacaagaccggcauuaugggaguc |
| dme-miR-1000-5p | NW_004764693:293358-293422:- | auauuguccugucacagcagu | ugcugggccggggcauuaaca | auauuguccugucacagcaguauguuuuauuuaaauauuuuacugcugggccggggcauuaaca |
| dme-miR-124-5p | NW_004765951:122178-122231:+ | gguauccacuguaggccuauaug | uaauagugcugcgguauacguu | uaauagugcugcgguauacguuuuucuccugguauccacuguaggccuauaug |
| dme-miR-125-3p | NW_004764666:411345-411401:- | ucacaaguuuugaucuccgguau | acuguuuaaaauuuuuugca | ucacaaguuuugaucuccgguauuggccgcaagcuuacuguuuaaaauuuuuugca |
| dme-miR-125-5p | NW_004764666:411378-411438:- | ucacaaguuuugaucuccgguau | ucccugagacccuaacuuguga | ucccugagacccuaacuugugacuuauuaucacaguuucacaaguuuugaucuccgguau |
| dme-miR-133-3p | NW_004775249:47185-47231:- | uugguccccuucaaccagcugu | agugguuggcaucaaccgaaa | uugguccccuucaaccagcuguagcagugguuggcaucaaccgaaa |
|  | NW_004775249:47209-47272:- | uugguccccuucaaccagcugu | agcugguuggcaucgggucagau | agcugguuggcaucgggucagaucgguuuauuucuaaguauuugguccccuucaaccagcugu |
| dme-miR-137-3p | NW_004768760:1823797-1823858:+ | uauugcuugagaauacacguag | acgcguauucuuagguuacuaac | acgcguauucuuagguuacuaacauguauuuguuauuguuauugcuugagaauacacguag |
| dme-miR-13a-3p | NW_004764810:44805-44863:- | uaucacagccauuuugaugag | cuucaagauguugugaaaug | cuucaagauguugugaaauguuaauauuuuucgaucauaucacagccauuuugaugag |
| dme-miR-13b-3p | NW_004773138:32968-33030:- | uaucacagccauuuugacgagu | gcgucgaauugguugugaauuaug | gcgucgaauugguugugaauuauguugcauuucuuauacauaucacagccauuuugacgagu |
| dme-miR-184-5p | NW_004764535:488364-488426:- | acuggacggagaacugauaagggc | ccuuaucauucucucgccccg | ccuuaucauucucucgccccgugugauuuuucuauacgacuggacggagaacugauaagggc |
| dme-miR-210-5p | NW_004764625:425404-425462:+ | uugugcgugugacagcggcu | agcugcuggccacugcacaagau | agcugcuggccacugcacaagauuagauuagaugacucuugugcgugugacagcggcu |
| dme-miR-219-5p | NW_004764604:250586-250651:- | ugauuguccaaacgcaauucu | gguugugagugggcaucguc | ugauuguccaaacgcaauucuuguuuaaaauuuaaauauuucaaggguugugagugggcaucguc |
| dme-miR-263b-5p | NW_004764589:548784-548848:+ | cuuggcacugggagaauucac | gguucuuugggugucaaaug | cuuggcacugggagaauucacaguuguuaucuguuagauucugugguucuuugggugucaaaug |
| dme-miR-275-3p | NW_004764949:144589-144653:- | ucagguaccugaaguagcgcgcg | cgcgcuaaucagagaccggggcu | cgcgcuaaucagagaccggggcugaauauugaaaagugcagucagguaccugaaguagcgcgcg |
| dme-miR-276a-3p | NW_004764586:87861-87924:- | agcgagguauagaguuccua | uaggaacuucauaccgugcucu | agcgagguauagaguuccuacgugcauuaauuugauaucuguaggaacuucauaccgugcucu |
| dme-miR-276b-3p | NW_004764586:456608-456668:- | uaggaacuuaauaccgugcucu | agcgagguauagaguuccuacg | agcgagguauagaguuccuacguuccuuuuccaauucguaggaacuuaauaccgugcucu |
| dme-miR-277-3p | NW_004764644:282896-282960:+ | uaaaugcacuaucugguacgac | cguaucaggggugacauuugca | cguaucaggggugacauuugcacuggguuuuuaaguaguuuguaaaugcacuaucugguacgac |
| dme-miR-278-3p | NW_004772082:239709-239771:- | ucggugggacuuucguccguuu | ccggaugaugguccucaacgacc | ccggaugaugguccucaacgaccguuuuaauuuauacuggucggugggacuuucguccguuu |
| dme-miR-279-3p | NW_004759241:10443-10502:- | ugacuagauccacacucau | gagugaggguccaguguuucaca | gagugaggguccaguguuucacauuucauucuaaauauugugacuagauccacacucau |
| dme-miR-281-1-5p | NW_004765100:103135-103202:+ | ugucauggaauugcucucuuugu | aaagagagcuguccgucgacagu | aaagagagcuguccgucgacaguccaguuaaaccaauauaauacugucauggaauugcucucuuugu |
| dme-miR-282-5p | NW_004772416:16481-16564:+ | uagccucuacuaggcuuugucugu | agacauagccuaaaagagguuagg | uagccucuacuaggcuuugucuguaacuuggcuugaagcucgcuucaaauauauuauccagacauagccuaaaagagguuagg |
| dme-miR-283-5p | NW_004764766:39501-39575:- | aaauaucagcugguaauucu | aauuucaguugguauggau | aaauaucagcugguaauucugggauuuauuuacaaaaaacauuggcauuccccggaauuucaguugguauggau |
| dme-miR-286-3p | NW_004765279:69499-69564:+ | ugacuagaccgaacacucgugcu | ggcgauugucgguauagucucu | ggcgauugucgguauagucucuguguuuguuuuaggagucagugacuagaccgaacacucgugcu |
| dme-miR-2a-2-5p | NW_004764931:250422-250482:- | uaucacagccagcuuugaugagcu | ccucaucaagugguugugaua | ccucaucaagugguugugauauggauuaucaacgcauaucacagccagcuuugaugagcu |
| dme-miR-2a-3p | NW_004764810:45540-45608:- | ucacagccagcuuugaugagca | gucaucaaaaagggcuguuguaugaua | gucaucaaaaagggcuguuguaugauauuuugcauucgauugaguaucacagccagcuuugaugagca |
| dme-miR-2b-3p | NW_004764108:604243-604307:- | uaucacagccagcuuugaggagc | ucuucagaggggcugugaaaug | ucuucagaggggcugugaaauguuuugacuuuaaauaggcauaucacagccagcuuugaggagc |
| dme-miR-305-5p | NW_004764949:144379-144439:- | auuguacuucaucaggugcucugg | ccggcacauguugaaguacauuca | auuguacuucaucaggugcucuggugcauuuacaacccggcacauguugaaguacauuca |
| dme-miR-306-5p | NW_004766699:2246-2310:+ | ucagguacuuagugacucucaa | gggguucacuuuguaccuugcc | ucagguacuuagugacucucaauguuauuuuagaaaaaaucugggguucacuuuguaccuugcc |
|  | NW_004766699:2194-2268:+ | ucagguacuuagugacucucaa | gggauguccuaucgaaacuugauu | gggauguccuaucgaaacuugauuuucuuucaucugaucggaaaacuuuuguucagguacuuagugacucucaa |
| dme-miR-307a-5p | NW_004765350:41588-41658:- | acucacucaaccugggugugaug | cacaaccuccuugagugagcga | acucacucaaccugggugugauguguguauuucguuuuggcuauccaucacaaccuccuugagugagcga |
| dme-miR-307b-5p | NW_004765350:41593-41660:+ | ucacucaaggagguugugaugg | ucacacccagguugagugaguc | ucacucaaggagguugugauggauagccaaaacgaaauacacacaucacacccagguugagugaguc |
| dme-miR-308-3p | NW_004764512:984210-984273:- | aaucacaggauuauacugugag | cgcaguauauuuuuguguuuuug | cgcaguauauuuuuguguuuuuguuuauuuuuuuaaaaucaaaucacaggauuauacugugag |
| dme-miR-315-5p | NW_004764745:316123-316187:- | uuuugauuguugcucagaaagcc | cuuucgagcugcaauuaaaaac | uuuugauuguugcucagaaagcccucauuaaauaucaguuggcuuucgagcugcaauuaaaaac |
| dme-miR-316-5p | NW_004764513:1291695-1291754:+ | ugucuuuuuccgcuuacuggcg | acaggaaagggaaaaaggcgca | ugucuuuuuccgcuuacuggcgcuaauauuugagacgacaggaaagggaaaaaggcgca |
| dme-miR-31a-5p | NW_004764657:423817-423886:- | ggcaagaugucggcauagcuga | cgcuaugcugcaucuagucaa | ggcaagaugucggcauagcugagaauuuauuuuuguuuuuugaaaggccgcuaugcugcaucuagucaa |
| dme-miR-31b-5p | NW_004764786:163748-163830:+ | ggcuaugccucaucuagucaau | uggcaagaugucggaauagcug | uggcaagaugucggaauagcugauuguuucuuuauaaggcuaacaaaauuugcguuuuacggcuaugccucaucuagucaau |
| dme-miR-375-3p | NW_004764064:166955-167023:- | uuuguucguuuggcuuaaguua | acuugagccaagugaauacaaaca | acuugagccaagugaauacaaacauaaucaaaggcauuucaauaaguuuguucguuuggcuuaaguua |
| dme-miR-4-3p | NW_004765279:69667-69726:+ | auaaagcuagacaaccauuga | aguggucguuuaggcuuuaguga | aguggucguuuaggcuuuagugauaacauuuuggacucauaaagcuagacaaccauuga |
| dme-miR-5-5p | NW_004765279:70067-70130:+ | uaucacagugauuuuccuuuaua | aaaggaacgaucguugugauaug | aaaggaacgaucguugugauauguuucuuuuuuauagacauaucacagugauuuuccuuuaua |
| dme-miR-6-3p | NW_004765279:70628-70693:+ | uaucacaguggcuguucuuuuu | agggaacaguggcagugaugua | agggaacaguggcagugauguauuccauaugaugaaauauuuauaucacaguggcuguucuuuuu |
|  | NW_004765279:70432-70502:+ | uaucacaguggcuguucuuuuu | agagaacgacugcuugugaugua | agagaacgacugcuugugauguacucucuuuuugcccuagcauaguuauaucacaguggcuguucuuuuu |
|  | NW_004765279:70247-70317:+ | uaucacaguggcuguucuuuuu | agagaaugguggcugugaugua | agagaaugguggcugugauguaucucacauacaauucucuucuauguauaucacaguggcuguucuuuuu |
| dme-miR-7-5p | NW_004764996:93527-93604:+ | uggaagacuagugauuuuguuguu | caaaucaaaacgcuucuucuacc | caaaucaaaacgcuucuucuacccccccuccaucauucacgauacucauuguuuggaagacuagugauuuuguuguu |
|  | NW_004764996:93580-93642:+ | uggaagacuagugauuuuguuguu | caauaaaucccuugucuucuua | uggaagacuagugauuuuguuguuuugcuuuagauuuaaacaauaaaucccuugucuucuua |
| dme-miR-8-5p | NW_004764799:46794-46855:+ | uaauacugucagguaaagaugu | caucuuaccgggcagcauuaga | caucuuaccgggcagcauuagauuauauugaaauuucucuaauacugucagguaaagaugu |
| dme-miR-87-3p | NW_004765198:248867-248932:- | uugagcaaaauuucaggugug | cauuuguaccguuuauuu | uugagcaaaauuucaggugugugagaaauucgucuuaauauauucuacauuuguaccguuuauuu |
|  | NW_004765198:248911-248970:- | uugagcaaaauuucaggugug | caccugugucuugcugaacc | caccugugucuugcugaaccauuuccuuauguagcagguugagcaaaauuucaggugug |
|  | NW_004765198:249092-249157:- | uugagcaaaauuucaggugug | caccuguauuuugcugaacc | caccuguauuuugcugaaccguuuccauucucuucuauuuccgguugagcaaaauuucaggugug |
| dme-miR-927-5p | NW_004771871:37870-37931:- | caaagcguuuggauucugaaac | uuuagaauuccuacgcuuuacc | uuuagaauuccuacgcuuuaccuuguuauaugaaaauggcaaagcguuuggauucugaaac |
| dme-miR-929-5p | NW_004764397:586-643:+ | auugacucuaguagggagucc | cucccuaacggagucagauug | auugacucuaguagggaguccuuuuuauacgagugacucccuaacggagucagauug |
| dme-miR-932-5p | NW_004765303:120987-121052:- | ucaauuccguagugcauugcag | gcaagcauuguggaaaugaug | ucaauuccguagugcauugcaguuuguugaacuccgaaauuacugcaagcauuguggaaaugaug |
| dme-miR-957-3p | NW_004765239:22258-22322:+ | ugaaaccguccaaaacugaggc | cuuagcuuugggcggguuuuggu | cuuagcuuugggcggguuuugguguaugauaaguuaaccuauugaaaccguccaaaacugaggc |
| dme-miR-958-3p | NW_004764762:147433-147507:- | ugagauucuucuauucuacuuu | aguagaguagggggcuccucaca | aguagaguagggggcuccucacaugucuuucucaagaauuuuuugauauuugugagauucuucuauucuacuuu |
| dme-miR-965-3p | NW_004765285:98007-98097:+ | uaagcguauagcuuuuccccuu | gggguaaugcuguacguuguau | gggguaaugcuguacguuguauguuuuguuuauuugaaaauaucuaaacuccguuuauaugauaugcauaagcguauagcuuuuccccuu |
| dme-miR-970-3p | NW_004764635:370856-370919:- | ucauaagacacacgcggcuau | aguuggcguuuguuuuauuuggua | aguuggcguuuguuuuauuugguaguguuucgaacuaggcuaucauaagacacacgcggcuau |
| dme-miR-971-3p | NW_004764501:427500-427568:- | uugguguuacuucuuacaguga | augcgcagggucacauccaca | uugguguuacuucuuacagugauggcuggugaauuaagcagcaaauuaugcgcagggucacauccaca |
|  | NW_004764501:427546-427613:- | uugguguuacuucuuacaguga | gcuguaagaggauagcaacaaagc | gcuguaagaggauagcaacaaagcuguuuaaauaucugauaucgcuugguguuacuucuuacaguga |
| dme-miR-981-3p | NW_004764467:256073-256137:+ | uucguugucgacgaaaccug | gguuucgcaaacagcggucu | gguuucgcaaacagcggucuguauuuaaugcuauuugacauuaguucguugucgacgaaaccug |
| dme-miR-987-5p | NW_004764477:189985-190060:- | uaaaguaaauagucuggauugaug | ucaacaggcauuuacuuugac | uaaaguaaauagucuggauugaugaauuugcccaugggcuuugaaaauuguucaucaacaggcauuuacuuugac |
| dme-miR-988-3p | NW_004764620:1346671-1346728:+ | cccuuguugcaaaccucacgc | gugugauuugaagcaagguga | gugugauuugaagcaaggugauuuguauuugaaauccccuuguugcaaaccucacgc |
| dme-miR-989-3p | NW_004764904:311053-311133:- | ugugaugugacguagugg | aucuucgucuuaaacaug | ugugaugugacguaguggaacauuccugacguucaaaaaaaccaccaucauaaacaucauccaucuucgucuuaaacaug |
|  | NW_004764904:311115-311164:- | ugugaugugacguagugg | ucugcucaaauguacuuu | ucugcucaaauguacuuuaauuuauauuucaugugaugugacguagugg |
| dme-miR-993-5p | NW_004764550:451785-451874:+ | uacccuguaguuccgggcuuuu | gaagcucgucucuacagguaucu | uacccuguaguuccgggcuuuuguuuuaaaggcauuuuguucuaugaaugcauuucaugcuuaucagaagcucgucucuacagguaucu |
| dme-miR-994-5p | NW_004765168:123908-123984:- | cuaaggaaauaguagccgugau | cacaguugcuguuucuuuuagau | cuaaggaaauaguagccgugauuuuauugauccacagcaaacaucucacauaucacaguugcuguuucuuuuagau |
| dme-miR-996-5p | NW_004766187:11379-11444:- | ugacuagauuucaugcucgucu | gcgaacauggaucuagugcacg | gcgaacauggaucuagugcacgguuuauucauaaucaaguucgugacuagauuucaugcucgucu |
|  | NW_004773439:39727-39792:- | ugacuagauuucaugcucgucu | gcgaacauggaucuagugcacg | gcgaacauggaucuagugcacgguuuauucauaaucaaguucgugacuagauuucaugcucgucu |
| dme-miR-998-3p | NW_004765529:98710-98752:+ | uagcaccaugagauucagc | ugaauuucaaagcaca | uagcaccaugagauucagcucuggcgugaauuucaaagcaca |
| dme-miR-998-5p | NW_004765529:98552-98638:+ | acugaauucucgugggucugca | caaccucaugaaaauuauaaca | caaccucaugaaaauuauaacaacaaugugaacuuguaacuaaaagaaaaaaauucauuuuggaacugaauucucgugggucugca |
| dme-miR-999-3p | NW_004764466:163925-163986:- | uguuaacuguaagacugugucu | acauagucguacagaaaauguu | acauagucguacagaaaauguuguguuguucgauuccaauguuaacuguaagacugugucu |
| dme-miR-9a-5p | NW_004764933:72355-72416:- | ucuuugguuaucuagcuguauga | uaaagcuagcuuaccgaaguua | ucuuugguuaucuagcuguaugaguucuauaugacgucauaaagcuagcuuaccgaaguua |
| dme-miR-9b-5p | NW_004766699:4783-4845:+ | uagagcuuuauuaccaaaaacc | ucuuuggugauuuuagcuguaug | ucuuuggugauuuuagcuguaugguguauuucagauuccauagagcuuuauuaccaaaaacc |
| dme-miR-9c-5p | NW_004766699:1367-1446:+ | uaaagcuuuuauaccaaagcuc | ucuuugguauucuagcuguaga | ucuuugguauucuagcuguagauuuucuuguuuggaaaauaauuuuuaaauaucaucuaaagcuuuuauaccaaagcuc |
| dme-miR-iab-4-5p | NW_004764902:279571-279629:+ | cgguauaccuucaguauacguaac | acguauacugaauguauccuga | acguauacugaauguauccugaguguauccuauccgguauaccuucaguauacguaac |
| dps-miR-317 | NW_004764644:224369-224438:+ | ugaacacagcuggugguaucu | auacacccugugcucgcu | auacacccugugcucgcuuugaaguuuaagcuaacaauuugauaucagugaacacagcuggugguaucu |

^†^miRbase release 20, http://www.mirbase.org
