# Supplementary material for: Genome of the house fly, Musca domestica L., a global vector of diseases with adaptations to a septic environment
Source: Genome Biol. 2014 Oct 14;15:466. doi: 10.1186/s13059-014-0466-3 (PMC4195910; doi:10.1186/s13059-014-0466-3)
Supplement: Additional file 23: Table S16. — MicroRNA machinery and genes predicted to be involved in exogenous double-stranded RNA uptake in M. domestica. [file 13059_2014_466_MOESM23_ESM.docx]

**Table S16 MicroRNA machinery and genes predicted to be involved in exogenous dsRNA uptake in *Musca domestica***

| **Predicted gene function annotation^*^** | **Genome locus** | **XM number** | **XP number** |
| --- | --- | --- | --- |
| **Nuclear export and pri-miRNA processing** |  |  |  |
| exportin-5-like | LOC101895229 | XM_005192027 | XP_005192084 |
| GTP-binding nuclear protein Ran-like | LOC101897713 | XM_005191880 | XP_005191937 |
| ribonuclease 3-like (drosha-like) | LOC101895496 | XM_005186920 | XP_005186977 |
| DGCR8-like isoform X1 | LOC101893834 | XM_005191474 | XP_005191531 |
| isoform X2 |  | XM_005191475 | XP_005191532 |
| isoform X3 |  | XM_005191476 | XP_005191533 |
| **Hairpin processing and RISC loading** |  |  |  |
| protein argonaute-1-like isoform X1 | LOC101897720 | XM_005175251 | XP_005175308 |
| isoform X2 |  | XM_005175252 | XP_005175309 |
| isoform X3 |  | XM_005175253 | XP_005175310 |
| isoform X4 |  | XM_005175254 | XP_005175311 |
| protein argonaute-2-like isoform X1 | LOC101890924 | XM_005177121 | XP_005177178 |
| isoform X2 |  | XM_005177122 | XP_005177179 |
| isoform X3 |  | XM_005177123 | XP_005177180 |
| isoform X4 |  | XM_005177124 | XP_005177181 |
| isoform X5 |  | XM_005177125 | XP_005177182 |
| protein argonaute-2-like isoform X1 | LOC101892105 | XM_005183818 | XP_005183875 |
| isoform X2 |  | XM_005183819 | XP_005183876 |
| protein argonaute-3-like | LOC101895955 | XM_005183499 | XP_005183556 |
| endoribonuclease Dcr-1-like isoform X1 | LOC101896691 | XM_005179866 | XP_005179923 |
| isoform X2 |  | XM_005179867 | XP_005179924 |
| endoribonuclease Dcr-1-like isoform X1 | LOC101893656 | XM_005187081 | XP_005187138 |
| isoform X2 |  | XM_005187082 | XP_005187139 |
| RISC-loading complex subunit isoform X1 | LOC101888961 | XM_005188080 | XP_005188137 |
| isoform X2 |  | XM_005188081 | XP_005188138 |
| **Putatative exogenous dsRNA uptake genes** |  |  |  |
| eater^1^ | LOC101895867 | XM_005192179 | XP_005192236 |
| eater^2^ | LOC101896200 | XM_005192181 | XP_005192238 |
| nibbler^3^ | LOC101897839 | XM_005175553 | XP_005175610 |

^*^Annotation from the *M. domestica* Gnomon genome annotation, unless otherwise indicated

^1^*M.domestica* Gnomon annotation: uncharacterized protein

^2^*M.domestica* Gnomon annotation: fibrillin-2-like

^3^*M.domestica* Gnomon annotation: exosome component 10-like
